# Photoinduced *β*-fragmentation of aliphatic alcohol derivatives for forging C–C bonds

**DOI:** 10.1038/s41467-022-35249-7

**Published:** 2022-12-02

**Authors:** Yiman Gao, Jie Liu, Cong Wei, Yan Li, Kui Zhang, Liangliang Song, Lingchao Cai

**Affiliations:** Jiangsu Co-Innovation Center of Efficient Processing and Utilization of Forest Resources, International Innovation Center for Forest Chemicals and Materials, Jiangsu Province Key Laboratory of Green Biomass Based Fuels and Chemicals, College of Chemical Engineering, Nanjing Forestry University, Nanjing, 210037 China

**Keywords:** Synthetic chemistry methodology, Photocatalysis

## Abstract

Alcohols are ubiquitous in chemistry and are native functionalities in many natural products and bioactive molecules. As such, a strategy that utilizes hydroxy-containing compounds to develop bond disconnection and bond formation process would achieve molecular diversity. Herein we utilize bench-stable *N*-alkoxyphthalimides prepared from alcohols to couple with glycine derivatives via radical process under visible light irradiation, providing a variety of unnatural amino acid (UAA) and peptide derivatives. The approach allows to rapidly deconstruct molecular complexity via *β*-fragmentation such as saclareolide, *β*-pinene and camphor and provides products with unique scaffolds, which show inhibition toward the pathogenic fungi growth.

## Abstract (web summary)

Transformations of alcohols, which are ubiquitous in chemistry and are native functionalities in many natural products and bioactive molecules, are cornerstones of organic synthesis. Here the authors describe photocatalyzed cross-couplings of activated alcohols with α-amino acids, providing a direct approach for a variety of unnatural amino acids.

## Introduction

**Fig. 1:**
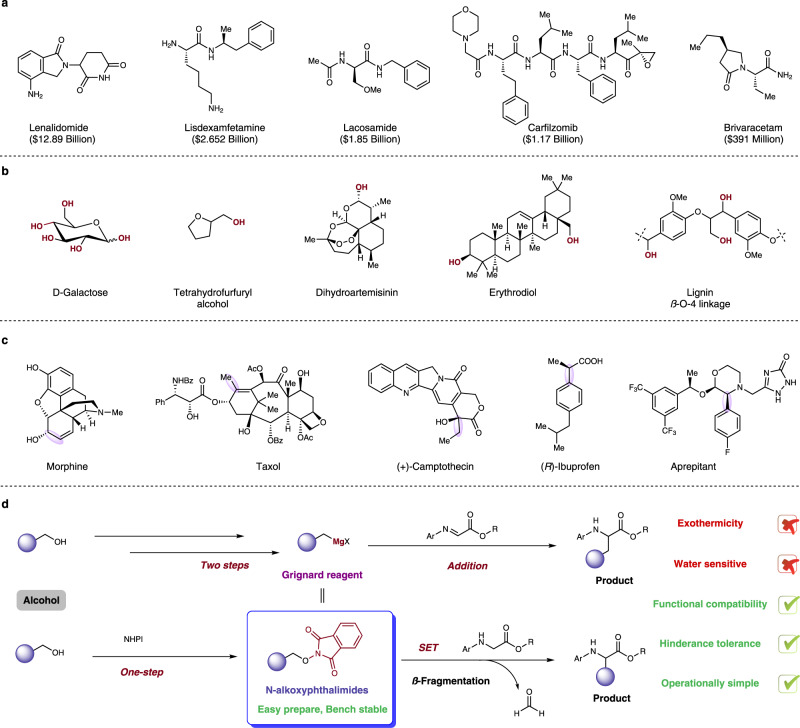
Project background and design plan. **a** Various drugs derived from *α*-amino acid; **b** Representative biomass derivatives, pharmaceuticals, and natural products containing hydroxy functional group; **c** Pharmaceutical and natural products syntheses featuring the Grignard reaction (formed bonds highlighted in purple); **d** Classical Grignard transformation from alcohols and our *N*-alkoxyphthalimide-based alternative approach. NHPI, N-hydroxyphthalimide, SET single-electron transfer.

Around 50 of 2021s top-200 best-selling small molecule pharmaceuticals contain *α*-amino acid-derived scaffolds as exemplified by lenalidomide, lisdexamfetamine, lacosamide, carfilzomib, and brivaracetam (Fig. 1a)^1^. Meanwhile, UAAs have been genetically encoded in various cells and model organisms, investigating the structure and dynamics of proteins, to study their interactions and functions, to control their activities in living cells, as well as to introduce novel functions in proteins unachievable in nature^2^. As such, amino acids are valuable and indispensable building blocks in the manufacture of a wide range of pharmaceuticals and biological applications.

Aliphatic alcohols are found ubiquitously in pharmaceuticals, agricultural chemicals, biomass degradation products, and many bioactive natural products (Fig. 1b). Classically, the Grignard reaction is an often adopted method for utilization of alcohols with an addition toward carbonyl-derived substrates, which is a prominent textbook process to form new C–C bonds and widely applied in synthetic and pharmaceutical chemistry for complex natural product syntheses^3–6^ and drug development^7,8^ since its discovery in 1912 (Fig. 1c)^9–12^. The conversion toward Grignard reagents from alcohols typically requires two-step procedures: Appel reaction and magnesium metal reduction. From a safety perspective, the preparation of Grignard reagents with magnesium metal is often hard to control, and strongly exothermic as the subsequent Grignard addition. Meanwhile, Grignard reaction suffer from low functional group tolerance and hazardous wastes. Furthermore, the bond formation becomes less efficient when steric hindrance is amplified, limiting its broad utility. Given the potential applications of Grignard-type reactions, especially in complex molecule syntheses and the importance of UAAs, the development of a convenient protocol for C–C bond formation from ubiquitous alcohol precursors under mild conditions is an enduring demand and fulfills the requirement of chemical sustainability.

Recently, radical approaches for selective functional transformations under photocatalyzed conditions experience a surge of developments, which switch the traditional two-electron approach to a single-electron approach, thus achieving various challenging transformations, which are unattainable by classical methods. The photo-induced alkoxyl radical has emerged as a versatile intermediate and has been applied in a wide variety of synthetic transformations^13–15^. Proton-coupled electron transfer (PCET)^16,17^, ligand-to-metal charge transfer (LMCT)^18–23^, transition metal mediated homolysis^24–31^, and in situ generation of O–X species^32,33^, have appeared as powerful strategies to enable the alkoxyl radical-based hydrogen atom transfer^34^, *β*-fragmentation^35^, and alkene addition^36^. Meanwhile, alkoxy radical generation from *N*-alkoxyphthalimides triggered by a transient reductant that is generated by low-intensity irradiation with visible light is used preferred as alternative^37–41^, among many other oxygen-heteroatom bonds precursors such as nitrites^42^, nitrates^43^, hypohalites^44^, and sulphenates^45^, etc. due to its stability and accessibility. Chen^37^, Tang^38^, Aggarwal^39,41^, and Martin^40^ groups, respectively, developed attractive methodologies to achieve C(sp^3^)–C(sp^3^), C(sp^3^)–B, and C(sp^3^)–C(sp^2^) bond formation from *N*-alkoxyphthalimides (NHPI) precursors via alkoxyl radical.

Herein, we devised a strategy between bench stable *N-*alkoxyphthalimides prepared from alcohols and glycine derivatives to form C–C bonds via a redox-neutral radical–radical coupling process as alternative to Grignard-type addition, bypassing the need to utilize harsh organometallic reagents and minimizing the adverse environmental impact (Fig. 1d).

## Results

### Reaction optimization

We started our model reaction using ethyl *p*-tolylglycinate (**1a**) and tetrahydrofurfuryl alcohol-derived *N*-alkoxyphthalimide **2a** (Table 1). Gratifyingly, the desired product **3** was isolated in 92% (d.r. = 1: 1) when the reaction was carried out in DMSO at 60 °C for 5 h with blue LED irradiation and 4CzlPN as a photocatalyst (Table 1, entry 1). Other photocatalysts such as TTP, eosin Y, Acr^+^-Mes, and rhodamine B were proved ineffective for this transformation (Table 1, entries 2–5). 60 °C and DMSO were proved to be the optimal temperature and solvent for this reaction (Table 1, entries 6–9). During the optimization process, we found higher reaction concentration would diminish the reaction efficiency and the 0.03 M gave the best result (Table 1, entry 10). Control experiments also demonstrated that the reaction could not occur in the absence of either the photocatalyst or light (Table 1, entries 11–12).

**Table 1:** Optimization of the reaction conditions


Entry	Variation from the standard conditions	Yield (%)^a^
1	None	92^b^
2	TTP instead of 4CzIPN	<5
3	Eosin Y instead of 4CzIPN	<5
4	[Acr-Mes] ^+^ (BF_4_^−^) instead of 4CzIPN	<5
5	Rhodamine B instead of 4CzIPN	<5
6	80 °C instead of 60 °C	70
7	40 °C instead of 60 °C	69
8	DMA instead of DMSO	75
9	MeCN instead of DMSO	56
10	0.1 M instead of 0.03 M	56
11	No photocatalyst	*nd*
12	No light	*nd*


Standard conditions: 5 mol% 4CzIPN, 0.2 mmol **1a**, 0.1 mmol **2a**, DMSO (0.03 M), 15 W Blue LEDs, 60 °C, 5 h.

*DMSO* dimethyl sulfoxide, *DMA* dimethylacetamide, *MeCN* acetonitrile, *nd* not detected.

^a^Isolated yield.

^b^d.r. = 1: 1.

### Evaluation of substrate scopes

**Fig. 2:**
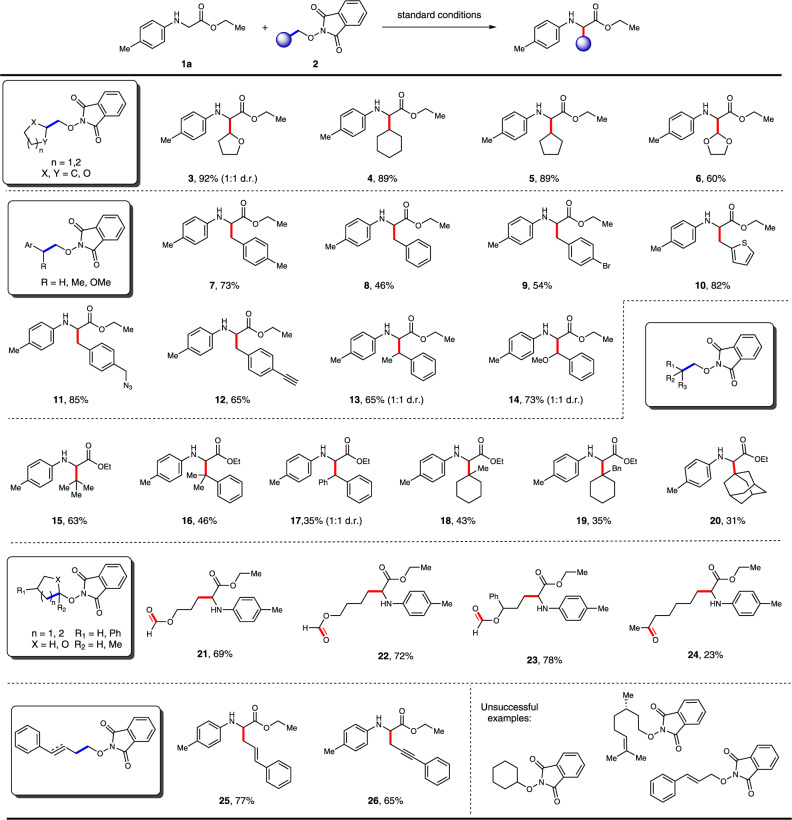
Scope of *N*-alkoxyphthalimides (broken bonds highlighted in blue and formed bonds highlighted in red). Conditions: All reactions were carried out with 4CzIPN (5 mol%), **1a (**0.2 mmol), **2** (0.1 mmol), and DMSO (0.03 M) irradiated under 15 W Blue LEDs at 60 °C.

**Fig. 3:**
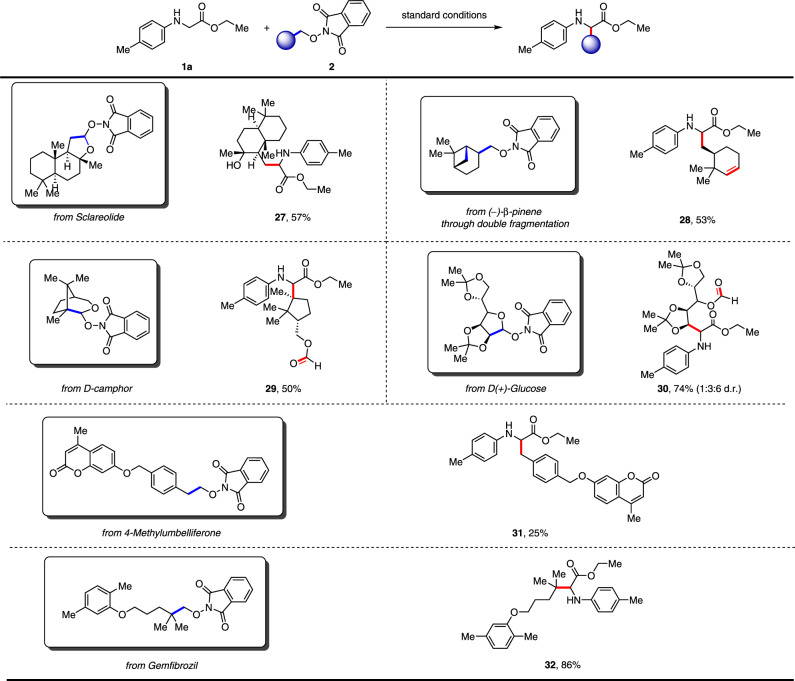
*N*-alkoxyphthalimides from nature sources. Conditions: All reactions were carried out with 4CzIPN (5 mol%), **1a** (0.2 mmol), **2** (0.1 mmol), and DMSO (0.03 M) irradiated under 15 W Blue LEDs at 60 °C.

Having established the optimal reaction conditions, we proceeded to probe the scope of this transformation. We first evaluated this method with different *N*-alkoxyphthalimides. As shown in Fig. 2, various five and six-membered cyclic *N*-alkoxyphthalimides smoothly proceeded this transformation to provide the desired products **3**–**6** in good to excellent yields (60–92%). Benzylic and heteroaryl benzylic *N*-alkoxyphthalimides were also examined and found to be good substrates, regardless of the substituent on the aromatic ring (**7**–**10**, 46–82%). Notably, azido and alkynyl groups were well tolerated in the reaction (**11** and **12**, 85 and 65%), which provided an opportunity for further “click bioconjugation”. Benzylic substrates with an additional substituent on the benzyl position were also compatible with this transformation, thus delivering the corresponding UAAs **13** and **14** in 65 and 73% yields, respectively. In order to prove the efficiency of this transformation in consideration of the steric effect, varieties of sterically bulky substrates were tested and synthetically useful yields (31–63%) were obtained, which offered complementarity to Grignard-type addition (**15**–**20**). It is worthy to mention that these sterically bulky substrates were easily prepared from sterically less hindered primary alcohols. *N*-alkoxyphthalimides derived from hemiacetal and cyclic tertiary alcohol reacted smoothly under the standard conditions and afforded ring-opened formate and ketone tethered products (**21**–**24**), whose functionalities could not be compatible with Grignard-type reaction conditions. Allylic and propargylic radicals could be generated facilely from corresponding precursors and furnished the desired products in 77 and 65% yield, respectively (**25** and **26**). To our disappointed, *N*-alkoxyphthalimides which could generate alkenyl radical, aldehyde tethered products, and radical intermediates via 5*-exo-trig* process, failed to give any products. In addition, more complex scaffolds derived from sclareolide, *β*-pinene, *D*-camphor, glucose, coumarin, and gemfibrozil precursors were screened and offered simplified products in moderate to high yields (**27**–**32**, 25–86%) (Fig. 3).

**Fig. 4:**
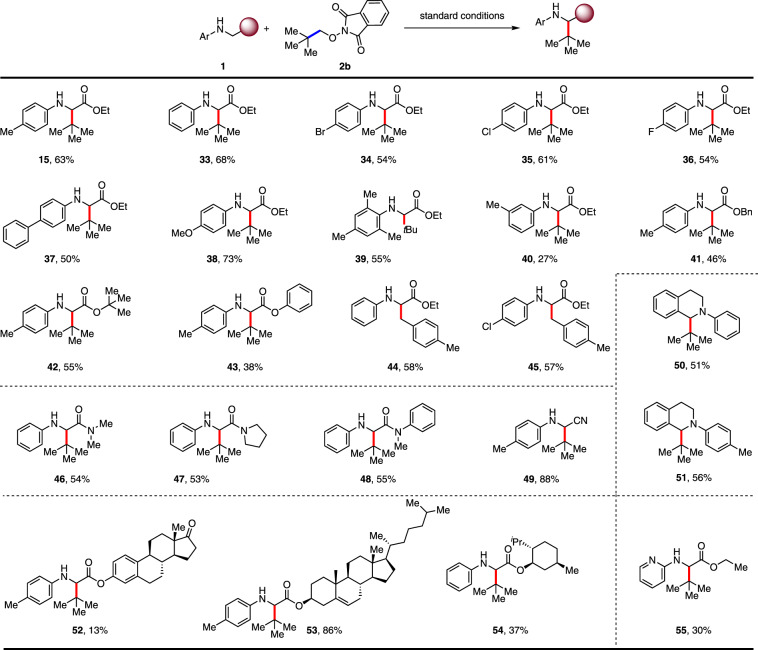
Scope of *N*-aryl protected derivatives. Conditions: All reactions were carried out with 4CzIPN (5 mol%), **1** (0.2 mmol), **2b** (0.1 mmol), and DMSO (0.03 M) irradiated under 15 W Blue LEDs at 60 °C.

Further expansion of the substrate scope was focused on glycine derivatives (Fig. 4). In order to prove the steric tolerance of this transformation, **2b** was employed as the substrate. The ethyl esters of glycine bearing both electron-donating and electron-withdrawing groups on the aromatic ring were well tolerated (**15**, **33**–**38**), resulting in 50–73% yields. In addition, the efficiency was maintained when *N*-mesityl glycine ester was used for the reaction (**39**, 55%). Then switching the ethyl ester to benzyl, *tert*-butyl, and phenyl esters, the desired products were obtained in moderate yields (**41**–**43**, 38–55%). Glycinamides and glycinitrle worked smoothly to deliver the desired products in 53–88% yields (**46**–**49**). *N*-aryl tetrahydroisoquinolines were employed for this reaction and gave coupling products in 51–56% yields (**50** and **51**). Substrates derived from natural products such as estrone, cholesterol, and menthol were successfully tolerated under standard conditions, reflecting the mildness of the reaction conditions (**52**–**54**). Finally, we found *N*-pyridine derived glycine ester worked for this reaction (**55**, 30%), giving promise for further exploring its application in agrochemical discovery.

**Fig. 5:**
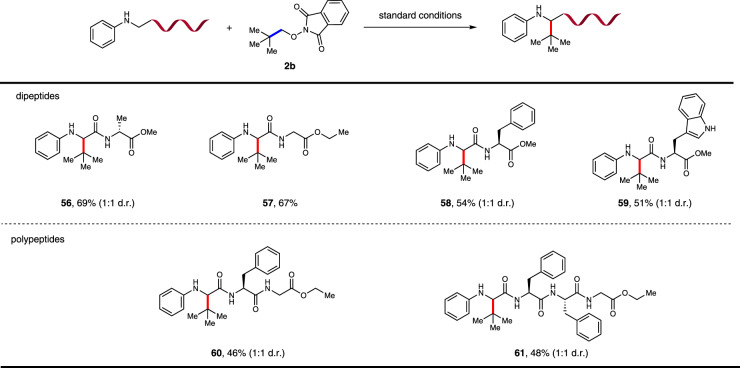
Scope of peptides. Conditions: All reactions were carried out with 4CzIPN (5 mol%), peptide (0.2 mmol), **2b** (0.1 mmol), and DMSO (0.03 M) irradiated under 15 W Blue LEDs at 60 °C.

Further utility of this transformation was demonstrated by conjugation of the peptide with **2b** (Fig. 5). The *N*-phenyl modified dipeptides, synthesized from a combination of glycine with alanine, glycine, phenylalanine, and tryptophan, successfully delivered the conjugated products in 51–68% yields (**56**–**59**). More complex tripeptide and tetrapeptide also demonstrated the efficiency of this transformation, furnishing the products in synthetically useful yields (**60** and **61**, 46% and 48%).

### Examining functional group compatibility

**Fig. 6:**
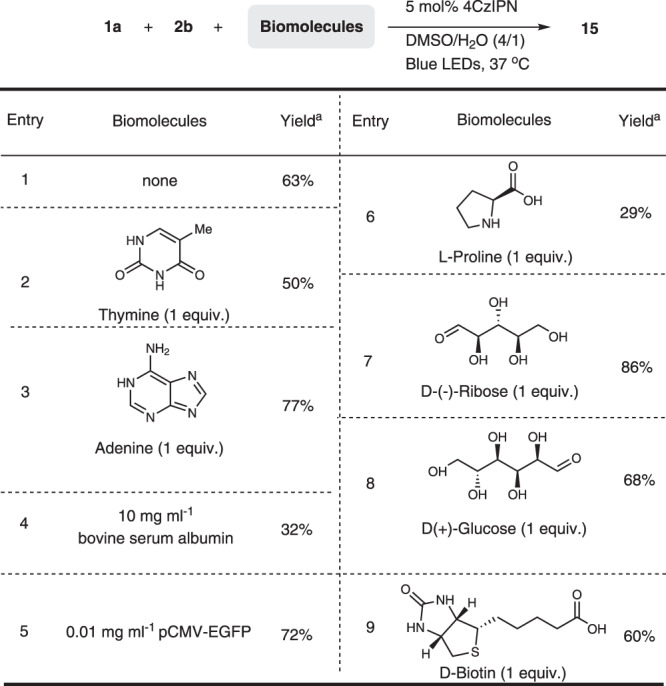
Examining functional group compatibility. Conditions: All reactions were carried out with 4CzIPN (5 mol%), **1a** (0.2 mmol), **2b** (0.1 mmol), biomolecule, and DMSO (0.03 M) irradiated under 15 W Blue LEDs at 60 °C. ^a^ Yields were determined by HPLC analysis.

After the demonstration of the broad substrate scope, especially coupling with the peptides, stimulated us to explore the compatibility with biomolecules, which could prove the possibility of this reaction for biological application. To this end, a mixture solvent system (H_2_O: DMSO = 1: 4) and biological temperature (37 °C) was employed and a broad of biomolecules were selected, including nucleobase, amino acid, saccharide, biotin, protein, and DNA. As shown in Fig. 6, all reactions worked and gave desired product in the presence of these biomolecules (entries 1–9).

### In vitro antifungal activities

Phytopathogenic fungi have imposed a major restriction on the stability and safety of agricultural production^46^. Discovering novel antifungal agrochemicals from natural sources is highly appealing due to their low toxicity and easy degradation. Terpenes such as sclareolide, camphor, limonene, pinene, etc., have shown diverse biological activities, including anti-bacteria, anti-fungus, and anti-tumor. By employing our decomplexity strategy, we transformed a few phytochemicals into glycine derivatives **27**–**29** with unique scaffolds and subjected these compounds against antifungal activity tests toward six crop pathogenic fungi with boscalid as a positive control. As shown in Table 2, (–)-*β-*pinene-derived compound **28** and (+)-camphor-derived compound **29** showed more potent activities than their parent compounds, respectively. However, compound **27** derived from sclareolide showed worse activities than sclareolide. Compound **29** presented fair to potent antifungal activities against *B. cinerea, S. sclerotiorum, C. orbiculare*, and *T. cucumeris*. The anti-*S. sclerotiorum* effect of compound **29** was up to 75%, which set a good starting point for further progress. In contrast, no inhibition was observed with camphor toward *S. sclerotiorum*. In addition, compound **29** showed better inhibition toward *C. orbiculare* and *C. paradoxa* than the positive control. The current preliminary anti-fungus activities indicate a promising prospect for further scaffold optimization based on terpenoid and glycine derivatives.

**Table 2:** In vitro antifungal activities of the target compounds at 50.0 mg/L

Compd	Inhibition rate (%)^a^					
	*B. cinerea*	*S. sclerotiorum*	*C. orbiculare*	*T. cucumeris*	*Alternariamali*	*C. paradoxa*
boscalid	90.3 ± 0.9	85.1 ± 0.8	21.5 ± 0.8	86.6 ± 1.5	82.5 ± 1.0	26.7 ± 3.8
(+)-sclareolide	16.7 ± 1.5	92.0 ± 1.9	23.9 ± 1.7	49.7 ± 2.6	40.4 ± 1.4	27.7 ± 4.5
**27**	2.7 ± 1.5	42.6 ± 3.3	5.4 ± 5.5	7.8 ± 2.4	7.7 ± 0.5	4.9 ± 1.3
(-)-*β*-pinene	―	52.7 ± 2.2	9.9 ± 0.4	5.9 ± 2.5	2.4 ± 1.3	9.1 ± 3.3
**28**	21.8 ± 3.9	40.1 ± 8.4	21.2 ± 1.0	44.4 ± 0.4	24.9 ± 2.9	26.2 ± 1.8
(+)-camphor	―	―	3.8 ± 1.7	18.7 ± 3.9	13.5 ± 1.3	27.9 ± 1.5
**29**	36.7 ± 1.9	75.0 ± 4.6	33.1 ± 1.7	45.7 ± 3.2	29.6 ± 1.3	29.9 ± 6.7


^a^Values are the mean ± standard deviation of three replicates.

### Mechanistic studies

**Fig. 7:**
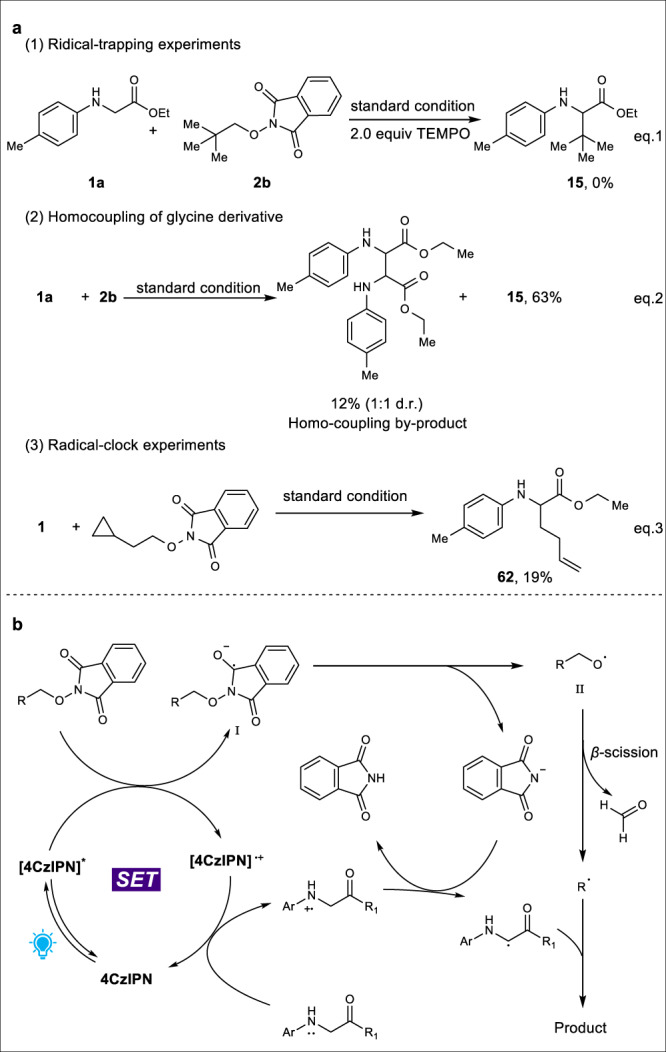
Mechanistic studies. **a** Control experiments; **b** Proposed mechanism.

To gain mechanistic insights into this coupling reaction, radical inhibitor 2,2,6,6-tetramethyl-1-peperidyloxy (TEMPO) was added to the model reaction system and it was found that it completely inhibited the coupling reaction (Fig. 7a eq.1). Moreover, during the substrate scope exploration, we could isolate the homo-coupling product in 12% yield (Fig. 7a eq.2). Furthermore, the radical clock was devised and ring-opened product **62** was isolated in 19% yield (Fig. 7a eq.3). Combining all the evidences from above reactions, a radical–radical coupling mechanism was proposed. As shown in Fig. 7b, the photoexcited 4CzlPN is able to undergo single-electron transfer (SET) reduction with *N*-alkoxyphthalimide substrates to form radical anion intermediate **I**, which could trigger the oxygen radical formation via phthalimide fragmentation. The resulting oxygen radical **II** quickly undergoes *β*-fragmentation to generate carbon-centered radical and radical stability explains the efficiency of *tert*-butyl radical addition. On the other way, glycine derivatives could quench the oxidized 4CzlPN to regenerate the photocatalyst. Meanwhile, the resulting glycine radical could combine with carbon radical from *N*-alkoxyphthalimides to furnish the desired coupling products.

In conclusion, we have developed a convenient method based on a single-electron transfer process to construct C–C bond, which provides an alternative to the classical two-electron approach, bypassing the need to utilize harsh conditions and enabling the use of bench stable and easily prepared starting materials. The mild reaction conditions and broad functional group compatibility extend the application with peptides, making them preeminent and efficient species for potential biological and chemical applications. Deconstructing complex natural products offers a set of bioactive compounds and lays the foundation for further agrochemical development.

## Methods

### General procedure for the synthesis of 3–61

The *N*-alkoxyphthalimides (0.1 mmol), the esters, or amides of *N*-aryl-substituted glycine, or *N*-aryl tetrahydroisoquinoline, or peptides (0.2 mmol, 2.0 equiv.), 4CzIPN (5 mol%, 0.005 mmol, 4.0 mg), and DMSO (3 mL, 0.03 M) were added sequentially to a 4 mL clear-colored glass vial equipped with a magnetic stir bar. After bubbled with nitrogen gas for 5 min to remove oxygen, the vial was sealed and exposed to blue LEDs at 60 °C. The reaction mixture was monitored by TLC until the starting material *N*-alkoxyphthalimides were consumed. Then, the reaction was quenched with water (2 mL), extracted with ethyl acetate, washed with brine, dried over anhydrous Na_2_SO_4_, concentrated in vacuo, and purified by column chromatography to yield the products **3–61**.

### In vitro antifungal activities

Each target compound was dissolved in DMSO to prepare the stock solution (10.0 g/L). The stock solution was added to the PDA medium, and the concentration of target compounds in the medium was 50.0 mg/L. Pure DMSO without the target compounds was utilized as the blank control, and boscalid was coassayed as the reference compound. Fresh dishes with a diameter of 5 mm were taken from the edge of the PDA-cultured fungi colonies and inoculated on the above three PDA media. Each treatment was tested for three replicates, and the antifungal effect was averaged. The relative inhibitory rate I (%) of all the tested compounds was calculated through the equation: I (%) = [(C − T)/(C − 5)] × 100. In this equation, I is the inhibitory rate and C and T are the colony diameter of the blank control (mm) and treatment (mm), respectively.

### Reporting summary

Further information on research design is available in the Nature Portfolio Reporting Summary linked to this article.

## Supplementary information

Supplementary Information

Reporting Summary

## Data Availability

The authors declare that all data supporting the findings of this study are available within the article and Supplementary Information files.
