# Supplementary material for: Photoinduced β-fragmentation of aliphatic alcohol derivatives for forging C–C bonds
Source: Nat Commun. 2022 Dec 2;13:7450. doi: 10.1038/s41467-022-35249-7 (PMC9718844; doi:10.1038/s41467-022-35249-7)
Supplement: Supplementary file 1 — Supplementary Information [file 41467_2022_35249_MOESM1_ESM.pdf]

Supplementary Information

Photoinduced *β*-Fragmentation of aliphatic alcohol derivatives for forging C–C bonds

Yiman Gao, Jie Liu, Cong Wei, Kui Zhang, Liangliang Song, Lingchao Cai*

Table of Contents

1. Supplementary Methods 3

1.1 General Information 3

2. Supplementary Discussion 4

2.1 Substrate Preparation 4

2.2 Experimental Procedures 16

2.2.1 General Procedure for the Synthesis of the products **3–62** 16

2.2.2 Characterization Data for the product **3–62**. 17

3. Supplementary Notes 32

3.1 Reaction Profile General 32

3.2 Examining Functional Group Compatibility 33

3.3 In Vitro Antifungal Activities. 34

4. Supplementary Figures 38

4.1 NMR Spectra 38

5. Supplementary Reference 109

1. Supplementary Methods

1.1 General Information

All reactions were performed in dry solvents under an N_2 atmosphere and anhydrous conditions. DCM, THF, toluene, diethyl ether, and MeCN to be used in anhydrous reaction mixtures were dried by passage through activated alumina columns immediately prior to use. All other reagents were used as received from commercial sources. Reactions were monitored through thin layer chromatography (TLC) on 0.25-mm silica gel plates and visualized under UV light. Flash column chromatography (FCC) was performed using Flash silica gel (60-Å pore size, 40–63 μm). NMR spectra were recorded on Bruker Avance-400 or -600 instrument, calibrated to $CD(H)Cl_3$ as the internal reference (7.26 and 77.0 ppm for ^{1}H and ^{13}C NMR spectra, respectively). ^{1}H NMR spectral data were reported in terms of chemical shift (δ, ppm), multiplicity, coupling constant (Hz), and integration. ^{13}C NMR and ^{19}F NMR spectral data were reported in terms of chemical shift (δ, ppm). The following abbreviations indicated the multiplicities: s, singlet; d, doublet; t, triplet; q, quartet; m, multiplet; br, broad. High-resolution mass spectra were recorded using a SCIEX X500R LC-Q-TOF, ESI ion Source. The 15 W blue LED lamps were directly got from the supermarket. The crop pathogenic fungi, such as *Botrytis cinerea (B. cinerea), Sclerotinia sclerotiorum (S. sclerotiorum), Colletotrichum orbiculare (C. orbiculare), Thanatephorus cucumeris(Frank)Donk (T. cucumeris), Alternariamali, and Ceratocystis paradoxa (C. paradoxa)*, were purchased from the Agricultural Culture Collection of China, which were preserved in agar slants at 4 °C.

2. Supplementary Discussion

2.1 Substrate Preparation

Starting Materials

N-aryl protected derivatives

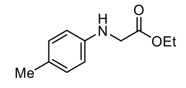

1a

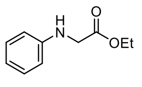

1b

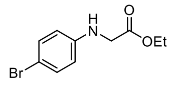

1c

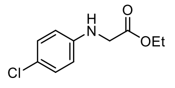

1d

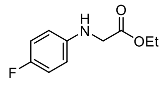

1e

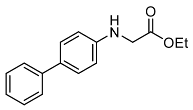

1f

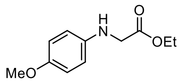

1g

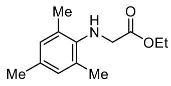

1h

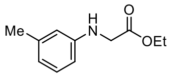

1i

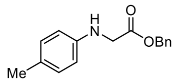

1j

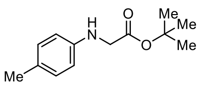

1k

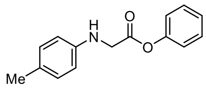

1l

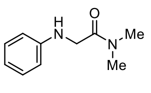

1m

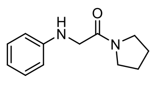

1n

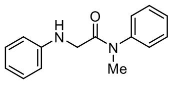

1o

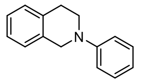

1p

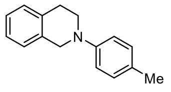

1q

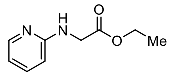

1r

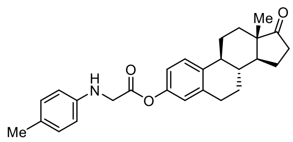

1s

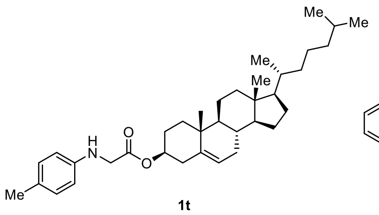

1t

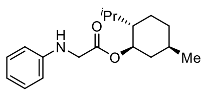

1u

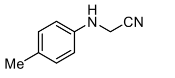

1v

Peptides

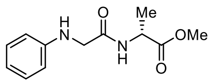

1aa

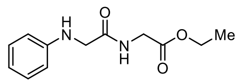

1ab

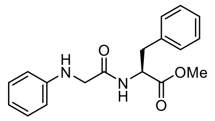

1ac

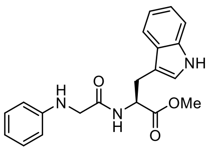

1ad

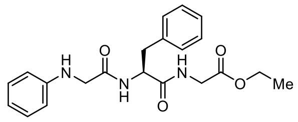

1ae

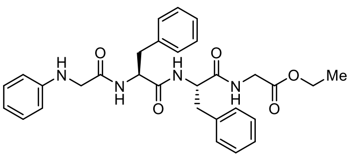

1af

N-alkoxyphthalimides

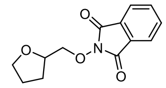

2a

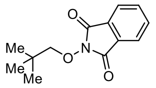

2b

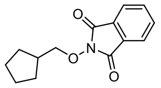

2c

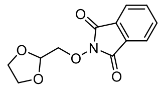

2d

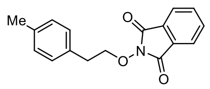

2e

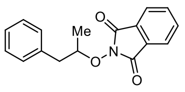

2f

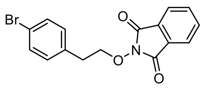

2g

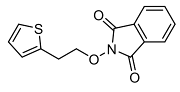

2h

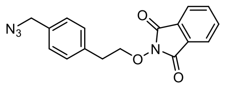

2i

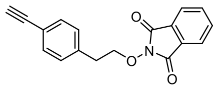

2j

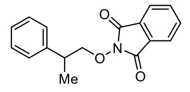

2k

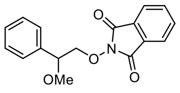

2l

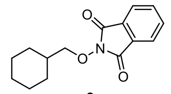

2m

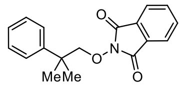

2n

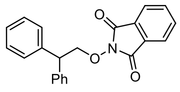

2o

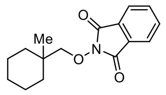

2p

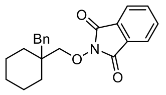

2q

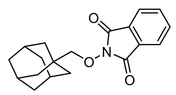

2r

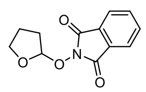

2s

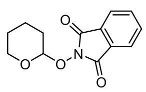

2t

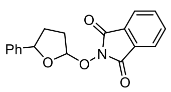

2u

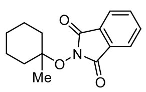

2v

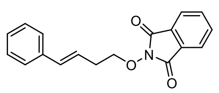

2w

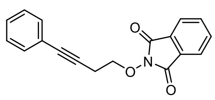

2x

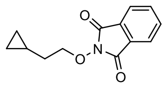

2y

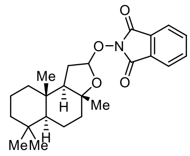

2aa
from Sclareolide

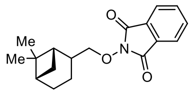

2ab
from (–)-β-pinene

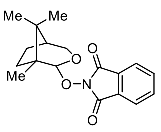

2ac
from D-camphor

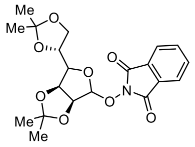

2ad
from D(+)-Glucose

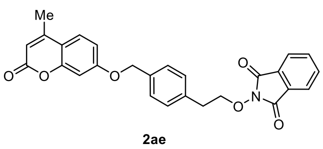

2ae
from 4-Methylumbelliferone

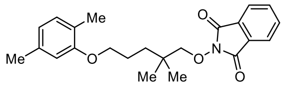

2af
from Gemfibrozil

2.1.1 Substrates 1 Preparation

Esters, amides of *N*-aryl-substituted glycine,[1] *N*-aryl tetrahydroisoquinoline,[2] *N*-pyridyl-substituted glycine,[3] *N*-(*p*-tolyl)-glycinitrile[30] were all prepared according to the previous reports.

Synthesis of *N*-aryl protected derivatives precursors

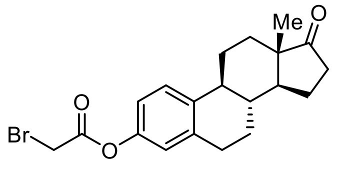

(8*R*,9*S*,13*S*,14*S*)-13-methyl-17-oxo-7,8,9,11,12,13,14,15,16,17-decahydro-6*H*-cyclopenta[*a*]phenanthren-3-yl 2-bromoacetate (1s-1)

The titled compound was synthesized following a literature procedure from estrone.[4] Spectra are consistent with reported literature values.

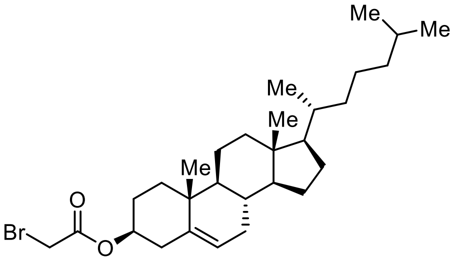

(3*S*,8*S*,9*S*,10*R*,13*R*,14*S*,17*R*)-10,13-dimethyl-17-((*R*)-6-methylheptan-2-yl)-2,3,4,7,8,9,10,11,12,13,14,15,16,17-tetradecahydro-1*H*-cyclopenta[a]phenanthren-3-yl 2-bromoacetate (1t-1)

The titled compound was synthesized following a literature procedure from cholesterol.[5] Spectra are consistent with reported literature values.

Synthesis of *N*-aryl protected derivatives

General method

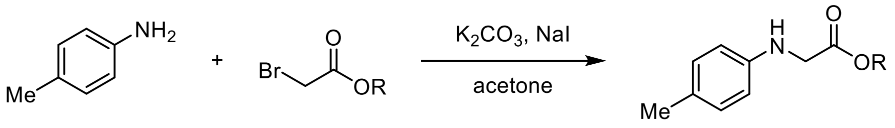

Following the literature procedure[6] with slight modifications. The mixture of *p*-toluidine (1.1 g, 10 mmol), potassium carbonate (1.7 g, 12 mmol) and sodium iodide (1.8 g, 12 mmol) were treated with the corresponding bromoacetate-ester (11 mmol), in dry acetone as the solvent, at 60 °C for 12 h. To the resulting mixture, was cooled to room temperature, and filtered through a celite pad, then concentrated in vacuo, and subjected to flash chromatography to afford the *N*-aryl protected derivatives.

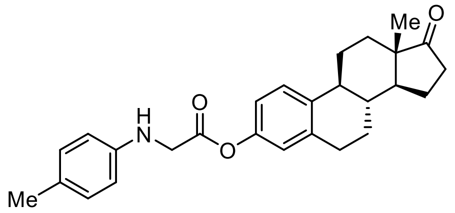

(8*R*,9*S*,13*S*,14*S*)-13-methyl-17-oxo-7,8,9,11,12,13,14,15,16,17-decahydro-6*H*-cyclopenta[*a*]phenanthren-3-yl *p*-tolylglycinate (1s)

Following the general method, the reaction of **1s-1** (390 mg, 1 mmol) afforded *N*-aryl protected derivatives **1s** as a yellow oil (0.17 g, 40% yield). ^{1}H NMR (400 MHz, $CDCl_3$) δ 7.31 (d, J = 8.4 Hz, 1H), 7.18 (d, J = 8.6 Hz, 1H), 7.06 (d, J = 7.5 Hz, 2H), 6.89 (d, J = 8.4 Hz, 1H), 6.84 (s, 1H), 6.64 (q, J = 8.5 Hz, 2H), 4.17 (s, 2H), 2.97–2.86 (m, 2H), 2.28 (s, 3H), 2.23–2.16 (m, 1H), 2.09–1.98 (m, 3H), 1.65–1.51 (m, 7H), 0.93 (s, 3H); ^{13}C NMR (151 MHz, $CDCl_3$) δ 221.0, 170.3, 153.7, 138.0, 131.9, 129.9, 126.5, 121.3, 118.5, 115.3, 113.4, 112.9, 50.5, 48.0, 46.6, 44.0, 38.4, 38.0, 35.9, 31.6, 26.5, 25.9, 21.6, 20.4, 13.9. HRMS (ESI, m/z) calcd for $C_{27}H_{32}NO_3$ $(M+H)^+$: 418.2377, found: 418.2372.

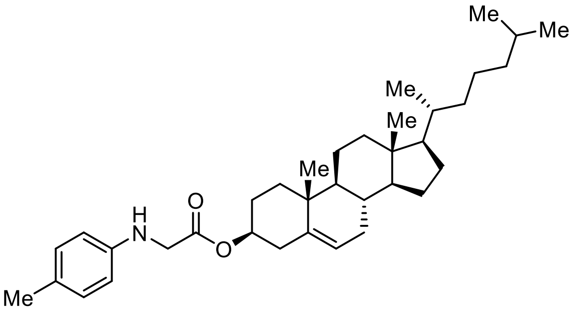

(3*S*,8*S*,9*S*,10*R*,13*R*,14*S*,17*R*)-10,13-dimethyl-17-((*R*)-6-methylheptan-2-yl)-2,3,4,7,8,9,10,11,12,13,14,15,16,17-tetradecahydro-1*H*-cyclopenta[a]phenanthren-3-yl *p*-tolylglycinate (1t)

Following the general method, the reaction of **1t-1** (506 mg, 1 mmol) afforded *N*-aryl protected derivatives **1t** as a white solid (0.35 g, 70% yield). m.p. 159–161 °C; ^{1}H NMR (400 MHz, $CDCl_3$) δ 7.03 (d, J = 7.6 Hz, 2H), 6.56 (d, J = 7.6 Hz, 2H), 5.40 (s, 1H), 4.74 (d, J = 8.4 Hz, 1H), 4.17 (s, 1H), 3.88 (d, J = 4.2 Hz, 2H), 2.37 (d, J = 7.8 Hz, 2H), 2.27 (s, 3H), 2.07–1.95 (m, 2H), 1.94–1.80 (m, 3H), 1.69–1.32 (m, 7H), 1.24–0.99 (m, 17H), 0.94 (d, J = 6.2 Hz, 3H), 0.89 (d, J = 6.4 Hz, 6H), 0.70 (s, 3H); ^{13}C NMR (101 MHz, $CDCl_3$) δ 170.7, 144.9, 139.4, 129.8, 127.4, 123.0, 113.2, 75.1, 56.7, 56.1, 50.0, 46.5, 42.3, 39.7, 39.5, 38.1, 36.9, 36.6, 36.2, 35.8, 31.9, 31.8, 28.2, 28.0, 27.7, 24.3, 23.8, 22.8, 22.6, 21.0, 20.4, 19.3, 18.7, 11.9. HRMS (ESI, m/z) calcd for $C_{36}H_{56}NO_2$ $(M+H)^+$: 534.4306, found: 534.4298.

Synthesis of Peptides

Dipeptides and polypeptides were all prepared according to previous reports[7] with slight modifications.

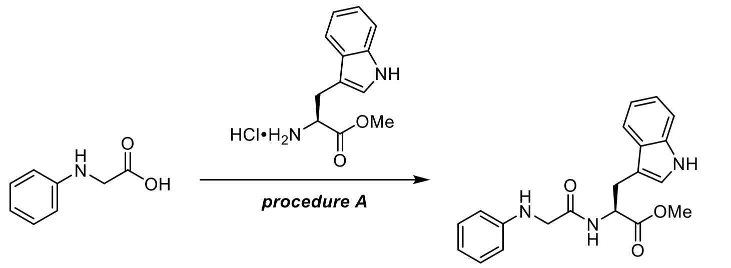

1ad

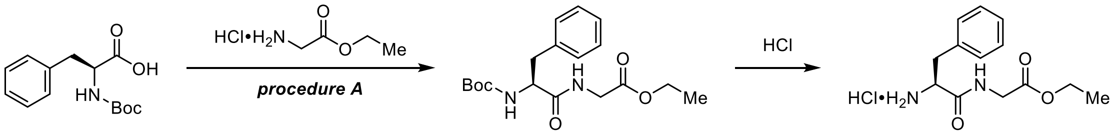

1ae-1 1ae-2

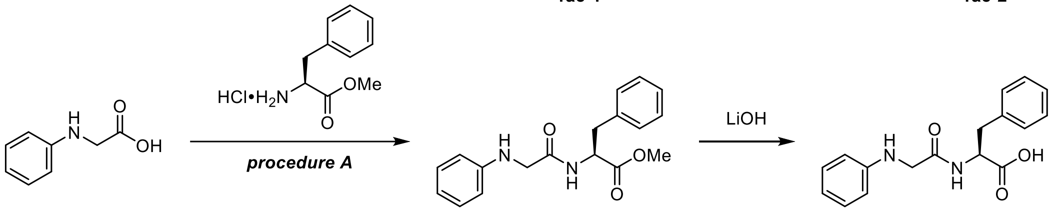

1af-1 1af-2

General Procedure A: methyl phenylglycyl-*L*-tryptophanate (1ad)

To a 100 mL round-bottom flask, *N*-phenyl glycine (3.5 mmol, 530 mg) and methyl *L*-tryptophanate hydrochloride (3.5 mmol, 891 g) were dissolved in 30 mL DCM. At 0 °C, Et_3N (7 mmol, 0.97 mL) was added and stirred for 5 min. HBTU (7 mmol, 2.65 g) was added. The reaction mixture was warmed to room temperature and stirred overnight. Subsequently, water was added to the round-bottom flask. The resulting mixture was extracted with DCM (20 mL x 3), and the combined organic layers were dried over anhydrous Na_2SO_4 and concentrated in vacuo. The residue was purified by column chromatography and the **1ad** was obtained as a pale-yellow solid. m.p. 164–165 °C; ^{1}H NMR (400 MHz, $CDCl_3$) δ 8.03 (s, 1H), 7.45 (d, J = 7.9 Hz, 1H), 7.31 (d, J = 8.1 Hz, 1H), 7.19 (q, J = 7.0 Hz, 4H), 7.07 (t, J = 7.4 Hz, 1H), 6.84 (t, J = 7.3 Hz, 1H), 6.60 (s, 1H), 6.54 (d, J = 7.7 Hz, 2H), 4.98 (m, J = 4.6 Hz, 1H), 3.74 (s, 2H), 3.69 (s, 3H), 3.29 (dq, J = 5.2, 16.2 Hz, 2H); ^{13}C NMR (101 MHz, $CDCl_3$) δ 172.1, 170.5, 146.9, 136.1, 129.4, 127.4, 123.0, 122.2, 119.7, 119.0, 118.4, 113.3, 111.3, 109.5, 52.4, 52.2, 48.6, 27.5. HRMS (ESI, m/z) calcd for $C_{20}H_{22}N_3O_3$ $(M+H)^+$: 352.1656, found: 352.1653.

ethyl *L*-phenylalanylglycinate hydrochloride (1ae-2)

Following the general procedure A, the reaction of glycine ethyl ester hydrochloride and *N*-(tert-butoxycarbonyl)-*L*-phenylalanine afforded the dipeptide **1ae-1** as a white solid. To a stirred solution of **1ae-1** (1 mmol, 350 mg) in dichloromethane (20 mL) was added 1N HCl (2 mL) and the resultant mixture was stirred for 60 min at room temperature. After completion of the reaction monitored by TLC, the reaction were dried over with Na_2SO_4, filtered and concentrated under reduced pressure The desired product **1ae-2** was afforded as yellow solid, which without further purification and used in next step directly.

phenylglycyl-*L*-tryptophan (1af-2)

Following the general procedure A, the reaction of *N*-phenyl glycine and Methyl *L*-phenylalaninate hydrochloride afforded the dipeptide **1af-1** as a white solid. To a stirred solution of **1af-1** (1 mmol, 312 mg) in methanol (20 mL) was added 1N LiOH (2 mL) and the resultant mixture was stirred for 8 h at room temperature. After completion of the reaction monitored by TLC, the reaction was concentrated under reduced pressure to remove methanol, and then extracted with EtOAc (30 mL). The aqueous layers were collected and the pH was adjusted to 2-3 by 2N HCl. Following extraction with EtOAc (20 mL x 2), the combined organic layers were dried over with Na_2SO_4, filtered and concentrated under reduced pressure. The desired product **1af-2** was afforded as yellow solid, which without further purification and used in next step directly.

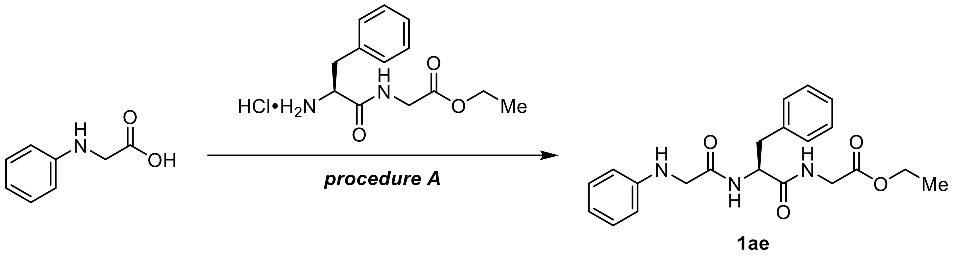

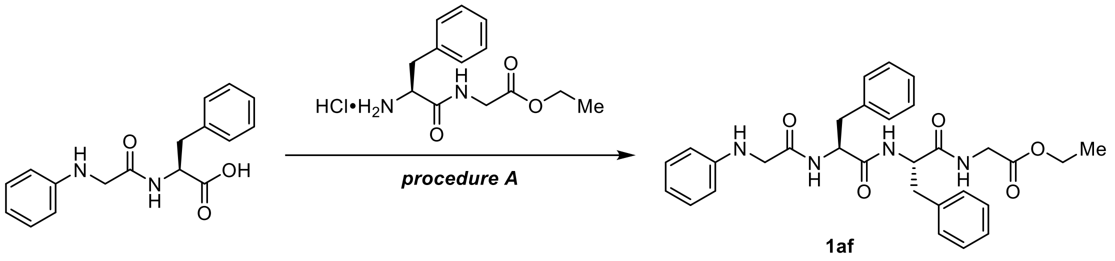

ethyl phenylglycyl-*L*-phenylalanylglycinate (1ae)

Following the general procedure A, the reaction of **1ae-2** afforded polypeptide **1ae** as a white solid. m.p. 135-136 °C; ^{1}H NMR (400 MHz, $CDCl_3$) δ 7.29 (d, J = 7.2 Hz, 1H), 7.20 (s, 5H), 7.08 (s, 2H), 6.83 (t, J = 7.2 Hz, 1H), 6.75 (s, 1H), 6.54 (d, J = 7.8 Hz, 2H), 4.82 (q, J = 7.2 Hz, 1H), 4.19 (q, J = 7.1 Hz, 2H), 3.94 (m, J = 9.7 Hz, 2H), 3.76 (q, J = 17.6 Hz, 2H), 3.07 (t, J = 6.4 Hz, 2H), 1.28 (t, J = 7.0 Hz, 3H); ^{13}C NMR (101 MHz, $CDCl_3$) δ 171.2, 171.1, 169.5, 146.9, 136.2, 129.5, 129.2, 128.6, 127.0, 119.2, 113.3, 61.6, 53.8, 48.6, 41.3, 37.7, 14.1. HRMS (ESI, m/z) calcd for $C_{21}H_{26}N_3O_4$ $(M+H)^+$: 384.1918, found: 384.1909.

ethyl phenylglycyl-*L*-phenylalanyl-*L*-phenylalanylglycinate (1af)

Following the general procedure A, the reaction of **1ae-2** and **1af-2** afforded polypeptide **1af** as a white solid. m.p. 155–156 °C; ^{1}H NMR (600 MHz, $CDCl_3$) δ 7.25–7.19 (m, 5H), 7.14 (d, J = 6.0 Hz, 3H), 7.07 (d, J = 7.2 Hz, 2H), 7.05 (d, J = 8.2 Hz, 1H), 6.97 (d, J = 5.6 Hz, 2H), 6.88 (s, 1H), 6.83 (t, J = 7.3 Hz, 1H), 6.54 (d, J = 8.0 Hz, 2H), 4.82 (q, J = 7.4 Hz, 1H), 4.77 (q, J = 7.1 Hz, 1H), 4.18 (q, J = 7.1 Hz, 2H), 4.01 (m, J = 7.9 Hz, 1H), 3.85 (m, J = 7.7 Hz, 1H), 3.70 (q, J = 16.2 Hz, 2H), 3.09 (m, J = 6.7 Hz, 1H), 2.96 (m, J = 4.3 Hz, 2H), 2.88 (m, J = 7.2 Hz, 1H), 1.28 (t, J = 7.1 Hz, 3H); ^{13}C NMR (101 MHz, $CDCl_3$) δ 171.3, 171.1, 170.9, 169.6, 147.0, 136.6, 136.0, 129.5, 129.4, 129.3, 128.6, 128.5, 127.0, 126.8, 119.0, 113.1, 61.5, 54.1, 54.0, 48.2,

41.3, 38.0, 37.8, 14.2. HRMS (ESI, m/z) calcd for $C_{30}H_{35}N_4O_5$ $(M+H)^+$: 531.2602, found: 531.2605.

2.1.2 Substrates 2 Preparation

The *N*-alkoxyphthalimides **2a–d**, **2f**, **2k**, **2m–n**,[8] **2l**,[9] **2w**,[10] **2x**,[11] **2s–u**,[12] **2v**,[13] **2ad**[14] were prepared using reported methods.

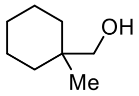

(1-methylcyclohexyl)methanol (1p-1)

The titled compound was synthesized following a literature procedure from 1-methylcyclohexanecarboxaldehyde.[15] Spectra are consistent with reported literature values.

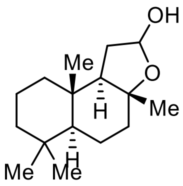

(3a*R*,5a*S*,9a*S*,9b*R*)-3a,6,6,9a-tetramethyldodecahydronaphtho[2,1-*b*]furan-2-ol (2aa-1)

The titled compound was synthesized following a literature procedure from sclareolide.[16] Spectra are consistent with reported literature values.

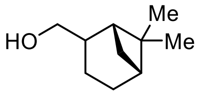

((1*R*,5*R*)-6,6-dimethylbicyclo[3.1.1]heptan-2-yl)methanol (2ab-1)

The titled compound was synthesized following a literature procedure from (1*S*)-(1)-beta-pinene.[17] Spectra are consistent with reported literature values.

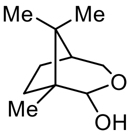

1,8,8-trimethyl-3-oxabicyclo[3.2.1]octan-2-ol (2ac-1)

The titled compound was synthesized following a literature procedure from *D*-camphor.[18] Spectra are consistent with reported literature values.

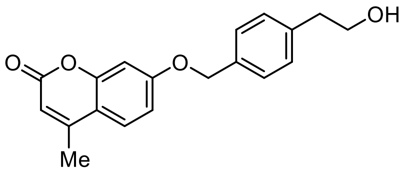

7-((4-(2-hydroxyethyl)benzyl)oxy)-4-methyl-2*H*-chromen-2-one (2ae-1)

The titled compound was synthesized following a literature procedure from 7-hydroxy-4-methyl-chromen-2-one.[19] Spectra are consistent with reported literature values.

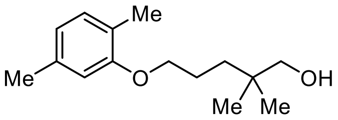

5-(2,5-dimethylphenoxy)-2,2-dimethylpentan-1-ol (2af-1)

The titled compound was synthesized following a literature procedure from gemfibrozil.[20] Spectra are consistent with reported literature values.

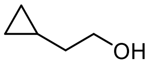

2-cyclopropylethan-1-ol (2y-1)

The titled compound was synthesized following a literature procedure from cyclopropylacetic acid.[21] Spectra are consistent with reported literature values.

Synthesis of *N*-alkoxyphthalimides

Method A

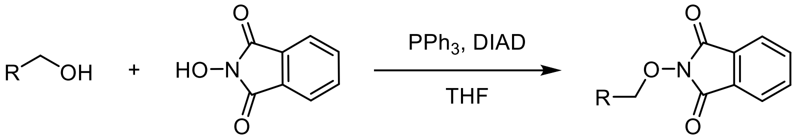

Following the literature procedure[22] with slight modifications.To a solution of the alcohol (10.0 mmol), PPh_3 (3.15 g, 12.0 mmol), and *N*-hydroxyphthalimide (1.96 g, 12.0 mmol) in THF (30 mL) was added diisopropyl azodicarboxylate (2.4 mL, 12.0 mmol) over 10 min at room temperature. The resulting mixture was stirred for 3-24 h, taken up in EtOAc (20 mL), and washed with saturated $NaHCO_3$ (3 x 20 mL) and brine (2 x 30 mL). The organic layers were dried over anhydrous Na_2SO_4, concentrated in vacuo, and subjected to flash chromatography to afford the *N*-alkoxyphthalimides.

Method B

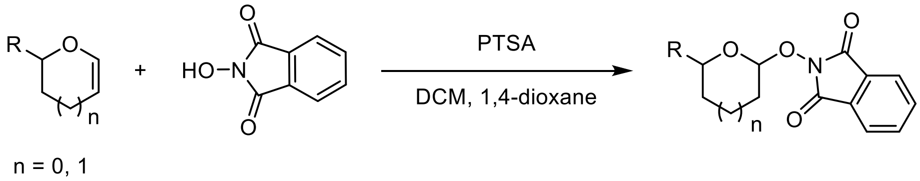

Following the literature procedure[23] with slight modifications. To a solution of the *N*-hydroxyphthalimide (1.5 g, 9.2 mmol) in DCM (30 mL) were add 1,4-dioxane (20 mL), PTSA (31.6 mg, 0.02 mmol) and dihydropyran or dihydrofuran (13.6 mmol) at room temperature. The reaction mixture was stirred for 3 h. To the resulting mixture, DCM (10 mL) and saturated Na_2CO_3 solution in H_2O (50 mL) was added. The aqueous layer was extracted with DCM (20 mL x 3), the combined organic layers were dried over anhydrous Na_2SO_4, concentrated in vacuo, and subjected to flash chromatography to afford the *N*-alkoxyphthalimides.

Method C

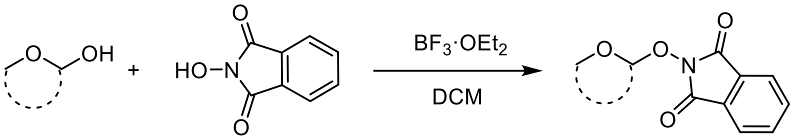

Following the literature procedure[24] with slight modifications. To a solution of hemiacetal (10 mmol) and *N*-hydroxyphthalimide (3.26 g, 20 mmol) in 70 mL wet DCM, then $BF_3 \cdot OEt_2$ (6.8 mL, 25 mmol) was added dropwise slowly at 0 ºC. The reaction mixture was stirred for 5 h at room temperature. To the resulting mixture, DCM (10 mL) and saturated Na_2CO_3 solution in H_2O (50 mL) was added. The aqueous layer was extracted with DCM (20 mL x 3), the combined organic layers were dried over anhydrous Na_2SO_4, concentrated in vacuo, and subjected to flash chromatography to afford the *N*-alkoxyphthalimides.

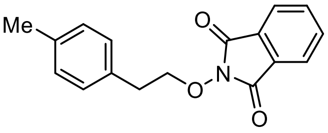

2-(4-methylphenethoxy)isoindoline-1,3-dione (2e)

Following the general method A, the reaction of 2-(p-Tolyl)ethanol (408 mg, 3 mmol) afforded *N*-alkoxyphthalimides **2e** as a white solid (0.67 g, 79% yield). m.p. 82–84 °C; ^{1}H NMR (400 MHz, $CDCl_3$) δ 7.85 (m, 2H), 7.76 (m, 2H), 7.20 (d, J = 7.4 Hz, 2H), 7.13 (d, J = 7.4 Hz, 2H), 4.43 (t, J = 7.3 Hz, 2H), 3.13 (t, J = 7.3 Hz, 2H), 2.32 (s, 3H); ^{13}C NMR (101 MHz, $CDCl_3$) δ 163.6, 136.2, 134.5, 133.6, 129.3, 128.9, 128.7, 123.5, 78.6, 34.2, 21.0. HRMS (ESI, m/z) calcd for $C_{17}H_{16}NO_3$ $(M+H)^+$: 282.1125, found: 282.1125.

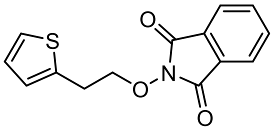

2-(2-(thiophen-2-yl)ethoxy)isoindoline-1,3-dione (2h)

Following the general method A, the reaction of 2-Thiopheneethanol (1.28 g, 10 mmol) afforded *N*-alkoxyphthalimides **2h** as a white solid (1.53 g, 56% yield). m.p. 67–69 °C; ^{1}H NMR (400 MHz, $CDCl_3$) δ 7.86 (m, 2H), 7.77 (m, 2H), 7.18 (d, J = 4.8 Hz, 1H), 6.98 (m, 2H), 4.46 (t, J = 6.9 Hz, 2H), 3.38 (t, J = 7.1 Hz, 2H); ^{13}C NMR (101 MHz, $CDCl_3$) δ 163.6, 138.7, 134.6, 128.9, 127.0, 125.8, 124.1, 123.6, 78.2, 29.0. HRMS (ESI, m/z) calcd for $C_{14}H_{12}NO_3S$ $(M+H)^+$: 274.0533, found: 274.0537.

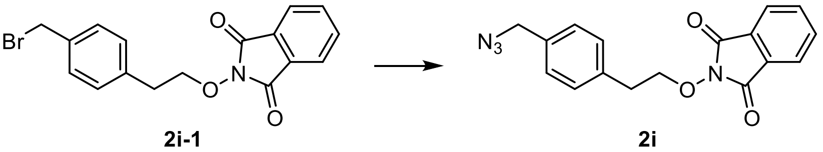

2-(4-(azidomethyl)phenethoxy)isoindoline-1,3-dione (2i)

Following the general method A, the reaction of 2-(4-(bromomethyl)phenyl)ethanol afforded *N*-alkoxyphthalimides **2i-1,** The **2i** was synthesized following a literature procedure[25] from **2i-1** as a white solid (0.84 g, 60% yield). m.p. 112–114 °C; ^{1}H NMR (400 MHz, $CDCl_3$) δ 7.85 (m, 2H), 7.77 (m, 2H), 7.36 (d, J = 7.6 Hz, 2H), 7.28 (d, J =

7.4 Hz, 2H), 4.46 (t, J = 7.1 Hz, 2H), 4.32 (s, 2H), 3.18 (t, J = 7.2 Hz, 2H); ^{13}C NMR (101 MHz, $CDCl_3$) δ 163.6, 137.1, 134.6, 133.8, 129.4, 128.9, 128.5, 123.6, 78.3, 54.5, 34.3. HRMS (ESI, m/z) calcd for $C_{17}H_{18}N_5O_3$ $(M+NH_4)^+$: 340.1404, found: 340.1406.

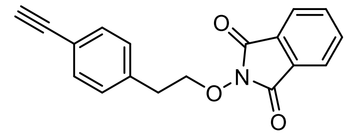

2-(4-ethynylphenethoxy)isoindoline-1,3-dione (2j)

Following the general method A, the reaction of 2-(4-ethynylphenyl)ethanol (292 mg, 2 mmol) afforded *N*-alkoxyphthalimides **2j** as a white solid (349 mg, 60% yield). m.p. 111–112 °C; ^{1}H NMR (400 MHz, $CDCl_3$) δ 7.85 (m, 1H), 7.78 (m, 1H), 7.46 (d, J = 7.6 Hz, 2H), 7.30 (d, J = 8.4 Hz, 2H), 4.45 (t, J = 7.0 Hz, 2H), 3.17 (t, J = 7.1 Hz, 2H), 3.07 (s, 1H); ^{13}C NMR (101 MHz, $CDCl_3$) δ 163.6, 137.8, 134.6, 132.3, 128.9 (2C), 123.6, 120.4, 83.5, 78.1, 77.0, 34.5. HRMS (ESI, m/z) calcd for $C_{18}H_{17}N_2O_3$ $(M+NH_4)^+$: 309.1233, found: 309.1242.

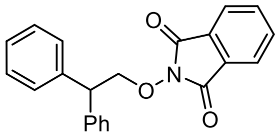

2-(2,2-diphenylethoxy)isoindoline-1,3-dione (2o)

Following the general method A, the reaction of 2,2-diphenyl ethanol (396 mg, 2 mmol) afforded *N*-alkoxyphthalimides **2o** as a white solid (500 mg, 73% yield). m.p. 155–156 °C; ^{1}H NMR (400 MHz, $CDCl_3$) δ 7.82 (m, 2H), 7.75 (m, 2H), 7.42–7.30 (m, 8H), 7.23 (t, J = 7.0 Hz, 2H), 4.79 (d, J = 7.4 Hz, 2H), 4.65 (t, J = 7.4 Hz, 1H); ^{13}C NMR (101 MHz, $CDCl_3$) δ 163.4, 140.5, 134.5, 128.8, 128.7, 128.3, 126.9, 123.5, 79.9, 49.8. HRMS (ESI, m/z) calcd for $C_{18}H_{17}N_2O_3$ $(M+NH_4)^+$: 361.1546, found: 361.1544.

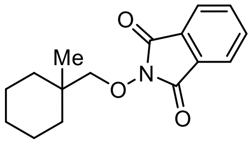

2-((1-methylcyclohexyl)methoxy)isoindoline-1,3-dione (2p)

Following the general method A, the reaction of **2p-1** (256 mg, 2 mmol) afforded *N*-alkoxyphthalimides **2p** as a colorless oil (0.29 g, 51% yield). ^{1}H NMR (400 MHz, $CDCl_3$) δ 7.81 (m, 2H), 7.73 (m, 2H), 3.94 (s, 2H), 1.61–1.31 (m, 10H), 1.12 (s, 3H); ^{13}C NMR (101 MHz, $CDCl_3$) δ 163.4, 134.3, 129.0, 123.3, 86.5, 34.5, 34.3, 26.2, 22.9, 21.6. HRMS (ESI, m/z) calcd for $C_{16}H_{22}NO_4$ $(M+H_2O+H)^+$: 291.1544, found: 291.1541.

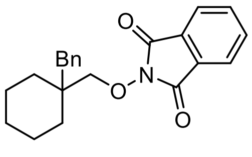

2-((1-benzylcyclohexyl)methoxy)isoindoline-1,3-dione (2q)

Following the general method A, the reaction of (1-benzylcyclohexyl)methanol (817 mg, 4 mmol) afforded *N*-alkoxyphthalimides **2q** as a white solid (0.84 g, 60% yield). m.p. 132–133 °C; ^{1}H NMR (400 MHz, $CDCl_3$) δ 7.86 (m, 1H), 7.77 (m, 1H), 7.47 (d,

J = 7.4 Hz, 2H), 7.36 (t, J = 7.3 Hz, 2H), 7.26 (q, J = 6.8 Hz, 1H), 3.99 (s, 2H), 2.85 (s, 2H), 1.67–1.37 (m, 10H); ^{13}C NMR (101 MHz, $CDCl_3$) δ 163.5, 137.7, 134.4, 131.1, 129.1, 127.9, 126.1, 123.4, 80.8, 42.1, 37.9, 32.6, 26.1, 21.6. HRMS (ESI, m/z) calcd for $C_{22}H_{24}NO_3$ $(M+H)^+$: 350.1751, found: 350.1749.

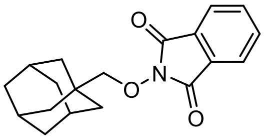

2-(((3*r*,5*r*,7*r*)-adamantan-1-yl)methoxy)isoindoline-1,3-dione (2r)

Following the general method A, the reaction of 1-Adamantanemethanol (831 mg, 5 mmol) afforded *N*-alkoxyphthalimides **2r** as a white solid (0.68 g, 44% yield). m.p. 139–140 °C; ^{1}H NMR (400 MHz, $CDCl_3$) δ 7.83 (m, 2H), 7.75 (m, 2H), 3.80 (s, 3H), 2.04 (s, 12H), 1.75 (s, 1H); ^{13}C NMR (101 MHz, $CDCl_3$) δ 163.5, 134.3, 129.1, 123.4, 88.2, 39.0, 37.0, 34.0, 28.0. HRMS (ESI, m/z) calcd for $C_{19}H_{22}NO_3$ $(M+H)^+$: 312.1594, found: 312.1599.

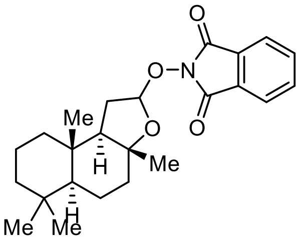

2-(((3a*R*,5a*S*,9a*S*,9b*R*)-3a,6,6,9a-tetramethyldodecahydronaphtho[2,1-*b*]furan-2-yl)oxy)isoindoline-1,3-dione (2aa)

Following the general method C, the reaction of **2aa-1** (250 mg, 1 mmol) afforded *N*-alkoxyphthalimides **2aa** as a white solid (0.14 g, 36% yield). m.p. 137–138 °C; ^{1}H NMR (400 MHz, $CDCl_3$) δ 7.82 (m, 2H), 7.74 (m, 2H), 5.79 (t, J = 5.5 Hz, 1H), 2.30 (m, 1H), 2.10–1.98 (m, 2H), 1.84–1.65 (m, 2H), 0.95 (s, 3H), 1.53–1.35 (m, 5H), 1.29–0.99 (m, 5H), 0.88 (d, J = 10.0 Hz, 6H); ^{13}C NMR (101 MHz, $CDCl_3$) δ 163.6, 134.3, 129.1, 123.3, 110.1, 84.1, 60.5, 57.0, 42.4, 39.7, 36.2, 33.5, 33.1, 28.9, 23.0, 21.0, 20.6, 18.3, 15.2. HRMS (ESI, m/z) calcd for $C_{24}H_{35}N_2O_4$ $(M+NH_4)^+$: 415.2591, found: 415.2596.

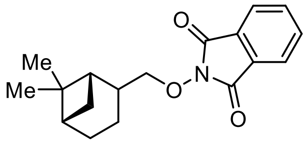

2-(((1R,5R)-6,6-dimethylbicyclo[3.1.1]heptan-2-yl)methoxy)isoindoline-1,3-dione (2ab)

Following the general method A, the reaction of **2ab-1** (926 mg, 6 mmol) afforded *N*-alkoxyphthalimides **2ab** as a white solid (0.90 g, 50% yield). ^{1}H NMR (400 MHz, $CDCl_3$) δ 7.84 (m, 2H), 7.76 (m, 2H), 4.19 (m, J = 7.3 Hz, 2H), 2.61 (m, 1H), 2.40 (m, 1H), 2.19–1.86 (m, 5H), 1.69 (m, J = 6.3 Hz, 1H), 1.23 (s, 3H), 1.01 (s, 3H); ^{13}C NMR (101 MHz, $CDCl_3$) δ 163.5, 134.4, 128.9, 123.3, 83.3, 43.1, 41.1, 39.6, 38.4, 32.5, 27.7, 25.8, 23.2, 18.6. HRMS (ESI, m/z) calcd for $C_{18}H_{22}NO_3$ $(M+H)^+$: 300.1594, found: 300.1599.

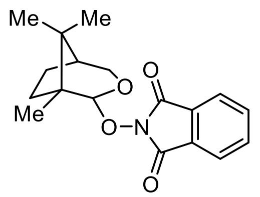

2-(((1S,2R,5S)-1,8,8-trimethyl-3-oxabicyclo[3.2.1]octan-2-yl)oxy)isoindoline-1,3-dione (2ac)

Following the general method C, the reaction of **2ac-1** (340 mg, 2 mmol) afforded *N*-alkoxyphthalimides **2ac** as a white solid (252 mg, 40% yield). m.p. 138–139 °C; ^{1}H NMR (400 MHz, $CDCl_3$) δ 7.82 (m, 2H), 7.73 (m, 2H), 4.96 (s, 1H), 4.61 (d, J = 10.7 Hz, 1H), 3.45 (d, J = 10.7 Hz, 1H), 1.93–1.83 (m, 1H), 1.78–1.57 (m, 5H), 1.47 (s, 3H), 1.20 (s, 3H), 0.94 (s, 3H); ^{13}C NMR (101 MHz, $CDCl_3$) δ 163.8, 134.2, 129.2, 123.3, 112.4, 67.8, 46.5, 45.7, 40.9, 35.4, 25.5, 24.5, 20.6, 15.4. HRMS (ESI, m/z) calcd for $C_{18}H_{21}NO_4Na$ $(M+Na)^+$: 338.1363, found: 338.1362.

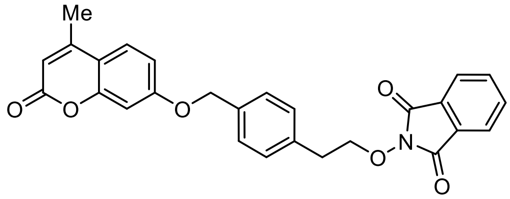

2-(4-(((4-methyl-2-oxo-2H-chromen-7-yl)oxy)methyl)phenethoxy)isoindoline-1,3-dione (2ae)

Following the general method A, the reaction of **2ae-1** (472 mg, 2 mmol) afforded *N*-alkoxyphthalimides **2ae** as a white solid (0.49 g, 65% yield). m.p. 196–200 °C; ^{1}H NMR (400 MHz, $CDCl_3$) δ 7.88–7.83 (m, 2H), 7.77 (m, 2H), 7.52 (d, J = 8.8 Hz, 1H), 7.43–7.34 (m, 4H), 6.98–6.87 (m, 2H), 6.15 (s, 1H), 5.11 (s, 2H), 4.46 (t, J = 7.4 Hz, 2H), 3.19 (t, J = 7.3 Hz, 3H), 2.41 (s, 3H); ^{13}C NMR (101 MHz, $CDCl_3$) δ 163.6, 161.7, 161.3, 155.2, 152.5, 137.2, 134.5, 134.2, 129.3, 128.9, 127.9, 125.6, 123.6, 113.8, 112.9, 112.1, 101.9, 78.4, 70.3, 34.4, 18.7. HRMS (ESI, m/z) calcd for $C_{27}H_{22}NO_6$ $(M+H)^+$: 456.1442, found: 456.1450.

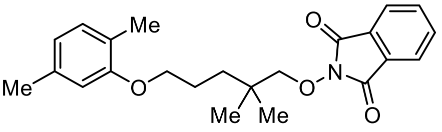

2-((5-(2,5-dimethylphenoxy)-2,2-dimethylpentyl)oxy)isoindoline-1,3-dione (2af)

Following the general method A, the reaction of **2af-1** (472 mg, 2 mmol) afforded *N*-alkoxyphthalimides **2af** as a colorless oil (0.49 g, 65% yield). ^{1}H NMR (400 MHz, $CDCl_3$) δ 7.85 (m, 2H), 7.76 (m, 2H), 7.03 (d, J = 7.7 Hz, 1H), 6.68 (m, 2H), 4.00 (m, 4H), 2.35 (s, 3H), 2.22 (s, 3H), 1.93 (m, J = 2.8 Hz, 2H), 1.65 (m, J = 4.2 Hz, 2H), 1.15 (s, 6H); ^{13}C NMR (101 MHz, $CDCl_3$) δ 163.4, 157.1, 136.4, 134.4, 130.3, 129.3, 123.6, 123.4, 120.6, 112.1, 86.1, 68.4, 35.3, 34.4, 24.4, 24.1, 21.4, 15.8. HRMS (ESI, m/z) calcd for $C_{23}H_{28}NO_4$ $(M+H)^+$: 382.2013, found: 382.2014.

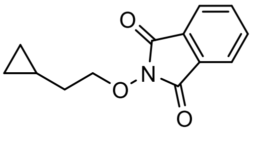

2-(2-cyclopropylethoxy)isoindoline-1,3-dione (2y)

Following the general method A, the reaction of **2y-1** (517 mg, 6 mmol) afforded *N*-alkoxyphthalimides **2y** as a white solid (1.0 g, 72% yield). m.p. 63–65 °C; ^{1}H NMR (400 MHz, $CDCl_3$) δ 7.85 (m, 2H), 7.76 (m, 2H), 4.29 (t, *J* = 6.9 Hz, 1H), 1.71 (q, *J* = 6.8 Hz, 1H), 0.89 (t, *J* = 6.3 Hz, 1H), 0.51 (d, *J* = 7.6 Hz, 1H), 0.13 (d, *J* = 4.6 Hz, 1H); ^{13}C NMR (101 MHz, $CDCl_3$) *δ* 163.7, 134.4, 129.0, 123.5, 78.6, 33.1, 7.2, 4.3. HRMS (ESI, m/z) calcd for $C_{13}H_{14}NO_3$ $(M+H)^+$: 232.0968, found: 232.0972.

2.2 Experimental Procedures

2.2.1 General Procedure for the Synthesis of the products 3–62

The *N*-alkoxyphthalimides (0.1 mmol), the esters, or amides of *N*-aryl-substituted glycine, or *N*-aryl tetrahydroisoquinoline, or peptides (0.2 mmol, 2.0 equiv.), 4CzIPN (5 mol%, 0.005 mmol, 4.0 mg), and DMSO (3 mL, 0.03 M) were added sequentially to a 4 mL clear-colored glass vial equipped with a magnetic stir bar. After bubbled with nitrogen gas for 5 minutes to remove oxygen, the vial was sealed and exposed to blue LEDs at 60 ºC (Supplementary Figure 1). The reaction mixture was monitored by TLC until the starting material *N*-alkoxyphthalimides were consumed. Then, the reaction was quenched with water (2 mL), extracted with ethyl acetate, washed with brine, dried over anhydrous Na_2SO_4, concentrated in vacuo, and purified by column chromatography to yield the products **3-62**.

Details for the photoreactor setup

The reaction vial was placed in a 60 ºC oil bath (the oil level height is 0.5 cm) at the center of a stir plate. Two parallel Blue LED lamps (E27PAR30/38, 15W, were purchased from https://m.tb.cn/h.U2gWmW5?tk=UErD2y9tSM1) were placed perpendicular to the sidewall of reaction vial so that the reaction vial can be equally exposed to the LEDs (at approximately 4 cm away from the light source, λ = 460–470 nm, 100–110 lm/W). A fan opposite to the reaction vial is always turned on during the reaction to offset the heat generated by the LED light.

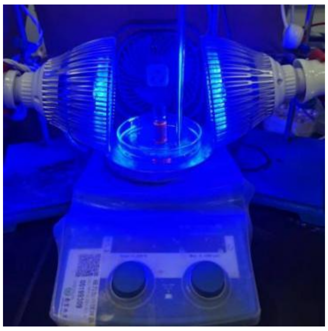

Supplementary Figure 1. Photoreactor setup

2.2.2 Characterization Data for the product 3–62.

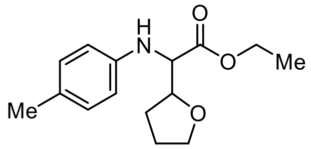

3: Yellow oil (24.3 mg, 92% yield); R_f = 0.40 (Petroleum ether/EtOAc, 5:1); ^{1}H NMR (400 MHz, $CDCl_3$) δ 7.00 (d, J = 7.9 Hz, 2H), 6.64 (d, J = 7.9 Hz, 1H), 6.59 (d, J = 7.9 Hz, 1H), 4.39–3.75 (m, 7H), 2.25 (s, 3H), 2.10–1.86 (m, 4H), 1.26 (t, J = 7.2 Hz, 3H); ^{13}C NMR (101 MHz, $CDCl_3$) δ 172.7, 172.4, 145.1, 144.6, 129.8, 129.7,127.8, 127.6, 114.1, 113.9, 79.9, 79.4, 69.2, 68.7, 61.2 (2C), 61.1, 60.2, 28.4, 28.0, 26.0, 25.6, 20.4, 14.2 (2C). Spectra are consistent with reported literature[27] values.

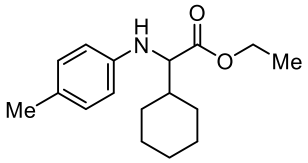

4: Yellow oil (24.5 mg, 89% yield); R_f = 0.67 (Petroleum ether/EtOAc, 5:1); ^{1}H NMR (400 MHz, $CDCl_3$) δ 6.99 (d, J = 8.0 Hz, 2H), 6.57 (d, J = 8.0 Hz, 2H), 4.18 (q, J = 7.1 Hz 2H), 4.02 (s, 1H), 3.84 (d, J = 4.8 Hz 1H), 2.24 (s, 3H), 1.87–1.68 (m, 6H),1.29–1.15 (m, 6H); ^{13}C NMR (101 MHz, $CDCl_3$) δ 173.9, 145.2, 129.8, 127.3, 113.7, 62.5, 60.7, 41.3, 29.6, 29.2, 26.1, 20.4, 14.3. Spectra are consistent with reported literature[1] values.

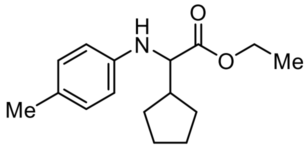

5: Yellow oil (46.4 mg, 89% yield); R_f = 0.62 (Petroleum ether/EtOAc, 5:1); ^{1}H NMR (400 MHz, $CDCl_3$) δ 6.99 (d, J = 7.9 Hz, 2H), 6.58 (d, J = 7.8 Hz, 2H), 4.17 (q, J = 7.1 Hz 2H), 4.02 (s, 1H), 3.85 (d, J = 7.8 Hz, 1H), 2.25 (s, 3H), 1.92–1.42 (m, 9H), 1.25 (t, J = 7.2 Hz, 6H); ^{13}C NMR (101 MHz, $CDCl_3$) δ 174.4, 145.1, 129.8, 127.5, 113.7, 61.3, 60.7, 43.2, 29.4, 29.1, 25.4, 25.1, 20.4, 14.3. Spectra are consistent with reported literature[1] values.

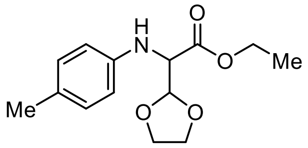

6: Yellow oil (30.7 mg, 60% yield); R_f = 0.32 (Petroleum ether/EtOAc, 5:1); ^{1}H NMR (400 MHz, $CDCl_3$) δ 7.00 (d, J = 7.7 Hz, 2H), 6.65 (d, J = 7.6 Hz, 2H), 5.38 (s, 1H), 4.35 (s, 1H), 4.25 (q, J = 7.6 Hz, 2H), 4.15–3.91 (m, 4H), 2.25 (s, 3H), 1.29 (t, J = 6.4 Hz, 3H); ^{13}C NMR (101 MHz, $CDCl_3$) δ 170.5, 144.7, 129.7, 127.9, 114.1, 103.5, 66.0, 65.6, 61.6, 60.7, 20.4, 14.2. Spectra are consistent with reported literature[28] values.

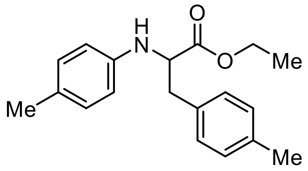

7: Yellow oil (21.7 mg, 73% yield); R_f = 0.68 (Petroleum ether/EtOAc, 5:1); ^{1}H NMR (400 MHz, $CDCl_3$) δ 7.11 (q, J = 7.2 Hz, 4H), 7.00 (d, J = 7.9 Hz, 2H), 6.56 (d, J = 7.8 Hz, 2H), 4.32 (t, J = 6.1 Hz, 1H), 4.14 (q, J = 6.7 Hz, 2H), 4.06 (s, 1H), 3.11 (t, J = 5.4 Hz, 2H), 2.34 (s, 3H), 2.26 (s, 3H), 1.21 (t, J = 7.1 Hz, 3H); ^{13}C NMR (101 MHz, $CDCl_3$) δ 173.4, 144.2, 136.5, 133.3, 129.8, 129.2 (2C), 127.6, 113.8, 61.0, 58.2, 38.3, 21.1, 20.4, 14.2. HRMS (ESI, m/z) calcd for $C_{19}H_{24}NO_2$ $(M+H)^+$: 298.1802, found: 298.1794.

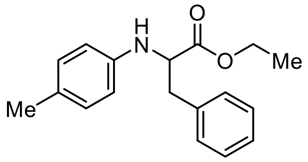

8: Yellow oil (13 mg, 46% yield); R_f = 0.79 (Petroleum ether/EtOAc, 5:1); ^{1}H NMR (400 MHz, $CDCl_3$) δ 7.29 (q, J = 7.3 Hz, 3H), 7.21 (d, J = 7.2 Hz, 2H), 7.01 (d, J = 8.1 Hz, 2H), 6.57 (d, J = 8.2 Hz, 2H), 4.34 (t, J = 6.3 Hz, 1H), 4.14 (dq, J = 2.2, 7.0 Hz, 2H), 4.13 (s, 1H), 3.14 (d, J = 6.3 Hz, 2H), 2.26 (s, 3H), 1.19 (t, J = 7.1 Hz, 3H); ^{13}C NMR (101 MHz, $CDCl_3$) δ 173.3, 144.1, 136.5, 129.8, 129.3, 128.5, 127.7, 126.9, 113.8, 61.0, 58.2, 38.7, 20.4, 14.1. HRMS (ESI, m/z) calcd for $C_{18}H_{22}NO_2$ $(M+H)^+$: 284.1645, found: 284.1646.

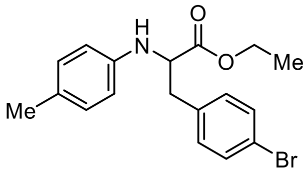

9: Yellow oil (13 mg, 54% yield); R_f = 0.55 (Petroleum ether/EtOAc, 5:1); ^{1}H NMR (600 MHz, $CDCl_3$) δ 7.44 (d, J = 8.1 Hz, 2H), 7.08 (d, J = 8.1 Hz, 2H), 7.02 (d, J = 8.0 Hz, 2H), 6.58 (d, J = 8.1 Hz, 2H), 4.33 (t, J = 6.2 Hz, 1H), 4.14 (q, J = 6.9 Hz, 2H), 3.10 (m, J = 6.4 Hz, 2H), 2.27 (s, 3H), 1.22 (t, J = 7.1 Hz, 3H); ^{13}C NMR (151 MHz, $CDCl_3$) δ 173.0, 143.8, 135.5, 131.5, 131.1, 129.9, 127.9, 120.9, 113.9, 61.2, 58.0, 38.0, 20.4, 14.2. HRMS (ESI, m/z) calcd for $C_{18}H_{20}BrNO_2$ $(M+H)^+$: 362.0750, found: 362.0744.

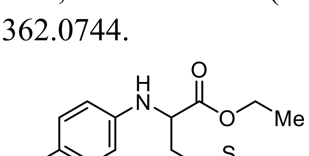

10: Yellow oil (23.7 mg, 82% yield); R_f = 0.58 (Petroleum ether/EtOAc, 5:1); ^{1}H NMR (400 MHz, $CDCl_3$) δ 7.19 (d, J = 4.7 Hz, 1H), 7.03 (d, J = 7.7 Hz, 2H), 6.96 (d, J = 2.8 Hz, 1H), 6.87 (s, 1H), 6.60 (d, J = 7.6 Hz, 2H), 4.36 (t, J = 5.7 Hz, 1H), 4.19 (q, J = 7.1 Hz, 2H), 3.39 (t, J = 7.3 Hz, 2H), 2.27 (s, 3H), 1.26 (t, J = 7.1 Hz, 3H); ^{13}C NMR (101 MHz, $CDCl_3$) δ 172.7, 143.9, 138.1, 129.9, 127.8, 126.8, 126.6, 124.8, 114.0, 61.3, 58.0,

32.8, 20.5, 14.3, 14.2. HRMS (ESI, m/z) calcd for $C_{16}H_{20}NO_2S$ (M+H)$^+$: 290.1209, found: 290.1211.

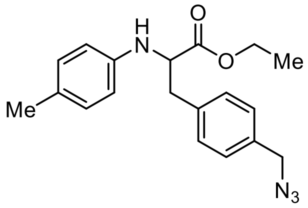

11: Yellow oil (28.7 mg, 85% yield); R_f = 0.36 (Petroleum ether/EtOAc, 5:1); ^{1}H NMR (400 MHz, $CDCl_3$) δ 7.25 (q, J = 10.4 Hz, 4H), 7.01 (d, J = 8.1 Hz, 2H), 6.57 (d, J = 8.4 Hz, 2H), 4.34 (d, J = 6.2 Hz, 3H), 4.13 (t, J = 7.2 Hz, 2H), 3.15 (q, J = 3.4 Hz, 2H), 2.26 (s, 3H), 1.19 (t, J = 7.1 Hz, 3H); ^{13}C NMR (101 MHz, $CDCl_3$) δ 173.2, 144.0, 136.8, 134.0, 129.9, 129.8, 128.4, 127.8, 113.9, 61.1, 58.1, 54.5, 38.4, 20.4, 14.2. HRMS (ESI, m/z) calcd for $C_{19}H_{23}N_4O_2$ (M+H)$^+$: 339.1816, found: 339.1818.

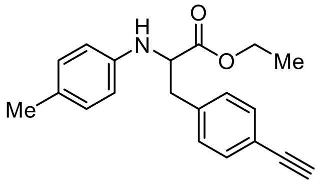

12: Yellow oil (20 mg, 65% yield); R_f = 0.54 (Petroleum ether/EtOAc, 5:1); ^{1}H NMR (400 MHz, $CDCl_3$) δ 7.43 (d, J = 7.8 Hz, 2H), 7.16 (d, J = 7.8 Hz, 2H), 7.01 (d, J = 7.7 Hz, 2H), 6.58 (d, J = 7.8 Hz, 2H), 4.33 (t, J = 6.0 Hz, 1H), 4.13 (t, J = 3.5 Hz, 2H), 3.14 (d, J = 5.9 Hz, 2H), 3.08 (s, 1H), 2.26 (s, 3H), 1.19 (t, J = 7.1 Hz, 3H); ^{13}C NMR (151 MHz, $CDCl_3$) δ 173.0, 143.9, 137.5, 132.2, 129.9, 129.4, 127.9, 120.7, 113.9, 83.5, 77.2, 61.2, 58.0, 38.5, 20.4, 14.2. HRMS (ESI, m/z) calcd for $C_{20}H_{22}NO_2$ (M+H)$^+$: 308.1645, found: 308.1635.

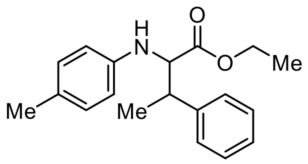

13: Yellow oil (18.5 mg, 65% yield); R_f = 0.62 (Petroleum ether/EtOAc, 5:1); ^{1}H NMR (400 MHz, $CDCl_3$) δ 7.37–7.23 (m, 10H), 6.97 (d, J = 7.9 Hz, 4H), 6.53 (t, J = 7.9 Hz, 6H), 4.22–4.12 (m, 4H), 4.08 (s, 1H), 3.96 (q, J = 6.8 Hz, 2H), 4.08 (s, 1H), 3.32 (m, J = 6.9 Hz, 1H), 3.20 (m, J = 6.9 Hz, 1H), 2.24 (s, 6H), 1.48 (d, J = 7.1Hz, 3H), 1.43 (d, J = 7.0 Hz, 3H), 1.24 (t, J = 7.1 Hz, 3H), 1.0 (t, J = 7.12 Hz, 3H); ^{13}C NMR (101 MHz, $CDCl_3$) δ 173.5, 173.2, 144.9, 144.8, 142.3, 141.9, 129.8, 129.7, 128.6, 128.4, 127.9 (2C), 127.7, 127.6, 127.1, 127.0, 114.1, 113.9, 63.6, 63.0, 61.0, 60.7, 43.3, 42.6, 20.4, 18.4, 17.5, 14.2, 13.9. HRMS (ESI, m/z) calcd for $C_{19}H_{24}NO_2$ (M+H)$^+$: 298.1802, found: 298.1802.

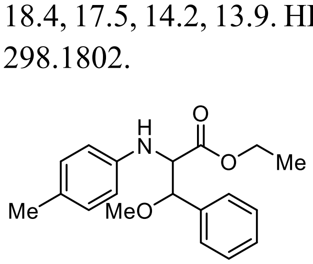

14: Yellow oil (22.8 mg, 73% yield); R_f = 0.41 (Petroleum ether/EtOAc, 5:1); ^{1}H NMR (400 MHz, $CDCl_3$) δ 7.41–7.31 (m, 5H), 6.98 (d, *J* = 8.0 Hz, 1H), 6.92 (d, *J* = 7.9 Hz, 1H), 6.60 (d, *J* = 7.9 Hz, 1H), 6.44 (d, *J* = 7.9 Hz, 1H), 4.65 (dd, *J* = 25.1, 5.1 Hz, 1H), 4.30–4.05 (m, 3H), 3.36–3.30 (m, 3H), 2.22 (d, *J* = 10.4 Hz, 3H), 1.15 (dt, *J* = 20.6, 7.2 Hz, 3H); ^{13}C NMR (101 MHz, $CDCl_3$) δ 171.9, 171.5, 144.8, 137.7, 137.4, 129.8, 129.6, 128.5, 128.4, 128.4, 128.3, 128.3, 127.7, 127.4, 127.1, 114.6, 114.0, 83.9, 83.8, 63.7, 63.2, 61.2, 61.0, 57.6, 57.4, 20.4, 20.4, 14.1, 14.0. HRMS (ESI, m/z) calcd for $C_{19}H_{24}NO_3$ $(M+H)^+$: 314.1751, found: 314.1750.

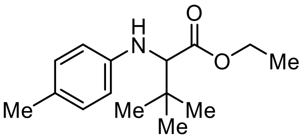

15: Yellow oil (15.6 mg, 63% yield); R_f = 0.79 (Petroleum ether/EtOAc, 5:1); ^{1}H NMR (400 MHz, $CDCl_3$) δ 7.00 (d, *J* = 7.96 Hz, 2H), 6.61 (d, *J* = 7.96 Hz, 2H), 4.16 (q, *J* = 7.06 Hz, 2H), 4.09 (d, *J* = 5.72 Hz, 1H), 3.76 (s, 1H), 2.25 (s, 3H), 1.25 (t, *J*=7.13 Hz, 3H), 1.08 (s, 9H); ^{13}C NMR (101 MHz, $CDCl_3$) δ 173.5, 145.4, 129.8, 127.6, 114.1, 66.0, 60.5, 34.4, 26.8, 20.4, 14.3. HRMS (ESI, m/z) calcd for $C_{15}H_{24}NO_2$ $(M+H)^+$: 250.1802, found: 250.1802.

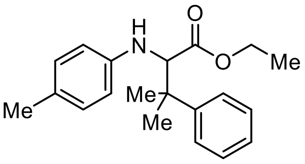

16: Yellow oil (14.4 mg, 46% yield); R_f = 0.60 (Petroleum ether/EtOAc, 5:1); ^{1}H NMR (400 MHz, $CDCl_3$) δ 7.44 (d, *J* = 7.9 Hz, 2H), 7.35 (t, *J* = 7.6 Hz, 2H), 7.26 (t, J=7.9 Hz, 1 H), 6.97 (d, *J* = 7.9 Hz, 2H), 6.54 (d, *J* = 7.9 Hz, 2H), 4.14 (d, *J* = 6.6 Hz, 1H), 4.02–3.92 (m, 3H), 2.24 (s, 3H), 1.54 (d, *J* = 11.9 Hz, 6H), 1.03 (d, *J* = 7.1 Hz, 3H); ^{13}C NMR (101 MHz, $CDCl_3$) δ 172.9, 145.5, 145.2, 129.7, 128.1, 127.7, 126.5 (2C), 114.2, 66.7, 60.5, 41.5, 25.7, 25.1, 20.4, 13.9. HRMS (ESI, m/z) calcd for $C_{20}H_{26}NO_2$ $(M+H)^+$: 312.1958, found: 312.1960.

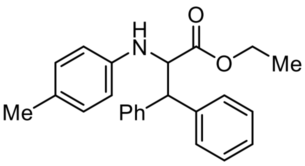

17: Yellow oil (12.6 mg, 35% yield); R_f = 0.59 (Petroleum ether/EtOAc, 5:1); ^{1}H NMR (400 MHz, $CDCl_3$) δ 7.40–7.20 (m, *J* = 7.7 Hz, 10H), 7.00 (d, *J* = 7.8 Hz, 2H), 6.57 (d, *J* = 7.8 Hz, 2H), 4.74 (d, *J* = 9.1 Hz, 1H), 4.42 (d, *J* = 9.2 Hz, 1H), 3.94 (m, *J* = 5.9 Hz, 2H), 2.25 (s, 3H), 0.97 (t, *J* = 7.1 Hz, 3H); ^{13}C NMR (101 MHz, $CDCl_3$) δ 173.4, 144.3, 140.8, 140.0, 129.8, 128.7, 128.6, 128.5 (2C), 127.9, 127.1 (2C), 113.8, 61.1, 60.9, 54.3, 20.4, 13.8. HRMS (ESI, m/z) calcd for $C_{24}H_{26}NO_2$ $(M+H)^+$: 360.1958, found: 360.1956.

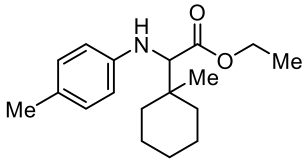

18: Yellow oil (12.4 mg, 43% yield); R_f = 0.75 (Petroleum ether/EtOAc, 5:1); ^{1}H NMR (400 MHz, $CDCl_3$) δ 6.99 (d, J = 7.8 Hz, 2H), 6.61 (d, J = 7.9 Hz, 2H), 4.15 (q, J = 7.0 Hz, 2H), 4.09 (s, 1H), 4.06 (s, 1H), 3.92 (s, 1H), 2.25 (s, 1H), 1.25 (t, J = 7.1 Hz, 3H), 1.69–1.30 (m, 10H), 1.05 (s, 3H); ^{13}C NMR (101 MHz, $CDCl_3$) δ 173.5, 145.5, 129.8, 127.5, 114.1, 64.9, 60.5, 37.1, 34.9, 26.1, 21.8, 21.7, 20.4, 14.3. HRMS (ESI, m/z) calcd for $C_{18}H_{28}NO_2$ $(M+H)^+$: 290.2115, found: 290.2120.

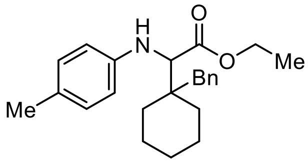

19: Yellow oil (12.8 mg, 35% yield); R_f = 0.69 (Petroleum ether/EtOAc, 5:1); ^{1}H NMR (400 MHz, $CDCl_3$) δ 7.34–7.22 (m, 5H), 6.98 (d, J = 7.9 Hz, 2H), 6.50 (d, J = 7.9 Hz, 2H), 4.14 (q, J = 7.1 Hz, 2H), 4.02 (s, 1H), 2.99 (d, J = 13.4 Hz, 1H), 2.86 (d, J = 13.4 Hz, 1H), 2.25 (s, 3H), 1.79–1.32 (m, 10H), 1.22 (t, J = 7.1 Hz, 3H); ^{13}C NMR (101 MHz, $CDCl_3$) δ 173.8, 145.0, 138.2, 131.1, 129.8, 128.0, 127.6, 126.2, 114.3, 61.2, 60.5, 40.6, 31.6, 30.8, 26.0, 21.8, 21.5, 20.4, 14.3. HRMS (ESI, m/z) calcd for $C_{24}H_{32}NO_2$ $(M+H)^+$: 366.2428, found: 366.2419.

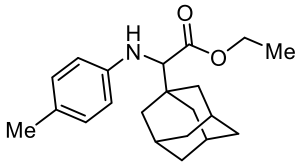

20: Yellow oil (20.5 mg, 31% yield); R_f = 0.80 (Petroleum ether/EtOAc, 5:1); ^{1}H NMR (400 MHz, $CDCl_3$) δ 6.99 (d, J = 8.0 Hz, 2H), 6.60 (d, J = 8.0 Hz, 2H), 4.16 (q, J = 7.1 Hz, 2H), 4.10 (s, 1H), 3.64 (s, 1H), 2.25 (s, 3H), 2.05 (s, 3H), 1.85–1.56 (m, 15H) 1.26 (t, J =7.3 Hz, 3H); ^{13}C NMR (101 MHz, $CDCl_3$) δ 173.0, 145.6, 129.7, 127.4, 114.0, 66.9, 60.5, 39.1, 36.9, 36.3, 28.4, 20.4, 14.4. HRMS (ESI, m/z) calcd for $C_{21}H_{30}NO_2$ $(M+H)^+$: 328.2271, found: 328.2268.

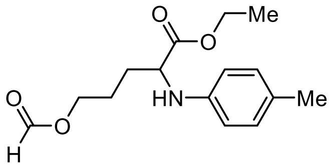

21: Yellow oil (15.9 mg, 69% yield); R_f = 0.26 (Petroleum ether/EtOAc, 5:1); ^{1}H NMR (400 MHz, $CDCl_3$) δ 8.07 (s, 1H), 7.01 (d, J = 7.9 Hz, 2H), 6.57 (d, J = 7.9 Hz, 2H), 4.27–4.16 (m, 4H), 4.07 (s, 2H), 2.25 (s, 3H), 2.01–1.79 (m, 4H), 1.27 (t, J = 7.0 Hz, 3H); ^{13}C NMR (101 MHz, $CDCl_3$) δ 173.9, 161.0, 144.4, 129.9, 127.8, 113.8, 63.4, 61.2, 56.6, 29.5, 24.8, 20.4, 14.2. HRMS (ESI, m/z) calcd for $C_{15}H_{21}NO_4$ $(M+H)^+$: 280.1543,

found: 280.1544.

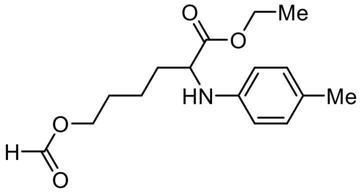

22: Yellow oil (21.2 mg, 72% yield); R_f = 0.30 (Petroleum ether/EtOAc, 5:1); ^{1}H NMR (400 MHz, $CDCl_3$) δ 8.07 (s, 1H), 7.00 (d, J = 7.8 Hz, 2H), 6.57 (d, J = 7.9 Hz, 2H), 4.27–4.14 (m, 4H), 4.05 (t, J = 6.0 Hz, 1H), 2.25 (s, 3H), 1.95–1.67 (m, 4H), 1.62–1.49 (m, 2H), 1.27 (t, J = 7.0 Hz, 3H); ^{13}C NMR (101 MHz, $CDCl_3$) δ 174.1, 161, 144.5, 129.8, 127.7, 113.7, 63.6, 61.1, 56.9, 32.6, 28.2, 22.0, 20.4, 14.3. HRMS (ESI, m/z) calcd for $C_{16}H_{24}NO_4$ $(M+H)^+$: 294.1700, found: 294.1696.

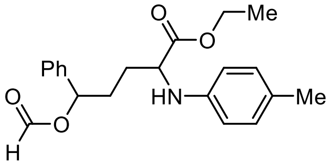

23: Yellow oil (26.8 mg, 78% yield); R_f = 0.52 (Petroleum ether/EtOAc, 5:1); ^{1}H NMR (400 MHz, $CDCl_3$) δ 8.12 (s, 1H), 7.33 (m, 5H), 7.00 (d, J = 7.9 Hz, 2H), 6.55 (d, J = 7.9 Hz, 2H), 5.91 (m, 1H), 4.19 (m, J= 3.5 Hz, 2H), 4.02 (m, 2H), 2.26 (s, 3H), 2.14-1.60 (m, 4H), 1.25 (m, J = 4.5 Hz, 3H); ^{13}C NMR (101 MHz, $CDCl_3$) δ 173.8, 160.3, 144.4 (2C), 139.4, 139.3, 129.9, 128.7, 128.4 (2C), 127.8 (2C), 126.6, 126.5, 113.9, 75.4, 75.3, 61.2, 56.8, 56.6, 32.3, 32.2, 29.0, 28.9, 20.4, 14.2. HRMS (ESI, m/z) calcd for $C_{21}H_{26}NO_4$ $(M+H)^+$: 356.1856, found: 356.1850.

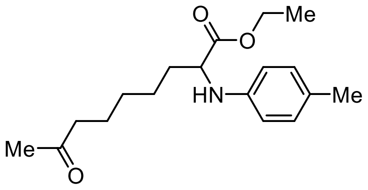

24: Yellow oil (7.1 mg, 23% yield); R_f = 0.45 (Petroleum ether/EtOAc, 5:1); ^{1}H NMR (400 MHz, $CDCl_3$) δ 7.01 (d, J = 7.6 Hz, 2H), 6.59 (d, J = 7.7 Hz, 2H), 4.28 (d, J = 6.6 Hz, 1H), 4.18 (d, J = 7.1 Hz, 2H), 4.10(s, 1H), 2.25 (d, J = 5.4 Hz, 6H), 1.92–1.50 (m, 8H), 1.25 (t, J = 7.4 Hz, 3H), 0.90 (t, J = 6.0 Hz, 2H); ^{13}C NMR (101 MHz, $CDCl_3$) δ 209.7, 172.7, 144.3, 129.8, 128.0, 114.0, 61.4, 58.1, 55.1, 30.1, 29.7, 27.6, 22.8, 22.7, 20.4, 14.1, 13.8. HRMS (ESI, m/z) calcd for $C_{18}H_{28}NO_3$ $(M+H)^+$: 306.2064, found: 306.2060.

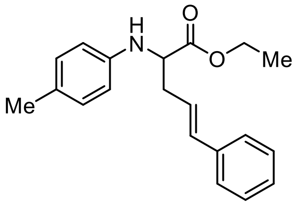

25: Yellow oil (23.8 mg, 77% yield); R_f = 0.54 (Petroleum ether/EtOAc, 5:1). Spectra are consistent with reported literature[26] values.

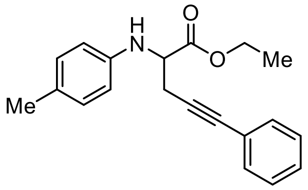

26: Yellow oil (20 mg, 65% yield); R_f = 0.43 (Petroleum ether/EtOAc, 5:1); ^{1}H NMR (400 MHz, $CDCl_3$) δ 7.44–7.30 (m Hz, 5H), 7.04 (d, J = 7.6 Hz, 2H), 6.64 (d, J = 7.5 Hz, 2H), 4.38–4.23 (m, 3H), 3.00 (d, J = 5.3 Hz, 2H), 2.27 (s, 3H), 1.31 (t, J = 7.0 Hz, 3H); ^{13}C NMR (101 MHz, $CDCl_3$) δ 172.3, 143.9, 131.7, 129.9, 129.8, 128.2, 128.1, 128.0, 123.2, 114.2, 84.3, 83.6, 61.5, 55.9, 23.9, 20.4, 14.3, 14.2. HRMS (ESI, m/z) calcd for $C_{20}H_{22}NO_2$ $(M+H)^+$: 308.1645, found: 308.1653.

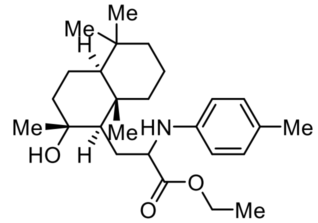

27: Yellow oil (23.8 mg, 57% yield); R_f = 0.82 (Petroleum ether/EtOAc, 5:1); ^{1}H NMR (400 MHz, $CDCl_3$) δ 6.99 (d, J = 7.8 Hz, 2H), 6.61 (t, J = 7.8 Hz, 2H), 4.28–3.90 (m, 4H), 2.25 (s, 3H), 1.80–1.50 (m, 6H), 1.49–1.33 (m, 4H), 1.27–1.19 (m, 3H), 1.07 (s, 3H), 1.04–0.96 (m, 2H), 0.94–0.87 (m, 6H), 0.84 (s, 3H), 0.83–0.77 (m, 2H); ^{13}C NMR (101 MHz, $CDCl_3$) δ 173.9, 173.7, 145.6, 145.3, 129.8, 129.7, 127.6, 127.5, 114.2, 114.2, 114.2, 65.3, 64.1, 60.4, 60.3, 56.0, 55.9, 50.1, 47.0, 41.9, 41.9, 40.6, 40.5 (2C), 38.3, 38.2, 34.8, 33.3 (2C), 33.1, 21.7, 21.6, 20.4, 19.3, 18.6, 18.3, 18.2, 18.1, 15.7, 15.4, 14.3, 8.7, 8.3. HRMS (ESI, m/z) calcd for $C_{26}H_{40}NO_2$ $(M–H_2O+H)^+$: 398.3054, found: 398.3044.

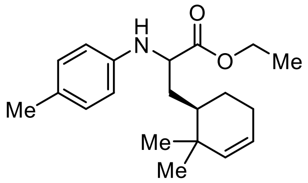

28: Yellow oil (16.7 mg, 53% yield); R_f = 0.79 (Petroleum ether/EtOAc, 5:1); ^{1}H NMR (400 MHz, $CDCl_3$) δ 7.00 (d, J = 7.8 Hz, 2H), 6.61 (d, J = 7.8 Hz, 2H), 5.69 (s, 2H), 4.12 (m, 3H), 2.25 (s, 3H), 1.98 (m, 7H), 1.24 (t, J = 7.1 Hz, 3H), 1.07 (s, 1H), 1.04 (s, 2H), 0.95 (m, 3H); ^{13}C NMR (101 MHz, CDCl3) δ 173.8, 145.4, 145.2, 129.8, 127.6, 127.6, 127.0, 126.9 (3C), 114.0 (2C), 62.5, 62.4, 60.5, 40.4, 39.6, 39.2, 39.1, 26.6 (2C), 26.4, 24.1, 23.7, 20.4, 20.3, 20.2 (2C), 20.0, 14.3 (2C). HRMS (ESI, m/z) calcd for $C_{20}H_{30}NO_2$ $(M+H)^+$: 316.2271, found: 316.2270.

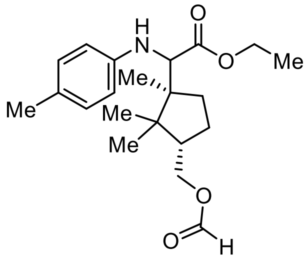

29: Yellow oil (17.7 mg, 50% yield); R_f = 0.49 (Petroleum ether/EtOAc, 5:1); ^{1}H NMR (400 MHz, $CDCl_3$) δ 8.07 (m, 1H), 7.00 (m, 2H), 6.61 (m, 2H), 4.17 (m, 5H), 2.40–2.19 (m, 4H), 2.03–1.33 (m, 4H), 1.28–0.79 (m, 12H); ^{13}C NMR (101 MHz, $CDCl_3$) δ 174.0, 173.7, 173.5, 173.4, 161.2 (2C), 144.5, 144.3, 144.2, 129.9, 129.8 (2C), 128.0 (2C), 127.8 (2C), 114.3, 114.0, 113.9, 66.7, 66.4, 66.1, 66.0, 62.1, 62.0, 61.7, 61.4, 60.6 (2C), 60.5, 51.4, 50.9, 49.1, 48.5, 47.7, 47.6, 46.9, 46.4, 46.2, 46.1, 45.9, 44.7, 36.1, 35.8, 35.3, 31.9, 26.9 (2C), 26.4, 25.2, 24.4, 24.0, 23.9, 22.1, 20.7 (2C), 20.4, 19.8, 18.5, 18.2, 17.4, 16.9, 16.4, 14.3 (2C), 14.2, 14.1. HRMS (ESI, m/z) calcd for $C_{21}H_{32}NO_4$ $(M+H)^+$: 362.2326, found: 362.2329.

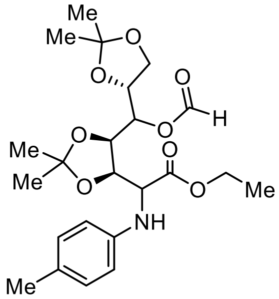

30: Yellow oil (33.4 mg, 74% yield); R_f = 0.32 (Petroleum ether/EtOAc, 5:1); ^{1}H NMR (400 MHz, $CDCl_3$) δ 8.24 (m, 1H), 7.01 (m, 2H), 6.63 (m, 2H), 5.37–5.25 (m, 8H), 4.40–3.90 (m, 8H), 2.25 (s, 1H), 1.50–1.21 (m, 15H); ^{13}C NMR (101 MHz, $CDCl_3$) δ 171.6, 171.5, 160.5, 160.1, 144.7, 143.9, 129.9, 129.8, 128.5, 128.2, 114.5, 114.1, 110.5, 110.1, 109.8, 109.5, 78.1, 77.7, 77.1, 76.6, 75.0, 74.9, 71.8, 71.5, 71.3, 71.0, 67.3, 67.0, 66.3, 66.0, 61.5 (2C), 61.4, 60.0, 56.3, 27.2, 27.0, 26.8, 26.6, 26.5, 25.4, 25.3, 20.4 (2C), 14.2. HRMS (ESI, m/z) calcd for $C_{23}H_{34}NO_8$ $(M+H)^+$: 452.2279, found: 452.2288.

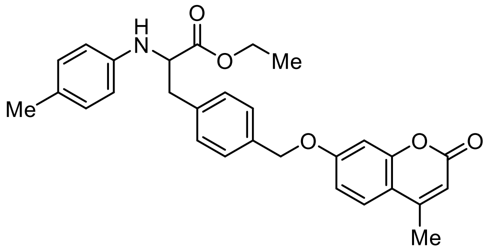

31: Yellow oil (33.4 mg, 74% yield); R_f = 0.31 (Petroleum ether/EtOAc, 5:1); ^{1}H NMR (400 MHz, $CDCl_3$) δ 7.52 (d, J = 8.8 Hz, 1H), 7.37 (d, J = 7.4 Hz, 2H), 7.24 (d, J = 7.5 Hz, 2H), 7.00 (d, J = 7.6 Hz, 2H), 6.94 (d, J = 8.8 Hz, 1H), 6.90 (s, 1H), 6.56 (d, J = 7.7 Hz, 2H), 6.16 (s, 1H), 5.12 (s, 2H), 4.34 (t, J = 6.1 Hz, 1H), 4.14 (d, J = 6.9 Hz, 2H), 3.15 (t, J = 6.1 Hz, 2H), 2.42 (s, 3H), 2.25 (s, 3H), 1.19 (t, J = 7.0 Hz, 3H); ^{13}C NMR (151 MHz, $CDCl_3$) δ 173.2, 161.7, 161.3, 155.2, 152.6, 144.0, 136.8, 134.5, 129.9, 129.7, 128.0, 127.7, 125.6, 113.9, 113.8, 113.0, 112.1, 101.9, 70.2, 61.2, 58.1, 38.3, 20.4, 18.7, 14.2. HRMS (ESI, m/z) calcd for $C_{29}H_{30}NO_5$ $(M+H)^+$: 472.2119, found: 472.2115.

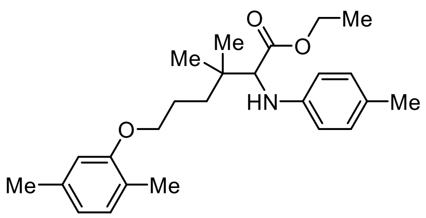

32: Yellow oil (34.2 mg, 86% yield); R_f = 0.81 (Petroleum ether/EtOAc, 5:1); ^{1}H NMR (400 MHz, $CDCl_3$) δ 7.02 (m, 3H), 6.67 (m, 4H), 4.17 (q, J = 7.1 Hz, 2H), 3.95 (t, J = 6.3 Hz, 2H), 3.89 (s, 1H), 2.34 (s, 3H), 2.27 (s, 3H), 2.20 (s, 3H), 1.89 (m, J = 5.9 Hz, 2H), 1.62 (m, J = 5.9 Hz, 2H), 1.26 (t, J = 7.1 Hz, 3H), 1.11 (d, J = 6.2 Hz, 6H); ^{13}C NMR (101 MHz, $CDCl_3$) δ 173.5, 157.0, 145.3, 136.5, 130.3, 129.8, 127.7, 123.7, 120.7, 114.2, 112.0, 68.3, 64.6, 60.6, 36.8, 36.1, 24.1, 24.0, 23.6, 21.4, 20.4, 15.8, 14.3. HRMS (ESI, m/z) calcd for $C_{25}H_{36}NO_3$ $(M+H)^+$: 398.2690, found: 398.2680.

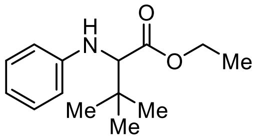

33: Yellow oil (15.9 mg, 68% yield); R_f = 0.73 (Petroleum ether/EtOAc, 5:1); ^{1}H NMR (400 MHz, $CDCl_3$) δ 7.19 (t, J = 7.5 Hz, 2H), 6.75 (t, J = 7.3 Hz, 1H), 6.69 (d, J = 7.9 Hz, 2H), 4.17 (q, J = 7.1 Hz, 3H), 3.81 (s, 1H), 1.26 (t, J = 7.2 Hz, 3H), 1.10 (s, 9H); ^{13}C NMR (101 MHz, $CDCl_3$) δ 173.4, 147.7, 129.3, 118.3, 113.8, 65.5, 60.6, 34.5, 26.8, 14.3. Spectra are consistent with reported literature[7] values.

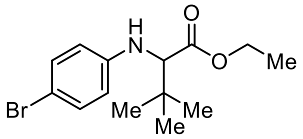

34: Yellow oil (16.6 mg, 53% yield); R_f = 0.64 (Petroleum ether/EtOAc, 5:1); ^{1}H NMR (400 MHz, $CDCl_3$) δ 7.26 (d, J = 7.9 Hz, 2H), 6.56 (d, J = 7.9 Hz, 2H), 4.21 (s, 1H), 4.17 (q, J = 7.0 Hz, 2 H), 3.74 (d, J = 10.0 Hz, 1H), 1.26 (t, J = 7.2 Hz, 3H), 1.08 (s, 9H); ^{13}C NMR (101 MHz, $CDCl_3$) δ 173.0, 152.8, 146.7, 132.0, 115.4, 109.9, 65.6, 60.8, 34.5, 26.7, 14.3. HRMS (ESI, m/z) calcd for $C_{14}H_{21}BrNO_2$ $(M+H)^+$: 314.0750, found: 314.0750.

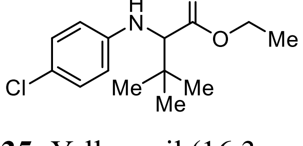

35: Yellow oil (16.3 mg, 61% yield); R_f = 0.69 (Petroleum ether/EtOAc, 5:1); ^{1}H NMR (400 MHz, $CDCl_3$) δ 7.13 (d, J = 8.0 Hz, 2H), 6.61 (d, J = 8.0 Hz, 2H), 4.17 (q, J = 6.9 Hz, 3H), 3.74 (d, J = 10.0 Hz, 1H), 1.26 (t, J = 7.5 Hz, 3H), 1.08 (s, 9H); ^{13}C NMR (101 MHz, $CDCl_3$) δ 173.1, 146.3, 129.1, 122.9, 115.0, 65.7, 60.7, 34.5, 26.8, 14.3. HRMS (ESI, m/z) calcd for $C_{14}H_{21}ClNO_2$ $(M+H)^+$: 270.1256, found: 270.1265.

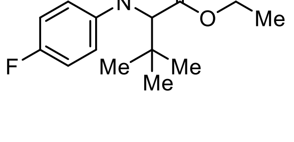

36: Yellow oil (27.2 mg, 54% yield); R_f = 0.72 (Petroleum ether/EtOAc, 5:1); ^{1}H NMR (400 MHz, $CDCl_3$) δ 6.89 (t, J = 8.5 Hz, 2H), 6.63 (dd, J = 4.41, 8.3 Hz, 2H), 4.17 (q, J = 4.3 Hz, 2H), 4.06 (d, J = 8.8 Hz, 1H), 3.70 (d, J = 9.1 Hz, 1H), 1.25 (t, J = 7.1 Hz, 3H), 1.09 (s, 9H); ^{13}C NMR (101 MHz, $CDCl_3$) δ 173.3, 156.3 (d, J = 235.9 Hz), 144.0 (d, J = 1.6 Hz), 115.7 (d, J = 22.4 Hz), 115.7 (d, J = 22.4 Hz), 115.1 (d, J = 7.4 Hz), 66.6, 60.6, 34.3, 26.8, 14.3. ^{19}F NMR (377 MHz, $CDCl_3$) δ -126.5. HRMS (ESI, m/z) calcd for $C_{14}H_{21}FNO_2$ $(M+H)^+$: 254.1551, found: 254.1553.

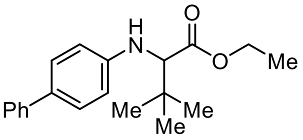

37: Yellow oil (31.0 mg, 50% yield); R_f = 0.58 (Petroleum ether/EtOAc, 5:1); ^{1}H NMR (400 MHz, $CDCl_3$) δ 7.58 (d, J = 7.6 Hz, 2H), 7.52–7.40 (m, 4H), 7.30 (t, J = 7.5 Hz, 1H), 6.79 (d, J = 8.0 Hz, 2H), 4.33 (d, J = 10.2 Hz, 1H), 4.22 (q, J = 7.1 Hz, 2H), 3.89 (d, J = 10.0 Hz, 1H), 1.30 (t, J = 7.1 Hz, 3H), 1.15 (s, 9H); ^{13}C NMR (101 MHz, $CDCl_3$) δ 173.3, 147.2, 141.2, 131.3, 128.7, 128.0, 126.4, 126.2, 114.1, 65.5, 60.7, 34.6, 26.8, 14.4. HRMS (ESI, m/z) calcd for $C_{20}H_{26}NO_2$ $(M+H)^+$: 312.1958, found: 312.1955.

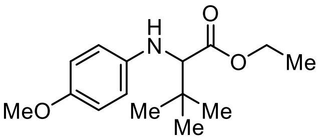

38: Yellow oil (38.6 mg, 73% yield); R_f = 0.63 (Petroleum ether/EtOAc, 5:1); ^{1}H NMR (400 MHz, $CDCl_3$) δ 6.78 (d, J = 8.8 Hz, 2H), 6.66 (d, J = 8.7 Hz, 2H), 4.20–4.10 (m, 2H), 3.92 (d, J = 4.3 Hz, 1H), 3.76 (s, 3H), 3.69 (s, 1H), 1.24 (t, J = 7.1 Hz, 3H), 1.09 (s, 9H); ^{13}C NMR (101 MHz, $CDCl_3$) δ 173.7, 152.8, 141.8, 115.6, 114.8, 67.1, 60.5, 55.7, 34.3, 26.8, 14.3. Spectra are consistent with reported literature[29] values.

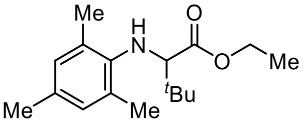

39: Yellow oil (16.3 mg, 55% yield); R_f = 0.78 (Petroleum ether/EtOAc, 5:1); ^{1}H NMR (400 MHz, $CDCl_3$) δ 6.77 (s, 2H), 4.00 (t, J = 6.7 Hz, 2H), 3.77 (s, 2H), 2.32 (s, 6H), 2.22 (s, 3H), 1.16 (s, 9H), 1.11 (t, J = 7.0 Hz, 3H); ^{13}C NMR (101 MHz, $CDCl_3$) δ 174.1, 141.4, 130.5, 129.7, 128.2, 67.6, 60.0, 34.4, 26.6, 20.5, 18.8, 14.1. HRMS (ESI, m/z) calcd for $C_{17}H_{28}NO_2$ $(M+H)^+$: 278.2115, found: 278.2108.

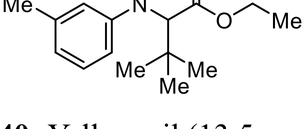

40: Yellow oil (13.5 mg, 27% yield); R_f = 0.68 (Petroleum ether/EtOAc, 5:1); ^{1}H NMR (400 MHz, $CDCl_3$) δ 7.08 (t, J = 7.6 Hz, 1H), 6.58 (d, J = 7.4 Hz, 1H), 6.54–6.46 (m, 2H), 4.18 (q, J = 7.1 Hz, 2H), 4.15 (s, 1H), 3.81 (d, J = 8.6 Hz, 1H), 2.29 (s, 3H), 1.27 (t, J = 7.2 Hz, 3H), 1.09 (s, 9H); ^{13}C NMR (101 MHz, $CDCl_3$) δ 173.4, 147.7, 139.1, 129.2, 119.2, 114.7, 110.9, 65.5, 60.6, 34.5, 26.8, 21.6, 14.3. HRMS (ESI, m/z) calcd

for $C_{15}H_{24}NO_2$ $(M+H)^+$: 250.1802, found: 250.1798.

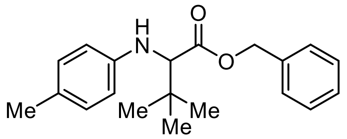

41: Yellow oil (14.3 mg, 46% yield); R_f = 0.69 (Petroleum ether/EtOAc, 5:1); ^{1}H NMR (400 MHz, $CDCl_3$) δ 7.40–7.29 (m, 5H), 7.19 (d, J = 8.0 Hz, 2H), 6.63 (d, J = 8.0 Hz, 2H), 5.51 (s, 2H), 4.10 (d, J = 7.5 Hz, 1H),3.86 (d, J = 8.0 Hz, 1H),2.28 (s, 3H), 1.10 (s, 9H); ^{13}C NMR (101 MHz, $CDCl_3$) δ 173.5, 145.3, 135.6, 129.8, 128.5, 128.4, 128.3, 127.7, 114.2, 66.5, 66.1, 34.5, 26.8, 20.4. HRMS (ESI, m/z) calcd for $C_{20}H_{26}NO_2$ $(M+H)^+$: 312.1958, found: 312.1956.

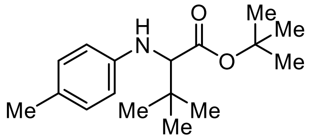

42: Yellow oil (30.6 mg, 55% yield); R_f = 0.66 (Petroleum ether/EtOAc, 5:1); ^{1}H NMR (400 MHz, $CDCl_3$) δ 7.00 (d, J = 7.8 Hz, 2H), 6.61 (d, J = 7.8 Hz, 2H), 4.06 (s, 1H), 3.65 (s, 1H), 2.26 (s, 3H), 1.49 (s, 9H), 1.09 (s, 9H); ^{13}C NMR (101 MHz, $CDCl_3$) δ 172.7, 145.7, 129.7, 127.3, 114.1, 81.3, 66.5, 34.4, 28.1, 26.9, 20.4. HRMS (ESI, m/z) calcd for $C_{17}H_{28}NO_2$ $(M+H)^+$: 278.2115, found: 278.2112.

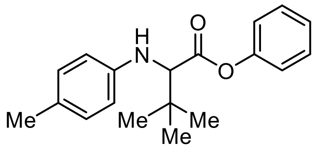

43: Yellow oil (11.3 mg, 38% yield); R_f = 0.52 (Petroleum ether/EtOAc, 5:1); ^{1}H NMR (400 MHz, $CDCl_3$) δ 7.37 (t, J = 7.6 Hz, 2H), 7.23 (t, J = 7.4 Hz, 1H), 7.05 (d, J = 7.8 Hz, 2H), 6.99 (d, J = 7.7 Hz, 2H), 6.72 (d, J = 7.9 Hz, 2H), 4.15 (s, 1H), 4.03 (s, 1H), 2.29 (s, 3H), 1.22 (s, 9H); ^{13}C NMR (101 MHz, $CDCl_3$) δ 172.3, 150.5, 145.1, 129.9, 129.4, 128.0, 125.9, 121.5, 114.2, 66.1, 34.6, 26.9, 20.4.MHz, $CDCl_3$) HRMS (ESI, m/z) calcd for $C_{19}H_{23}NO_2$ $(M+H)^+$: 298.1802, found: 298.1799.

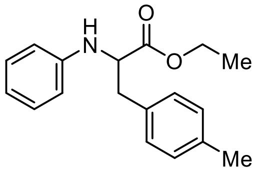

44: Yellow oil (16.4 mg, 58% yield); R_f = 0.61 (Petroleum ether/EtOAc, 5:1); ^{1}H NMR (400 MHz, $CDCl_3$) δ 7.21 (t, J = 7.5 Hz, 2H), 7.12 (q, J = 7.5 Hz, 4H), 6.78 (t, J = 7.2 Hz, 1H), 6.65 (d, J = 7.8 Hz, 2H), 4.37 (t, J = 6.1 Hz, 1H), 4.17 (q, J = 6.8 Hz, 2H), 3.14 (t, J = 6.7 Hz, 2H), 2.36 (s, 3H), 1.22 (t, J = 7.1 Hz, 3H); ^{13}C NMR (101 MHz, $CDCl_3$) δ 173.2, 146.4, 136.6, 133.2, 129.4, 129.2 (2C), 118.4, 113.7, 61.1, 57.8, 38.2, 21.1, 14.2. HRMS (ESI, m/z) calcd for $C_{18}H_{22}NO_2$ $(M+H)^+$: 284.1645, found: 284.1645.

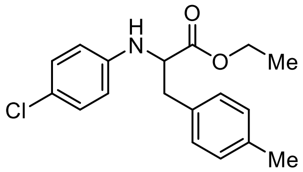

45: Yellow oil (18.1 mg, 57% yield); R_f = 0.67 (Petroleum ether/EtOAc, 5:1); ^{1}H NMR (600 MHz, $CDCl_3$) δ 7.17–7.10 (m, 4H), 7.07 (d, J = 8.0 Hz, 2H), 6.55 (d, J = 8.9 Hz, 2H), 4.30 (t, J = 6.2 Hz, 1H), 4.17 (m, J = 3.6 Hz, 2H), 3.11 (m, J = 7.5 Hz, 2H), 2.35 (s, 3H), 1.22 (t, J = 7.1 Hz, 3H); ^{13}C NMR (101 MHz, $CDCl_3$) δ 173.0, 145.1, 136.7, 132.9, 129.3, 129.2 (2C), 122.9, 114.7, 61.3, 57.8, 38.0, 21.1, 14.2. HRMS (ESI, m/z) calcd for $C_{18}H_{21}ClNO_2$ $(M+H)^+$: 318.1256, found: 318.1247.

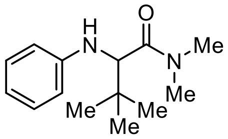

46: Yellow oil (20.4 mg, 44% yield); R_f = 0.19 (Petroleum ether/EtOAc, 5:1); ^{1}H NMR (400 MHz, $CDCl_3$) δ 7.17 (t, J = 7.6 Hz, 2H), 6.75–6.65 (m, 3H), 4.54 (s, 1H), 4.22 (s, 1H), 3.13 (s, 3H), 2.95 (s, 3H), 1.09 (s, 9H); ^{13}C NMR (101 MHz, $CDCl_3$) δ 173.1, 148.5, 129.3, 117.9, 114.0, 60.1, 38.2, 36.1, 35.6, 26.8. HRMS (ESI, m/z) calcd for $C_{14}H_{23}N_2O$ $(M+H)^+$: 235.1805, found: 235.1805.

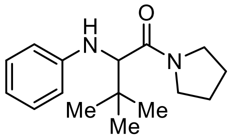

47: Yellow oil (13.8 mg, 53% yield); R_f = 0.20 (Petroleum ether/EtOAc, 5:1); ^{1}H NMR (400 MHz, $CDCl_3$) δ 7.16 (t, J = 7.7 Hz, 2H), 6.70 (m, J = 6.5 Hz, 3H), 4.54 (s, 1H), 4.00 (s, 1H), 3.52 (m, J = 6.0 Hz, 1H), 1.94 (m, 2H), 1.85 (t, 2H), 1.10 (s, 9H); ^{13}C NMR (101 MHz, $CDCl_3$) δ 171.4, 148.1, 129.3, 118.0, 114.1, 63.0, 47.5, 45.7, 36.0, 26.8, 26.1, 24.1. HRMS (ESI, m/z) calcd for $C_{16}H_{25}N_2O$ $(M+H)^+$: 261.1962, found: 261.1960.

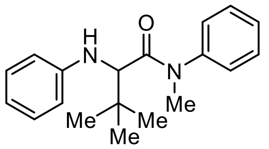

48: Yellow oil (16.3 mg, 55% yield); R_f = 0.46 (Petroleum ether/EtOAc, 5:1); ^{1}H NMR (400 MHz, $CDCl_3$) δ 7.36 (s, 2H), 7.12 (t, J = 7.08 Hz, 2H), 6.96 (s, 2H), 6.71 (t, J = 6.98 Hz, 1H), 6.44 (d, J = 7.56 Hz, 2H), 3.26 (s, 3H), 0.95 (s, 9H); ^{13}C NMR (101 MHz, $CDCl_3$) δ 172.9, 146.4, 143.3, 129.6, 129.2 (2C), 127.9, 118.9, 115.3, 62.5, 37.6, 36.3, 26.8. HRMS (ESI, m/z) calcd for $C_{19}H_{25}N_2O$ $(M+H)^+$: 297.1962, found: 297.1959.

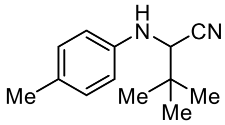

49: Yellow oil (17.8 mg, 88% yield); R_f = 0.76 (Petroleum ether/EtOAc, 5:1); ^{1}H NMR

(600 MHz, $CDCl_3$) δ 7.09 (d, *J* = 8.1 Hz, 2H), 6.70 (d, *J* = 8.3 Hz, 2H), 3.92 (s, 1H), 2.30 (s, 3H), 1.21 (s, 9H); ^{13}C NMR (101 MHz, $CDCl_3$) δ 143.2, 130.0, 129.5, 119.0, 114.6, 57.3, 34.7, 26.2, 20.5. HRMS (ESI, m/z) calcd for $C_{13}H_{19}N_2$ $(M+H)^+$: 203.1543, found: 203.1541.

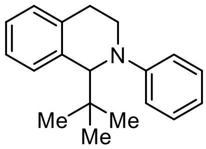

50: Yellow oil (13.5 mg, 51% yield); R_f = 0.85 (Petroleum ether/EtOAc, 5:1). Spectra are consistent with reported literature[2] values.

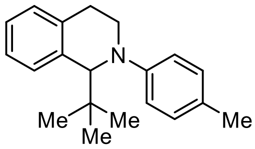

51: Yellow oil (15.6 mg, 56% yield); R_f = 0.83 (Petroleum ether/EtOAc, 5:1). Spectra are consistent with reported literature[2] values.

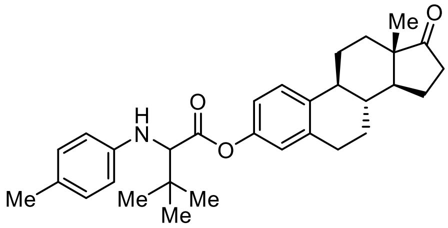

52: Yellow oil (6.1 mg, 13% yield); R_f = 0.36 (Petroleum ether/EtOAc, 5:1); ^{1}H NMR (400 MHz, $CDCl_3$) δ 7.26 (d, *J* = 8.7 Hz, 1H), 7.04 (d, *J* = 7.6 Hz, 2H), 6.76 (d, *J* = 8.4 Hz, 1H), 6.70 (d, *J* = 8.1 Hz, 3H), 4.01 (s, 1H), 2.89 (t, *J* = 3.6 Hz, 2H), 2.52 (q, *J* = 9.1 Hz, 1H), 2.45–2.36 (m, 1H), 2.28 (s, 3H), 2.19–1.93 (m, 2H), 1.70–1.41 (m, 10H), 1.20 (s, 9H), 0.92 (s, 3H); ^{13}C NMR (151 MHz, $CDCl_3$) δ 220.9, 172.5, 148.3, 145.1, 138.0, 137.5, 129.9, 126.4, 121.4, 118.6 (2C), 114.3, 66.2, 50.4, 48.0, 44.1, 38.0, 35.9, 34.7, 31.5, 29.4, 26.9, 26.3, 25.7, 21.6, 20.4, 13.8. HRMS (ESI, m/z) calcd for $C_{31}H_{40}NO_3$ $(M+H)^+$: 474.2930, found: 474.2996.

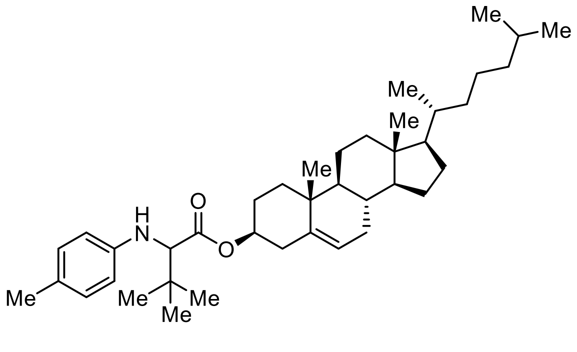

53: Pale solid (50.8 mg, 86% yield); m.p. 161–162 °C; R_f = 0.83 (Petroleum ether/EtOAc, 5:1); ^{1}H NMR (400 MHz, $CDCl_3$) δ 7.05–6.95 (m, 2H), 6.68–6.58 (m, 2H), 5.37 (s, 1H), 4.80–4.85 (m, 2H), 4.08 (s, 0.5H), 3.74 (s, 0.5H), 2.26 (s, 1H), 2.45–2.19 (m, 5H), 2.13–1.76 (m, 6H), 1.23–1.12 (m, 6H), 1.10 (s, 7H), 1.07–0.98 (m, 7H), 0.96–0.87 (m, 14H), 0.71 (s, 3H); ^{13}C NMR (101 MHz, $CDCl_3$) δ 172.8, 145.5 (2C),

139.5 (2C), 129.7, 127.5, 122.8, 114.1 (2C), 74.4, 66.0 (2C), 56.7, 56.2, 50.0, 42.3, 39.7, 39.5, 38.2, 37.0, 36.9, 36.6, 36.2, 35.8, 34.4, 31.9 (2C), 28.2, 28.0, 26.9, 24.3, 23.9, 22.8, 22.6, 21.0, 20.4, 19.3, 18.7, 14.2, 11.9. HRMS (ESI, m/z) calcd for $C_{40}H_{64}NO_2$ $(M+H)^+$: 590.4932, found: 590.4920.

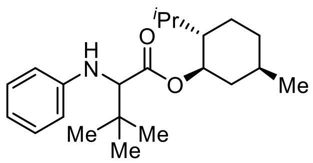

54: Yellow oil (12.8 mg, 37% yield); R_f = 0.85 (Petroleum ether/EtOAc, 5:1); ^1H NMR (400 MHz, $CDCl_3$) δ 7.16 (t, J = 7.7 Hz, 2H), 6.71 (m, J = 7.3 Hz, 3H), 4.66 (m, J = 5.4 Hz, 1H), 3.79 (s, 1H), 1.98–1.88 (m, 1H), 1.75–1.57 (m, 3H), 1.11 (s, 9H), 0.97–0.70 (m, 10H), 0.54 (q, J = 10.3 Hz, 3H); ^{13}C NMR (101 MHz, $CDCl_3$) δ 173.2, 172.8, 147.7, 147.6, 129.2, 129.1, 118.6, 118.3, 114.5, 113.7, 75.1, 75.0, 66.1, 66.0, 46.9, 46.8, 40.9, 40.8, 34.3, 34.2, 33.9, 31.4, 26.9, 26.8, 25.9, 25.3, 22.9, 22.7, 22.0, 20.8, 15.7, 15.3. HRMS (ESI, m/z) calcd for $C_{22}H_{36}NO_2$ $(M+H)^+$: 346.2741, found: 346.2738.

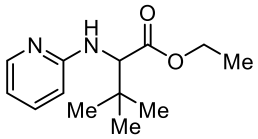

55: Yellow oil (7.1 mg, 30% yield); R_f = 0.48 (Petroleum ether/EtOAc, 3:1); ^1H NMR (400 MHz, $CDCl_3$) δ 8.04 (d, J = 5.0 Hz, 1H), 7.50 (t, J = 7.7 Hz, 1H), 6.64 (t, J = 6.1 Hz, 1H), 6.52 (d, J = 8.4 Hz, 1H), 5.75 (s, 1H), 4.29 (d, J = 9.2 Hz, 1H), 4.20 (q, J = 7.0, Hz, 2H), 1.28 (t, J = 6.6 Hz, 3H), 1.11 (s, 9H); ^{13}C NMR (101 MHz, $CDCl_3$) δ 172.4, 157.3, 138.9, 113.3, 108.5, 63.1, 60.9, 34.5, 26.8, 14.3. HRMS (ESI, m/z) calcd for $C_{13}H_{21}N_2O_2$ $(M+H)^+$: 237.1598, found: 237.1591.

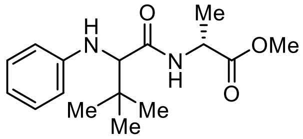

56: Pale solid (40.3 mg, 69% yield); m.p. 93–94 °C; R_f = 0.35 (Petroleum ether/EtOAc, 3:1); ^1H NMR (400 MHz, $CDCl_3$) δ 7.21 (t, J = 7.6 Hz, 1H), 7.02 (d, J = 6.2 Hz, 1H), 6.81 (t, J = 7.3 Hz, 1H), 6.65 (t, J = 9.6 Hz, 2H), 4.61 (m, J = 6.8 Hz, 1H), 4.08 (s, 1H), 3.74 (s, 1H), 3.66 (s, 2H), 3.43 (s, 1H), 1.37 (t, J = 14.2 Hz, 2H), 1.30 (t, J = 7.4 Hz, 2H), 1.14 (d, J = 6.7 Hz, 9H); ^{13}C NMR (101 MHz, $CDCl_3$) δ 173.0, 171.7, 147.4, 129.3, 129.3, 119.0, 114.0, 113.7, 68.4, 52.3, 47.9, 34.3, 27.2, 18.4. HRMS (ESI, m/z) calcd for $C_{16}H_{25}N_2O_3$ $(M+H)^+$: 293.1860, found: 293.1860.

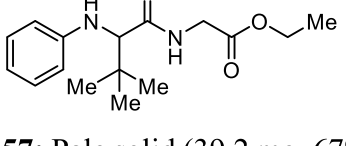

57: Pale solid (39.2 mg, 67% yield); m.p. 92–94 °C; R_f = 0.32 (Petroleum ether/EtOAc, 3:1); ^1H NMR (400 MHz, $CDCl_3$) δ 7.21 (t, J = 7.7 Hz, 2H), 7.03 (s,1 H), 6.81 (t, J = 7.3 Hz, 1H), 6.66 (d, J = 7.9 Hz, 2H), 4.21 (t, J = 3.1 Hz, 1H), 4.18 (t, J = 7.1 Hz, 2H), 4.10 (s, 1H), 3.89 (d, J = 4.7 Hz, 1H), 3.84 (d, J = 4.7 Hz, 1H), 3.47 (d, J = 2.2 Hz, 1H),

1.26 (t, J = 7.1 Hz, 3H), 1.16 (s, 1H); ^{13}C NMR (101 MHz, CDCl$_3$) δ 172.4, 169.8, 147.3, 129.4, 119.0, 113.8, 68.4, 61.4, 41.1, 34.3, 27.2, 14.1. HRMS (ESI, m/z) calcd for $C_{16}H_{25}N_2O_3$ $(M+H)^+$: 293.1860, found: 293.1860.

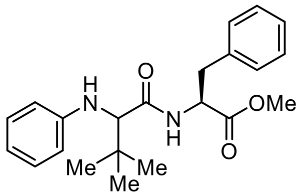

58: Pale solid (39.9 mg, 54% yield); m.p. 129–130 °C; R_f = 0.33 (Petroleum ether/EtOAc, 3:1); ^{1}H NMR (400 MHz, CDCl$_3$) δ 7.22 (q, J = 8.3 Hz, 6H), 7.08 (m, J = 10.3 Hz, 5H), 6.82 (m, J = 6.5 Hz, 3H), 6.73 (d, J = 7.5 Hz, 2H), 6.65 (d, J = 7.8 Hz, 2H), 6.61 (d, J = 7.9 Hz, 2H), 5.00 (m, J = 5.0 Hz, 1H), 4.90 (q, J = 6.9 Hz, 1H), 4.02 (s, 2H), 3.72 (s, 1H), 3.62 (s, 3H), 3.44 (s, 1H), 3.38 (s, 1H), 3.20 (d, J = 5.4 Hz, 0.5H), 3.16 (d, J = 5.4 Hz, 0.5H), 3.10 (d, J = 5.9 Hz, 0.5H), 3.06 (d, J = 5.8 Hz, 0.5H), 3.02 (d, J = 7.5 Hz, 0.5H), 2.98 (d, J = 7.5 Hz, 0.5H), 2.92 (d, J = 5.2 Hz, 0.5H), 2.89 (d, J = 5.2 Hz, 0.5H), 1.10 (s, 9H), 0.99 (s, 9H); ^{13}C NMR (101 MHz, CDCl$_3$) δ 172.2, 172.0, 171.8, 171.6, 147.6, 147.2, 136.1, 135.5, 129.5, 129.3 (3C), 128.7, 128.6, 127.1, 127.0, 119.1 (2C), 114.4, 113.8, 113.7, 68.8, 68.3, 53.6, 53.1, 52.3, 38.1, 38.0, 34.2 (2C), 27.3, 27.1. HRMS (ESI, m/z) calcd for $C_{22}H_{29}N_2O_3$ $(M+H)^+$: 369.2173, found: 369.2171.

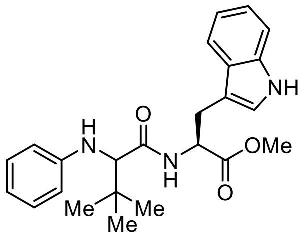

59: Pale solid (41.4 mg, 51% yield); m.p. 139–140 °C; R_f = 0.30 (Petroleum ether/EtOAc, 3:1); ^{1}H NMR (400 MHz, CDCl$_3$) δ 8.15 (s, 1H), 7.88 (d, J = 2.3 Hz, 2H), 7.77 (t, J = 2.8 Hz, 1H), 7.50 (d, J = 7.9 Hz, 1H), 7.40 (d, J = 7.9 Hz, 1H), 7.33 (d, J = 8.1 Hz, 1H), 7.20 (m, J = 8.1 Hz, 8H), 7.07 (q, J = 6.6 Hz, 2H), 6.83 (m, J = 8.5 Hz, 4H), 6.63 (t, J = 9.8 Hz, 4H), 6.23 (s, 1H), 4.95 (m, J = 7.3 Hz, 2H), 4.06 (s, 2H), 3.67 (s, 3H), 3.62 (s, 3H), 3.47 (s, 1H), 3.43 (s, 1H), 3.30 (m, J = 6.4 Hz, 3H), 3.10 (q, J = 6.5 Hz, 1H), 1.10 (s, 9H), 0.99 (s, 9H); ^{13}C NMR (101 MHz, CDCl$_3$) δ 172.4, 172.2 (2C), 171.8, 168.3, 147.7, 147.2, 136.1, 136.0, 134.3, 132.7, 129.4, 129.2, 127.5, 127.2, 123.5, 123.3, 122.9, 122.1, 122.0, 119.6, 119.5, 118.8, 118.7, 118.5, 118.4, 114.3, 113.6, 111.3, 111.2, 109.9, 109.2, 68.2 (2C), 52.7, 52.3, 52.2, 51.8, 34.4, 34.1, 27.6, 27.6, 27.2, 27.0. HRMS (ESI, m/z) calcd for $C_{24}H_{30}N_3O_3$ $(M+H)^+$: 408.2282, found: 408.2278.

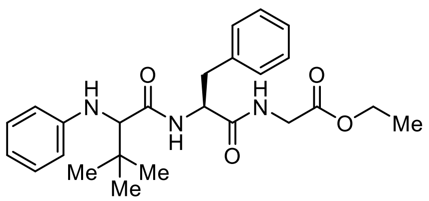

60: Pale solid (50.8 mg, 86% yield); R_f = 0.30 (Petroleum ether/EtOAc, 3:1); m.p. 145–147 °C; ^{1}H NMR (400 MHz, CDCl$_3$) δ 7.25–7.09 (m, 5H), 6.98–6.79 (m, 2H), 6.67–6.52 (m, 2H), 6.43–6.24 (m, 1H), 4.92–4.68 (m, 1H), 4.27–4.10 (m, 2H), 3.98–3.85 (m,

1.5H), 3.72–3.59 (m, 0.5H), 3.49–3.36 (m, 1H), 3.31–2.87 (m, 1.5H), 1.28 (s, 3H), 1.13–0.83 (m, H); ^{13}C NMR (101 MHz, $CDCl_3$) δ 172.6, 172.5, 171.0, 170.9, 169.3 (2C), 147.1, 146.9, 136.7, 136.0, 129.5, 129.4, 129.2 (2C), 128.7, 128.6, 127.0, 126.9, 119.1 (2C), 113.8, 113.6, 68.3 (2C), 61.6, 61.4, 53.8, 53.6, 41.4, 41.1, 37.6, 37.3, 34.2, 33.6, 27.2, 27.0, 14.2, 14.1. HRMS (ESI, m/z) calcd for $C_{25}H_{34}N_3O_4$ $(M+H)^+$: 440.2544, found: 440.2531.

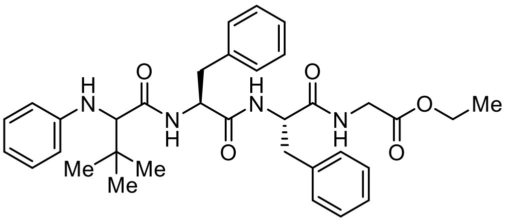

61: Pale solid (50.8 mg, 86% yield); R_f = 0.31 (Petroleum ether/EtOAc, 3:1); m.p. 159–160 °C; ^{1}H NMR (400 MHz, $CDCl_3$) δ 7.27–7.18 (m, 6H), 7.16–7.12 (m, 2H), 7.06–6.99 (m, 2H), 6.95–6.92 (m, 2H), 6.86–6.81 (m, 1H), 6.75–6.69 (m, 1H), 6.58 (d, J = 7.9 Hz, 1H), 6.32 (t, J = 5.1 Hz, 1H), 6.24 (d, J = 7.8 Hz, 1H), A4.72–4.56 (m, 2H), 4.24–4.18 (m, 2H), 4.04–3.96 (m, 1H), 3.89–3.80 (m, 1H), 3.45–3.33 (m, 1H), 3.05–2.98 (m, 2H), 2.97–2.81 (m, 2H), 1.29 (t, J = 7.1 Hz, 3H), 0.99 (s, 7H), 0.82 (s, 2H); ^{13}C NMR (101 MHz, $CDCl_3$) δ 172.9, 170.5, 170.4, 169.4, 136.3, 135.7, 130.3, 129.5, 129.3, 129.2, 129.0, 128.8, 128.7, 128.6, 128.5, 127.1, 127.0, 119.8, 113.9, 113.3, 68.2, 61.5, 54.5, 54.0, 41.3, 37.6, 37.5, 34.1, 27.2, 26.8, 14.2 (2C). HRMS (ESI, m/z) calcd for $C_{34}H_{43}N_4O_5$ $(M+H)^+$: 587.3228, found: 587.3217.

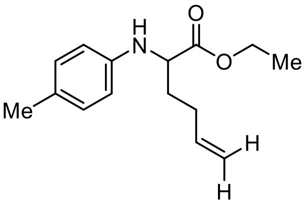

62: Yellow oil (9.3 mg, 19% yield); R_f = 0.77 (Petroleum ether/EtOAc, 5:1); ^{1}H NMR (400 MHz, $CDCl_3$) δ 7.00 (d, J = 8.1 Hz, 2H), 6.57 (d, J = 8.2 Hz, 2H), 5.91–5.78 (m, 1H), 5.08 (d, J = 17.4 Hz, 1H), 5.04 (d, J = 10.9 Hz, 1H), 4.19 (q, J = 7.11 Hz, 1H), 4.03 (br s, 1H), 2.25 (s, 3H), 2.00–1.89 (m, 1H), 1.89–1.79 (m, 1H), 1.27 (t, J = 7.0 Hz, 3H); ^{13}C NMR (101 MHz, $CDCl_3$) δ 174.3, 144.6, 137.3, 129.8, 127.6, 115.7, 113.8, 61.0, 56.5, 32.3, 29.7, 20.4. HRMS (ESI, m/z) calcd for $C_{15}H_{22}NO_2$ $(M+H)^+$: 248.1645, found: 248.1645.

3. Supplementary Notes

3.1 Reaction Profile General

Time profile of the transformation with the light ON/OFF over time.

As can be seen in the time-course plot, the reaction proceeds rapidly within the first hour under the standard conditions and The yield of **15** continued to increase slowly over the next 4 hours (**Supplementary Figure 2**, a). From the profile of the reaction with the light ON/OFF over time, it was observed that the transformation progressed

smoothly under light, but no further conversion was observed when the light is turned off, which suggested that the transformation proceeds through a photoredox catalytic pathway rather than radical chain propagation (**Supplementary Figure 2**, b).

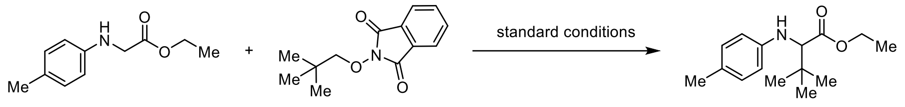

1a **2b** **15**

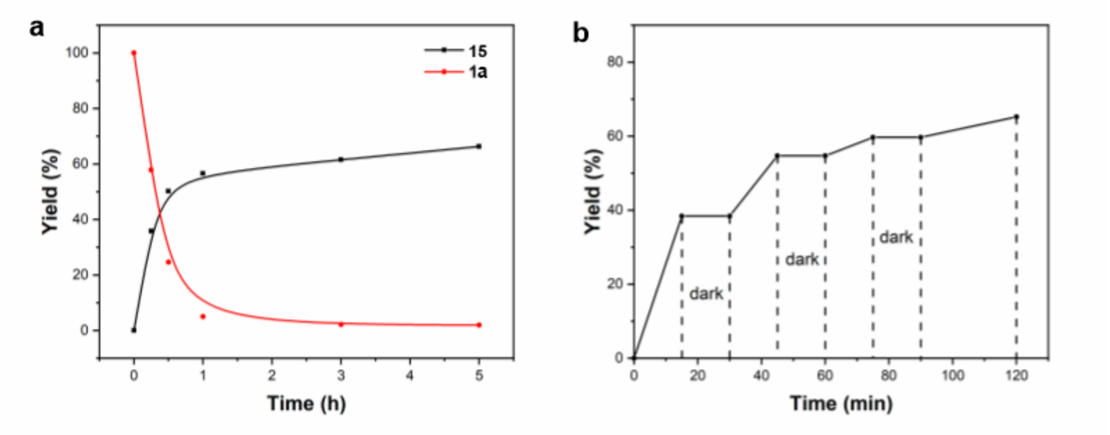

Standard conditions: 5 mol% 4CzIPN, 0.1 mmol **1a**, 2.0 equiv. **2b**, DMSO (0.03 M), 15 W Blue LEDs, 60 °C, 5 h

Supplementary Figure 2. Reaction profile. a Time course of the yield of **15** and consumption of **1a** during a reaction under the standard conditions. b Time course of the yield of **15** for during which the light was periodically turned on and off

3.2 Examining Functional Group Compatibility

In order to further prove the possibility of this reaction for biological application. We switched DMSO to a mixture H_2O/DMSO (1 : 4) solvent system and lowered the temperature to 37 °C. A broad of biomolecules were selected including nucleobase, amino acid, saccharide, biotin, protein and DNA. As shown in the following figure, all reactions worked and gave desired product in the presence of these biomolecules (entries 1–9).

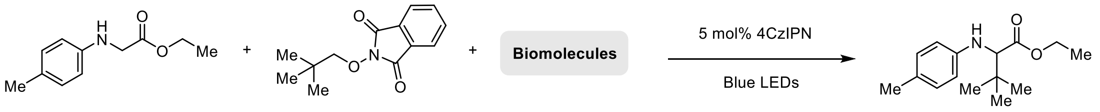

1a **2b** **15**

Entry	Biomolecules	Yield[a]
1	none	63%
2	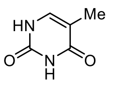 Thymine (1 equiv.)	50%
3	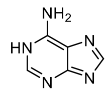 Adenine (1 equiv.)	77%
4	10 mg ml^{-1} bovine serum albumin	32%
5	0.01 mg ml^{-1} pCMV-EGFP	72%
6	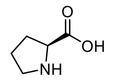 L-Proline (1 equiv.)	29%
7	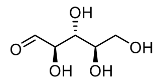 D-(−)-Ribose (1 equiv.)	86%
8	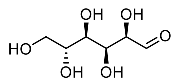 D(+)-Glucose (1 equiv.)	68%
9	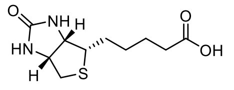 D-Biotin (1 equiv.)	60%

Supplementary Figure 3. Examining functional group compatibility

[a] Yields were determined by HPLC analysis.

Experiment procedure: The glycine **1a** (0.2 mmol, 38.7 mg), *N*-alkoxyphthalimides **2b** (0.1 mmol, 23.3 mg), 4CzIPN (5 mol%, 0.005 mmol, 4.0 mg), and DMSO (2.4 mL), H_2O (0.6 mL), were added sequentially to a 4 mL clear-colored glass vial equipped with a magnetic stir bar. After bubbled with nitrogen gas for 5 minutes to remove oxygen, the vial was sealed and exposed to blue LEDs at 37 °C. The reaction mixture was monitored by TLC until the starting material *N*-alkoxyphthalimides were consumed. After the reaction was completed, 100 μL reaction mixture was diluted 100 times by sequential dilution and analyzed by HPLC with 30 μL.

3.3 In Vitro Antifungal Activities.

Each target compound was dissolved in DMSO to prepare the stock solution (10.0 g/L). The stock solution was added into the PDA medium, and the concentration of target compounds in the medium was 50.0 mg/L. Pure DMSO without the target compounds was utilized as the blank control, and boscalid was coassayed as the reference compound. Fresh dishes with a diameter of 5 mm were taken from the edge of the PDA-cultured fungi colonies and inoculated on the above three PDA media. Each treatment was tested for three replicates, and the antifungal effect was averaged. The relative

inhibitory rate I (%) of all the tested compounds was calculated through the equation: I (%) = [(C − T)/(C − 5)] × 100. In this equation, I is the inhibitory rate and C and T are the colony diameter of the blank control (mm) and treatment (mm), respectively.

Mycelia growth of six crop pathogenic fungi after treating with the target compounds on PDA medium as illustrated in the figures (**Supplementary Figure 4. PDA medium**) below.

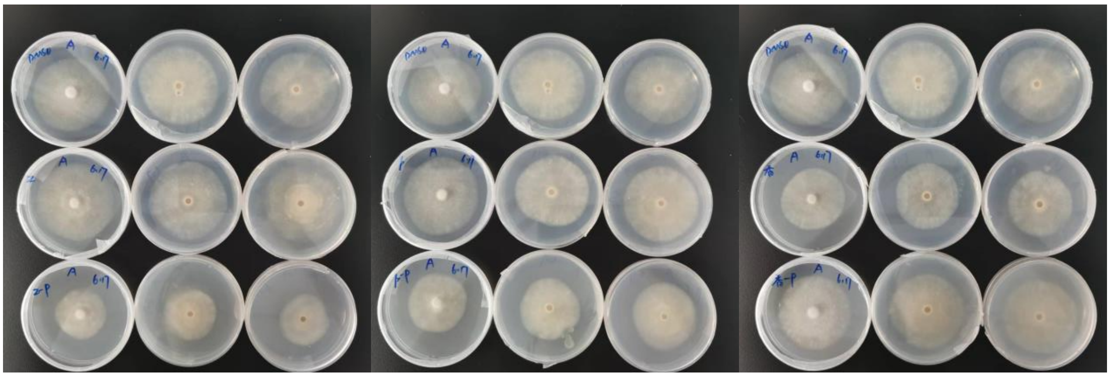

Supplementary Figure 4-1. PDA medium. *B. cinerea*

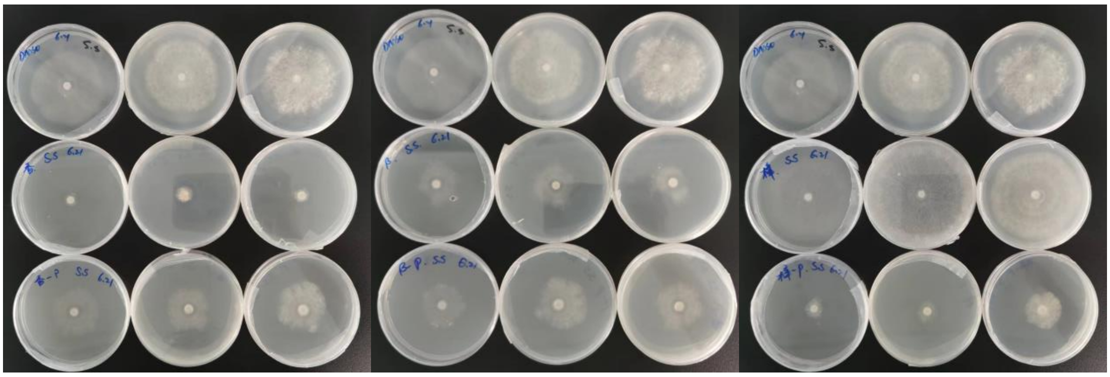

Supplementary Figure 4-2. PDA medium. *S. sclerotiorum*

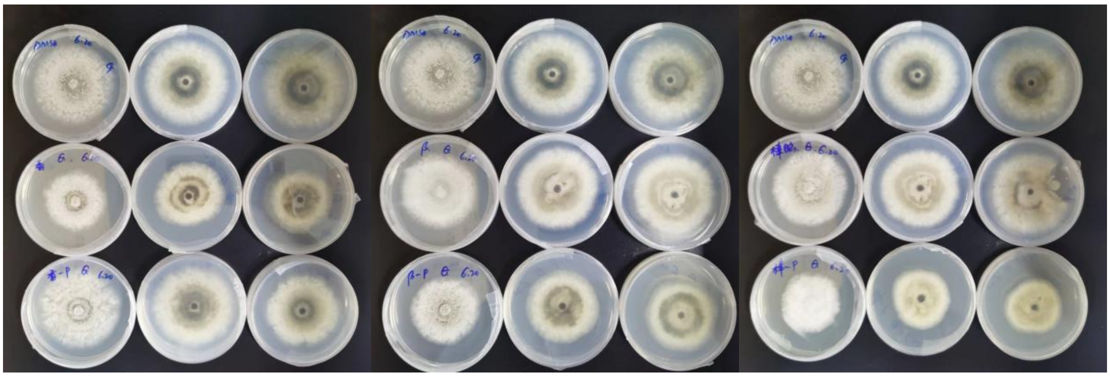

Supplementary Figure 4-3. PDA medium. *C. orbiculare*

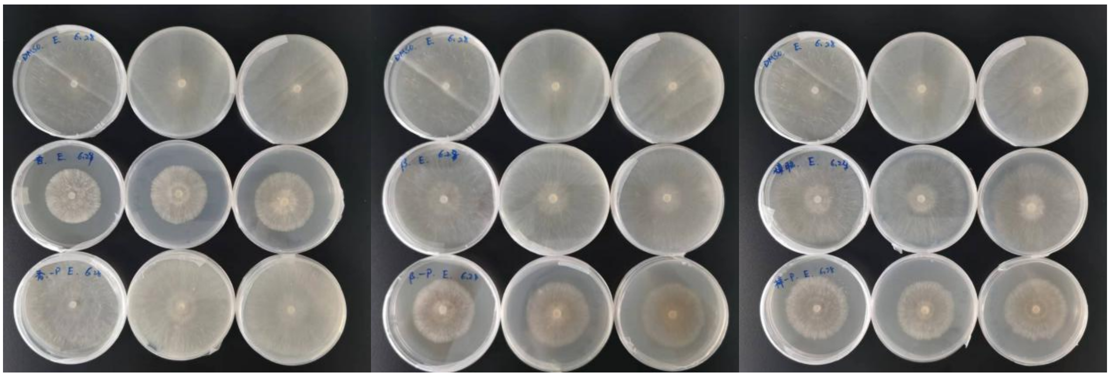

Supplementary Figure 4-4. PDA medium. *T. cucumeris*

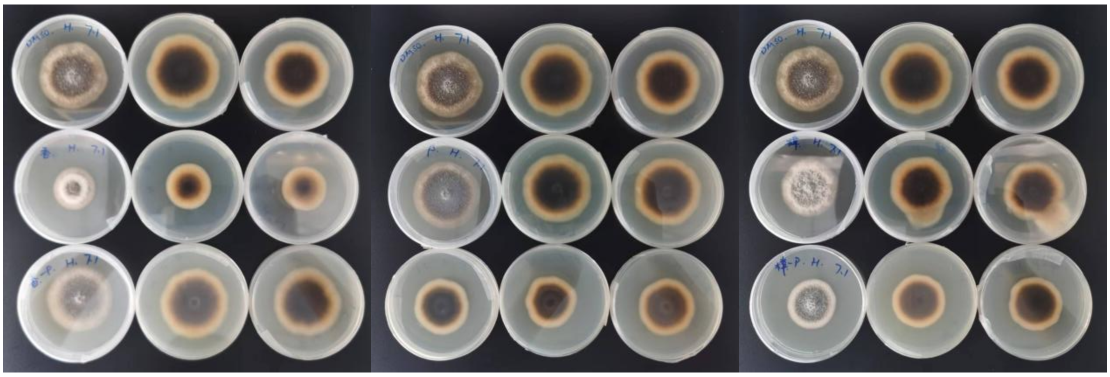

Supplementary Figure 4-5. PDA medium. *Alternariamali*

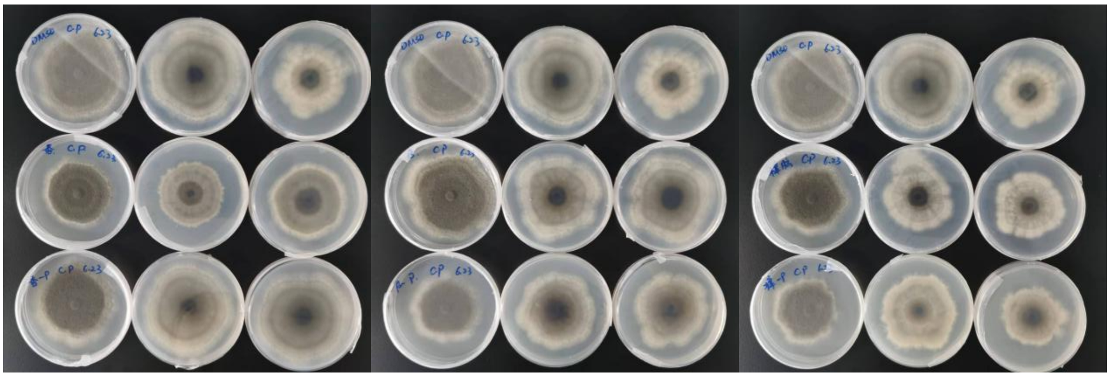

Supplementary Figure 4-6. PDA medium. *C. paradoxa*

Supplementary Table 1. In vitro antifungal activities.

In Vitro Antifungal Activities of the target compound 54 at 50.0 mg/L

Compd	Inhibition rate (%)[a]					
	B. cinerea	*S. sclerotiorum*	*C. orbiculare*	*T. cucumeris*	*Alternariamali*	*C. paradoxa*
boscalid	36.8 ± 5.4	90.0 ± 0.7	27.7 ± 3.3	91.0 ± 1.7	89.7 ± 2.5	29.0 ± 0
**54**	-55.2 ± 6.2	8.3 ± 7.5	-0.6 ± 2.8	23.7 ± 1.6	-2.9± 2.7	-0.9 ± 2.1

[a] Values are the mean ± standard deviation of three replicates.

Mycelia growth of six crop pathogenic fungi after treating with the target compound **54** on PDA medium as illustrated in the figures (**Supplementary Figure 5. PDA medium**) below.

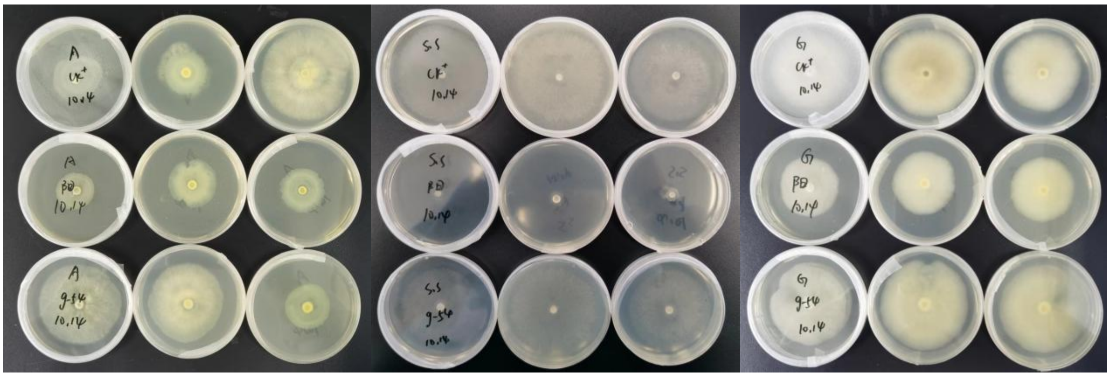

(a) (b) (c)

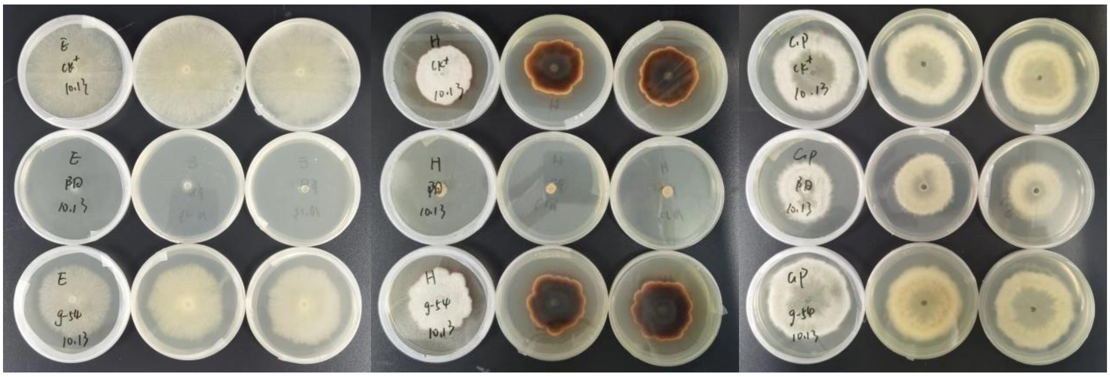

(d) (e) (f)

Supplementary Figure 5. PDA medium. (a) *B. cinerea*; (b) *S. sclerotiorum*; (c) *C. orbiculare*; (d) *T. cucumeris*; (e) *Alternariamali*; (e) *C. paradoxa*.

4. Supplementary Figures

4.1 NMR Spectra

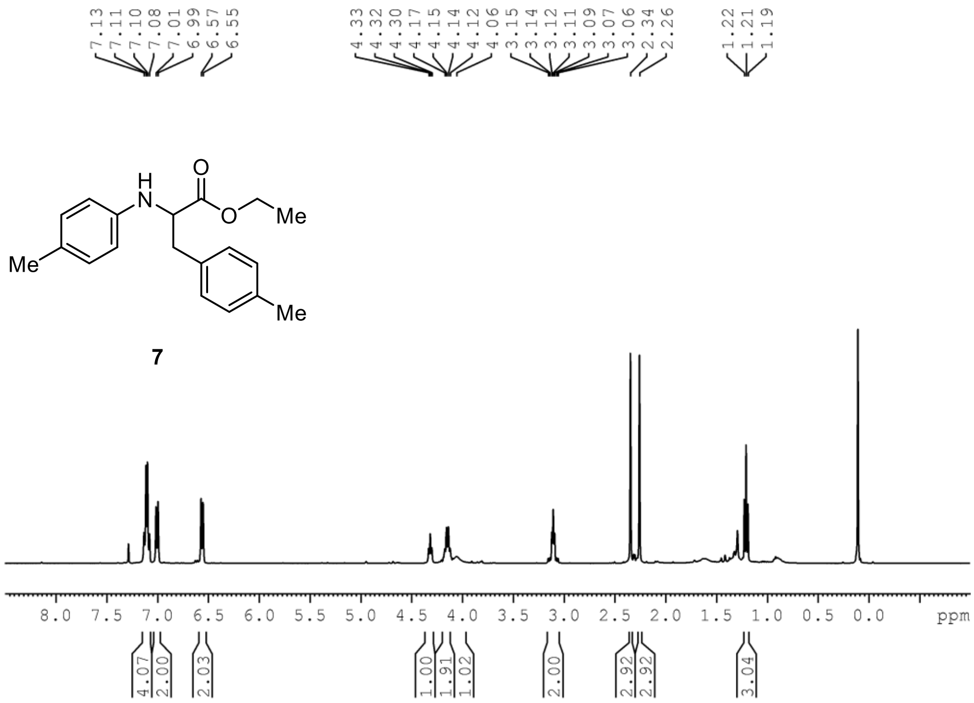

Supplementary Figure 6. ^{1}H NMR spectrum of compound 7

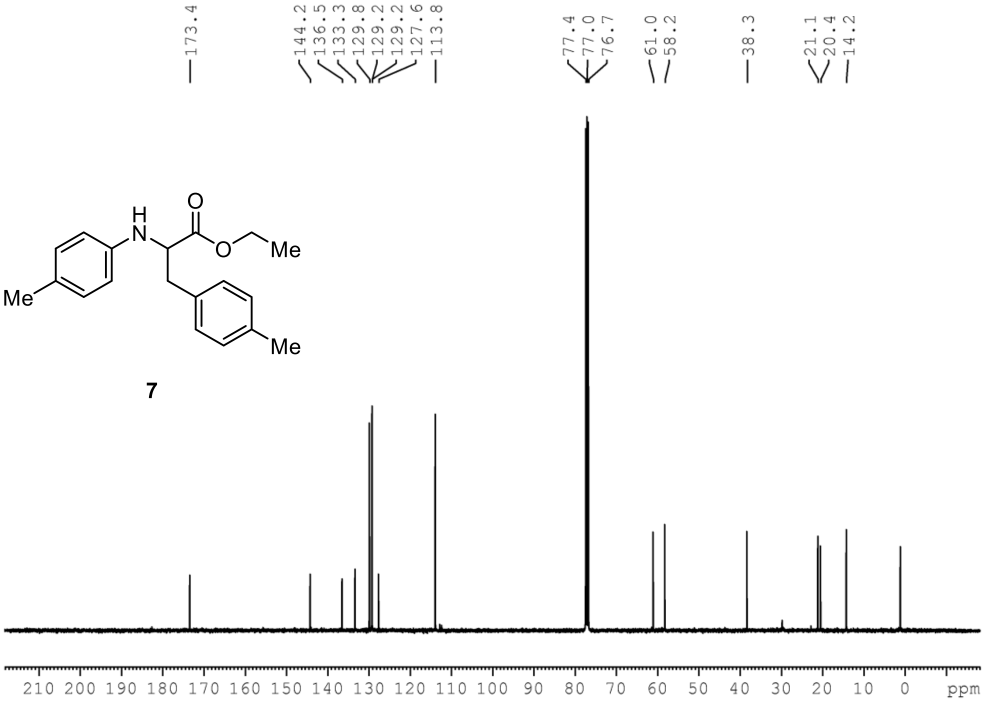

Supplementary Figure 7. ^{13}C NMR spectrum of compound 7

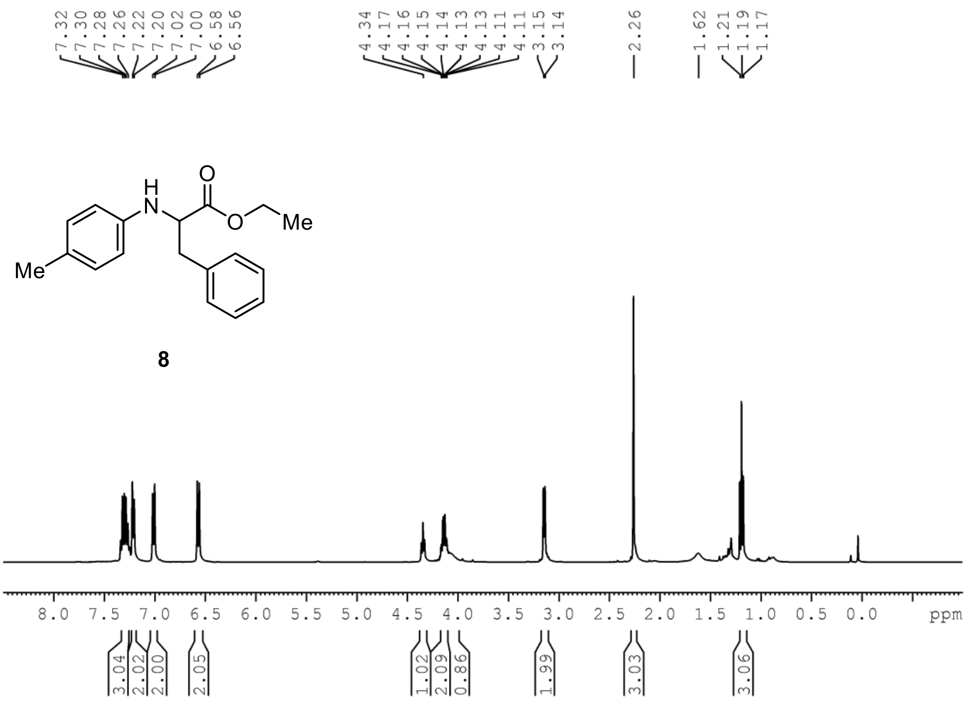

Supplementary Figure 8. ^{1}H NMR spectrum of compound 8

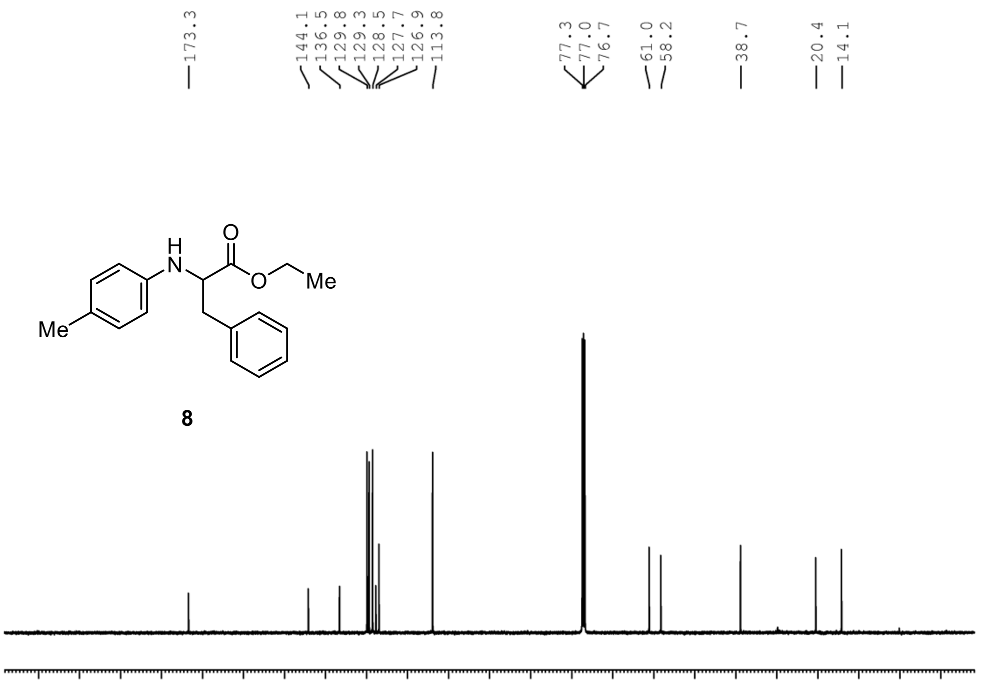

Supplementary Figure 9. ^{13}C NMR spectrum of compound 8

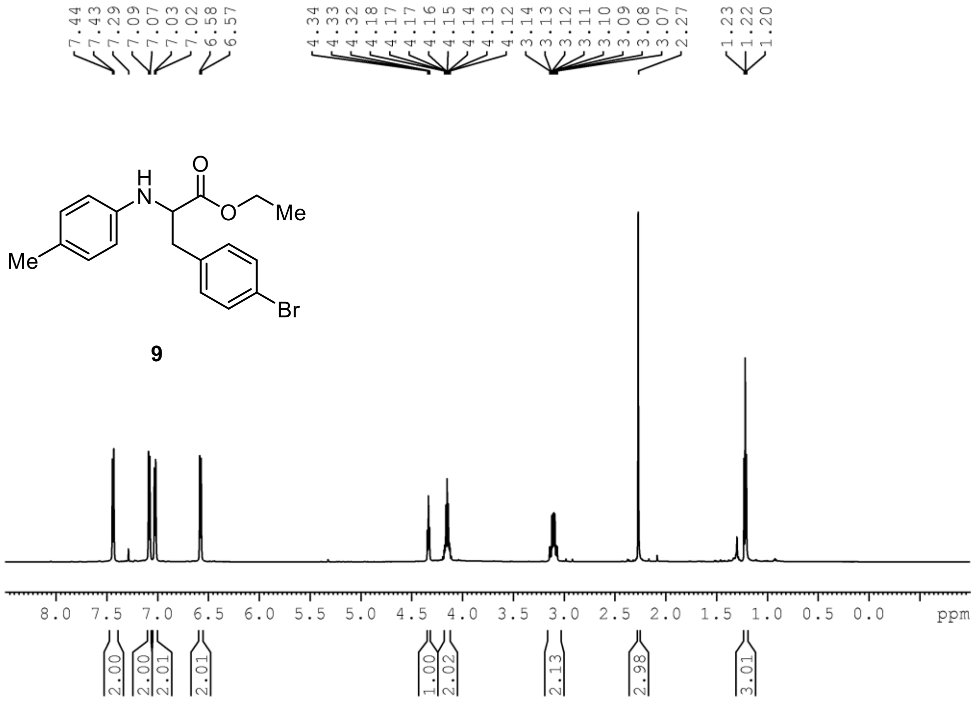

Supplementary Figure 10. ^{1}H NMR spectrum of compound 9

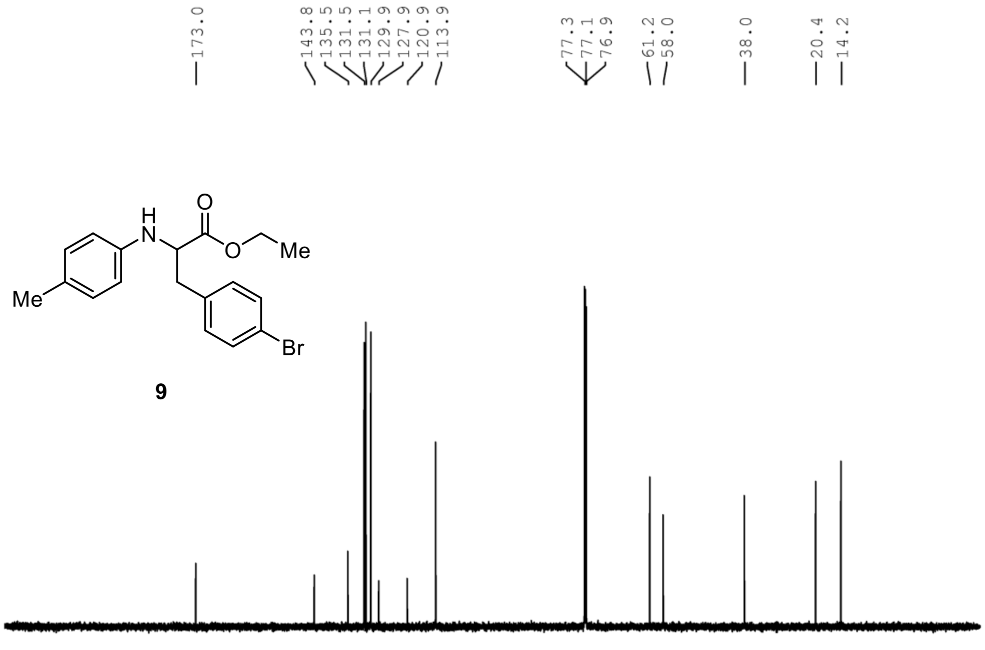

Supplementary Figure 11. ^{13}C NMR spectrum of compound 9

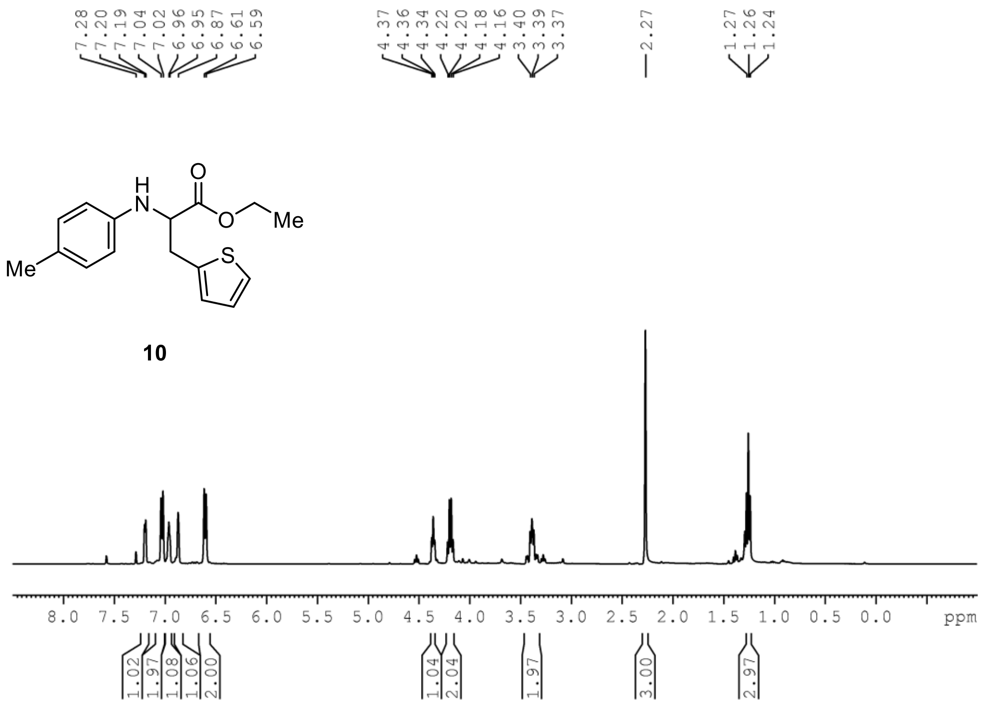

Supplementary Figure 12. ^{1}H NMR spectrum of compound 10

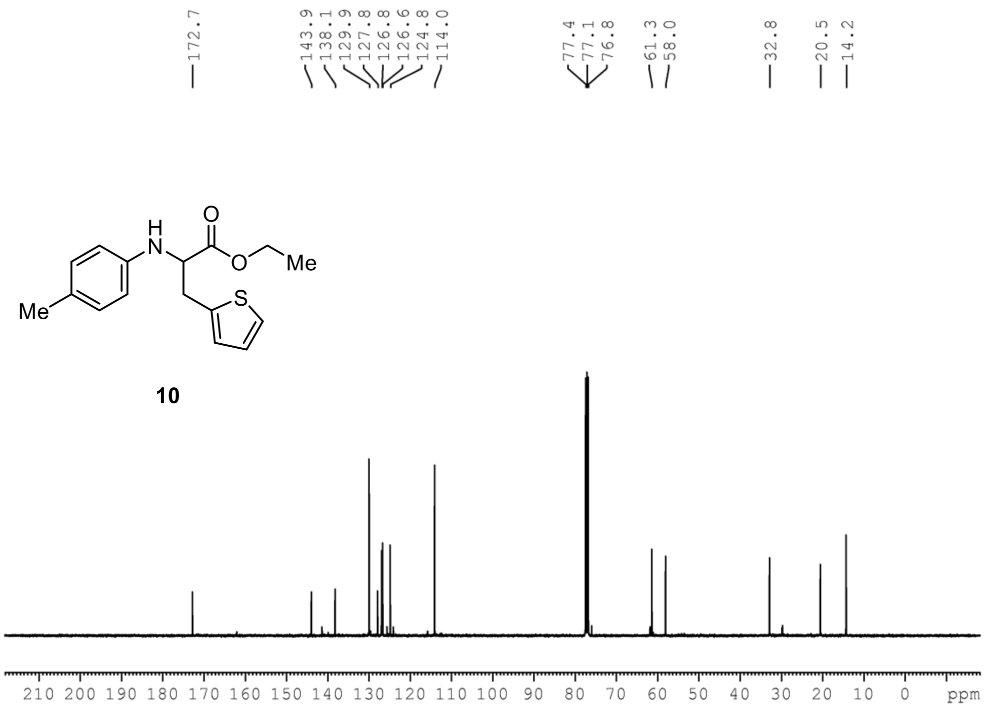

Supplementary Figure 13. ^{13}C NMR spectrum of compound 10

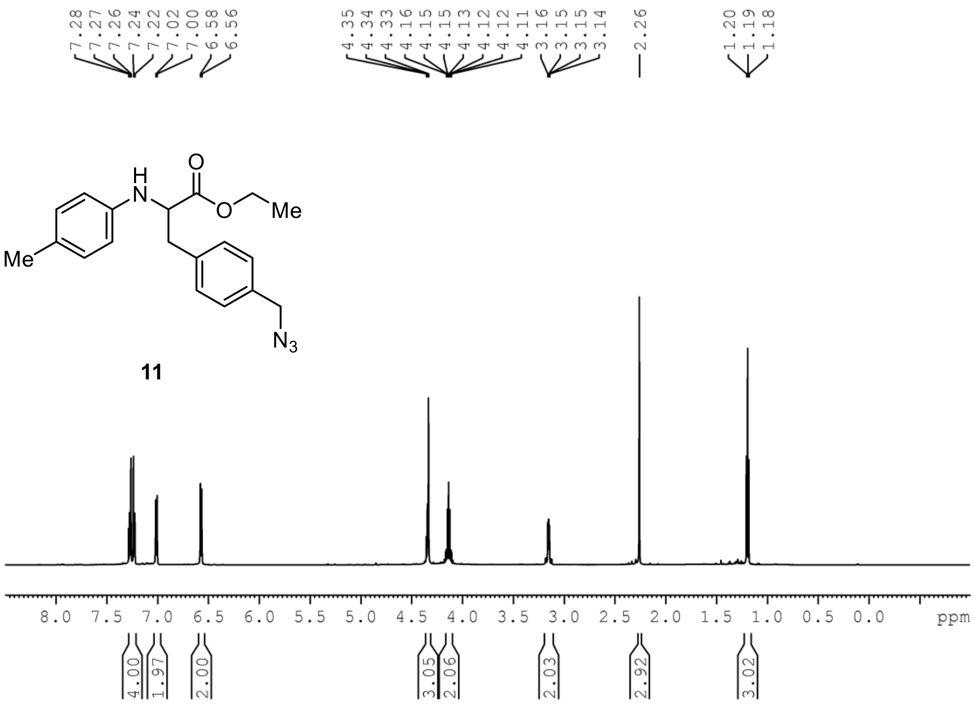

Supplementary Figure 14. ^{1}H NMR spectrum of compound 11

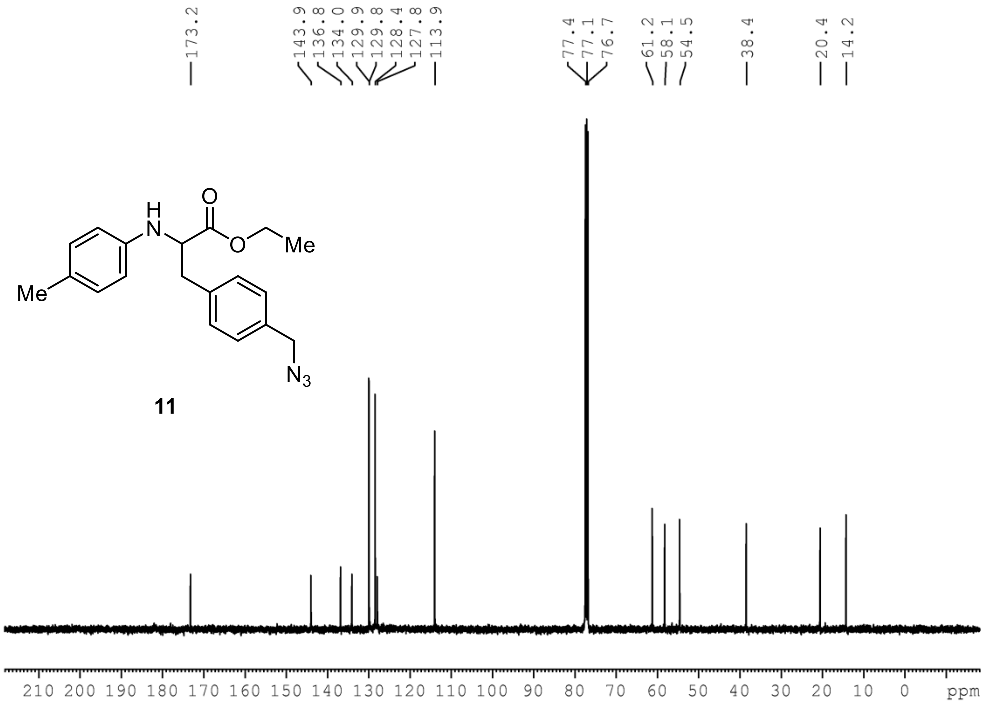

Supplementary Figure 15. ^{13}C NMR spectrum of compound 11

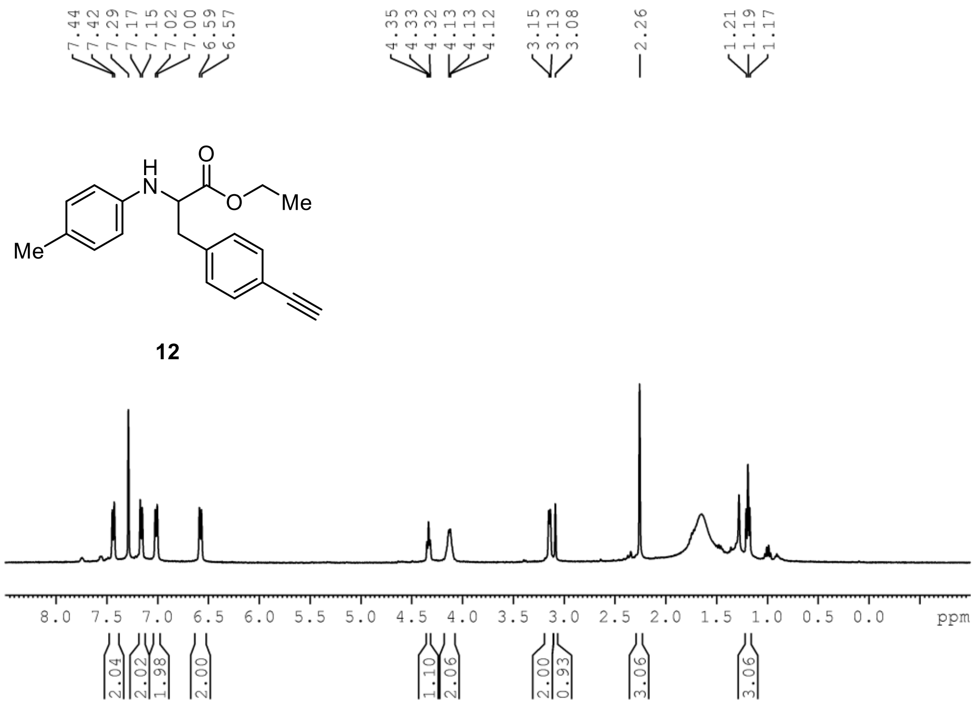

Supplementary Figure 16. ^{1}H NMR spectrum of compound 12

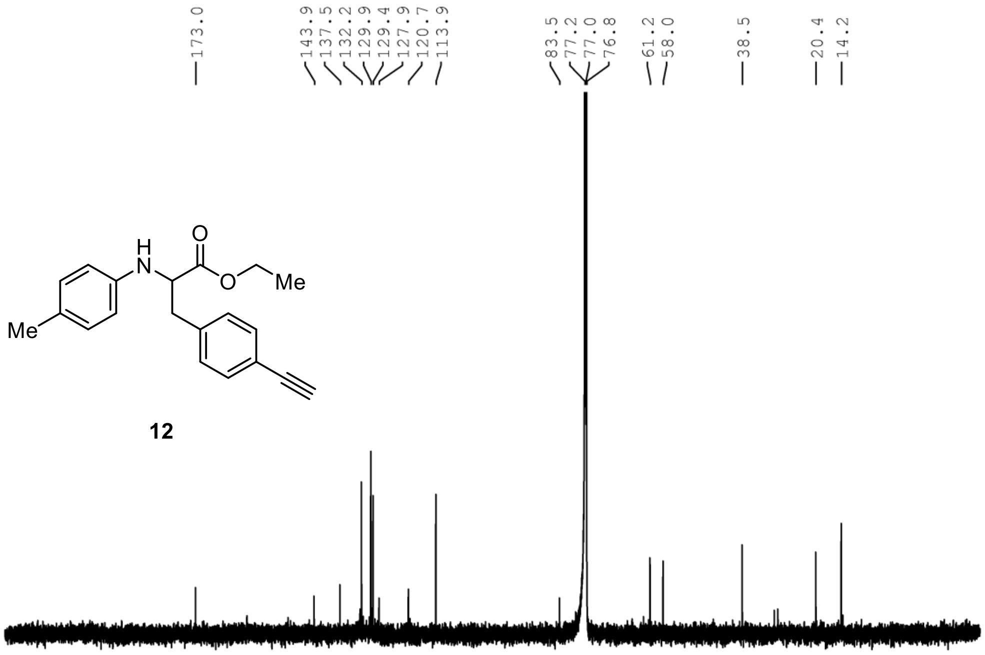

Supplementary Figure 17. ^{13}C NMR spectrum of compound 12

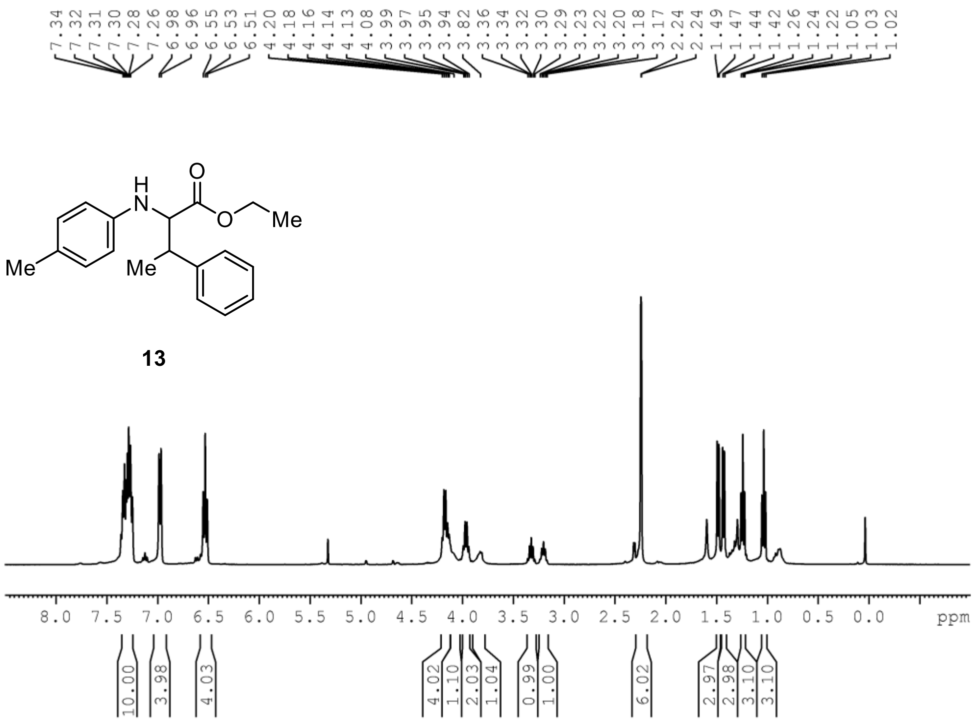

Supplementary Figure 18. ^{1}H NMR spectrum of compound 13

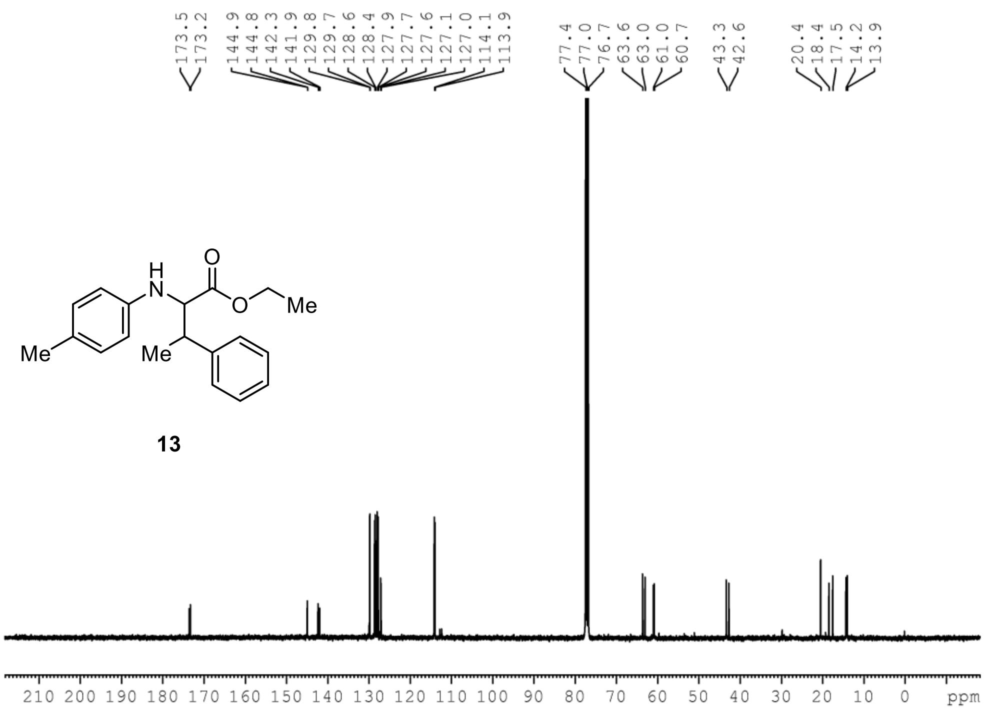

Supplementary Figure 19. ^{13}C NMR spectrum of compound 13

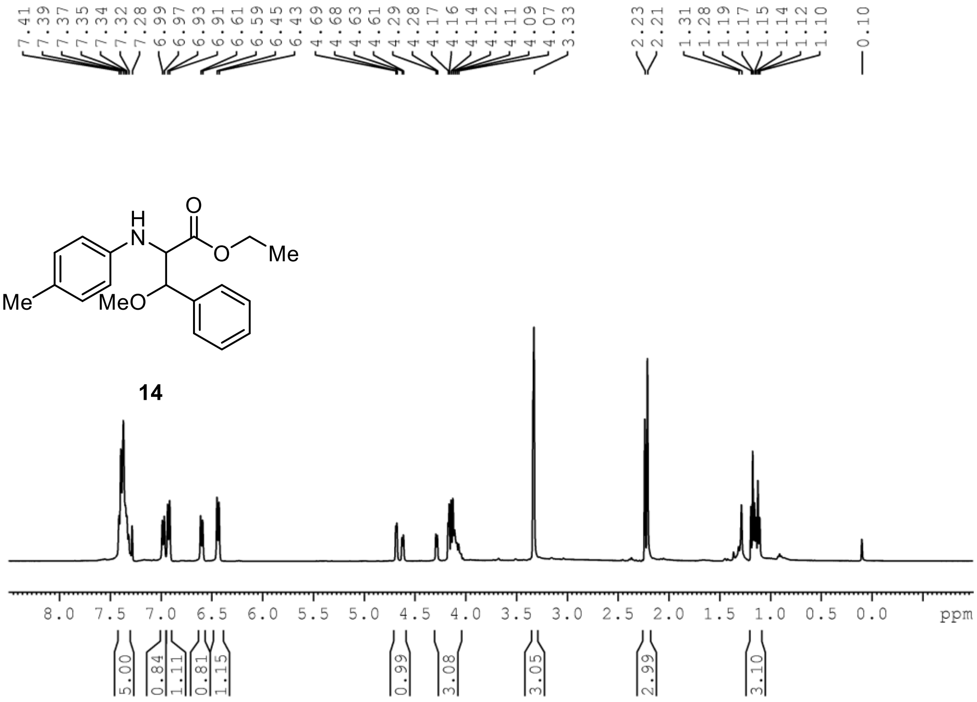

Supplementary Figure 20. ^{1}H NMR spectrum of compound 14

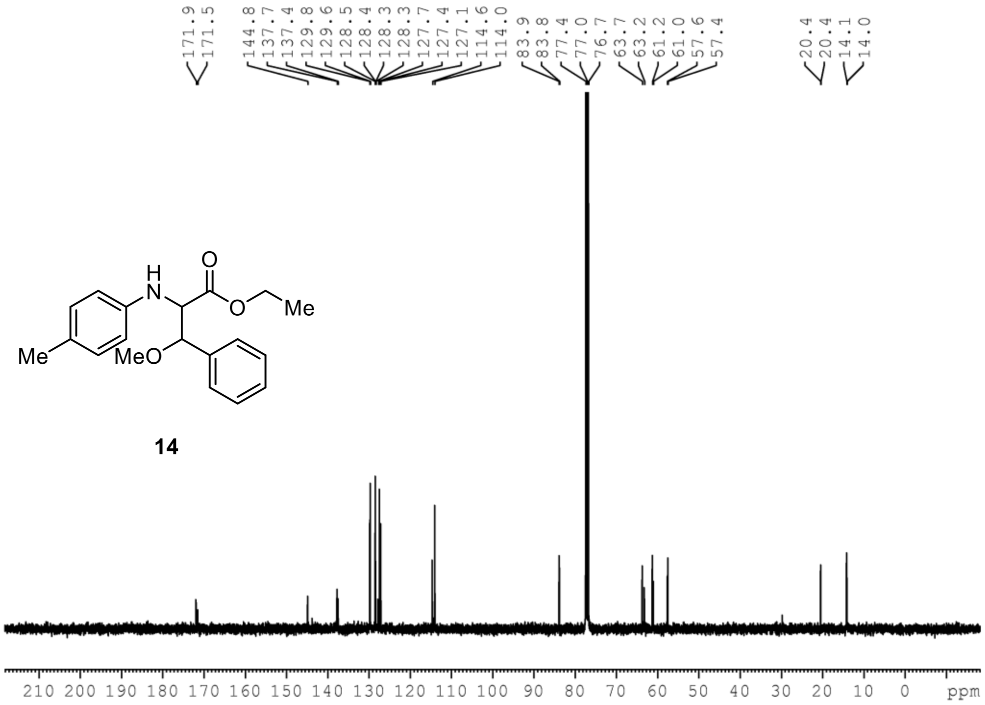

Supplementary Figure 21. ^{13}C NMR spectrum of compound 14

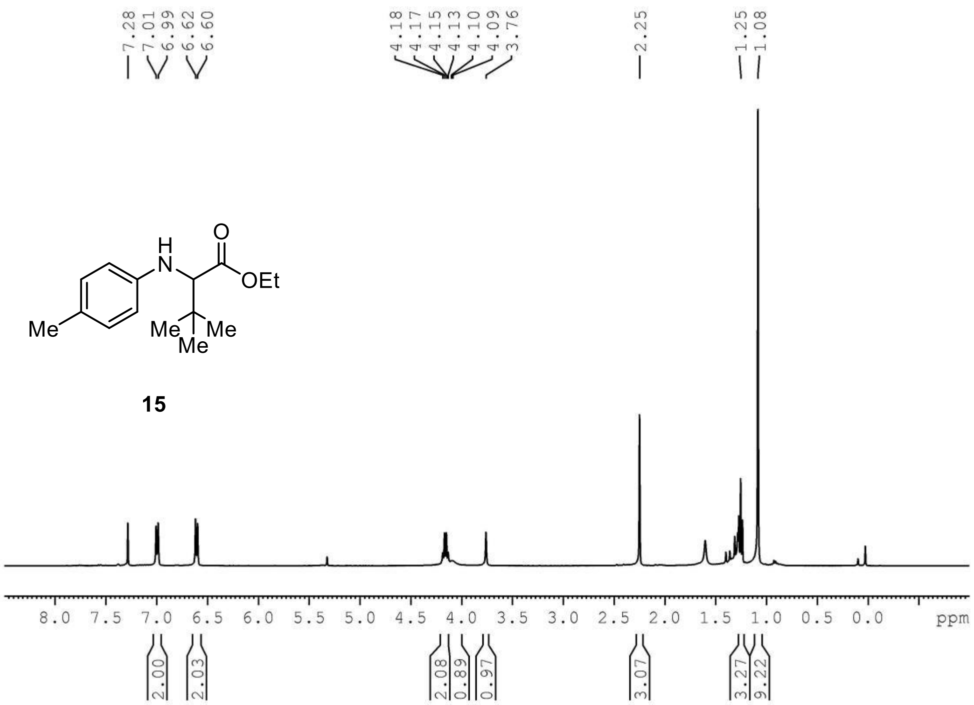

Supplementary Figure 22. ^{1}H NMR spectrum of compound 15

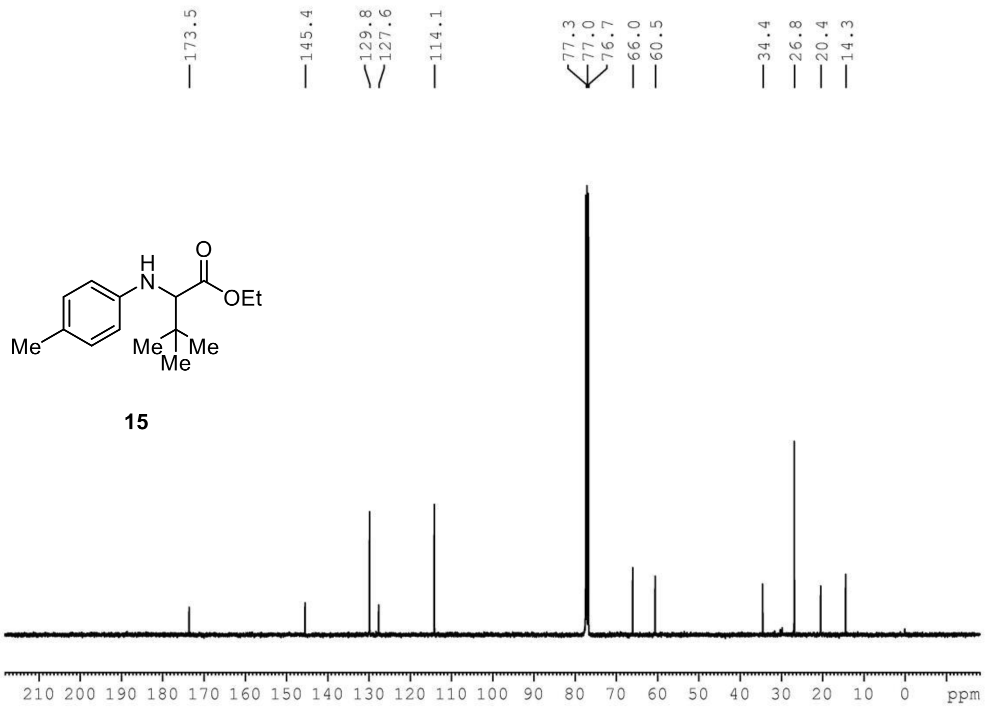

Supplementary Figure 23. ^{13}C NMR spectrum of compound 15

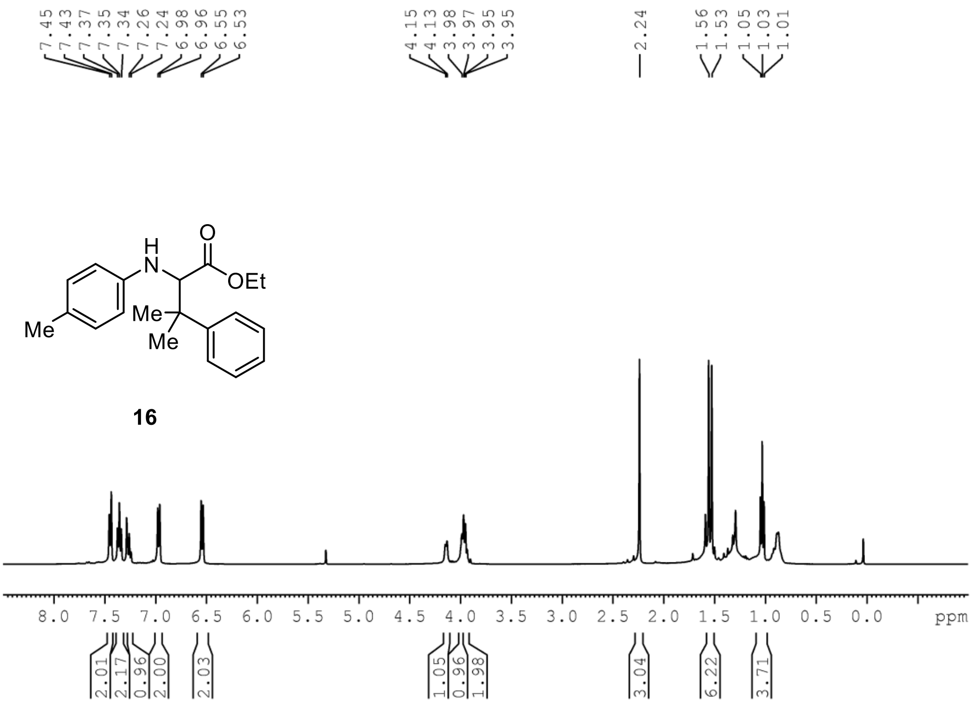

Supplementary Figure 24. ^{1}H NMR spectrum of compound 16

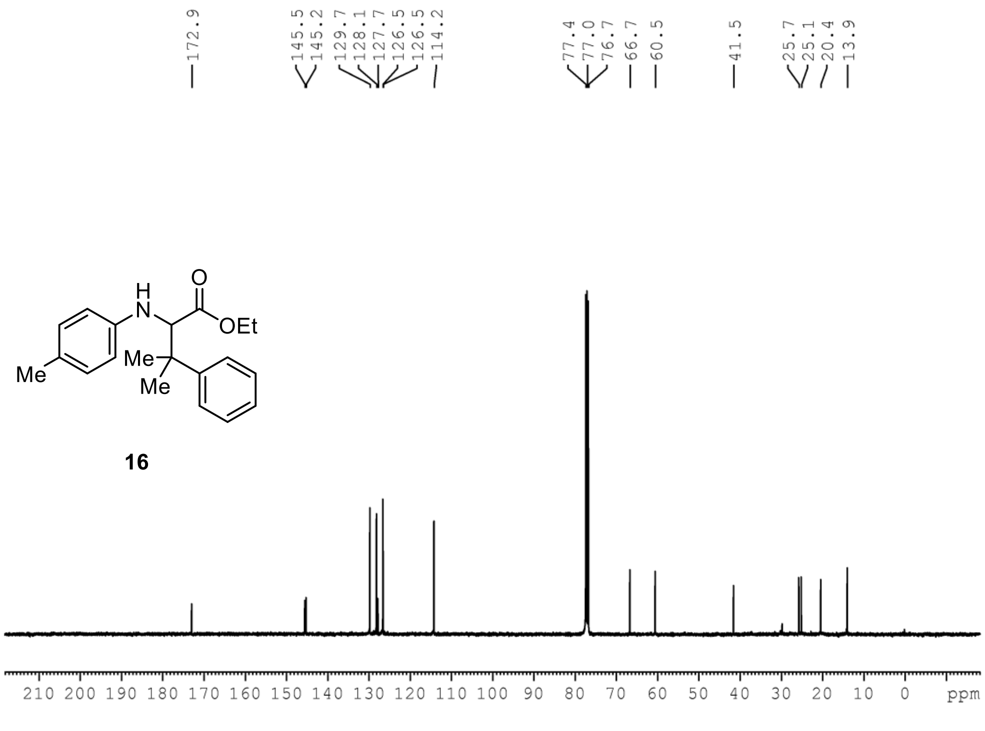

Supplementary Figure 25. ^{13}C NMR spectrum of compound 16

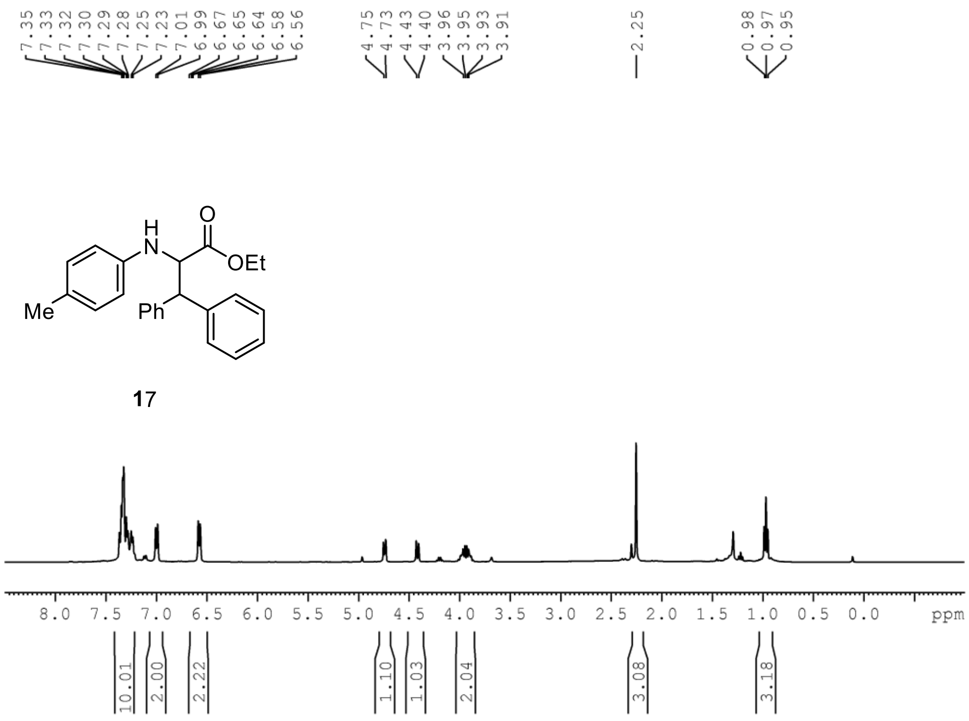

Supplementary Figure 26. ^{1}H NMR spectrum of compound 17

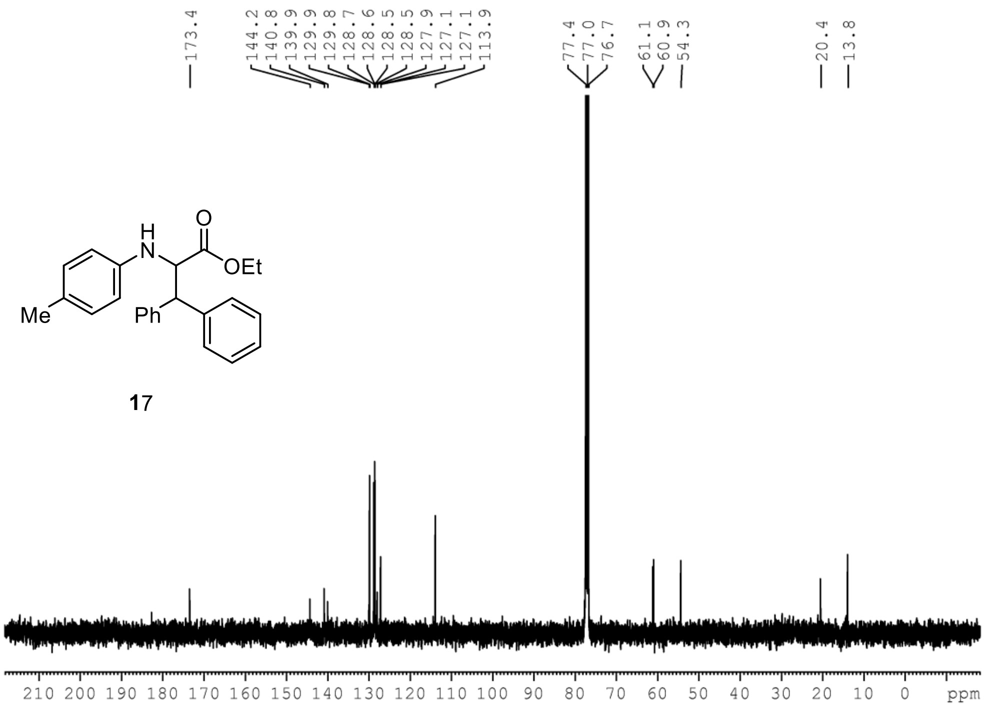

Supplementary Figure 27. ^{13}C NMR spectrum of compound 17

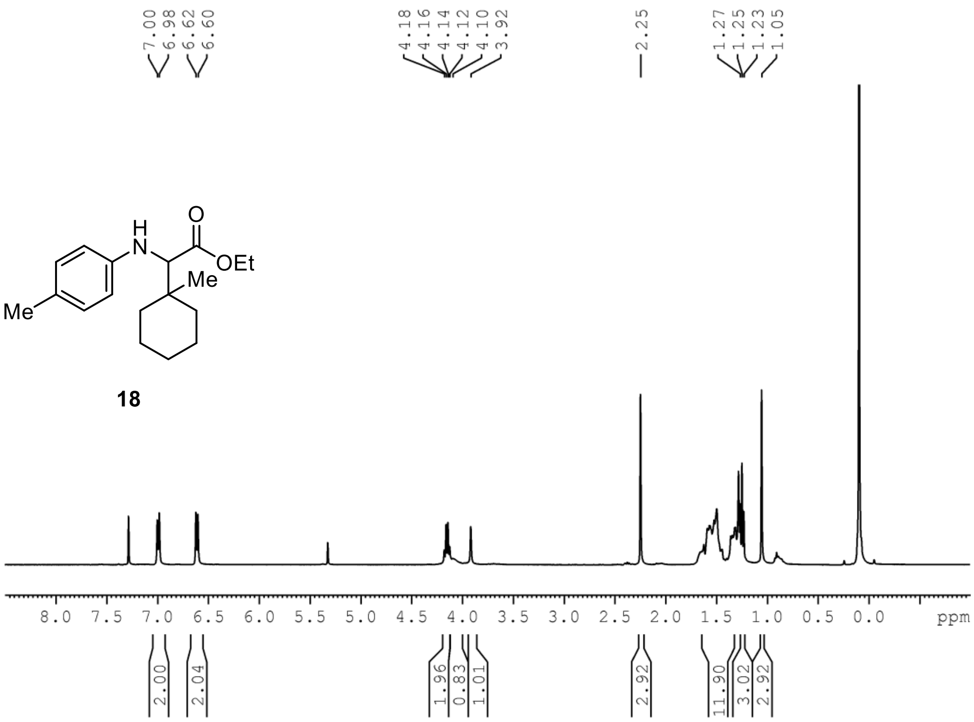

Supplementary Figure 28. ^{1}H NMR spectrum of compound 18

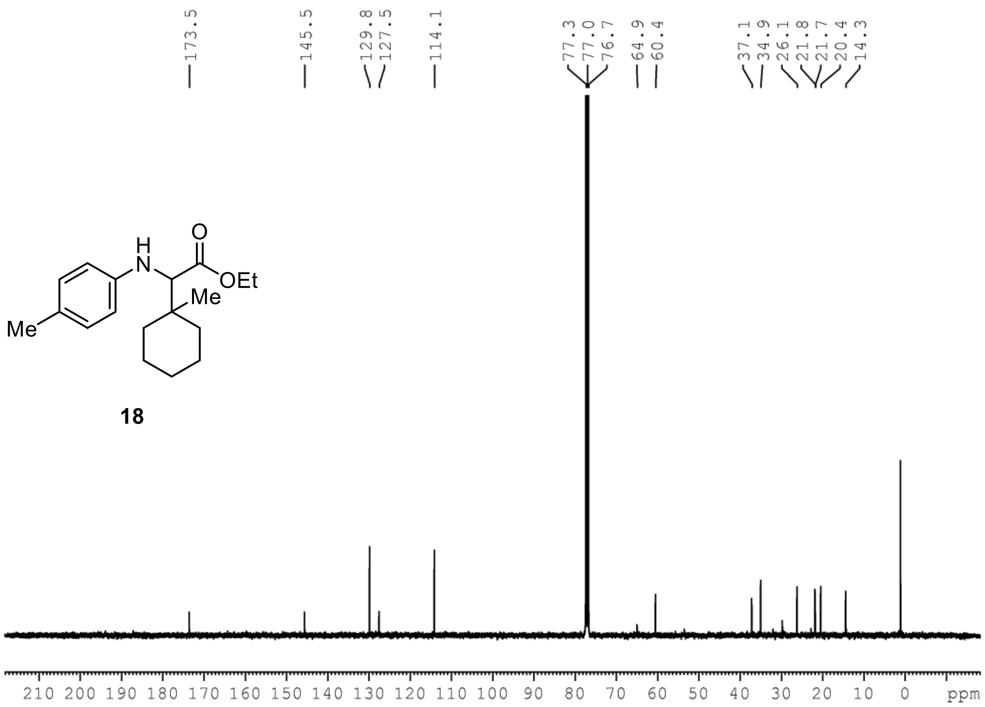

Supplementary Figure 29. ^{13}C NMR spectrum of compound 18

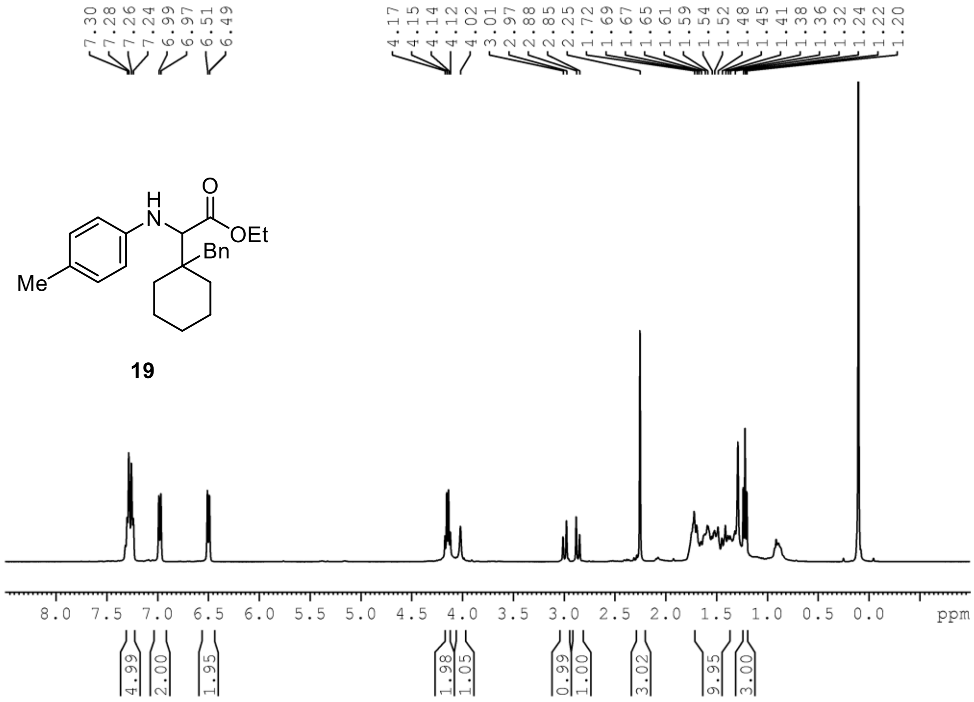

Supplementary Figure 30. ^{1}H NMR spectrum of compound 19

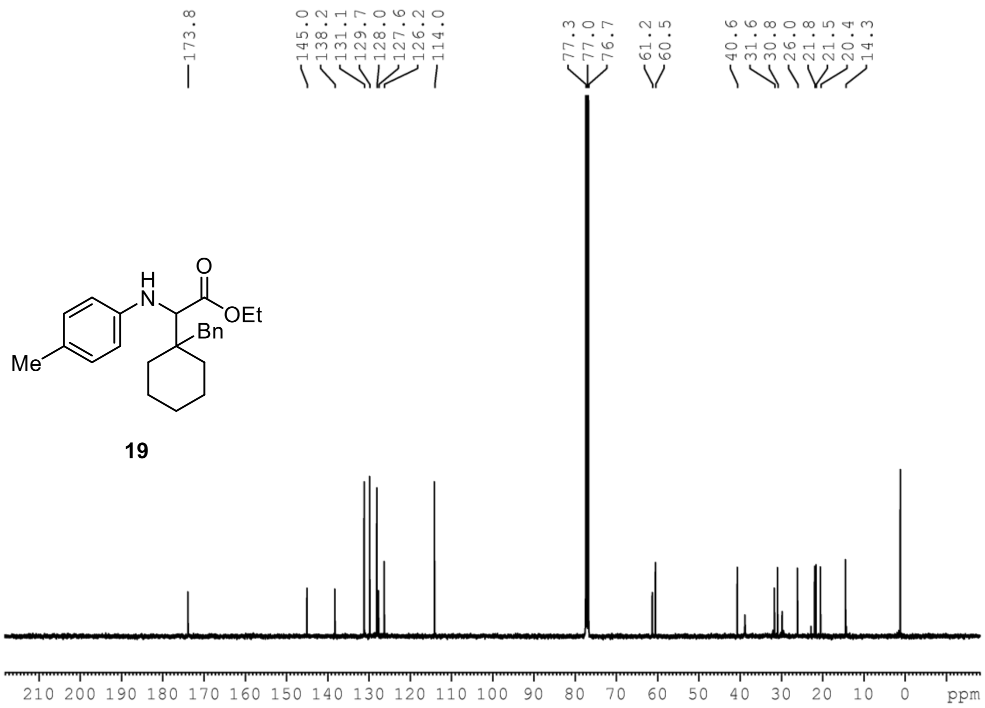

Supplementary Figure 31. ^{13}C NMR spectrum of compound 19

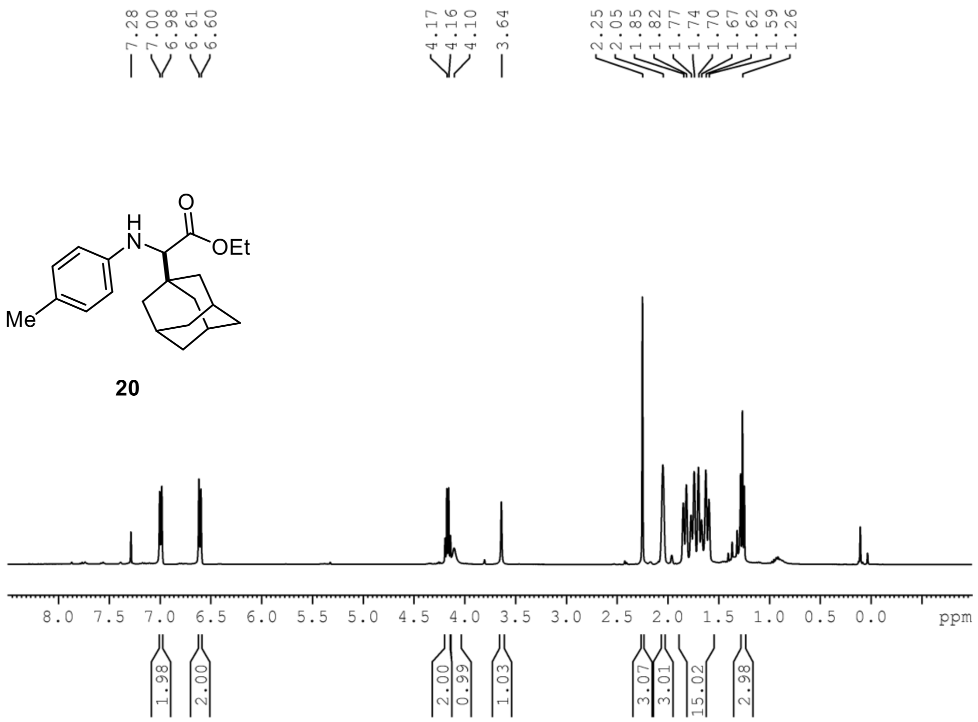

Supplementary Figure 32. ^{1}H NMR spectrum of compound 20

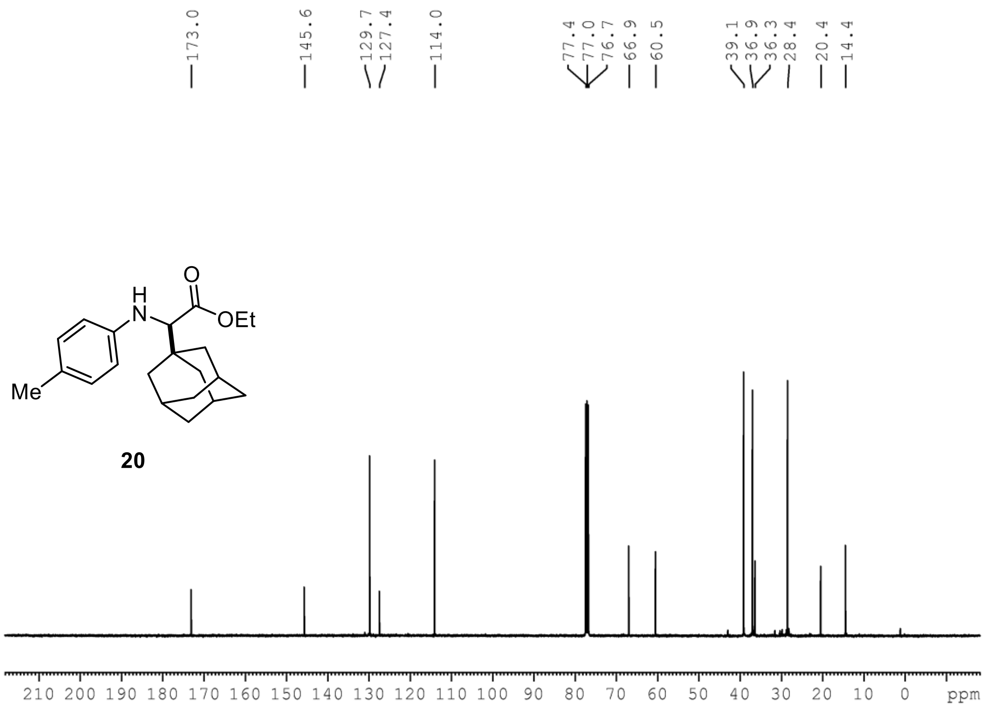

Supplementary Figure 33. ^{13}C NMR spectrum of compound 20

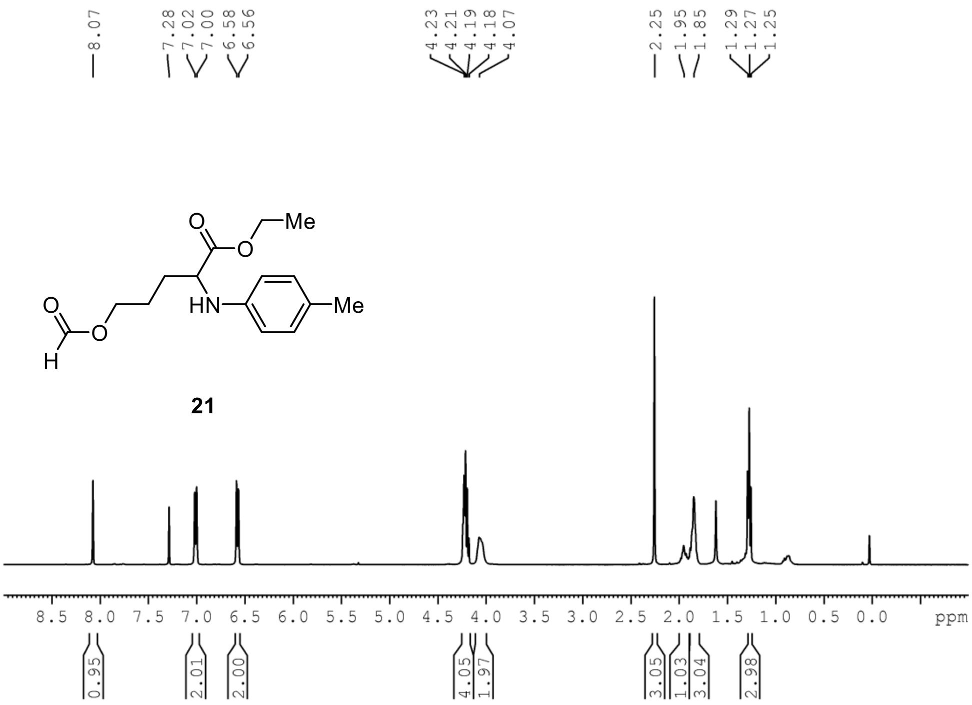

Supplementary Figure 34. ^{1}H NMR spectrum of compound 21

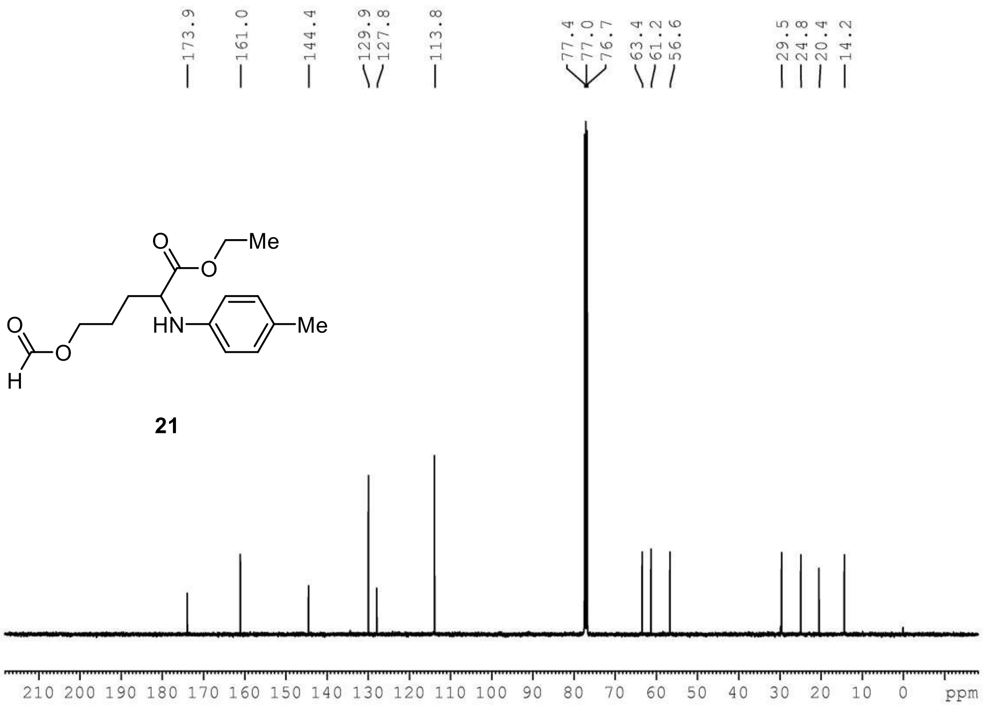

Supplementary Figure 35. ^{13}C NMR spectrum of compound 21

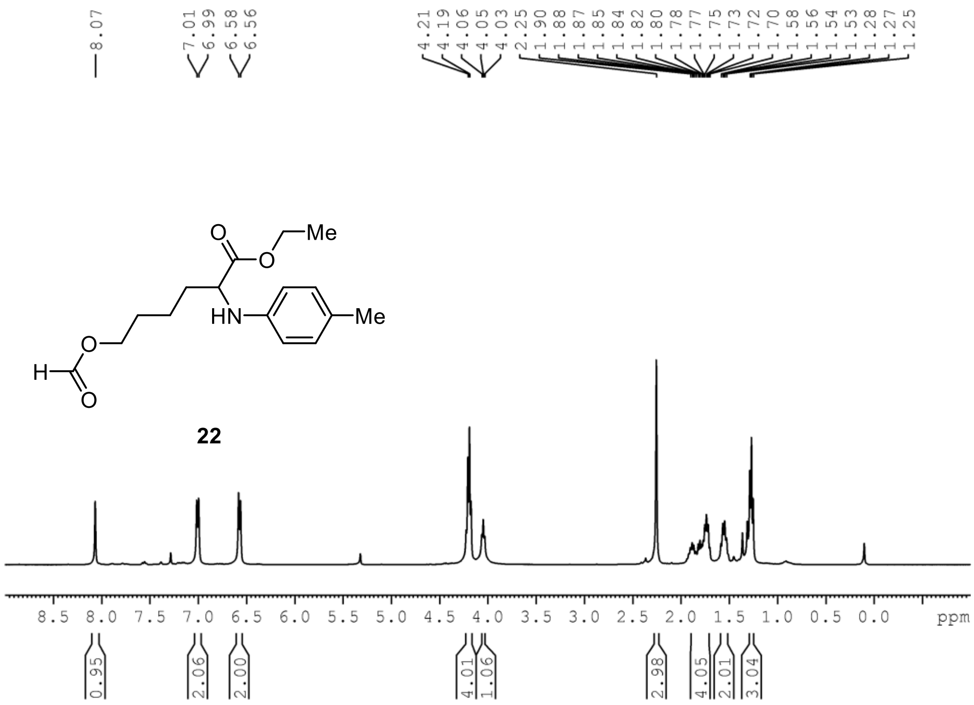

Supplementary Figure 36. ^{1}H NMR spectrum of compound 22

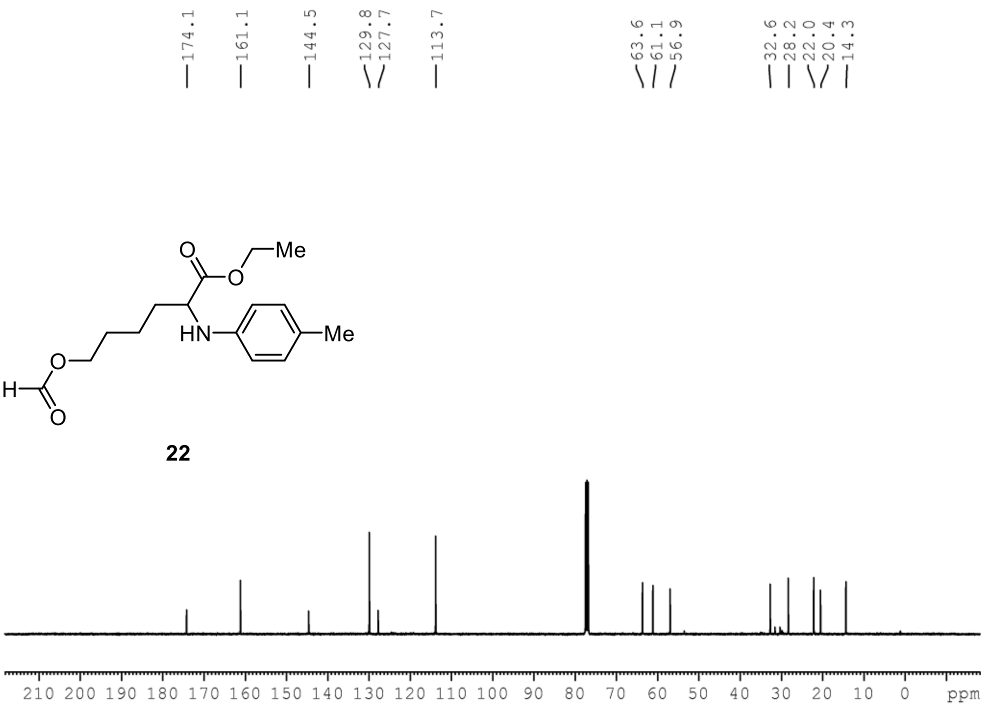

Supplementary Figure 37. ^{13}C NMR spectrum of compound 22

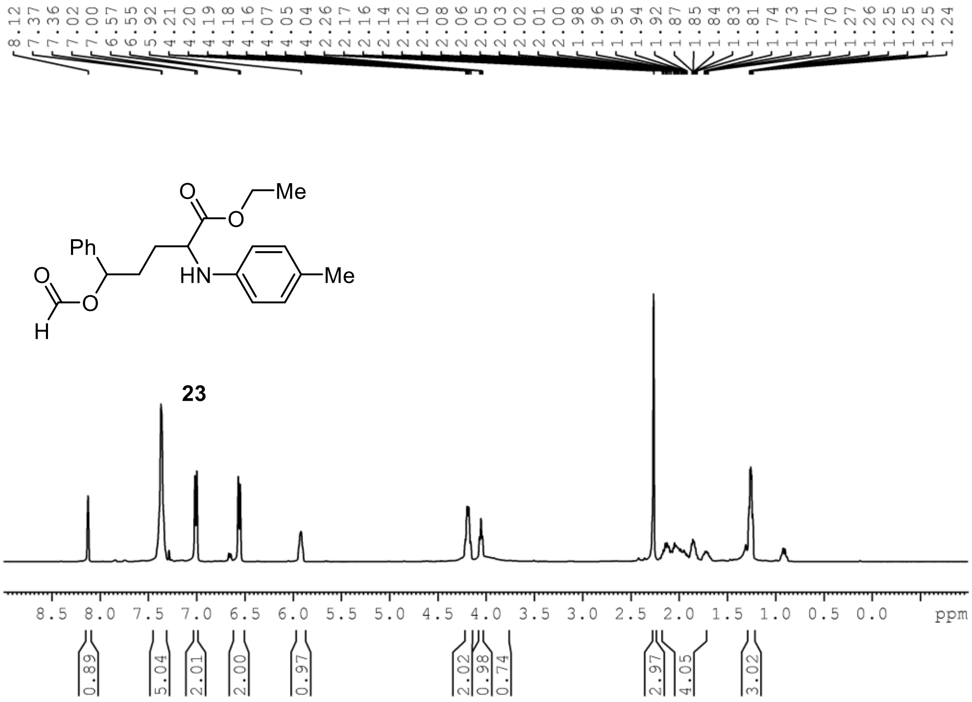

Supplementary Figure 38. ^{1}H NMR spectrum of compound 23

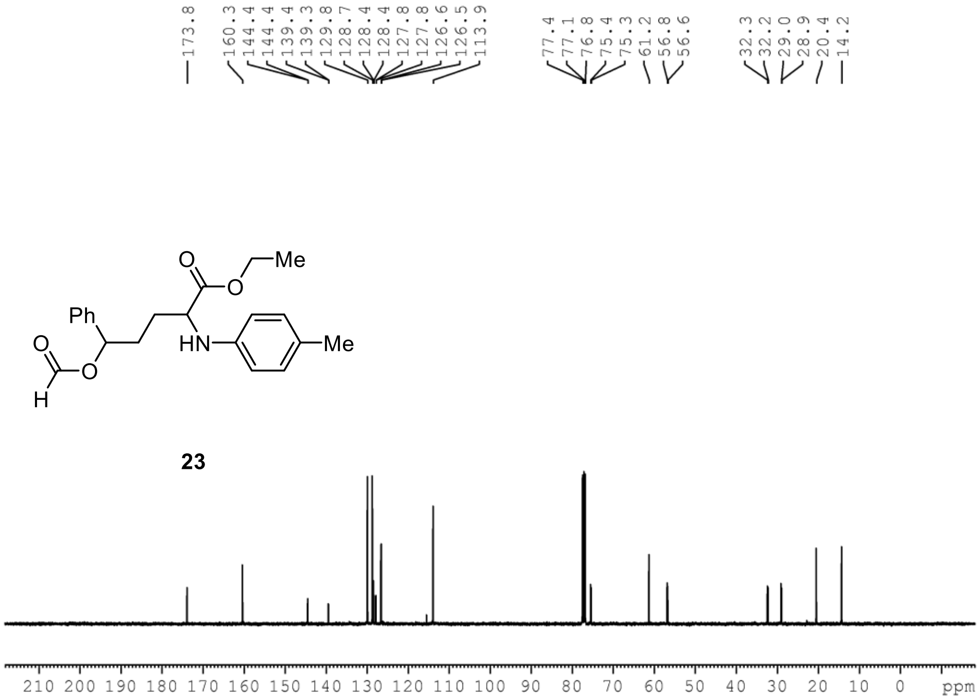

Supplementary Figure 39. ^{13}C NMR spectrum of compound 23

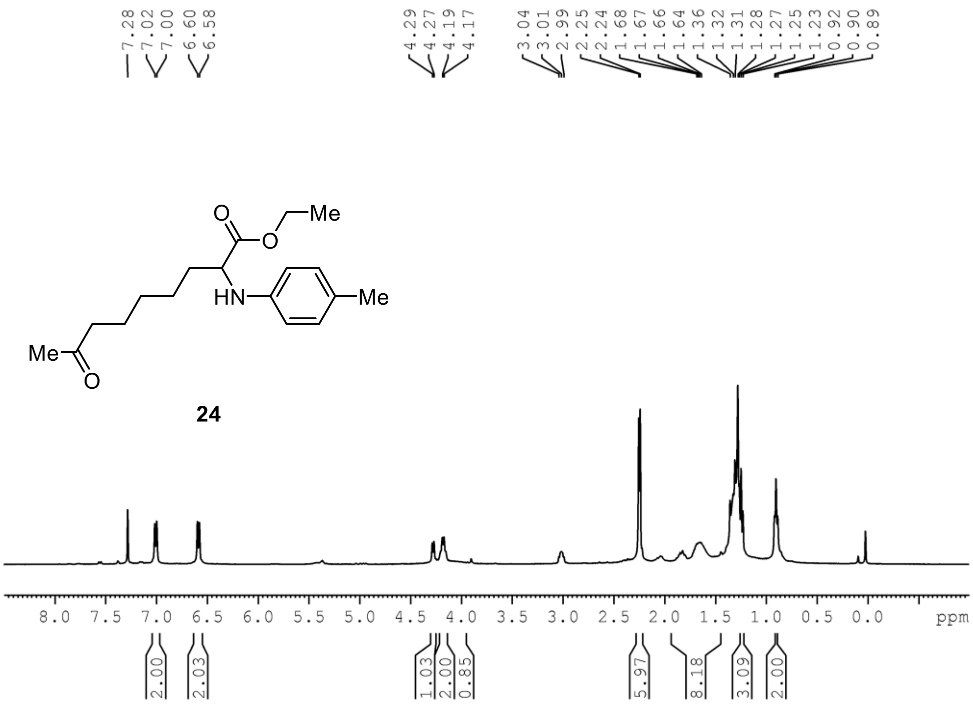

Supplementary Figure 40. ^{1}H NMR spectrum of compound 24

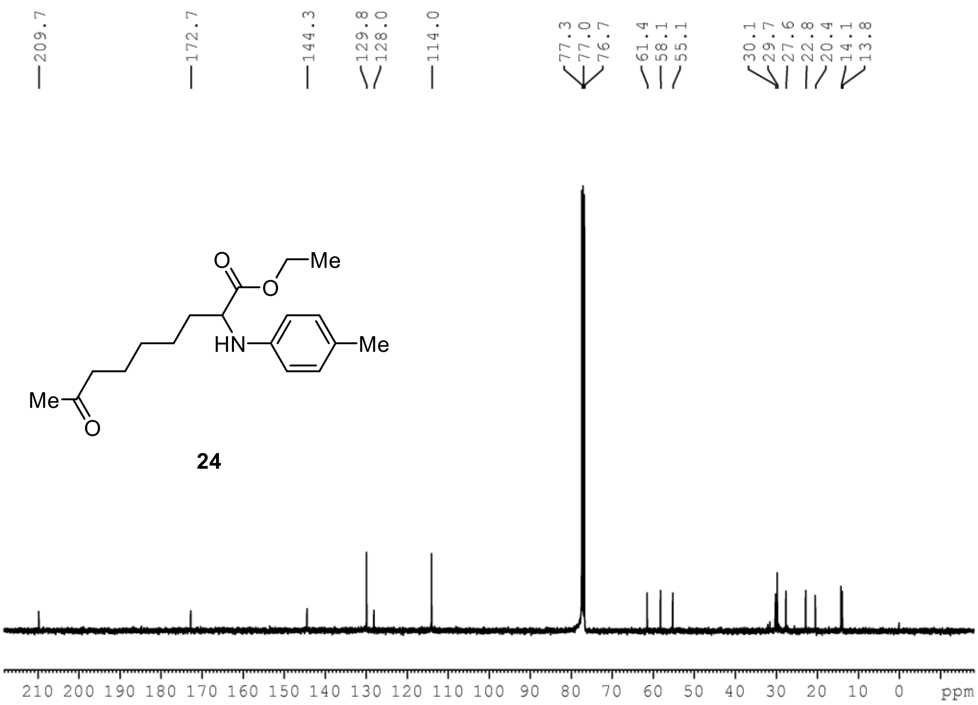

Supplementary Figure 41. ^{13}C NMR spectrum of compound 24

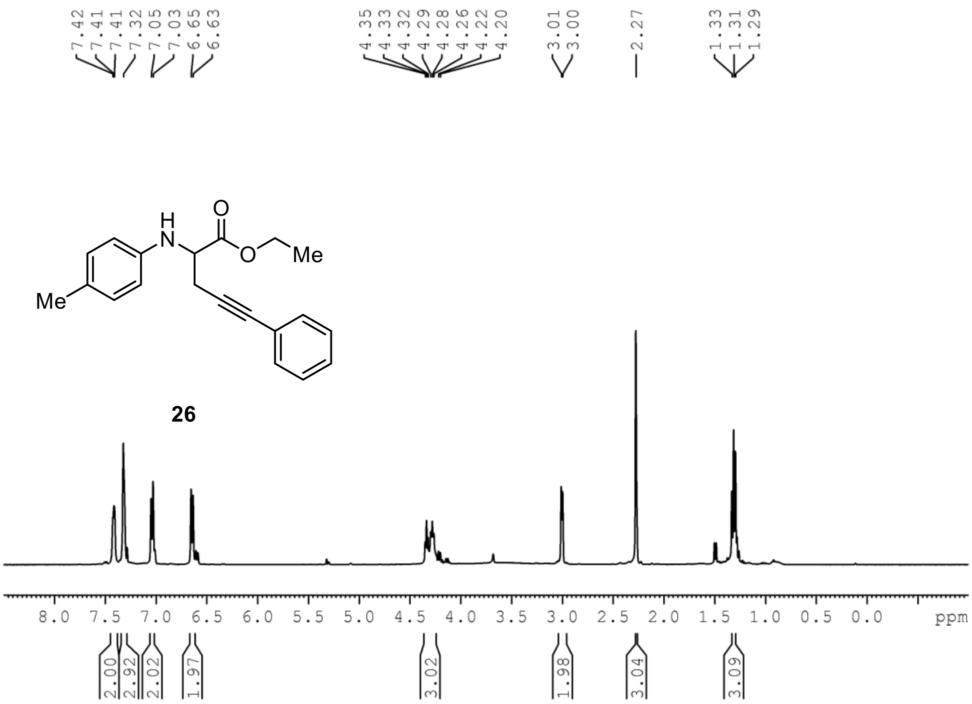

Supplementary Figure 42. ^{1}H NMR spectrum of compound 26

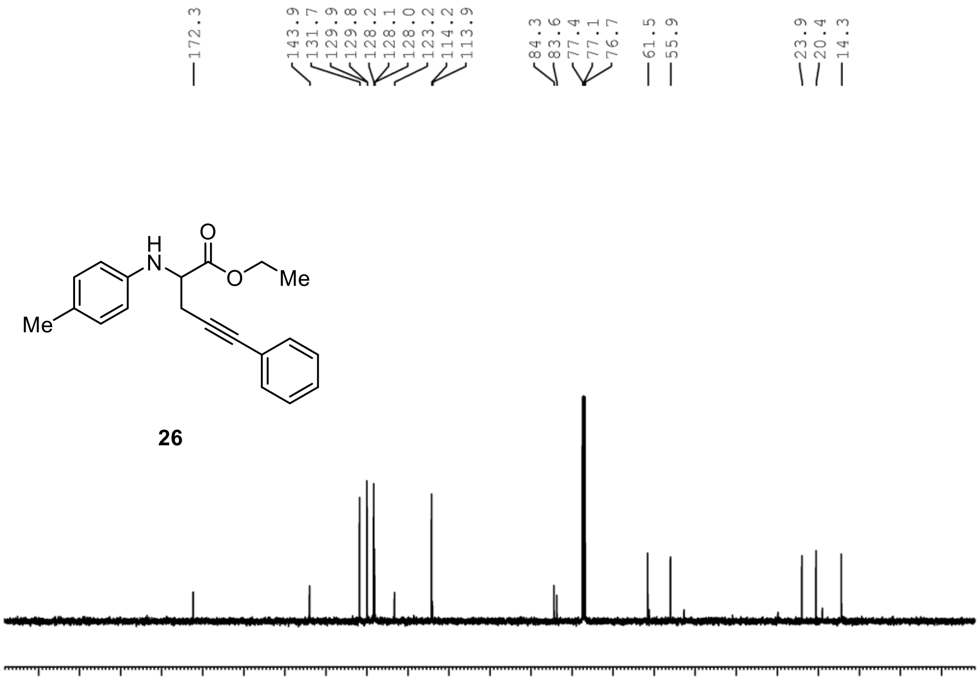

Supplementary Figure 43. ^{13}C NMR spectrum of compound 26

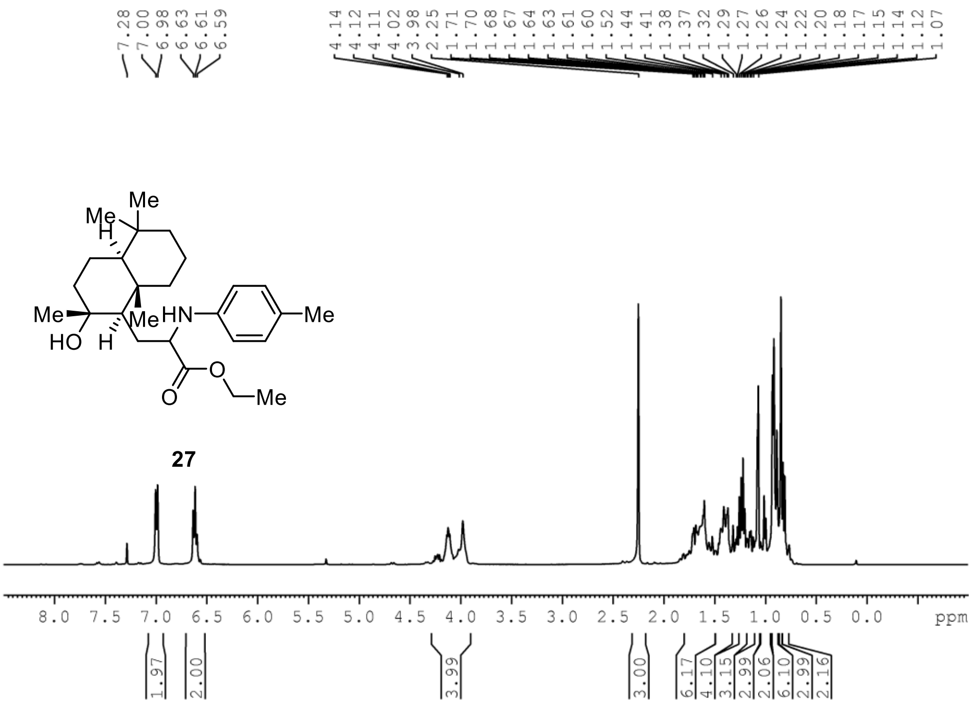

Supplementary Figure 44. ^{1}H NMR spectrum of compound 27

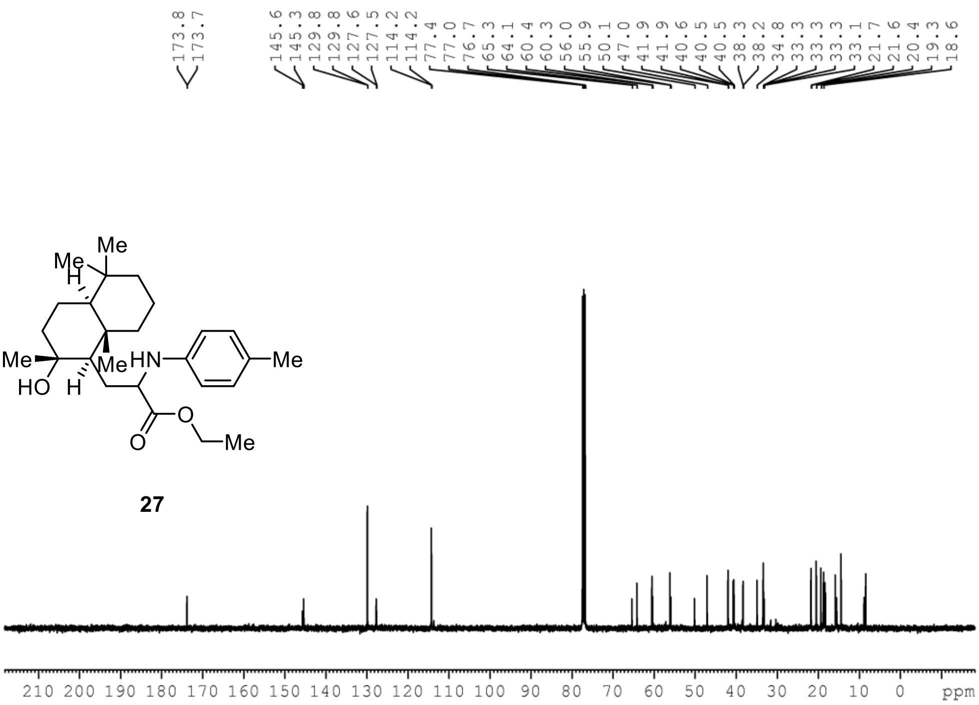

Supplementary Figure 45. ^{13}C NMR spectrum of compound 27

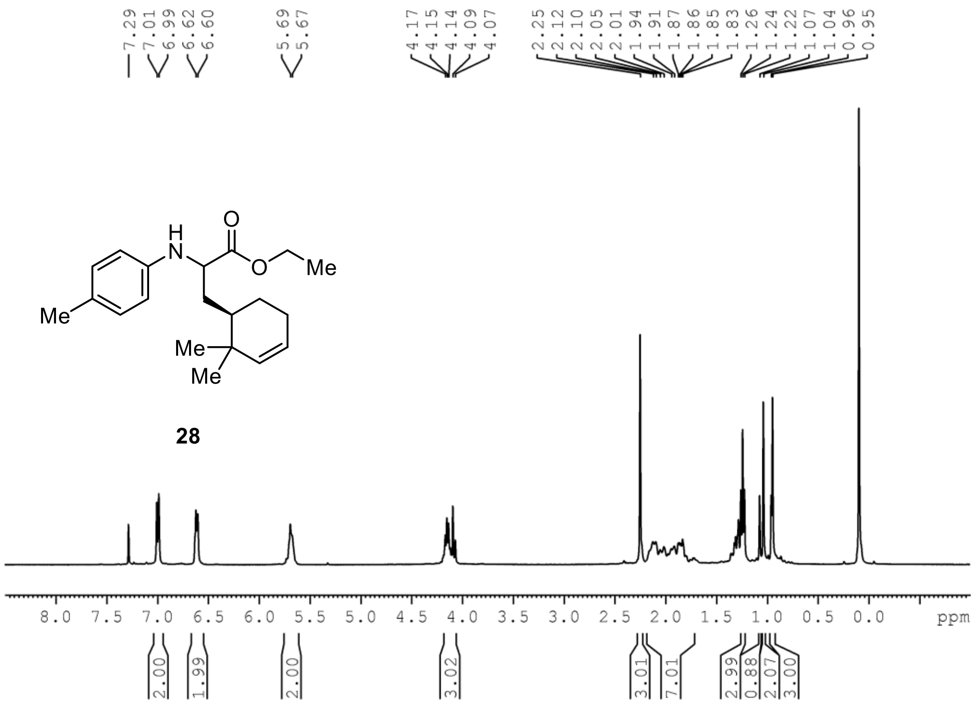

Supplementary Figure 46. ^{1}H NMR spectrum of compound 28

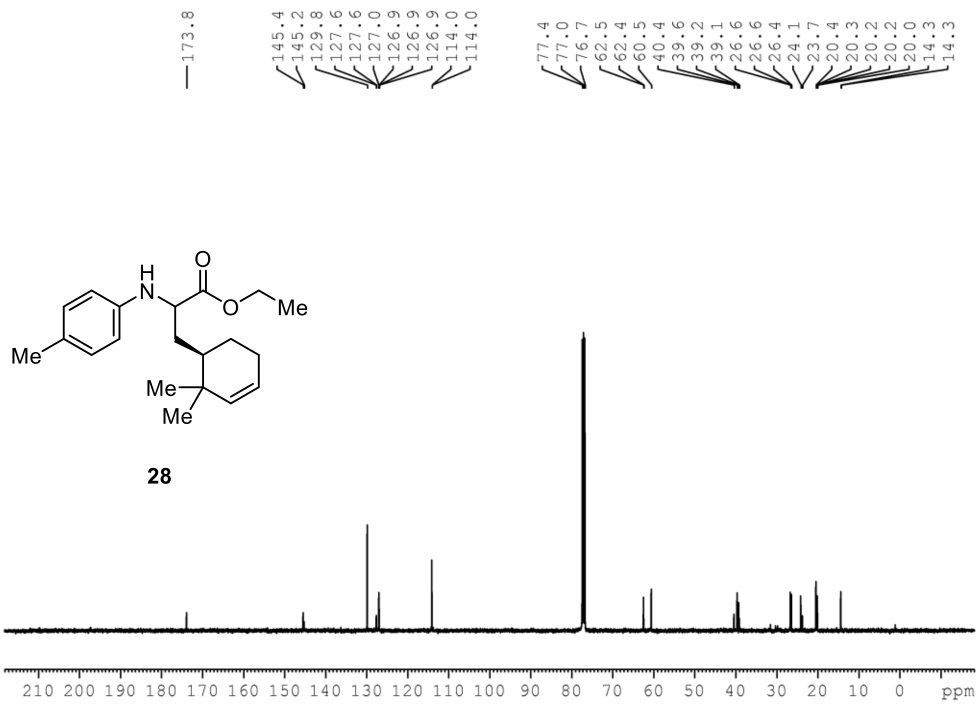

Supplementary Figure 47. ^{13}C NMR spectrum of compound 28

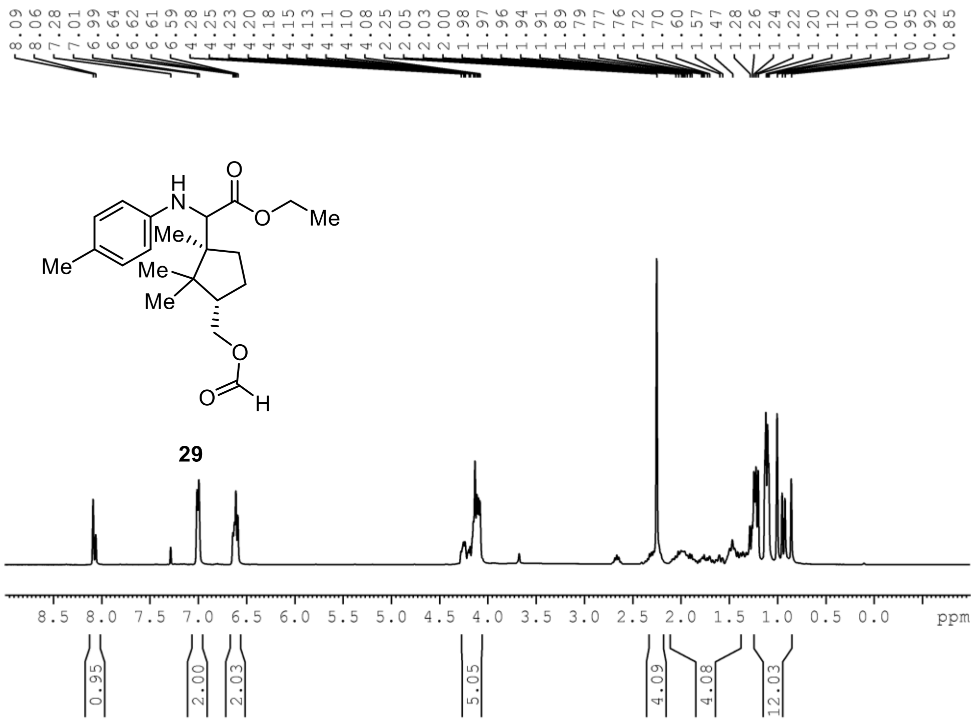

Supplementary Figure 48. ¹H NMR spectrum of compound 29

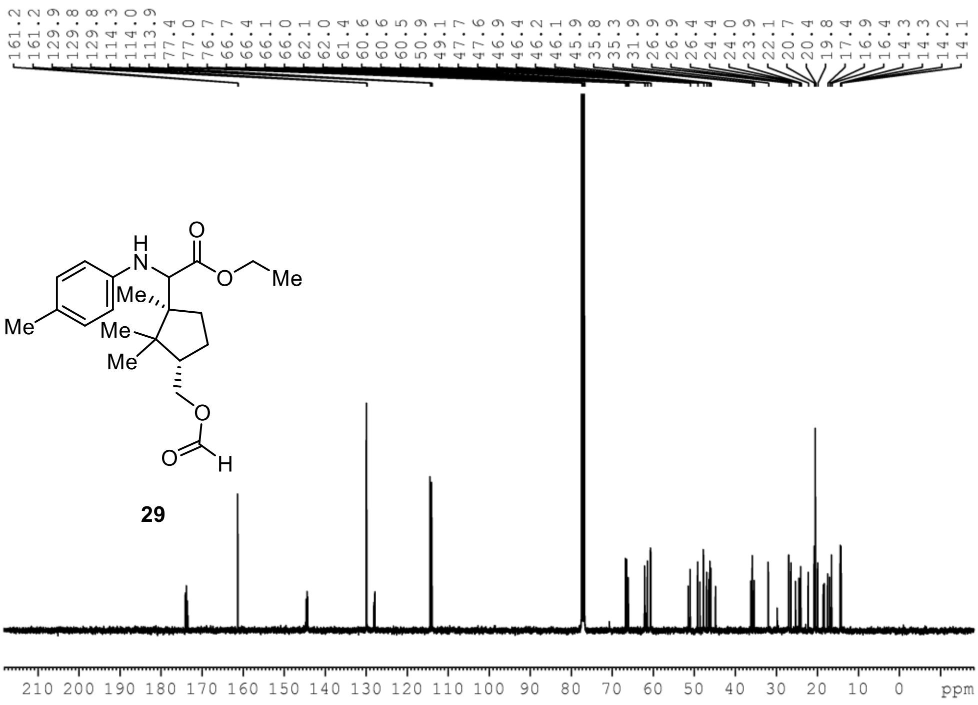

Supplementary Figure 49. ¹³C NMR spectrum of compound 29

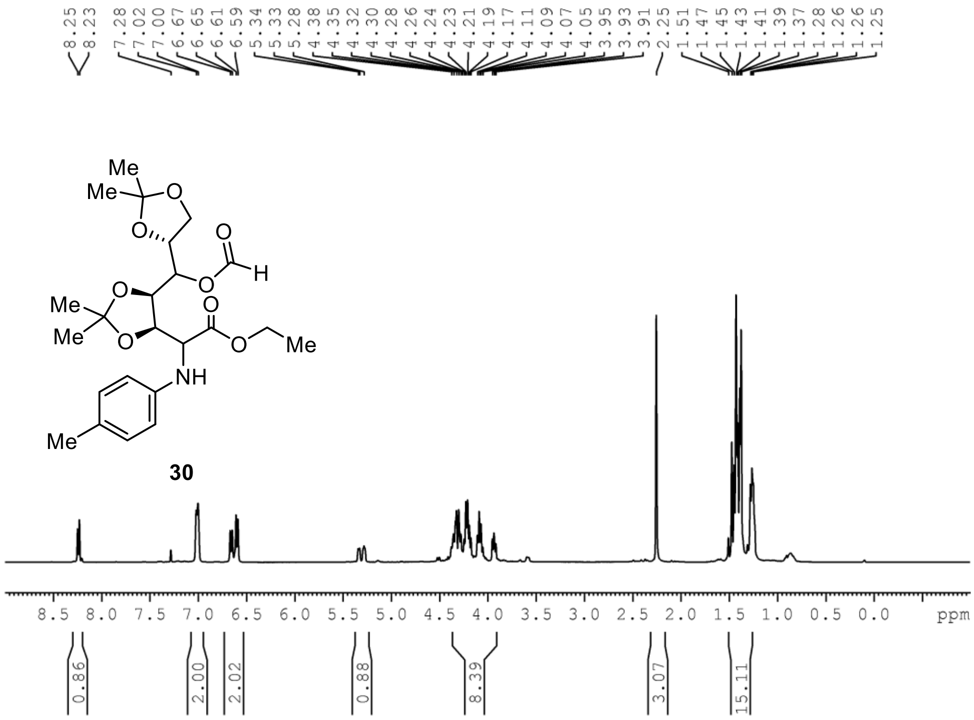

Supplementary Figure 50. ^{1}H NMR spectrum of compound 30

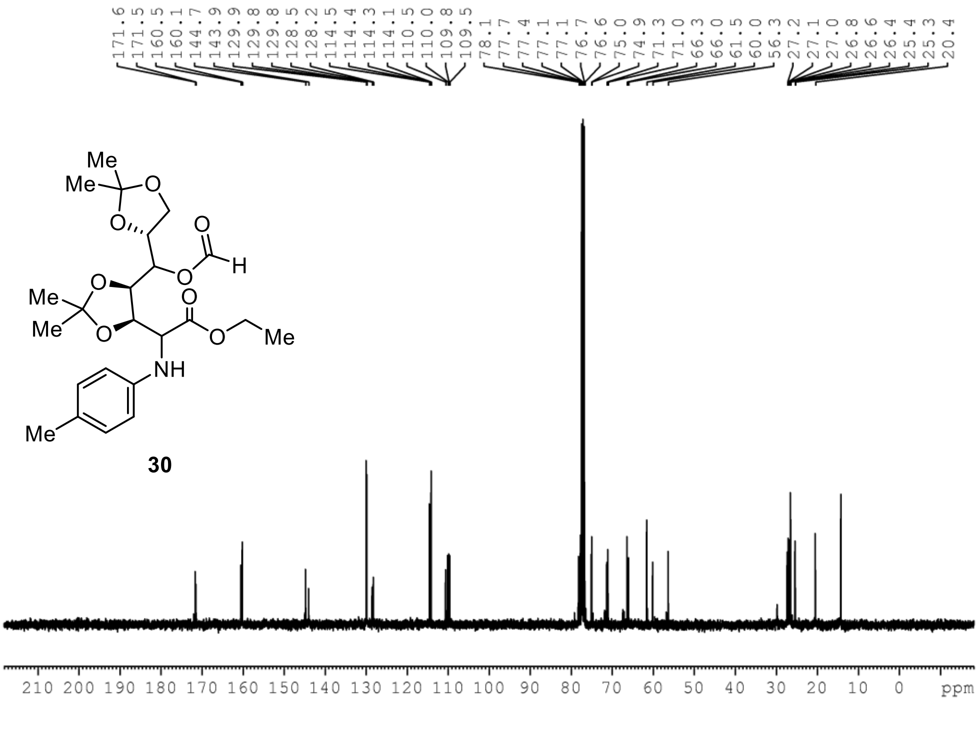

Supplementary Figure 51. ^{13}C NMR spectrum of compound 30

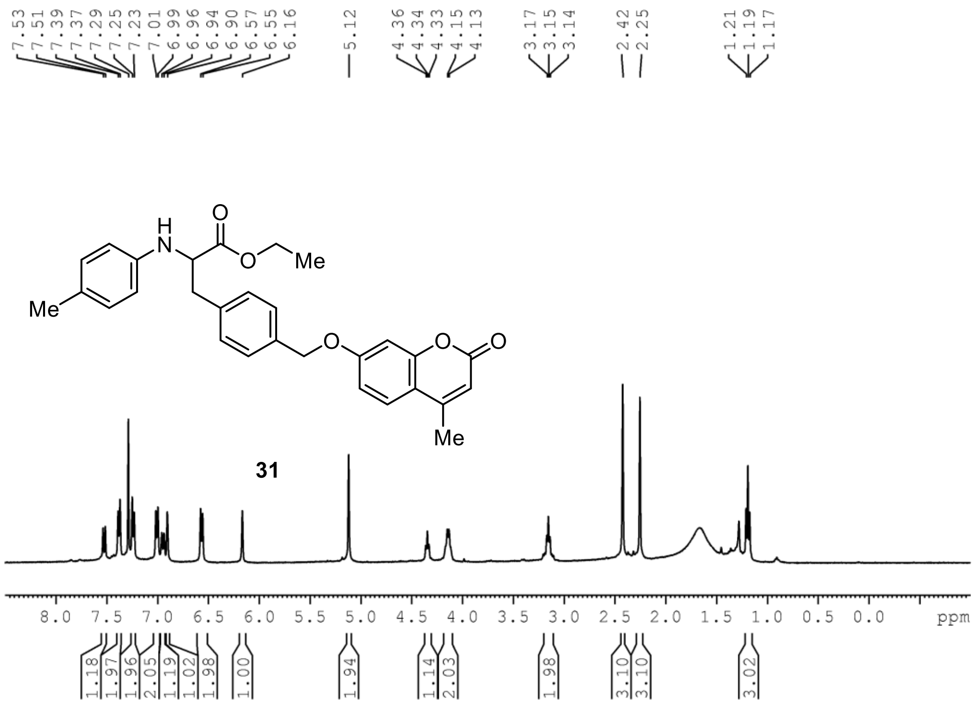

Supplementary Figure 52. ^{1}H NMR spectrum of compound 31

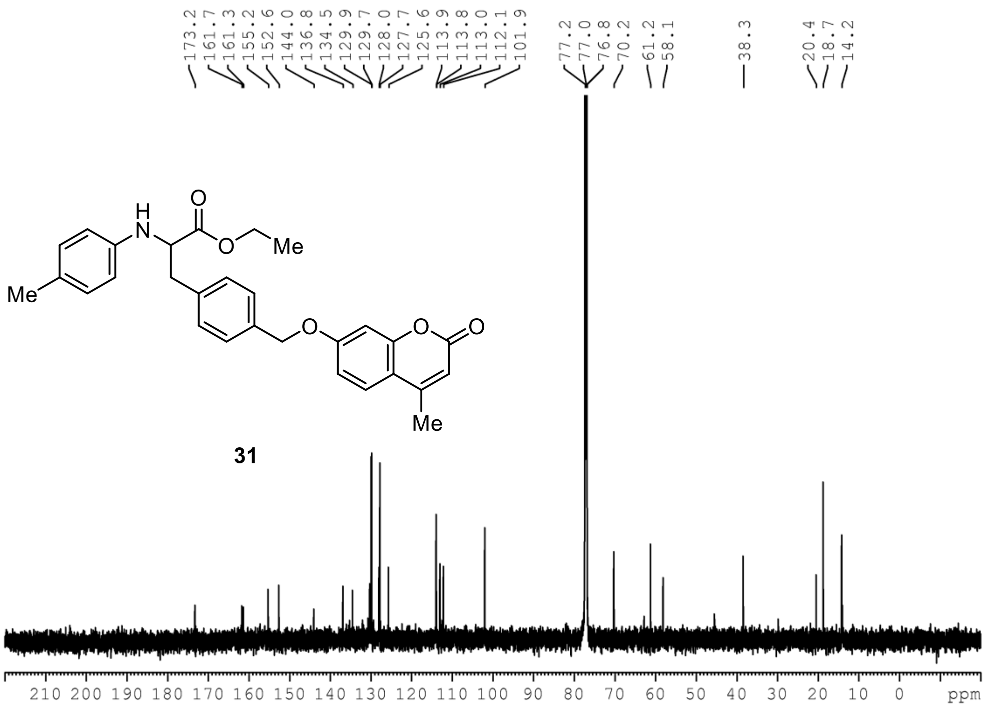

Supplementary Figure 53. ^{13}C NMR spectrum of compound 31

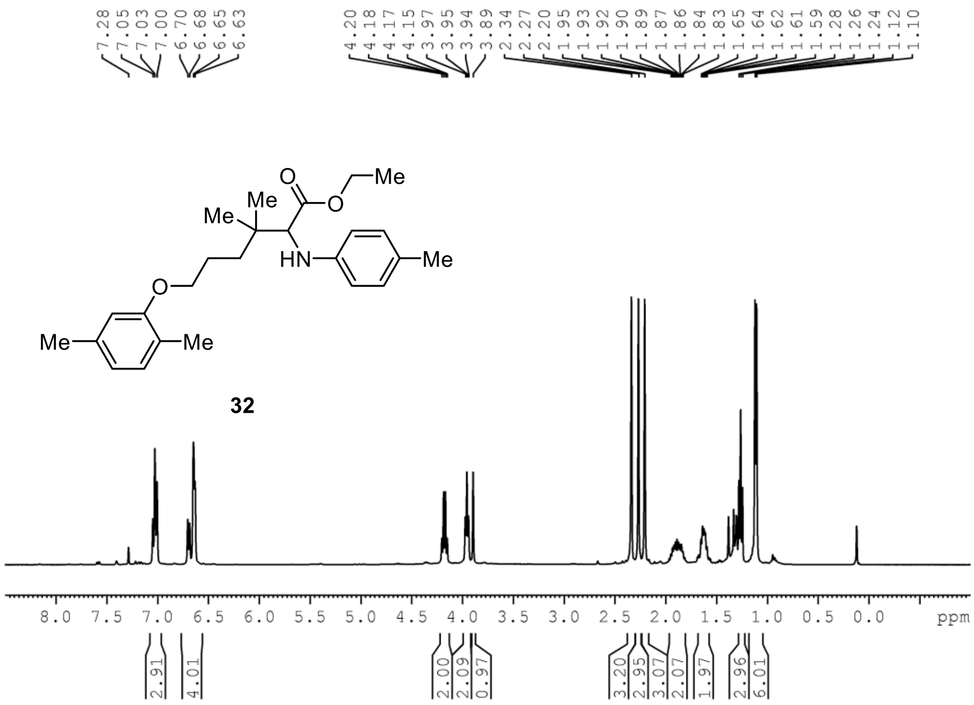

Supplementary Figure 54. ^{1}H NMR spectrum of compound 32

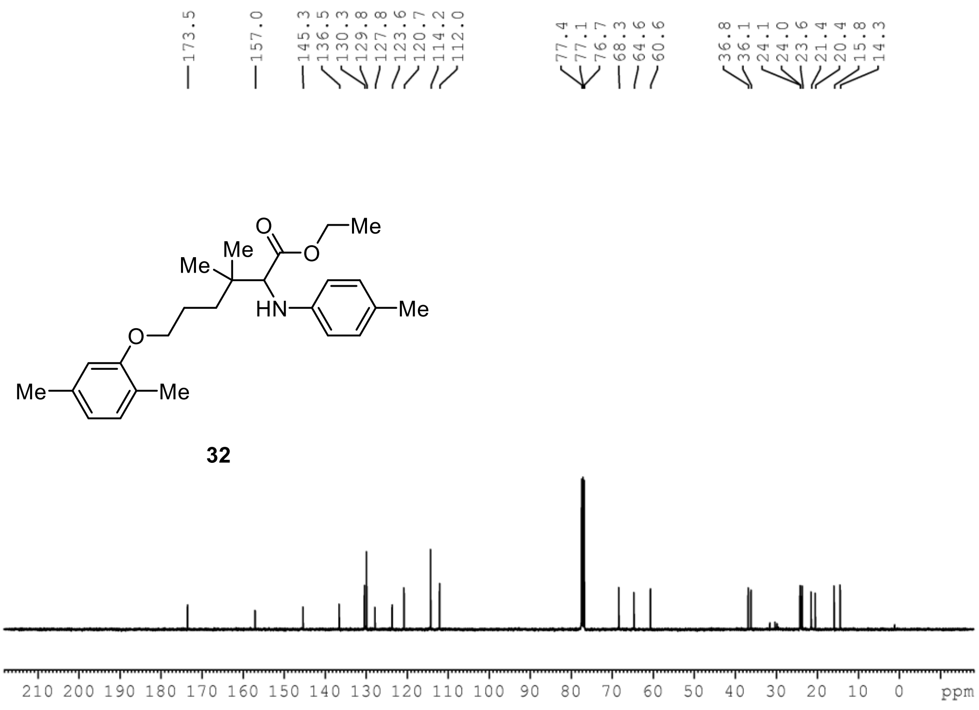

Supplementary Figure 55. ^{13}C NMR spectrum of compound 32

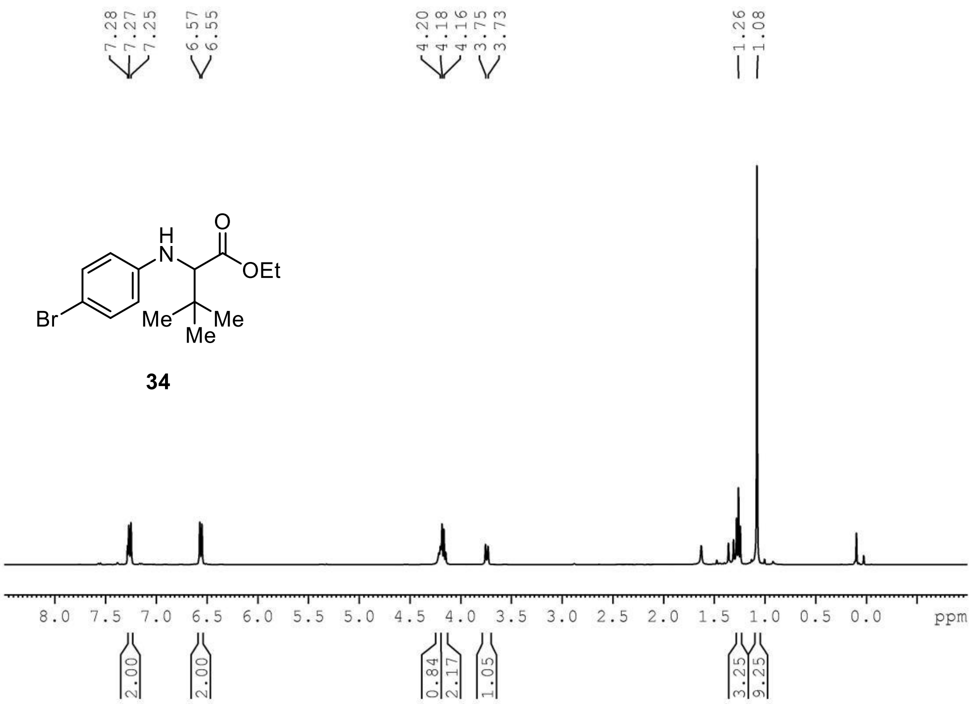

Supplementary Figure 56. ^{1}H NMR spectrum of compound 34

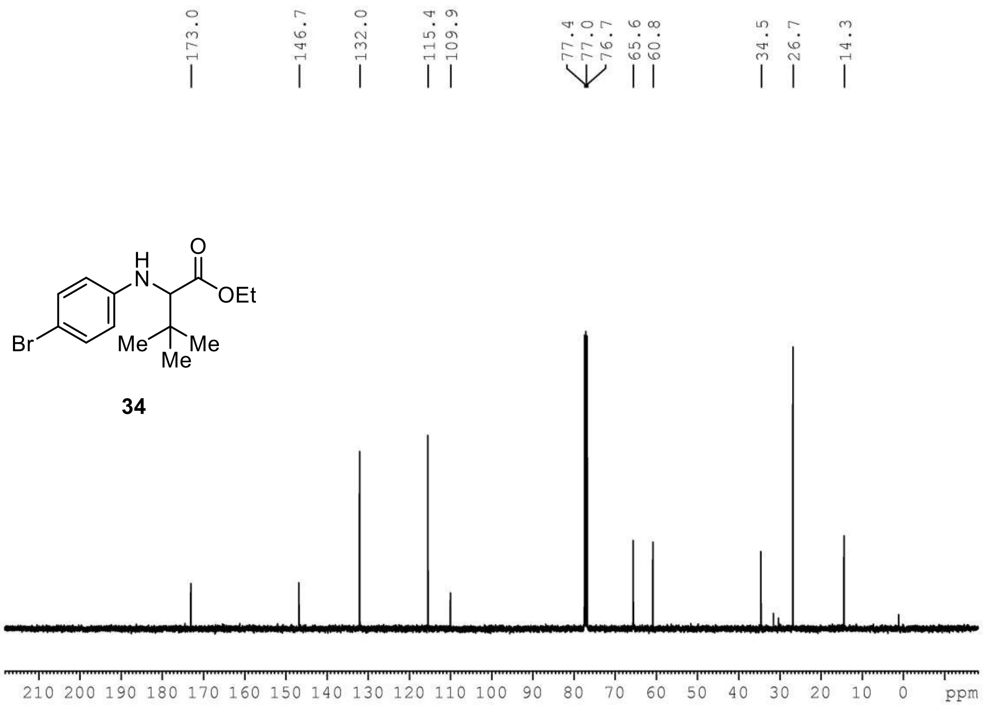

Supplementary Figure 57. ^{13}C NMR spectrum of compound 34

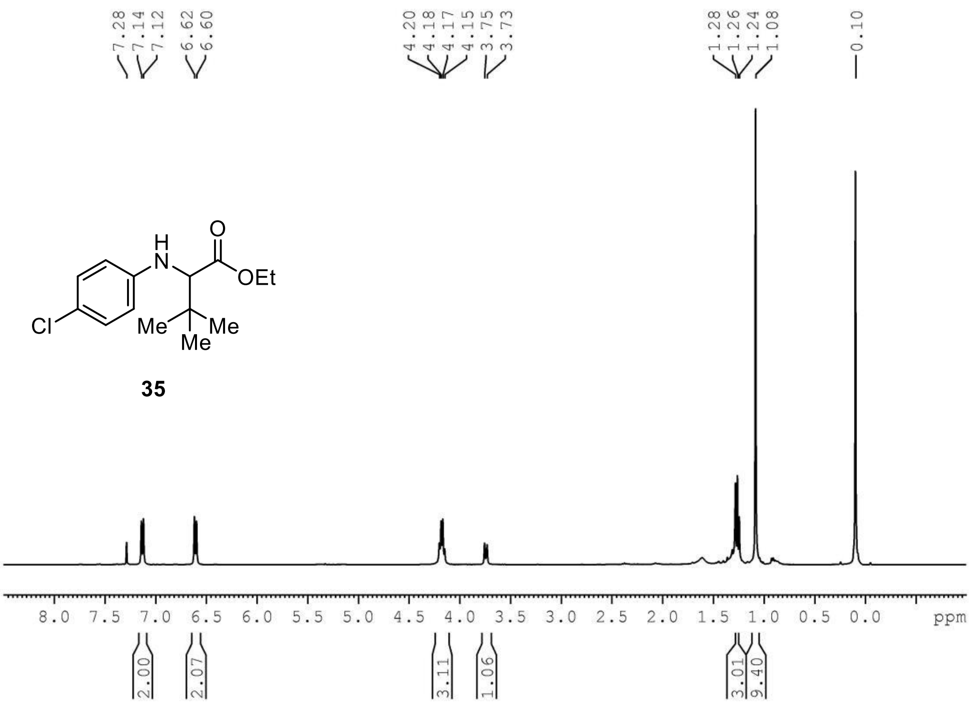

Supplementary Figure 58. ^{1}H NMR spectrum of compound 35

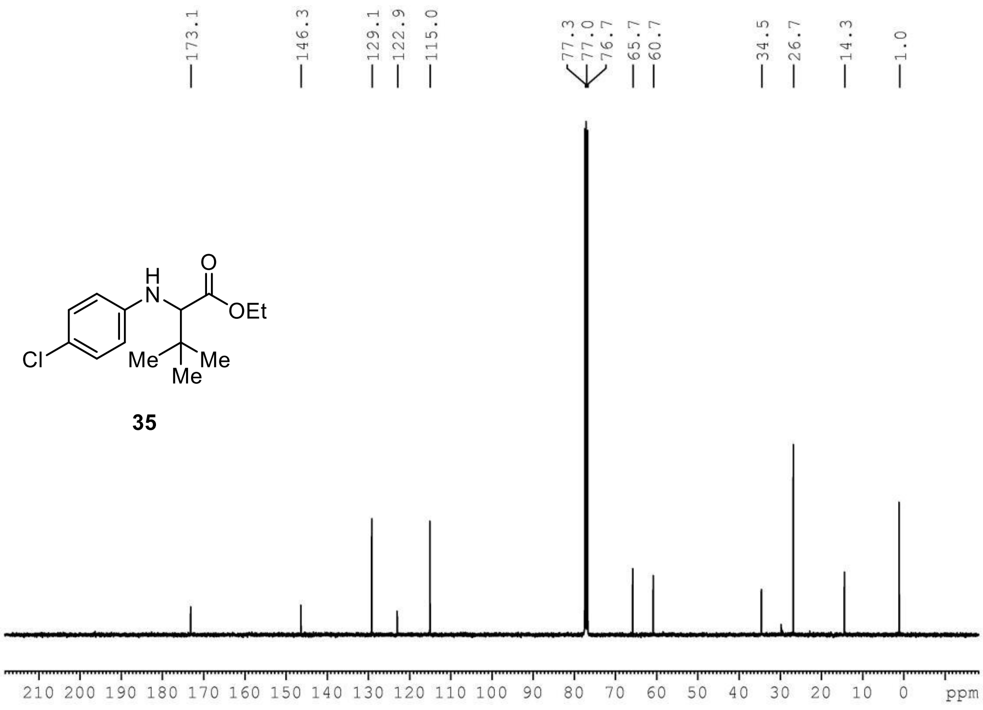

Supplementary Figure 59. ^{13}C NMR spectrum of compound 35

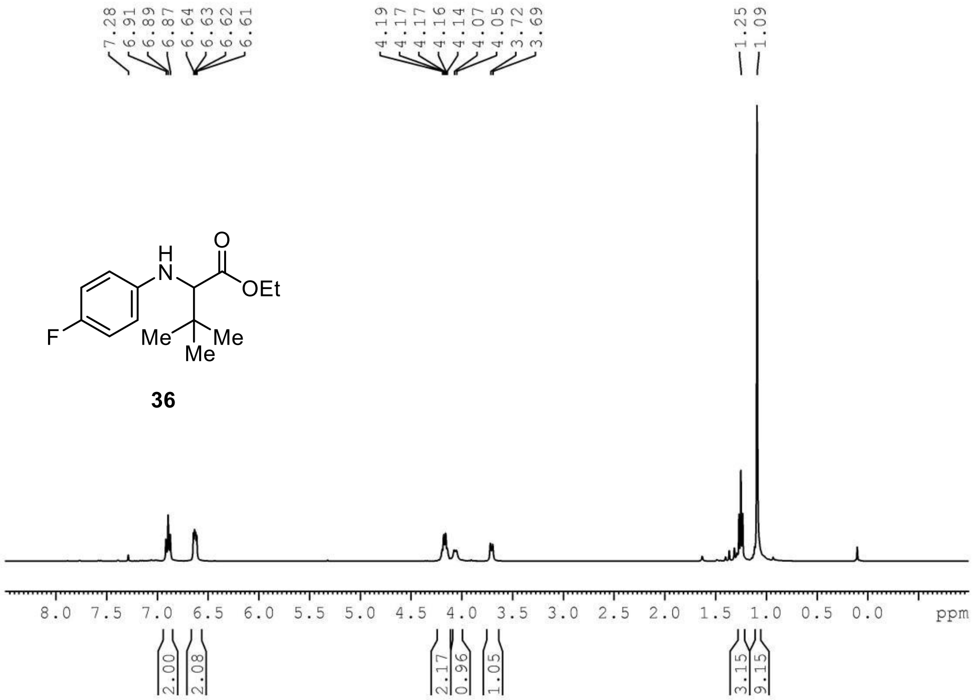

Supplementary Figure 60. ^{1}H NMR spectrum of compound 36

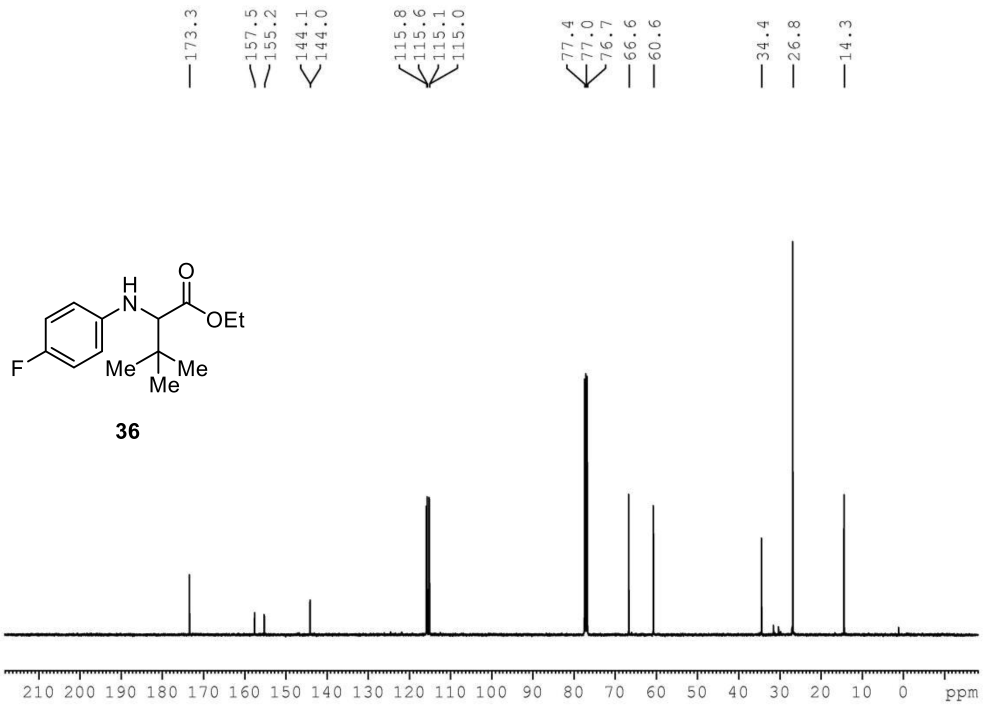

Supplementary Figure 61. ^{13}C NMR spectrum of compound 36

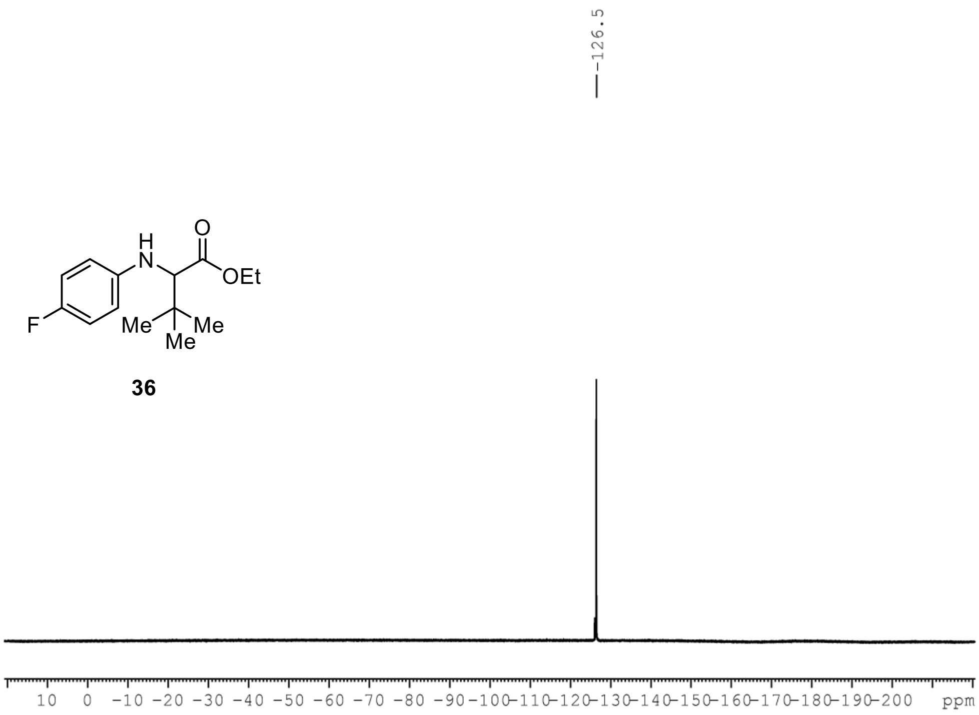

Supplementary Figure 62. ^{19}F NMR spectrum of compound 36

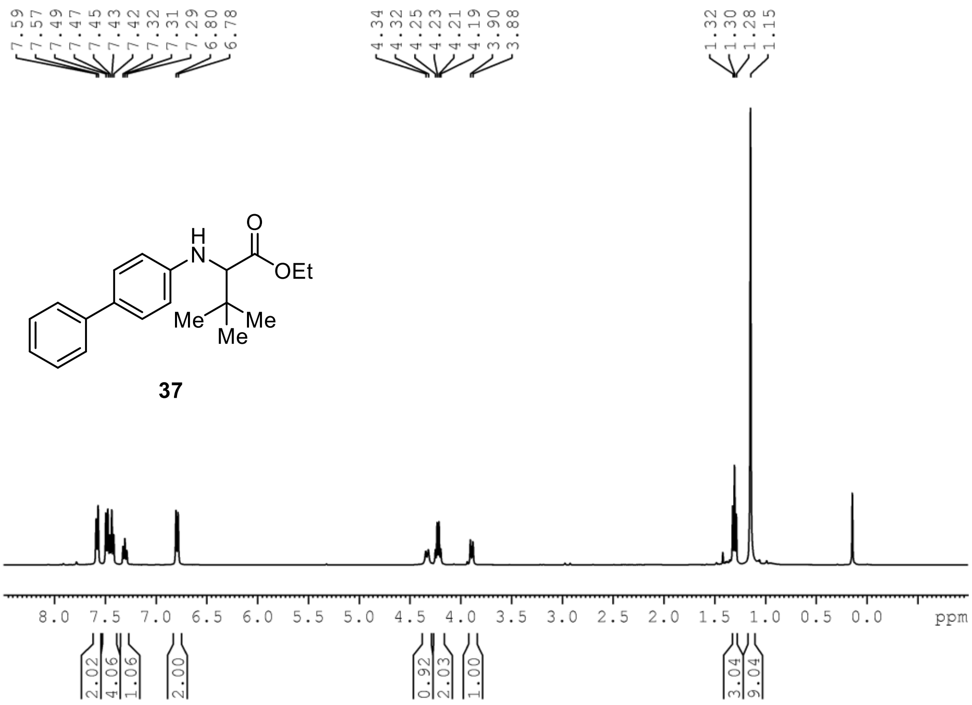

Supplementary Figure 63. ^{1}H NMR spectrum of compound 37

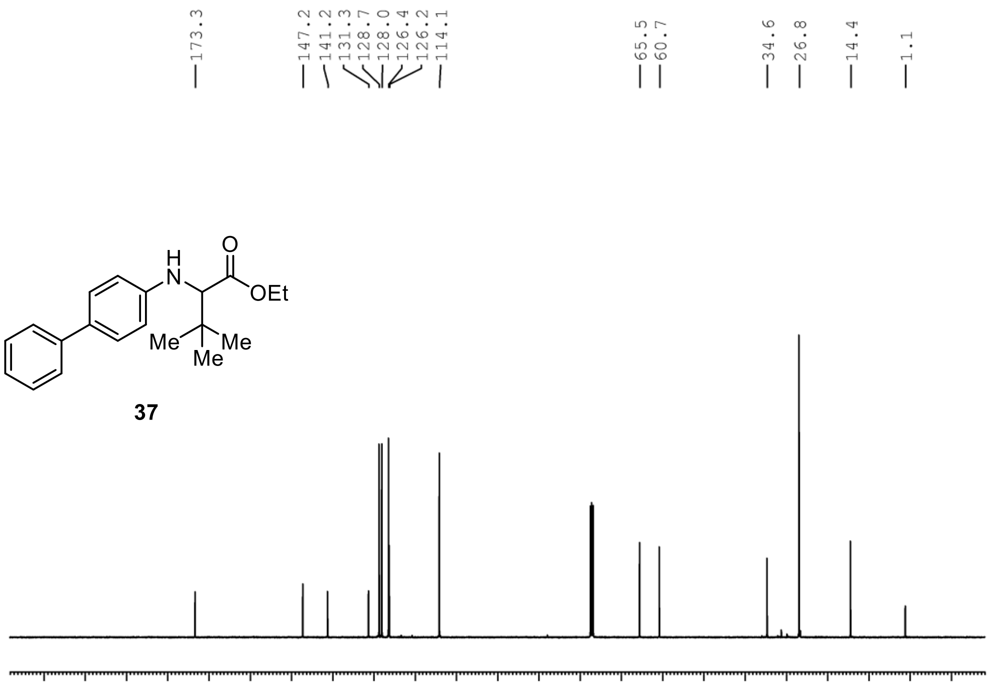

Supplementary Figure 64. ^{13}C NMR spectrum of compound 37

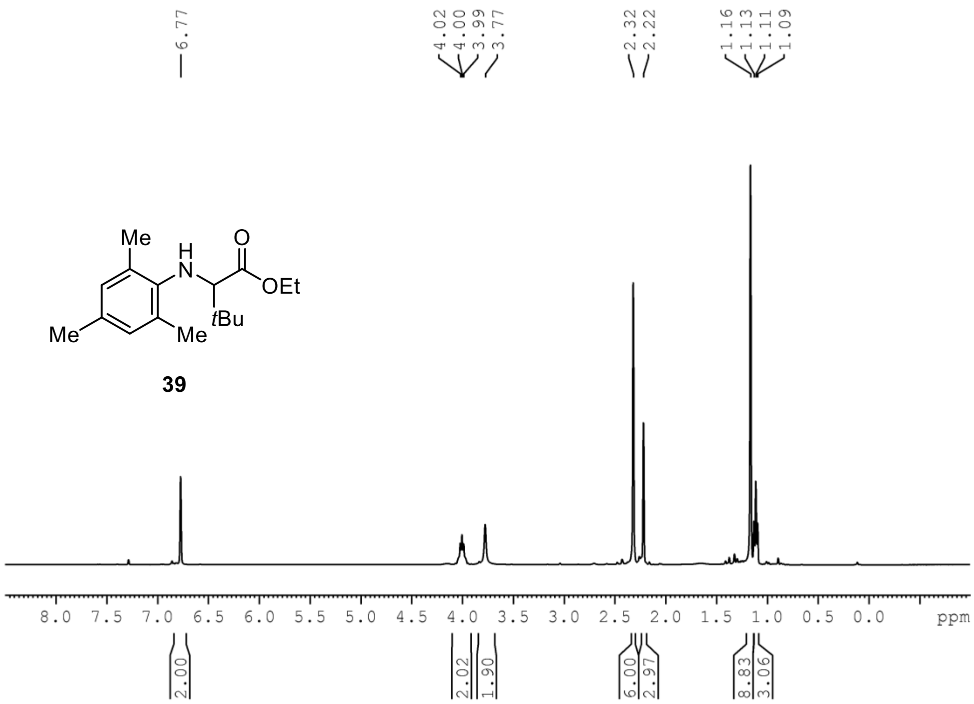

Supplementary Figure 65. ^{1}H NMR spectrum of compound 39

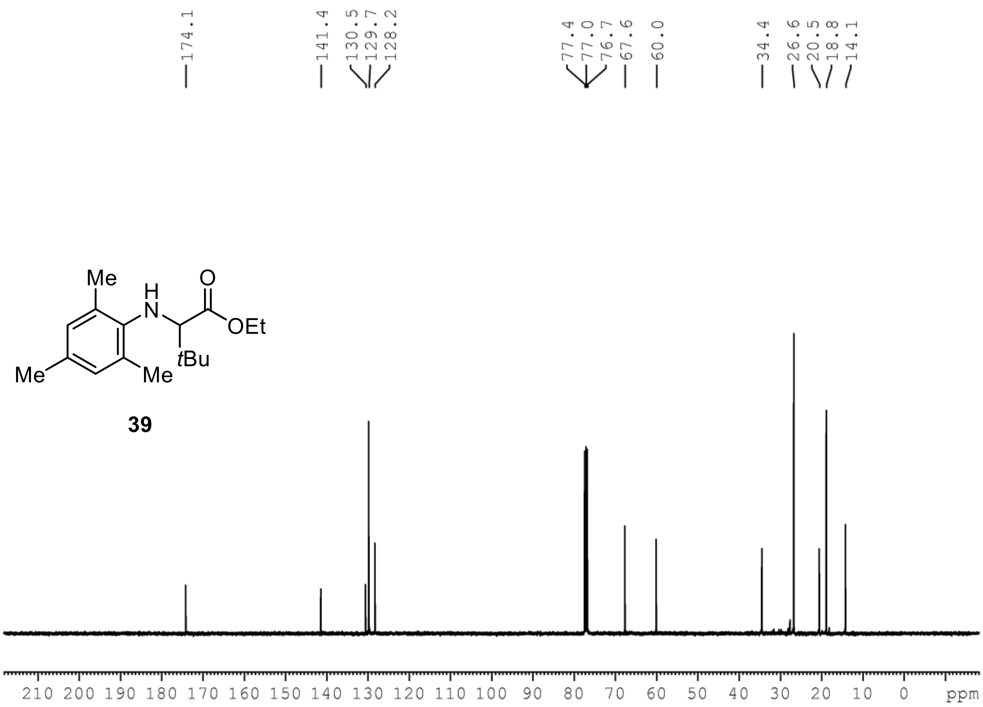

Supplementary Figure 66. ^{13}C NMR spectrum of compound 39

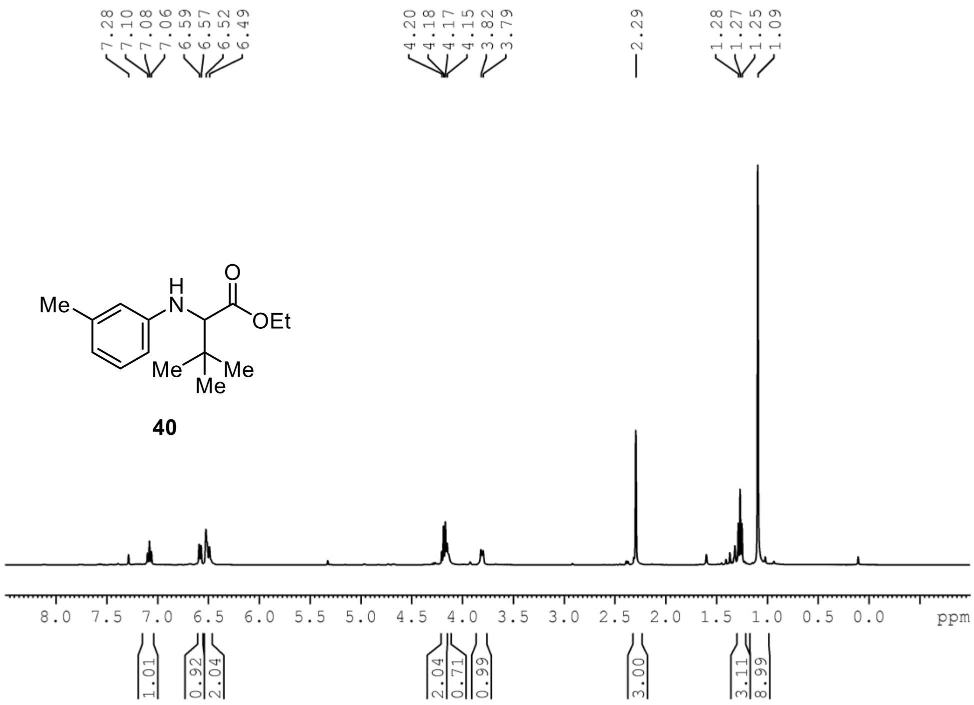

Supplementary Figure 67. ^{1}H NMR spectrum of compound 40

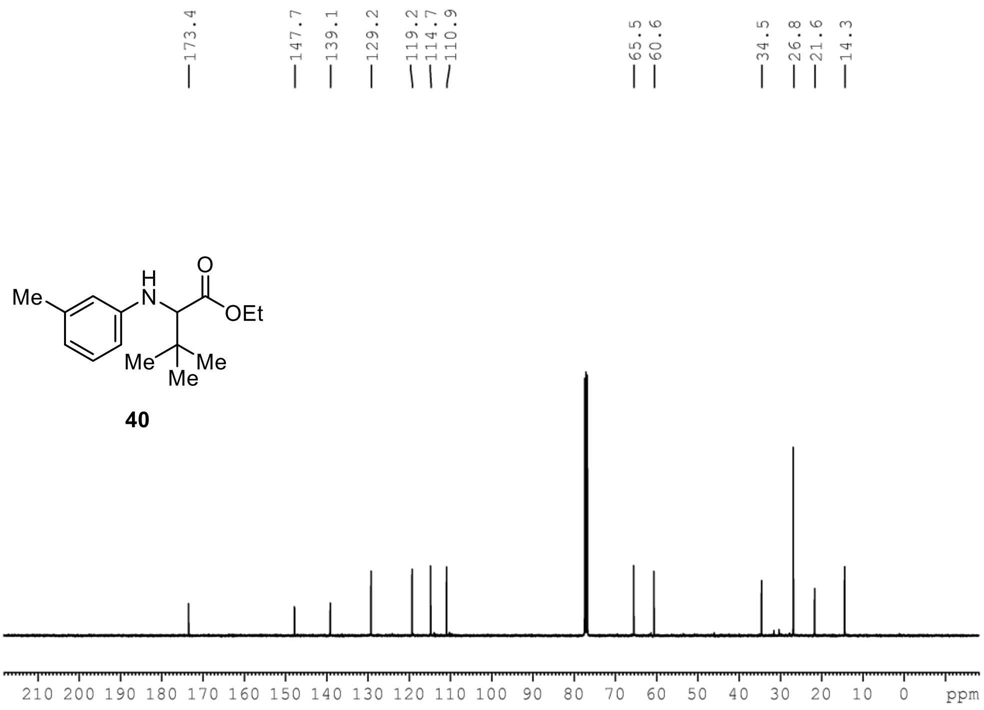

Supplementary Figure 68. ^{13}C NMR spectrum of compound 40

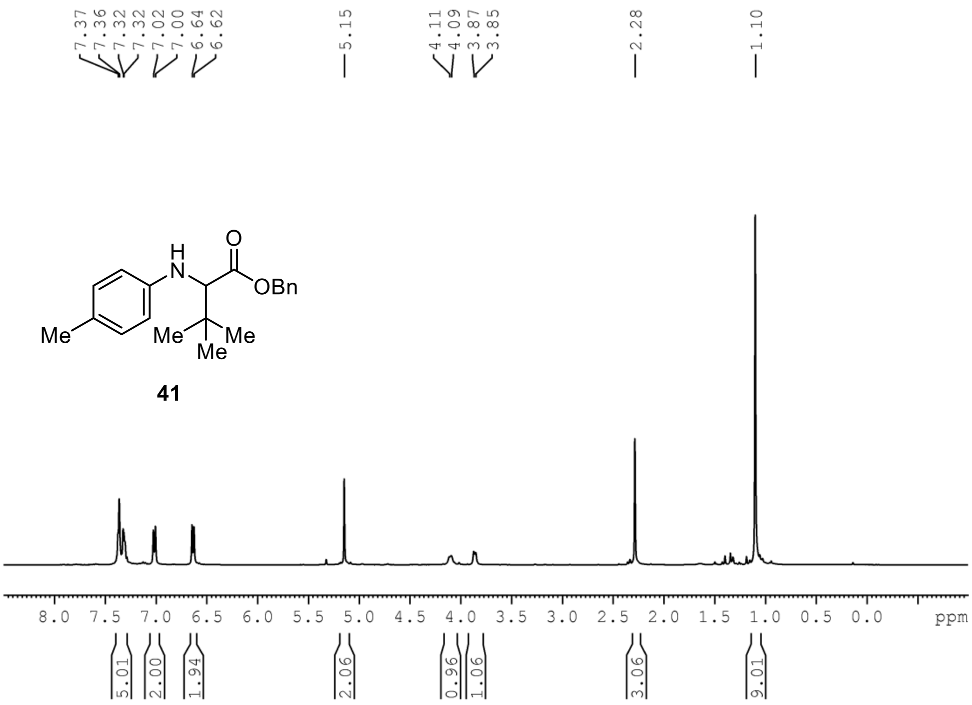

Supplementary Figure 69. ^{1}H NMR spectrum of compound 41

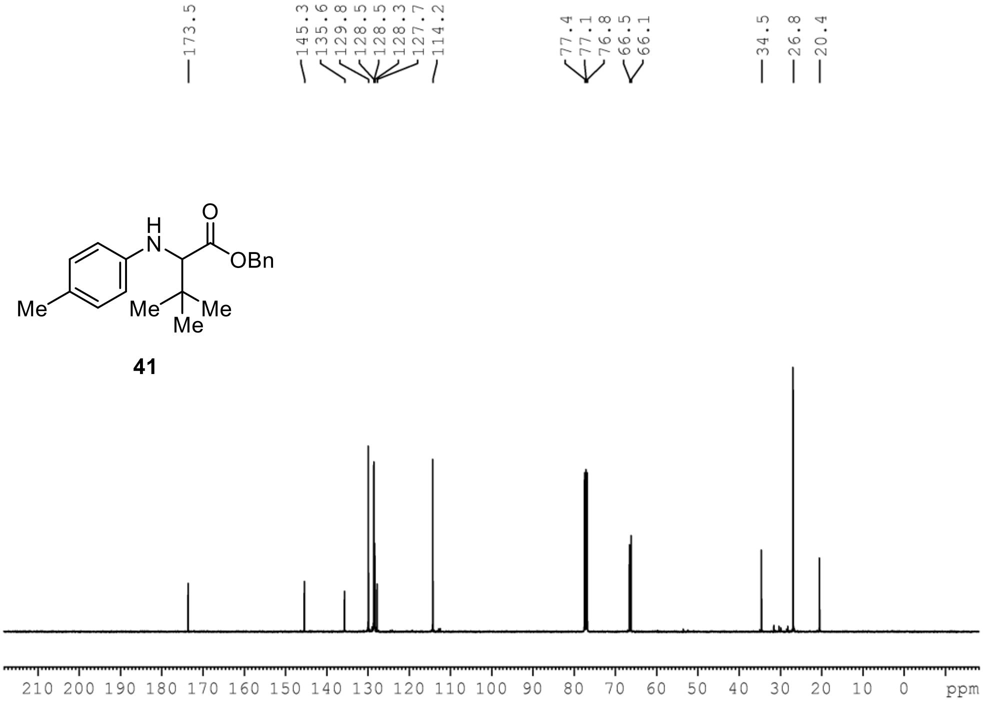

Supplementary Figure 70. ^{13}C NMR spectrum of compound 41

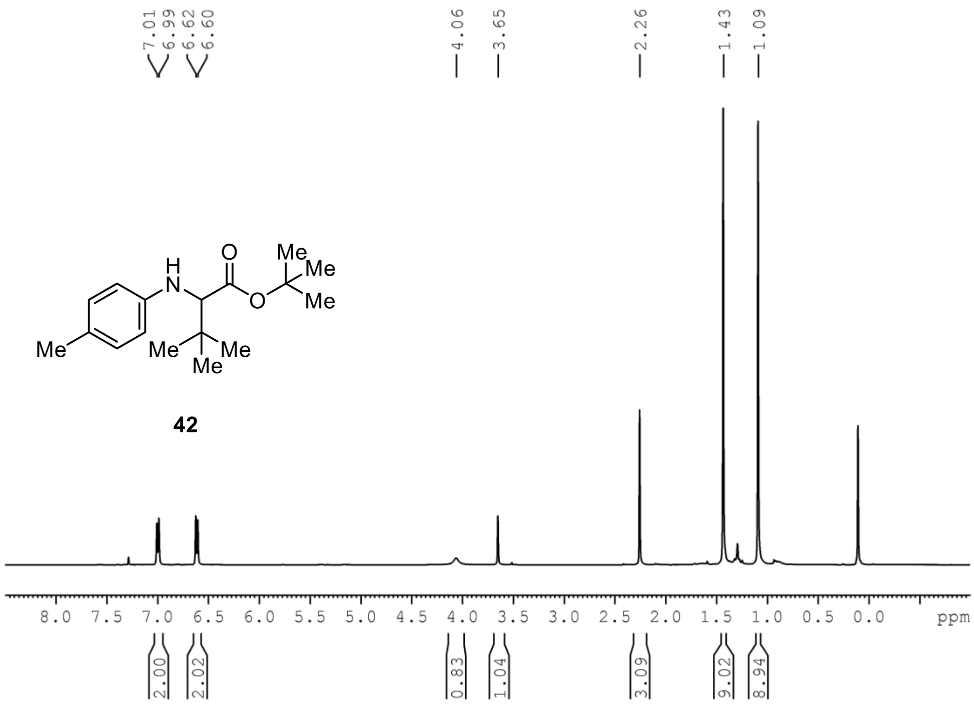

Supplementary Figure 71. ^{1}H NMR spectrum of compound 42

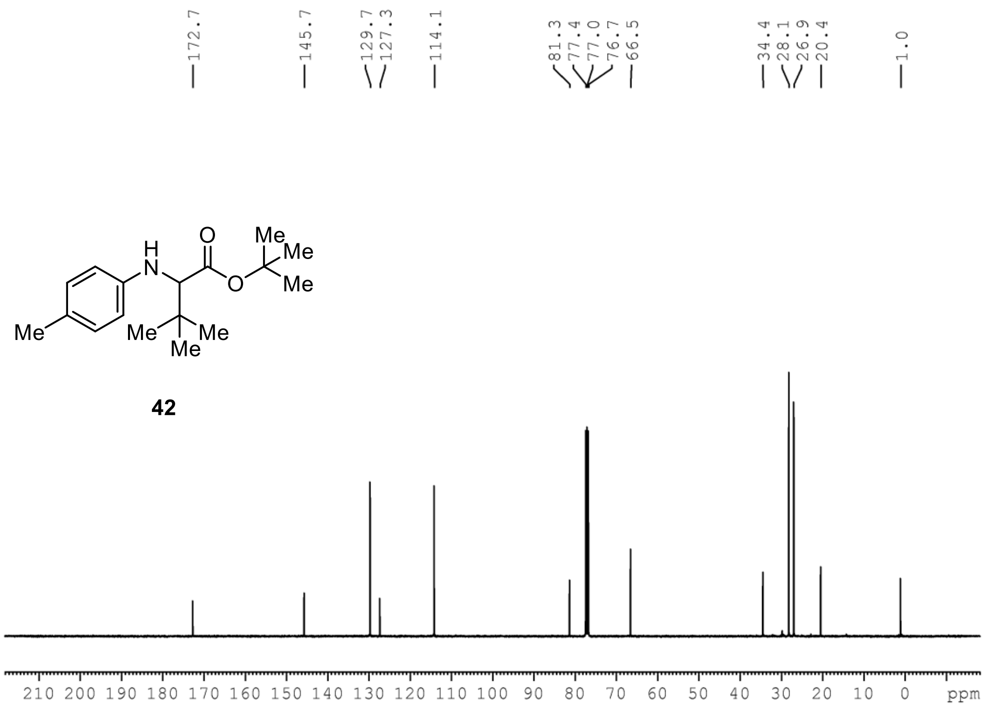

Supplementary Figure 72. ^{13}C NMR spectrum of compound 42

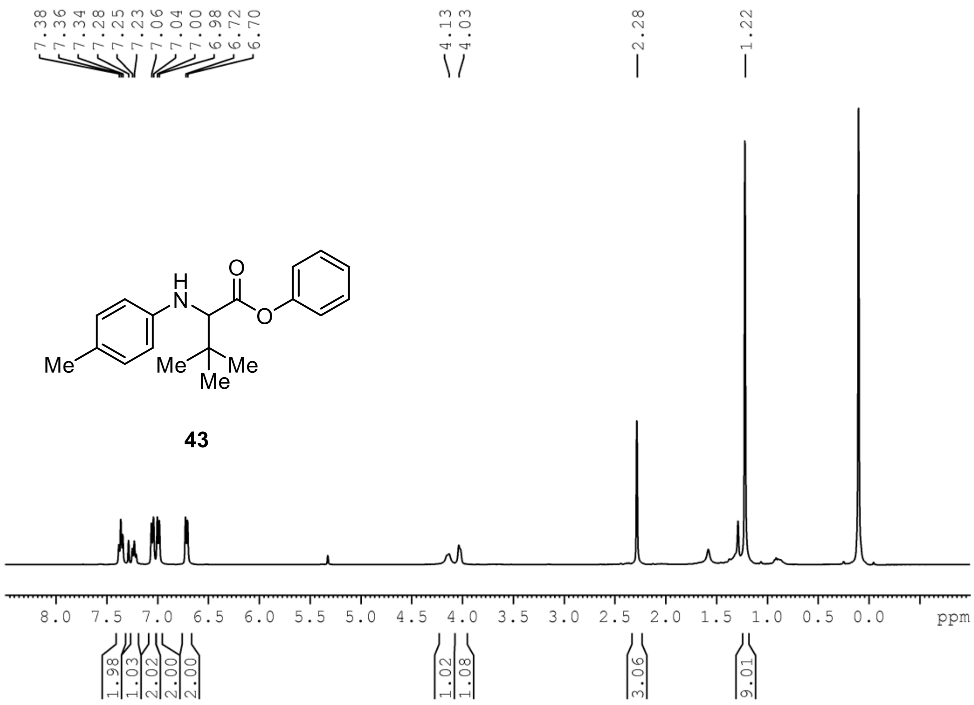

Supplementary Figure 73. ^{1}H NMR spectrum of compound 43

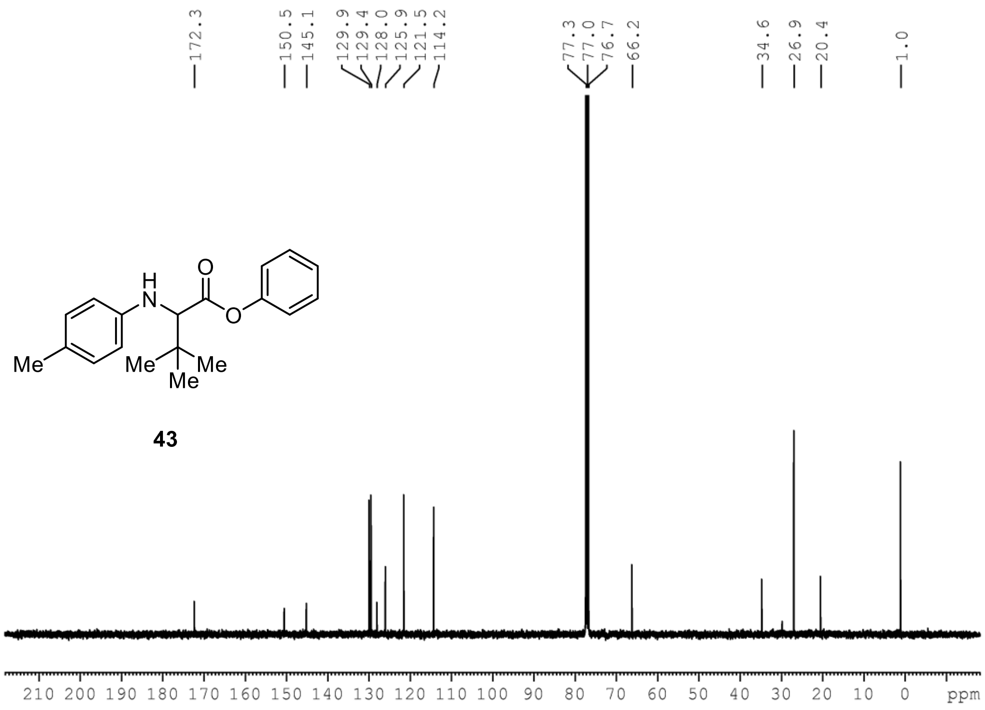

Supplementary Figure 74. ^{13}C NMR spectrum of compound 43

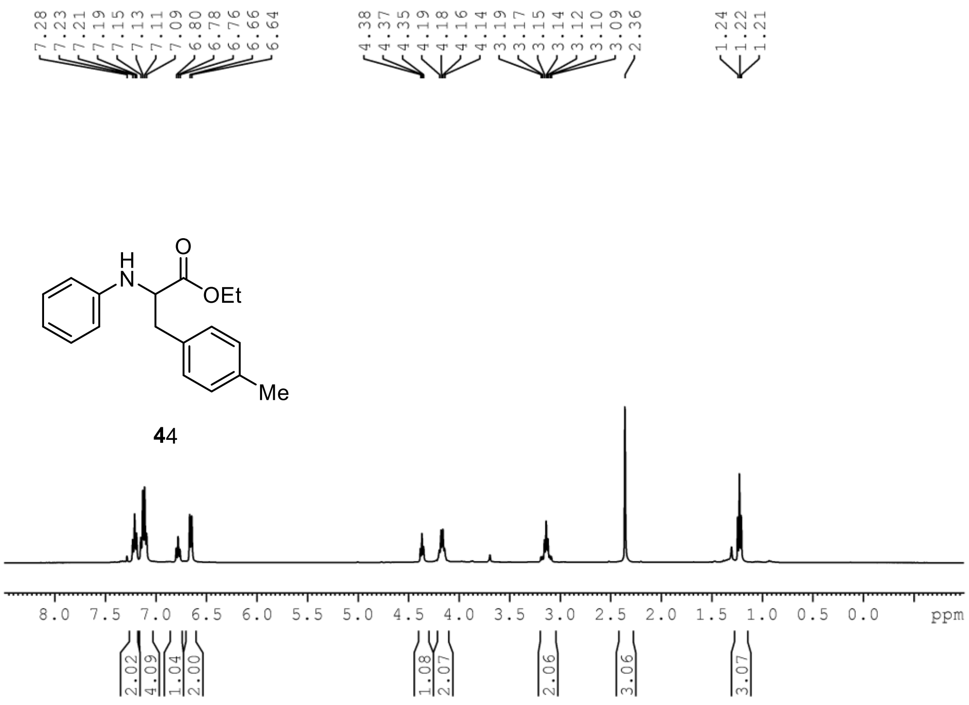

Supplementary Figure 75. ^{1}H NMR spectrum of compound 44

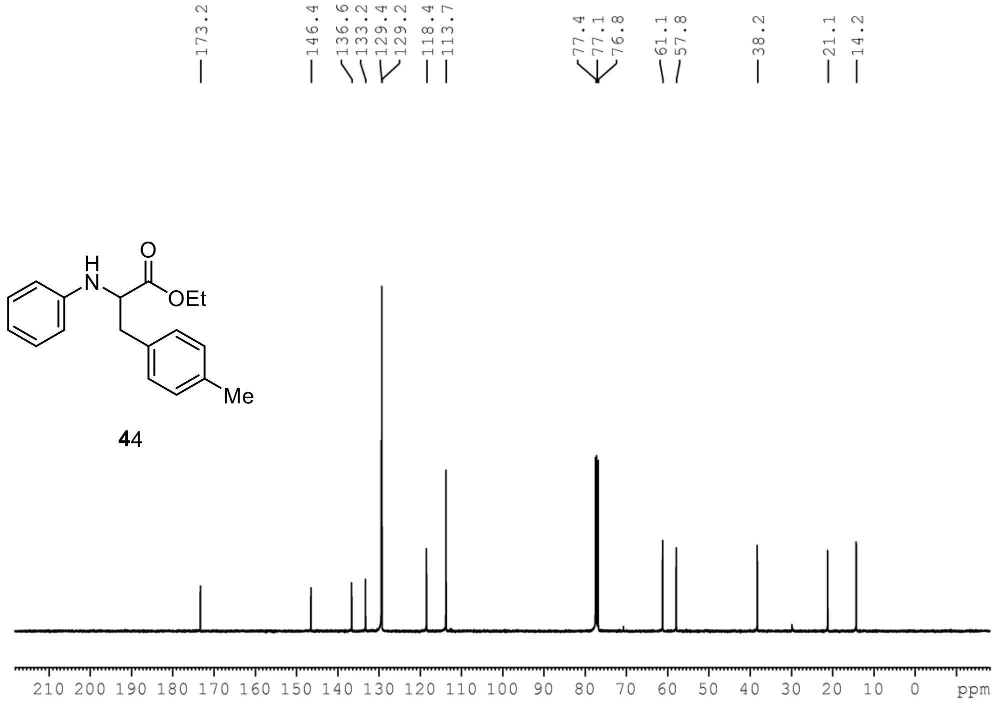

Supplementary Figure 76. ^{13}C NMR spectrum of compound 44

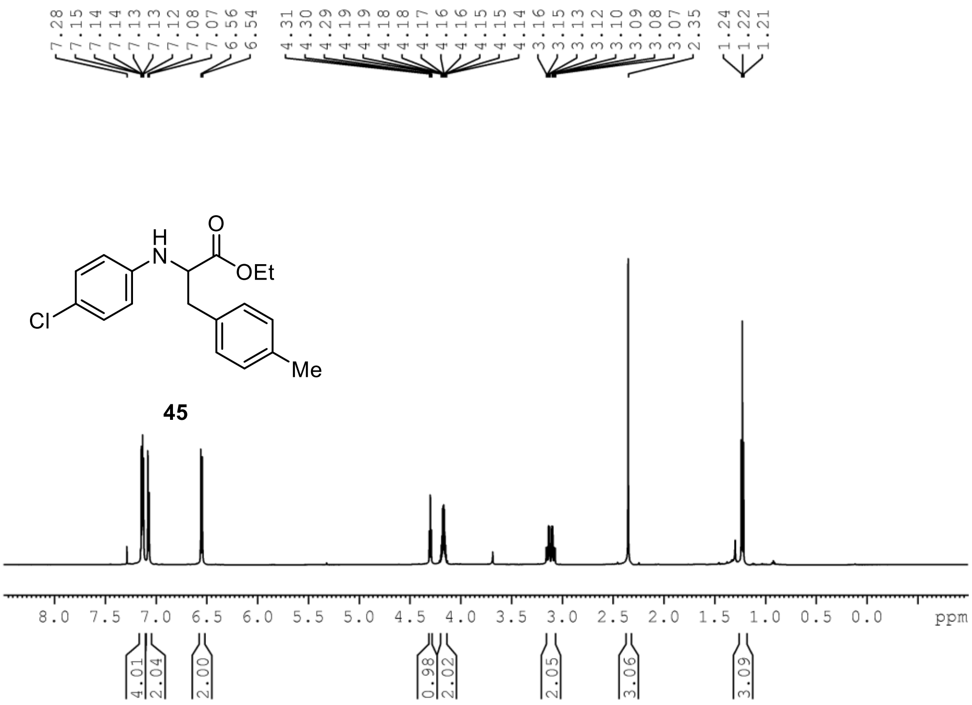

Supplementary Figure 77. ^{1}H NMR spectrum of compound 45

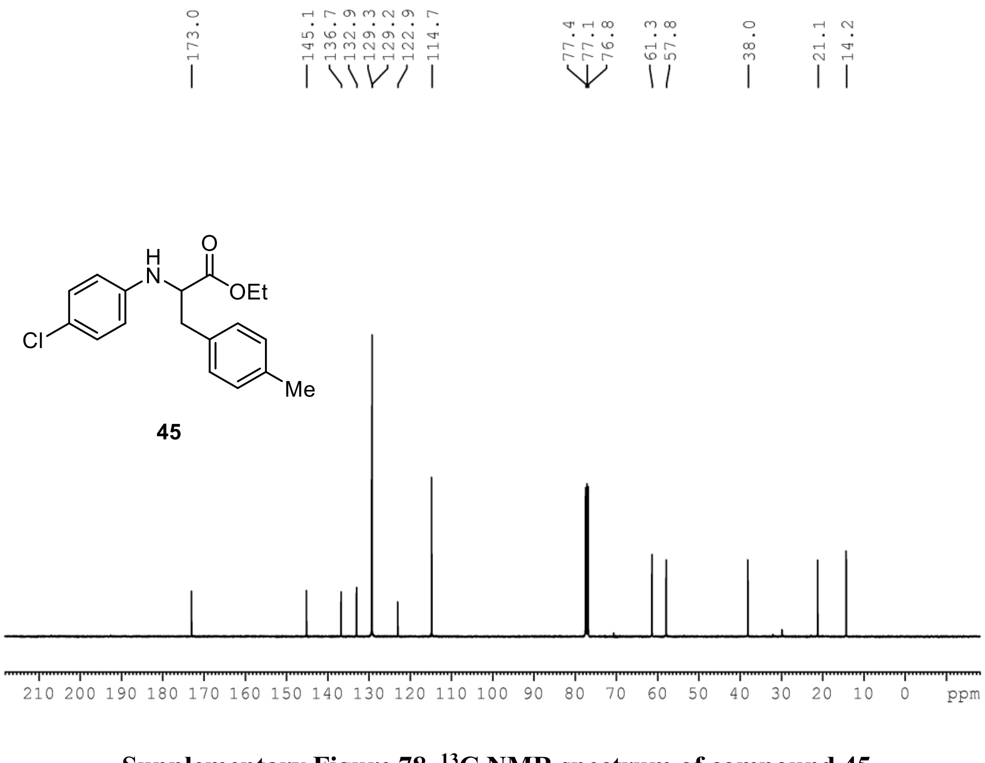

Supplementary Figure 78. ^{13}C NMR spectrum of compound 45

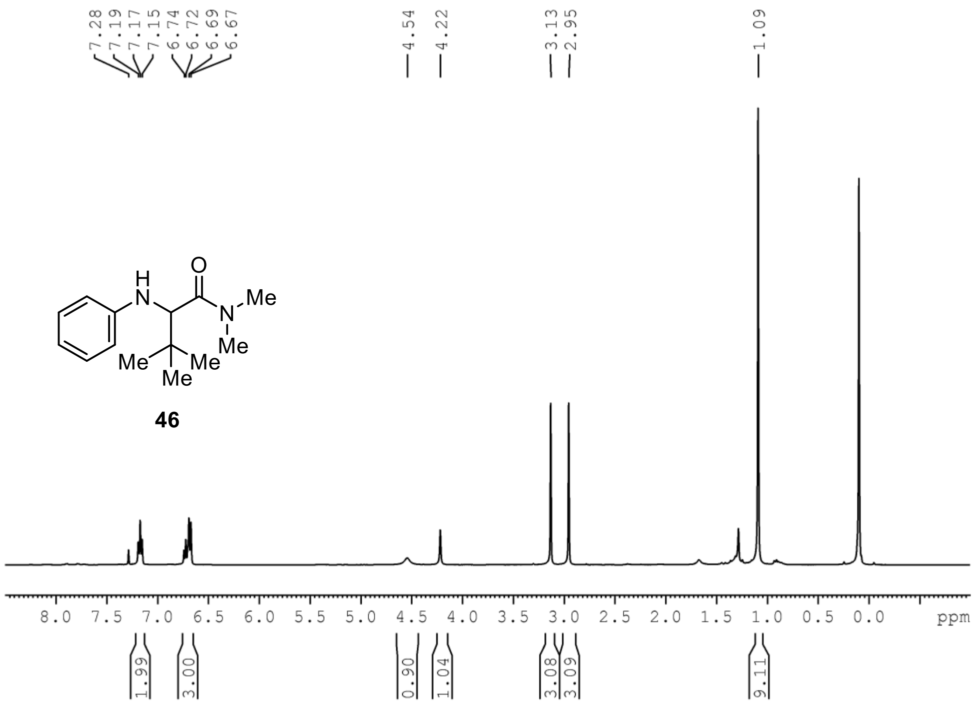

Supplementary Figure 79. ^{1}H NMR spectrum of compound 46

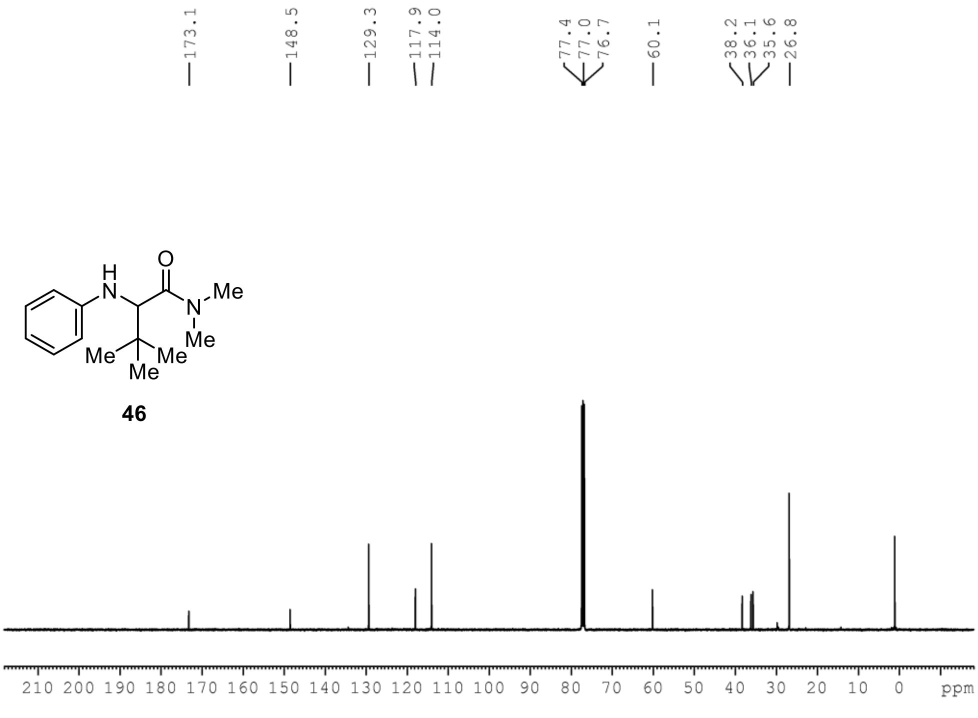

Supplementary Figure 80. ^{13}C NMR spectrum of compound 46

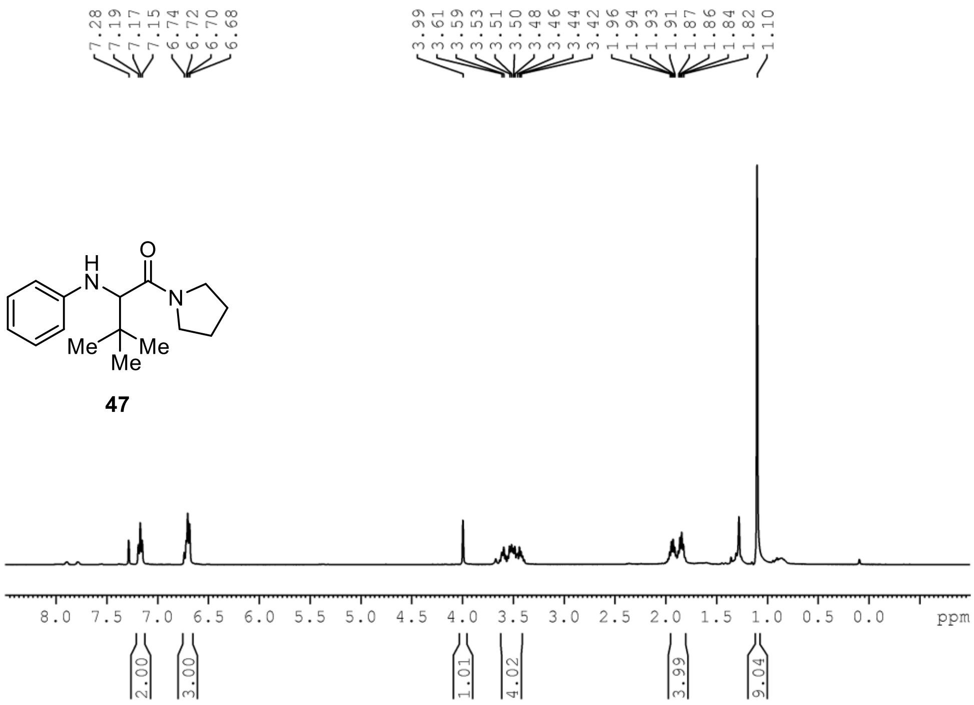

Supplementary Figure 81. ^{1}H NMR spectrum of compound 47

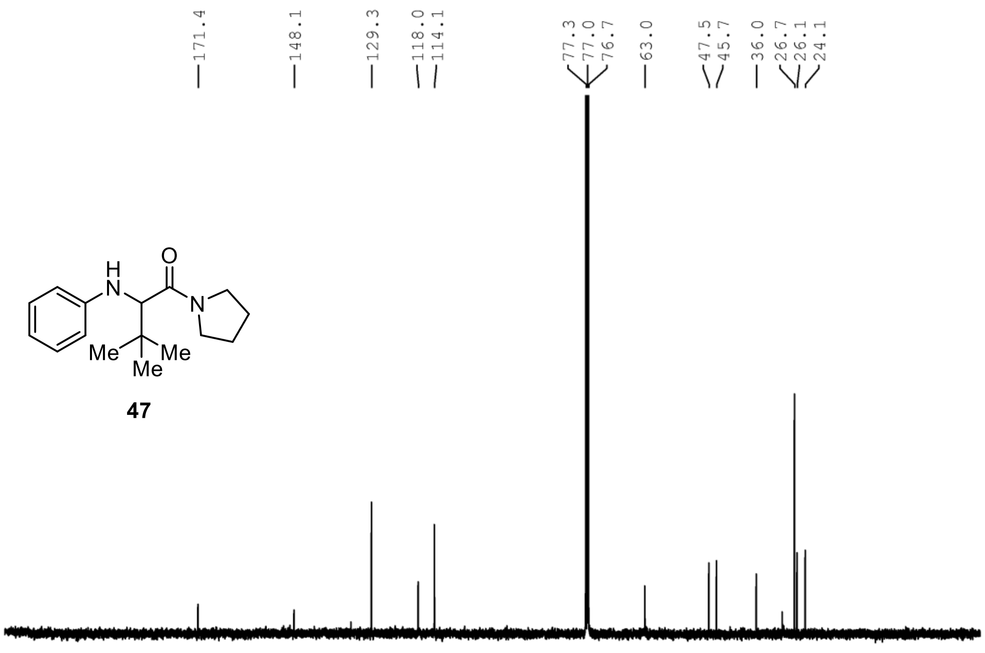

Supplementary Figure 82. ^{13}C NMR spectrum of compound 47

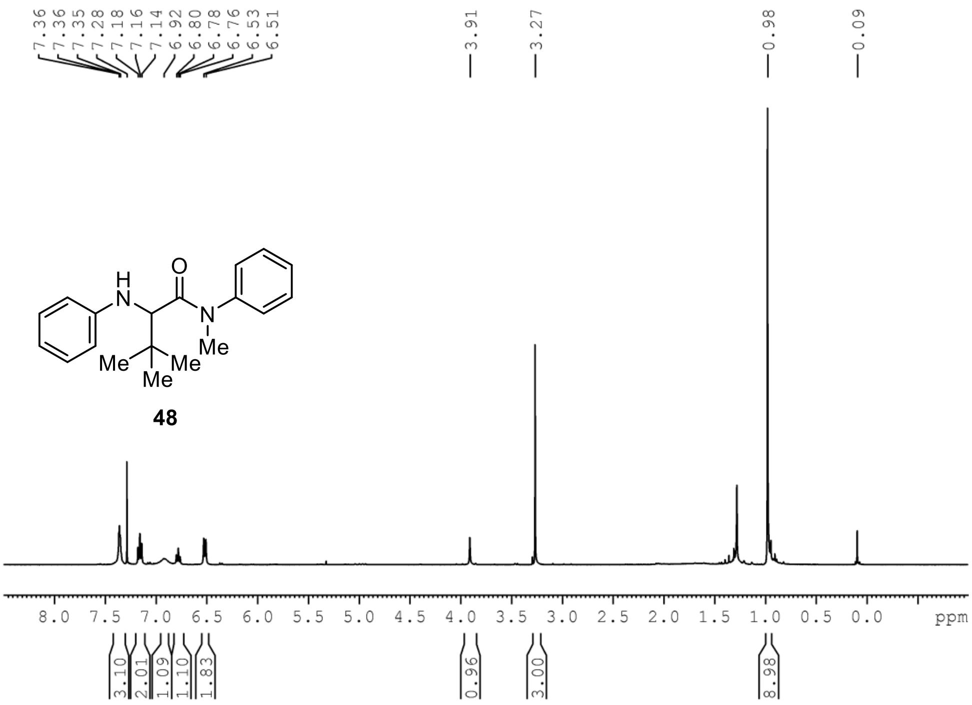

Supplementary Figure 83. ^{1}H NMR spectrum of compound 48

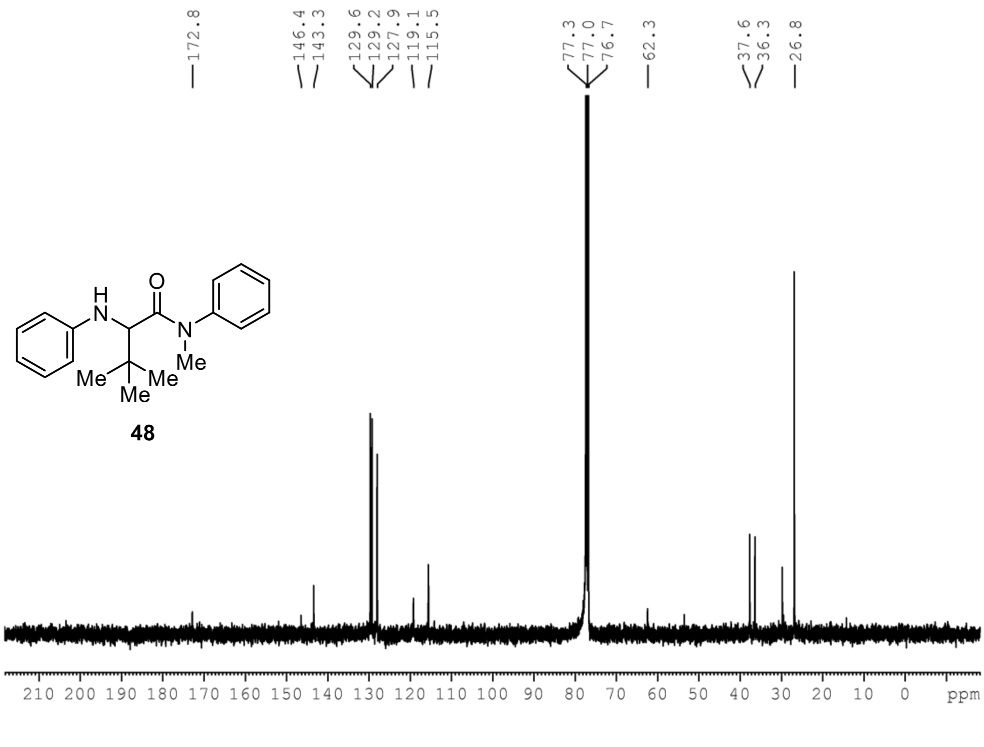

Supplementary Figure 84. ^{13}C NMR spectrum of compound 48

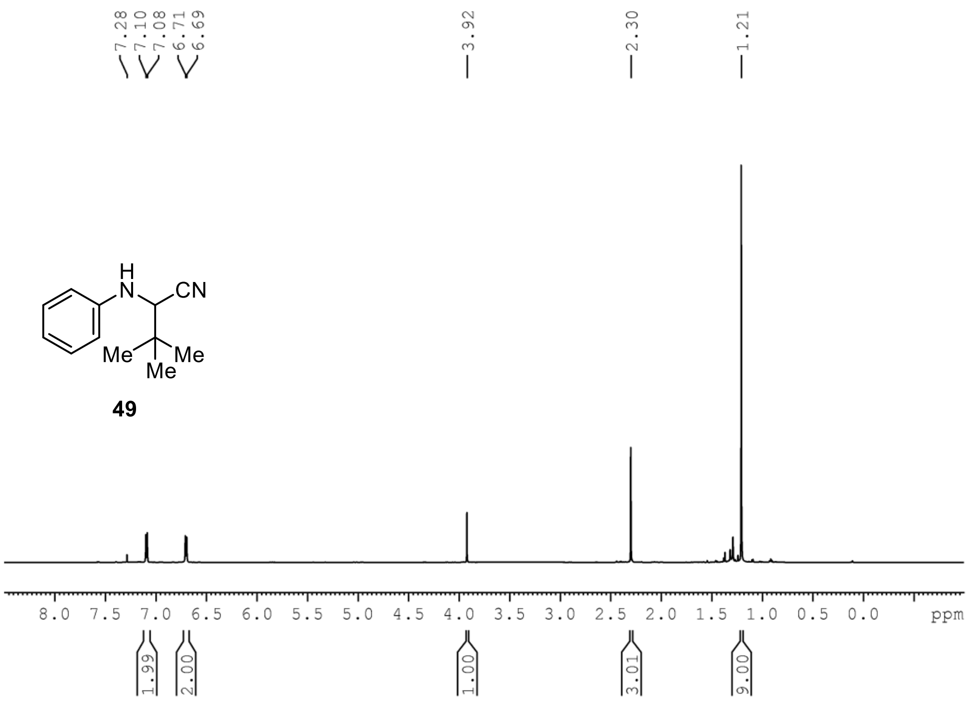

Supplementary Figure 85. ^{1}H NMR spectrum of compound 49

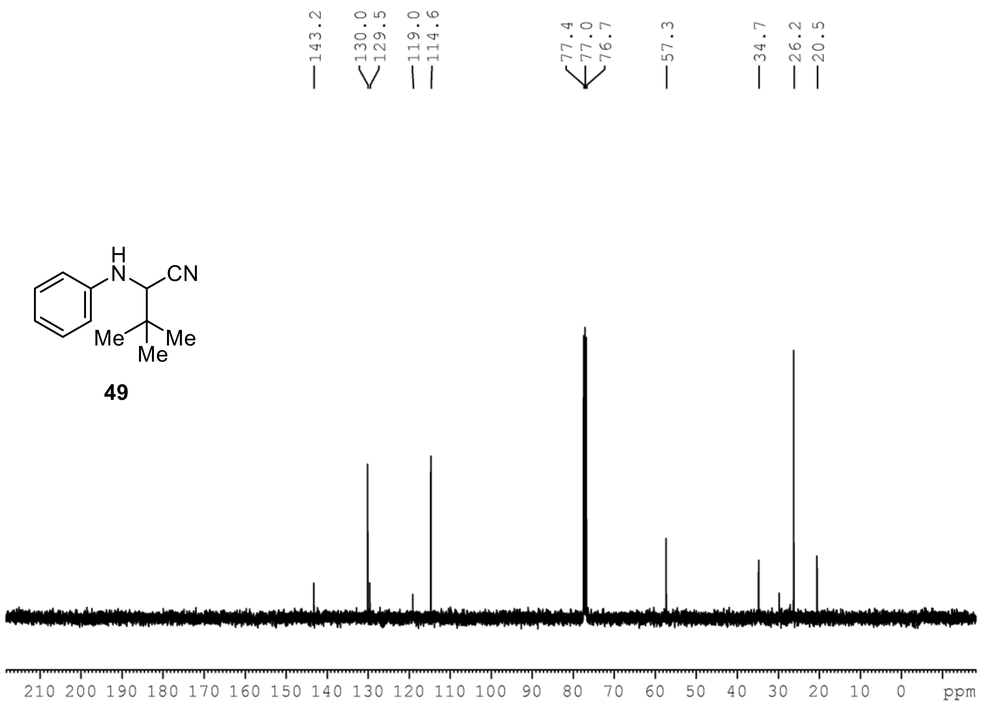

Supplementary Figure 86. ^{13}C NMR spectrum of compound 49

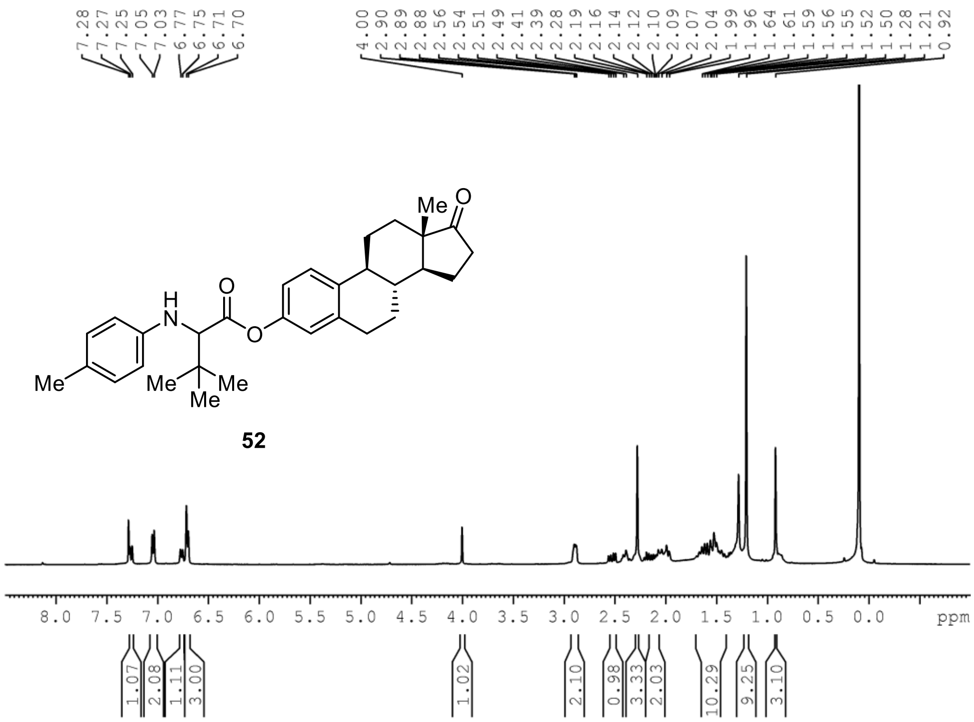

Supplementary Figure 87. ^{1}H NMR spectrum of compound 52

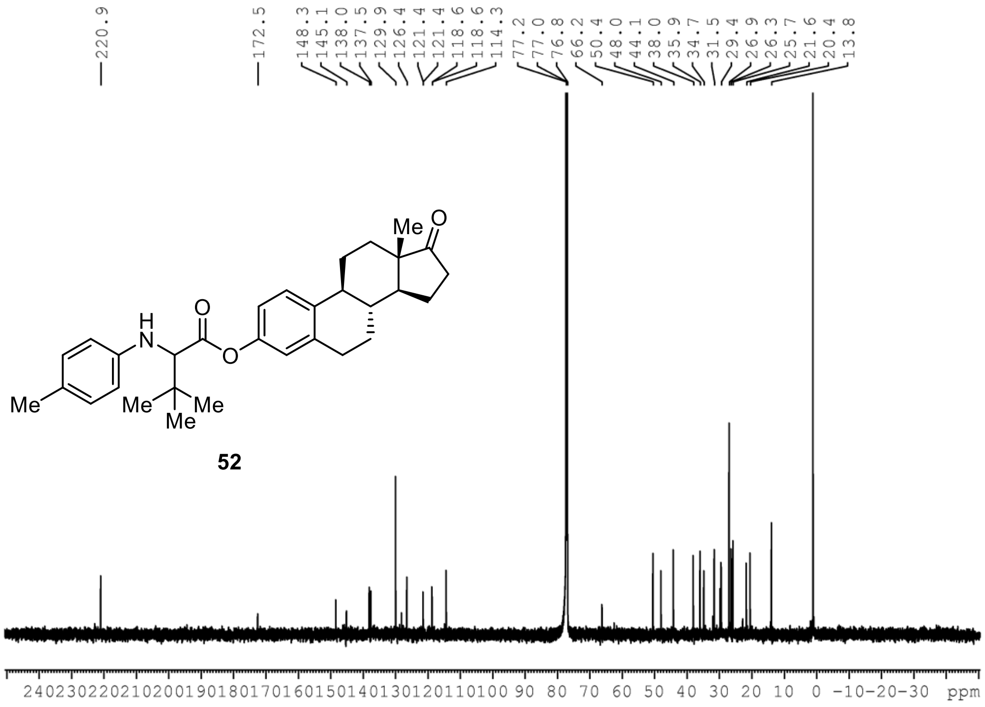

Supplementary Figure 88. ^{13}C NMR spectrum of compound 52

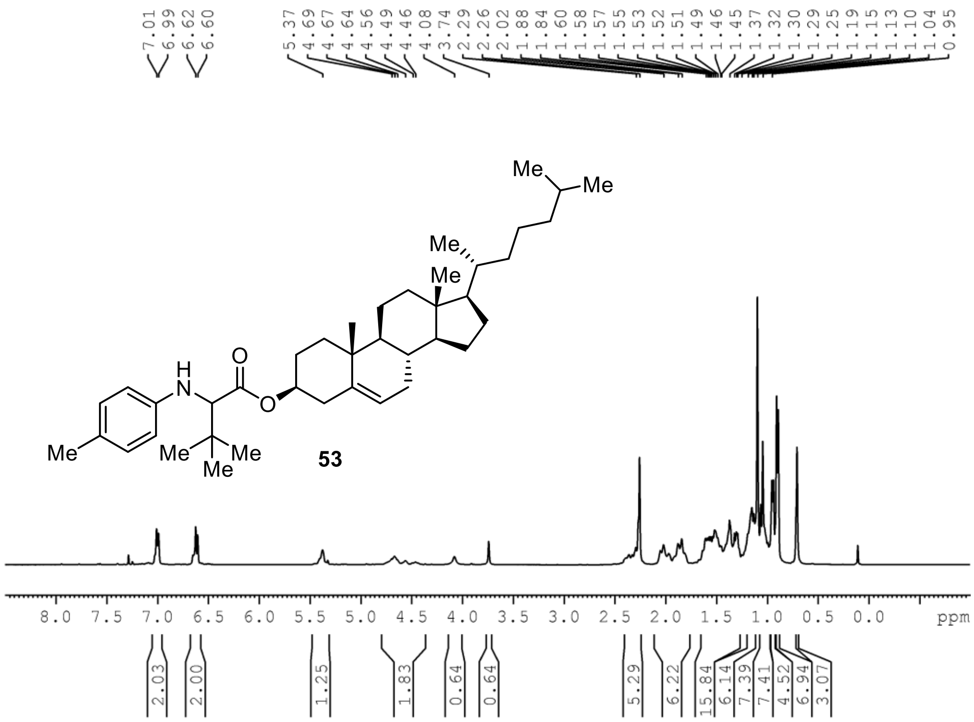

Supplementary Figure 89. ^{1}H NMR spectrum of compound 53

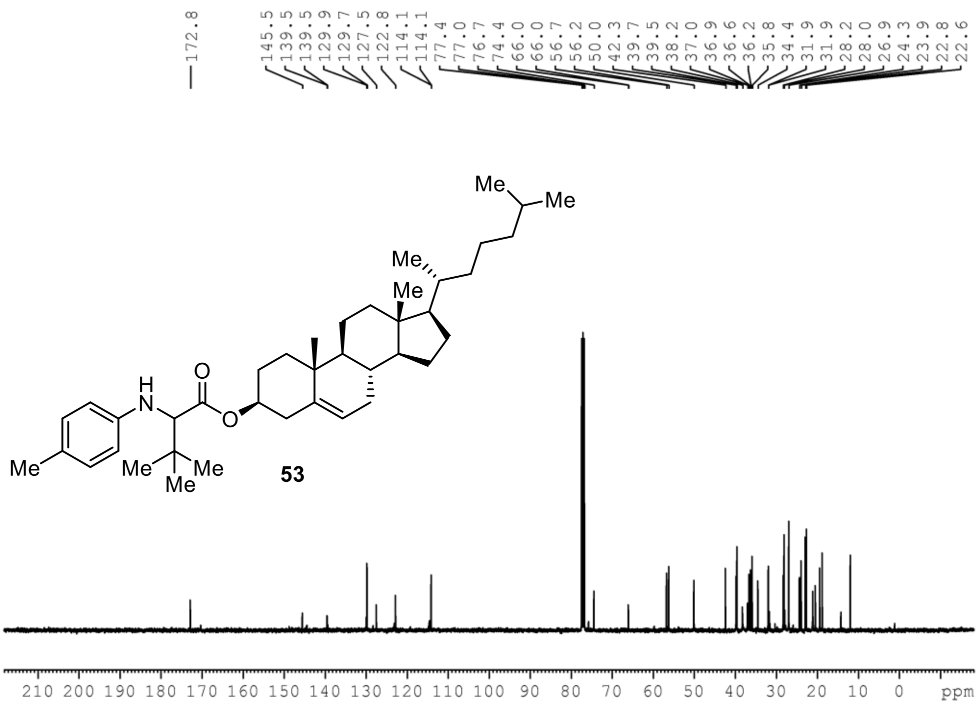

Supplementary Figure 90. ^{13}C NMR spectrum of compound 53

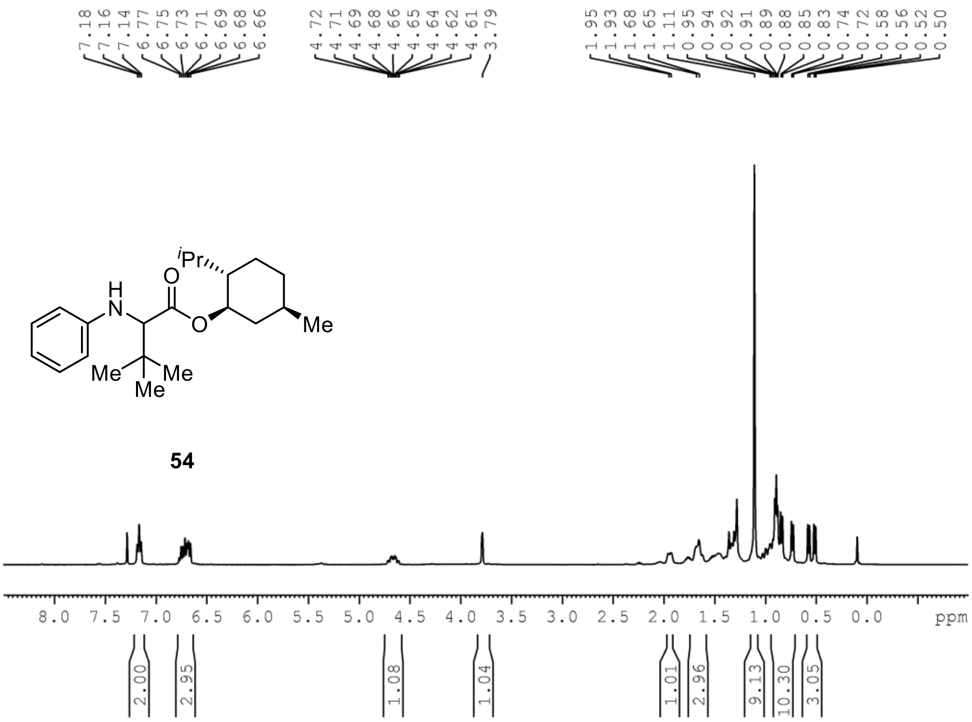

Supplementary Figure 91. ^{1}H NMR spectrum of compound 54

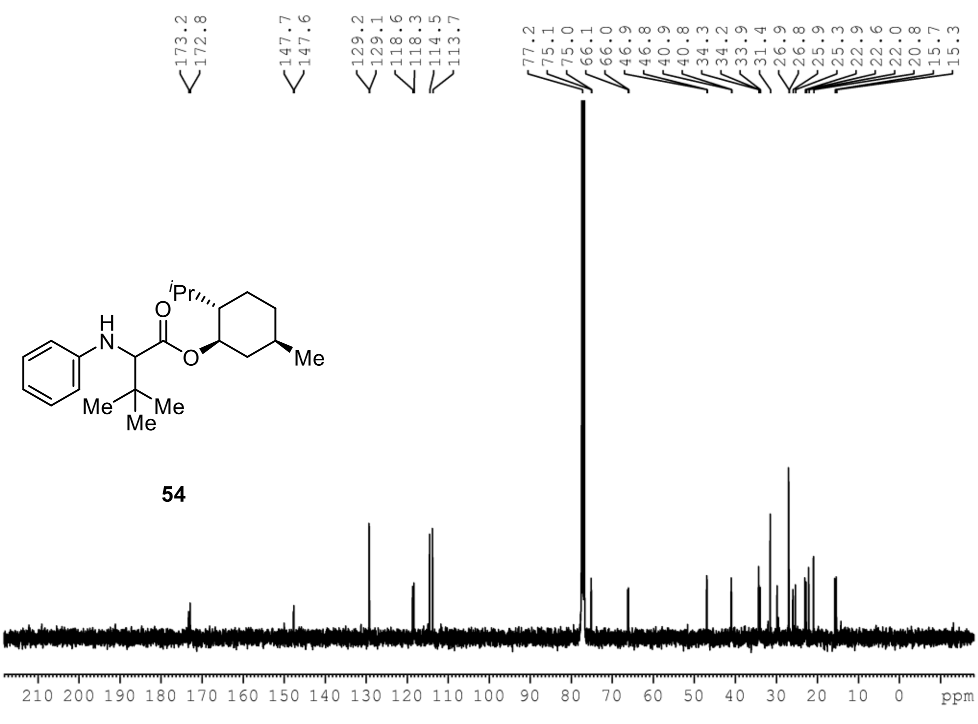

Supplementary Figure 92. ^{13}C NMR spectrum of compound 54

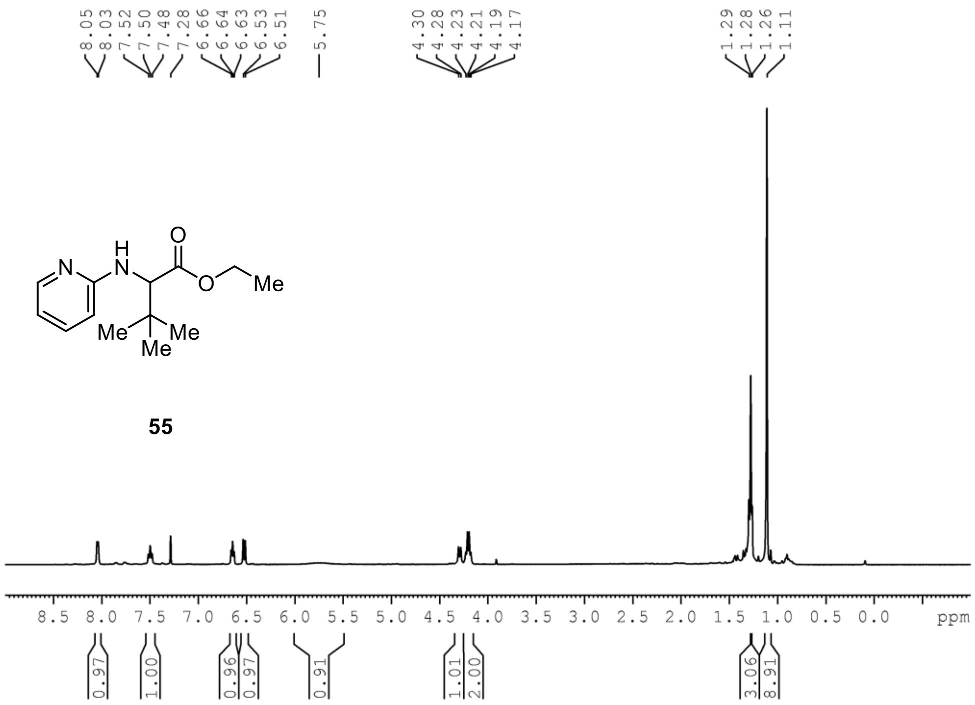

Supplementary Figure 93. ^{1}H NMR spectrum of compound 55

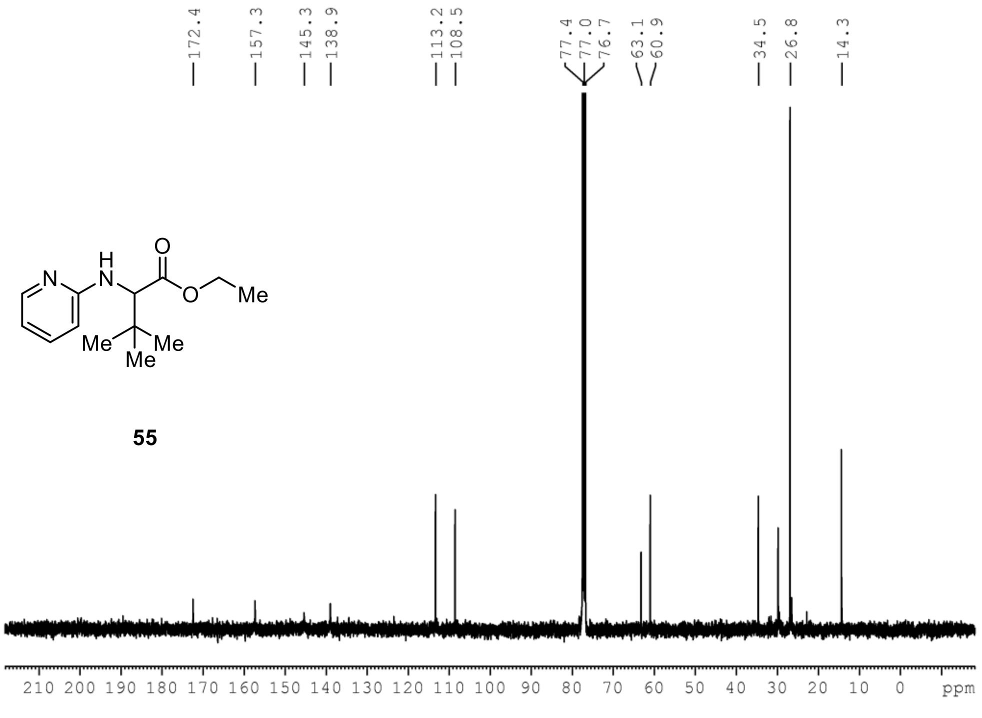

Supplementary Figure 94. ^{13}C NMR spectrum of compound 55

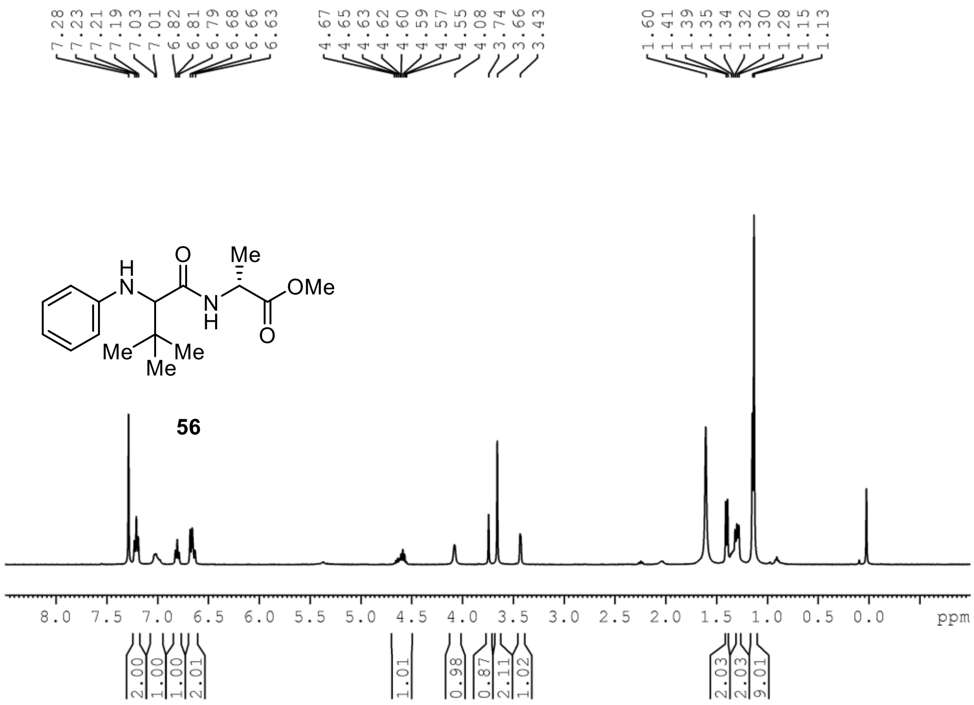

Supplementary Figure 95. ^{1}H NMR spectrum of compound 56

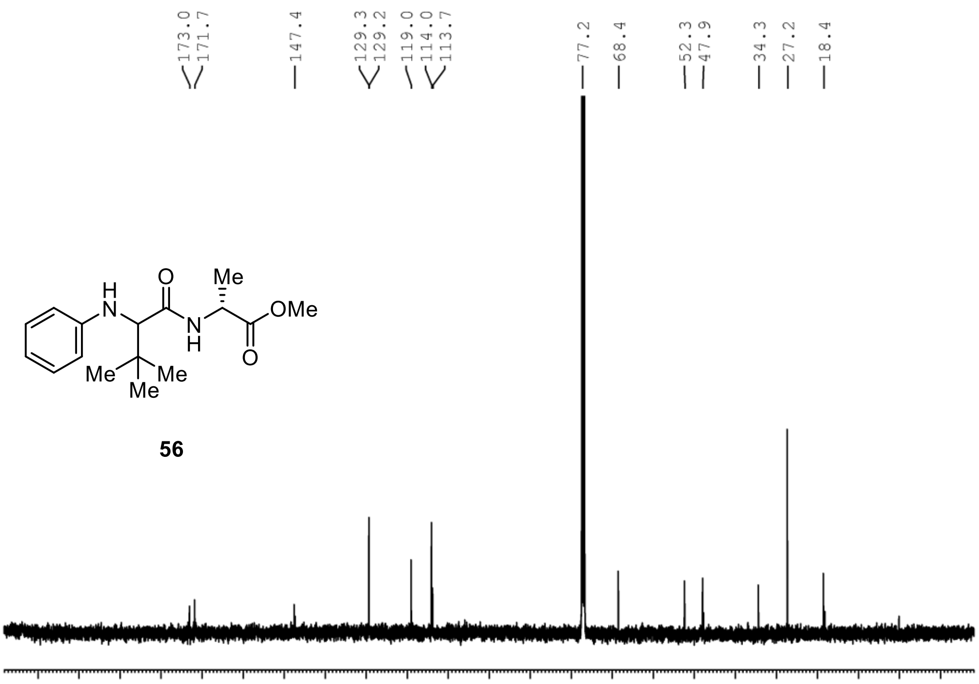

Supplementary Figure 96. ^{13}C NMR spectrum of compound 56

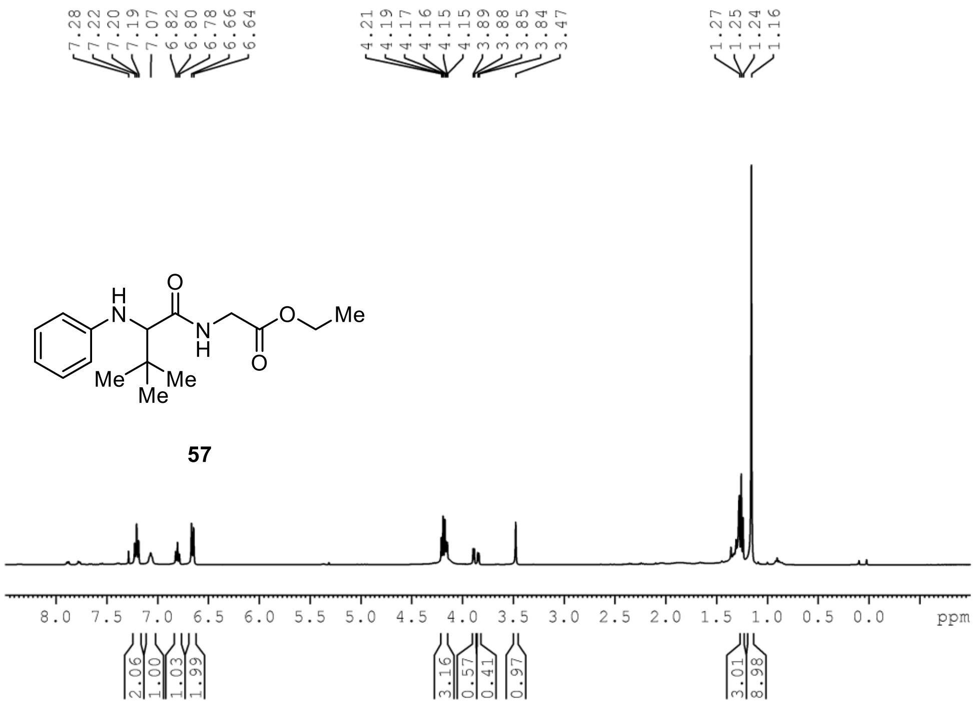

Supplementary Figure 97. ¹H NMR spectrum of compound 57

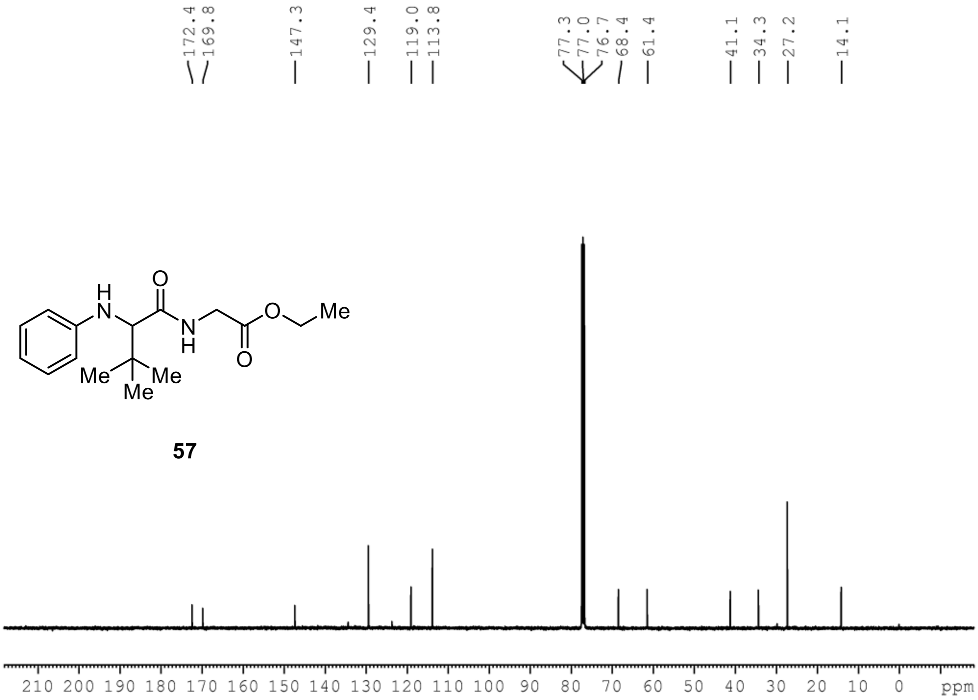

Supplementary Figure 98. ¹³C NMR spectrum of compound 57

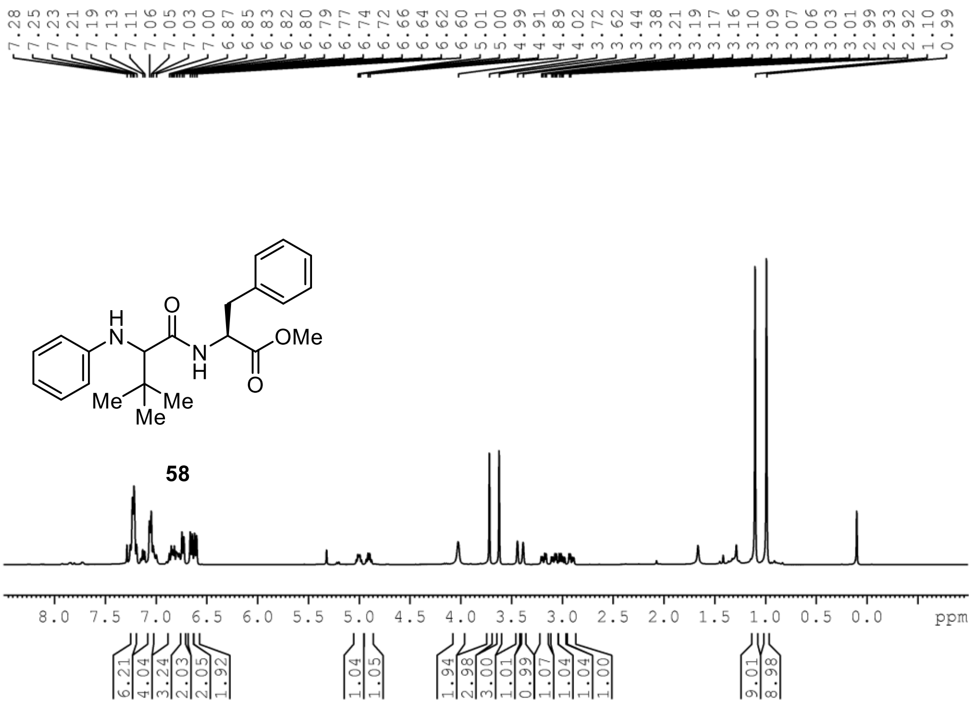

Supplementary Figure 99. ^{1}H NMR spectrum of compound 58

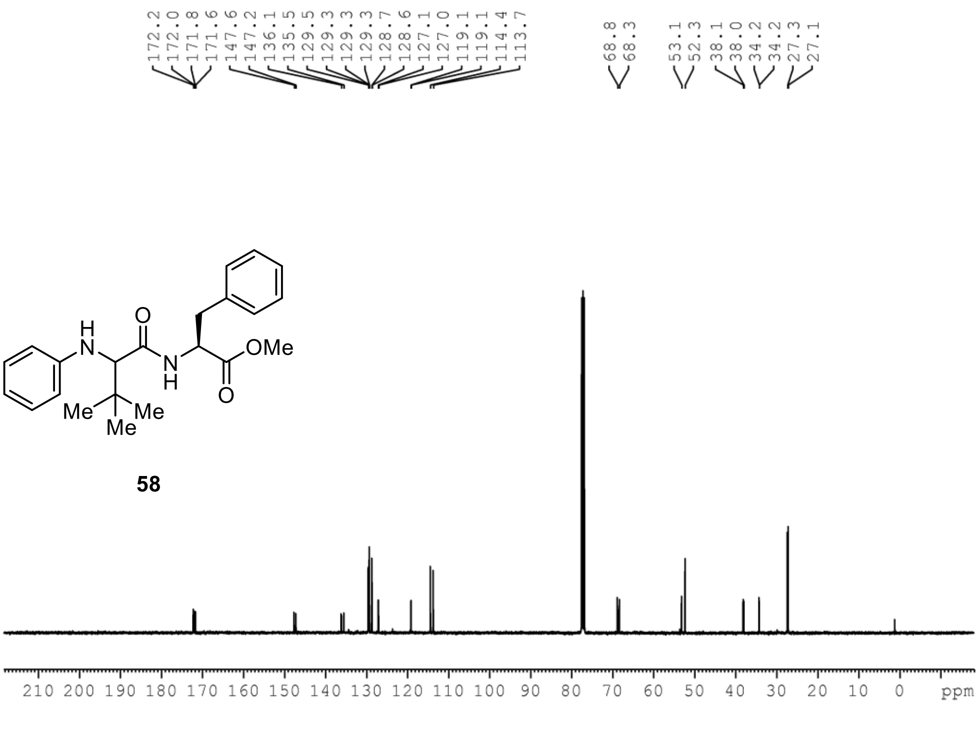

Supplementary Figure 100. ^{13}C NMR spectrum of compound 58

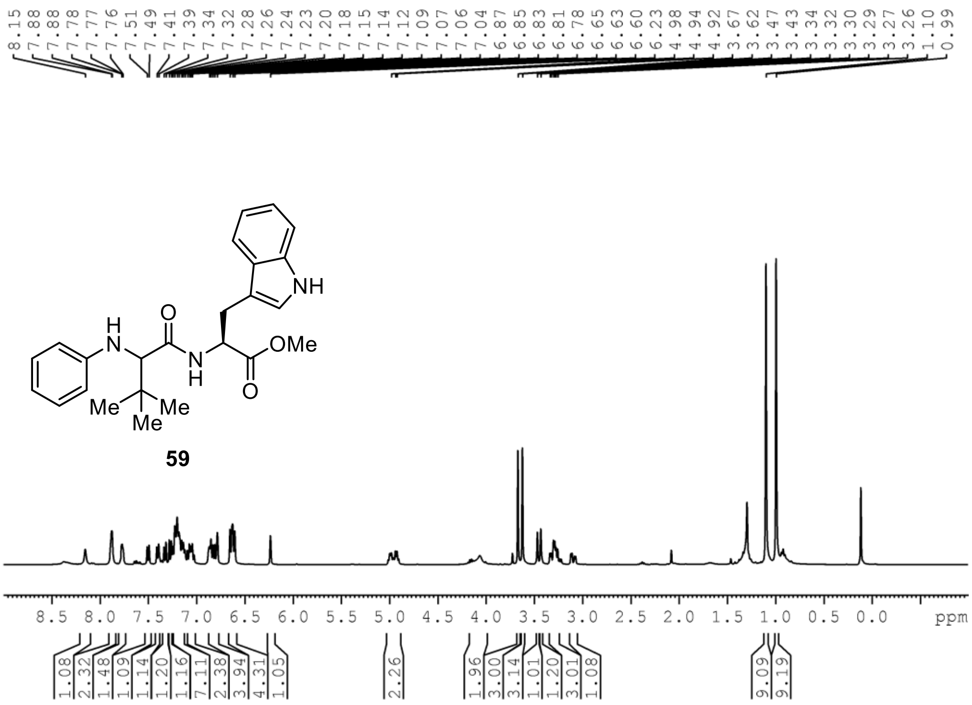

Supplementary Figure 101. ^{1}H NMR spectrum of compound 59

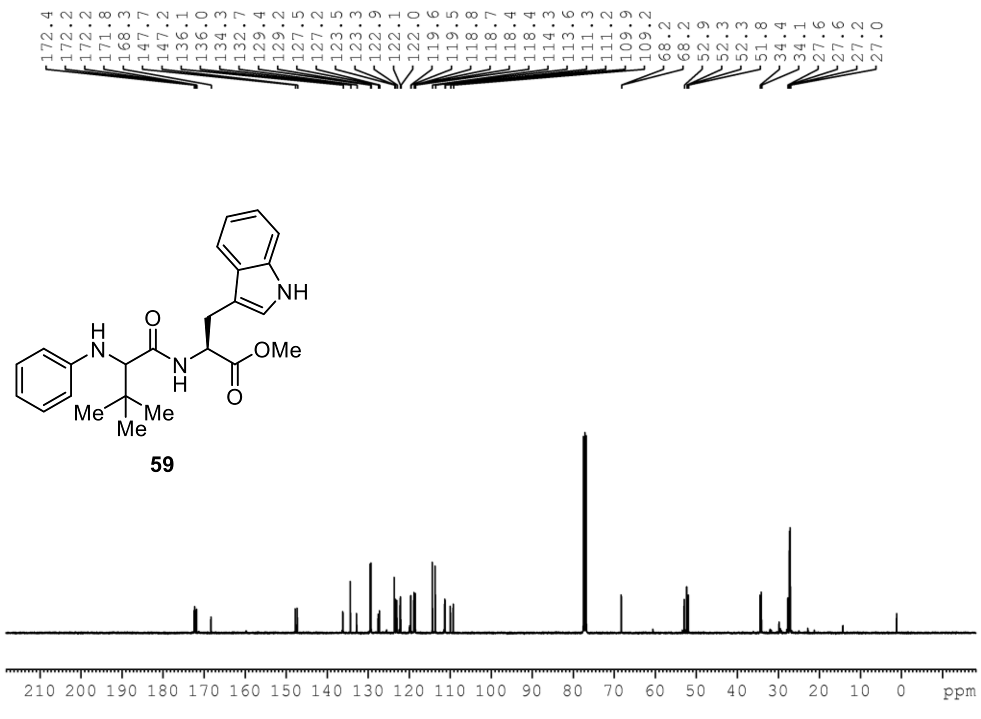

Supplementary Figure 102. ^{13}C NMR spectrum of compound 59

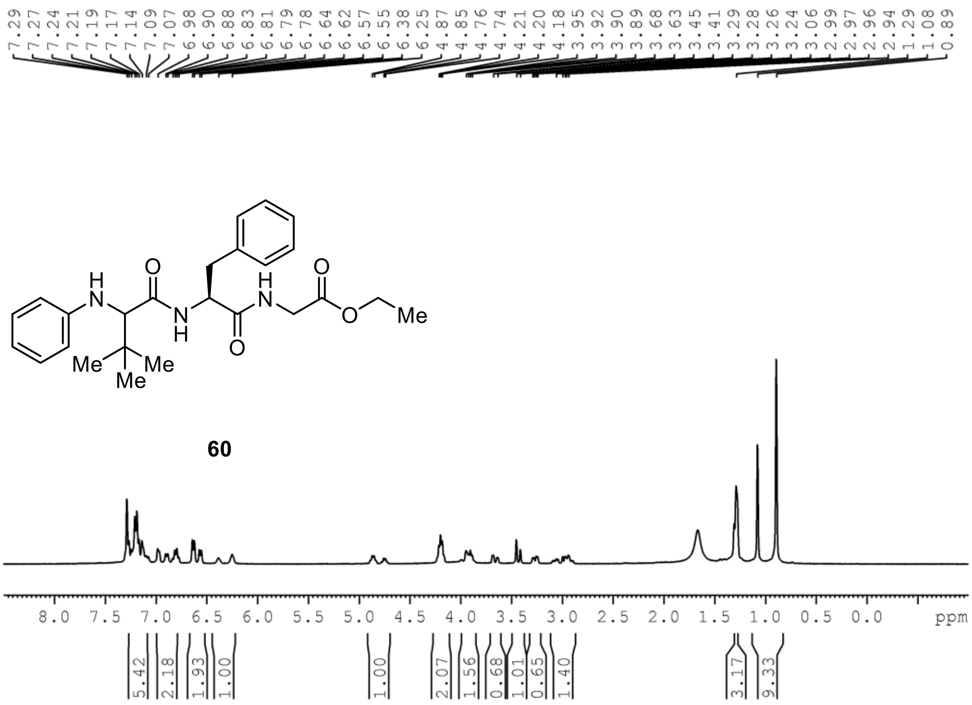

Supplementary Figure 103. ^{1}H NMR spectrum of compound 60

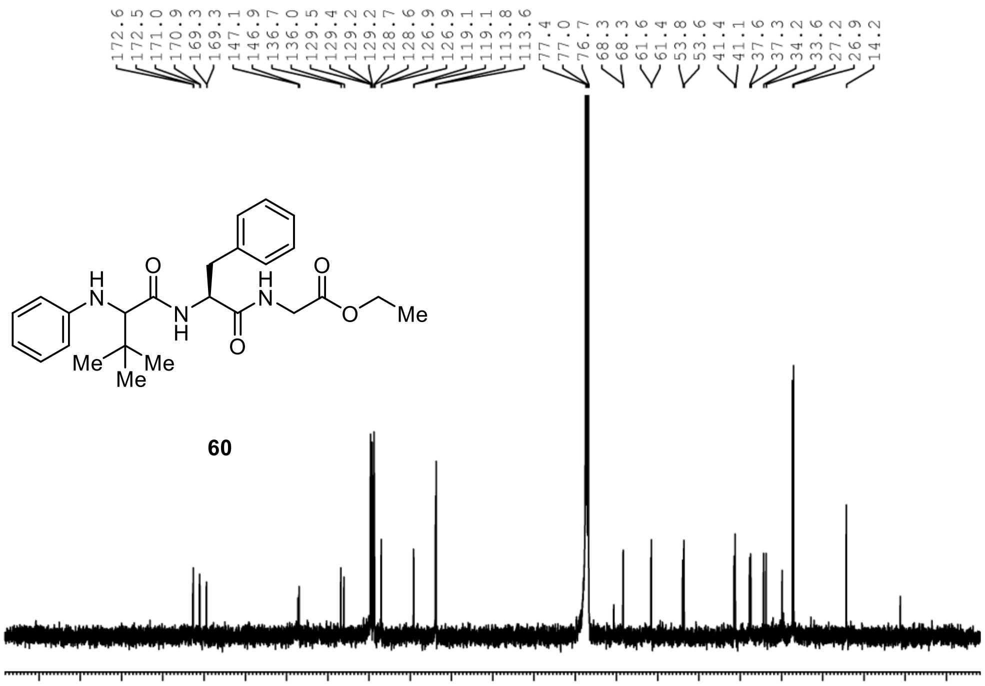

Supplementary Figure 104. ^{13}C NMR spectrum of compound 60

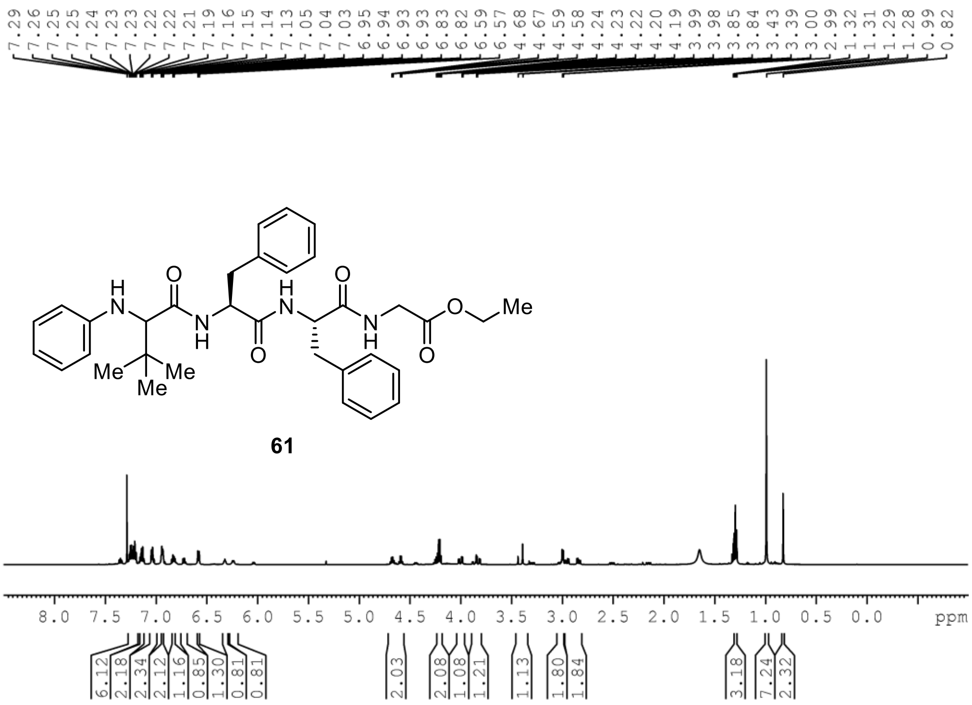

Supplementary Figure 105. ^{1}H NMR spectrum of compound 61

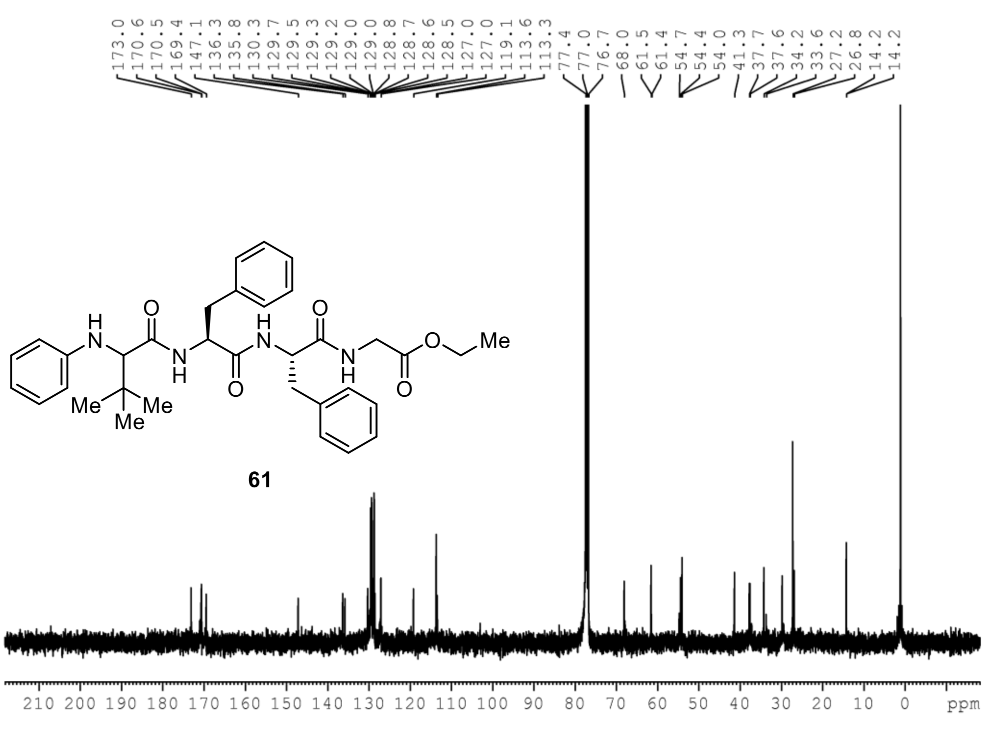

Supplementary Figure 106. ^{13}C NMR spectrum of compound 61

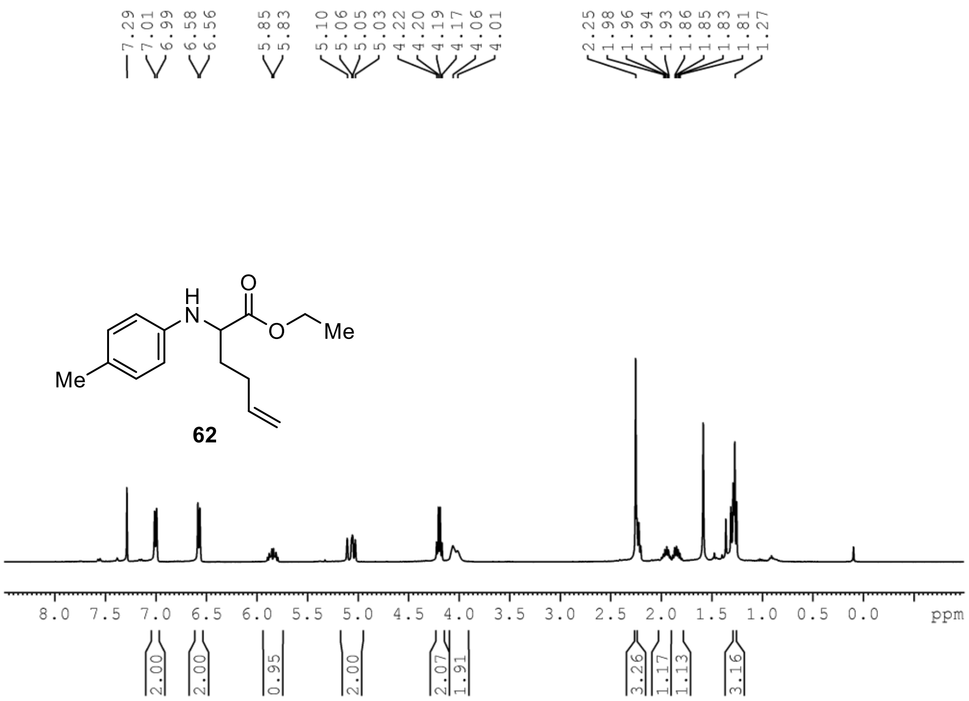

Supplementary Figure 107. ^{1}H NMR spectrum of compound 62

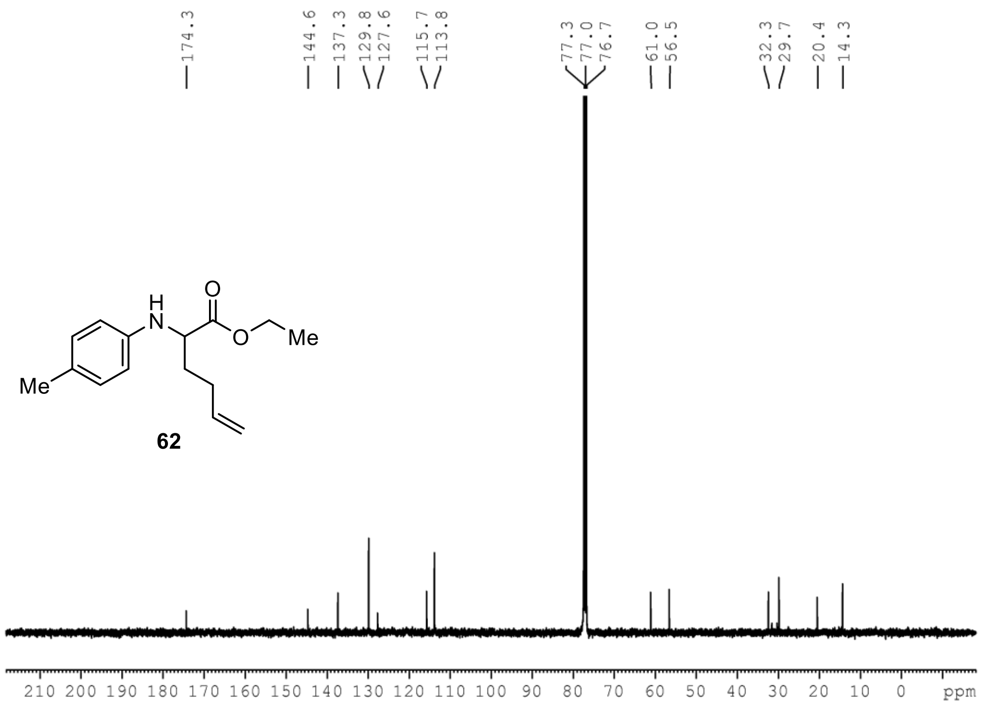

Supplementary Figure 108. ^{13}C NMR spectrum of compound 62

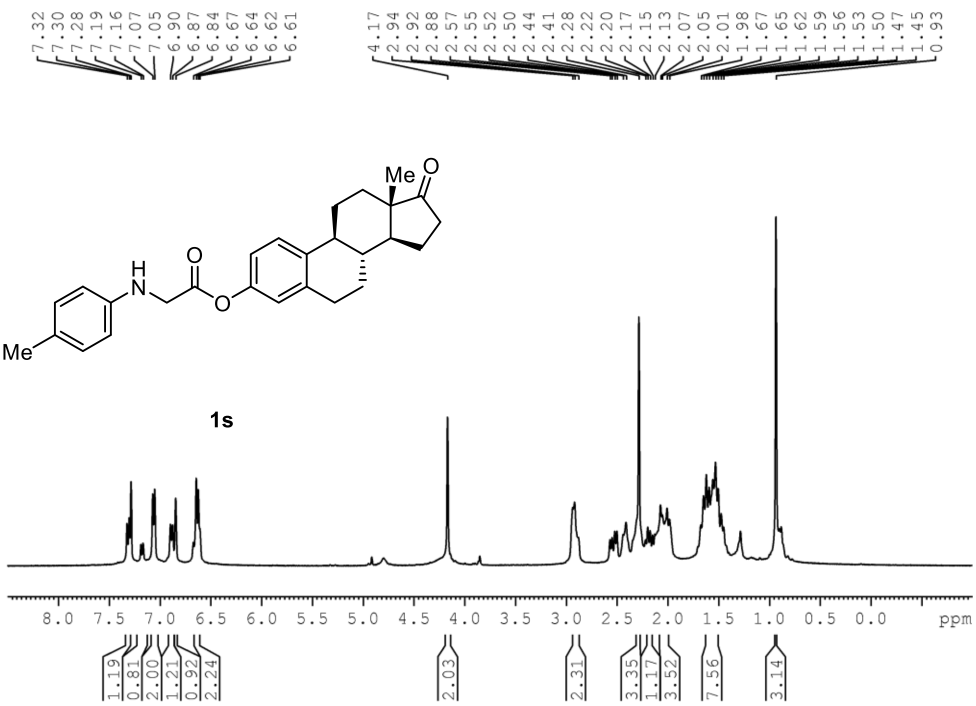

Supplementary Figure 109. ^{1}H NMR spectrum of compound 1s

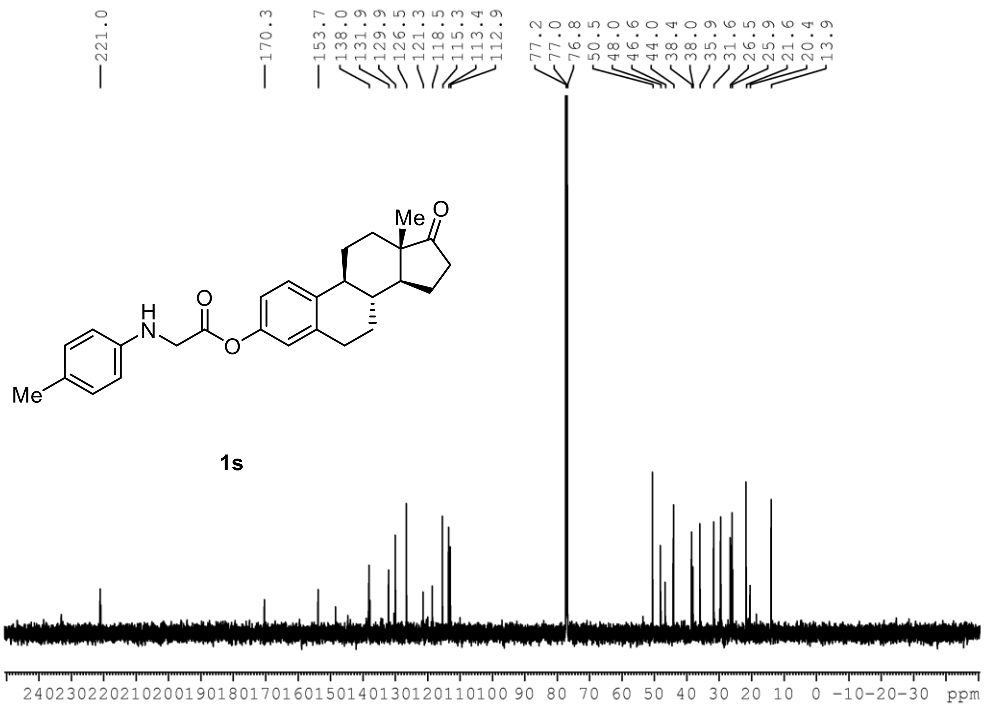

Supplementary Figure 110. ^{13}C NMR spectrum of compound 1s

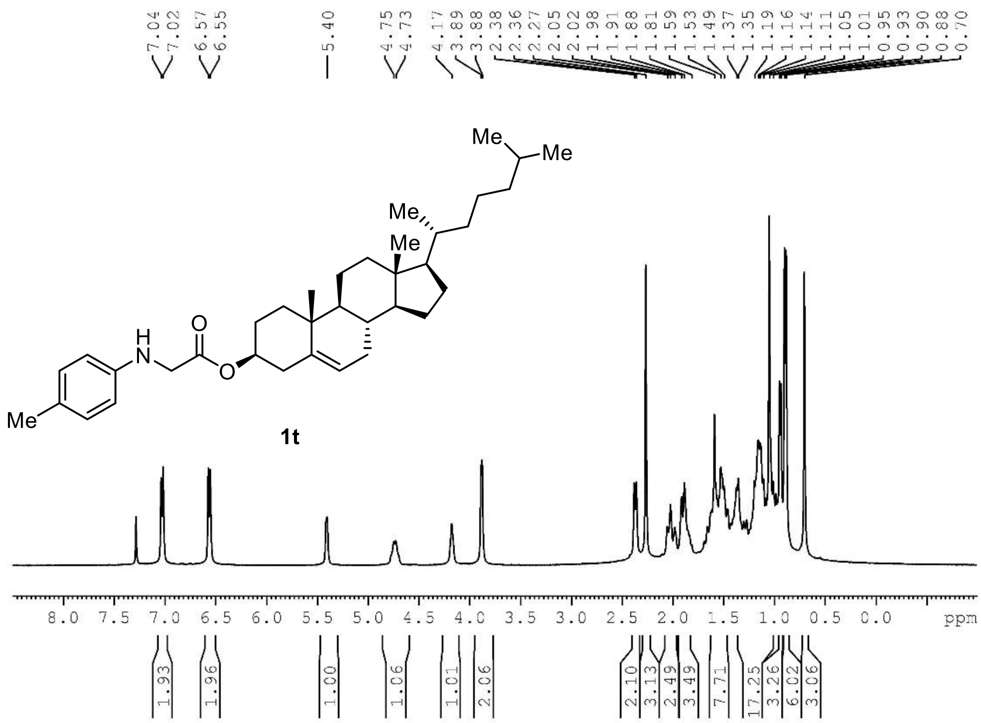

Supplementary Figure 111. ^{1}H NMR spectrum of compound 1t

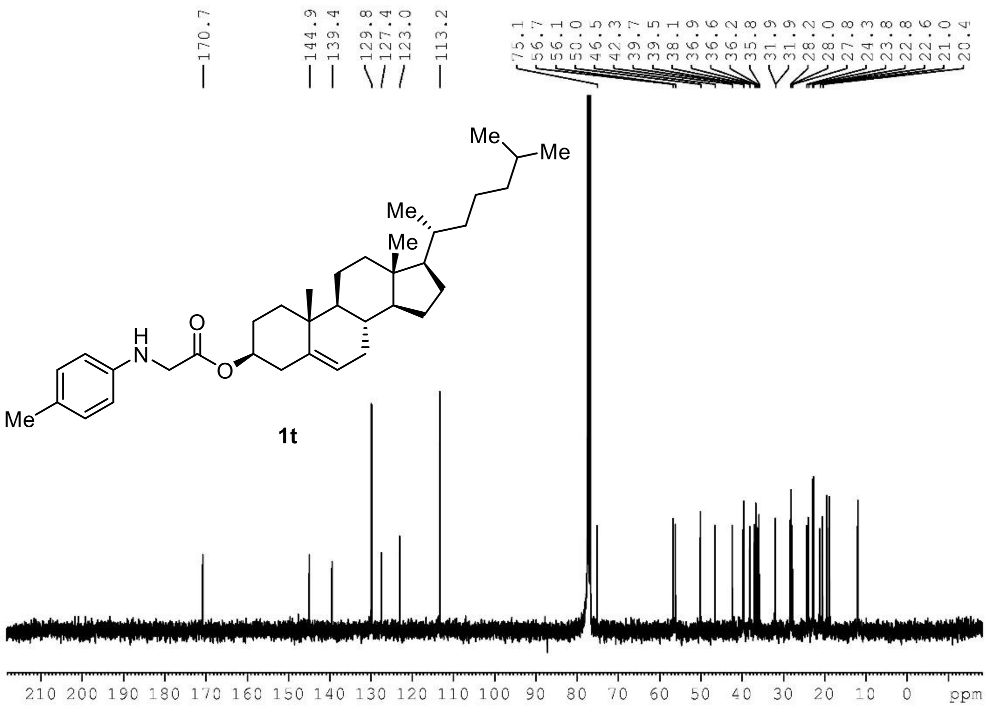

Supplementary Figure 112. ^{13}C NMR spectrum of compound 1t

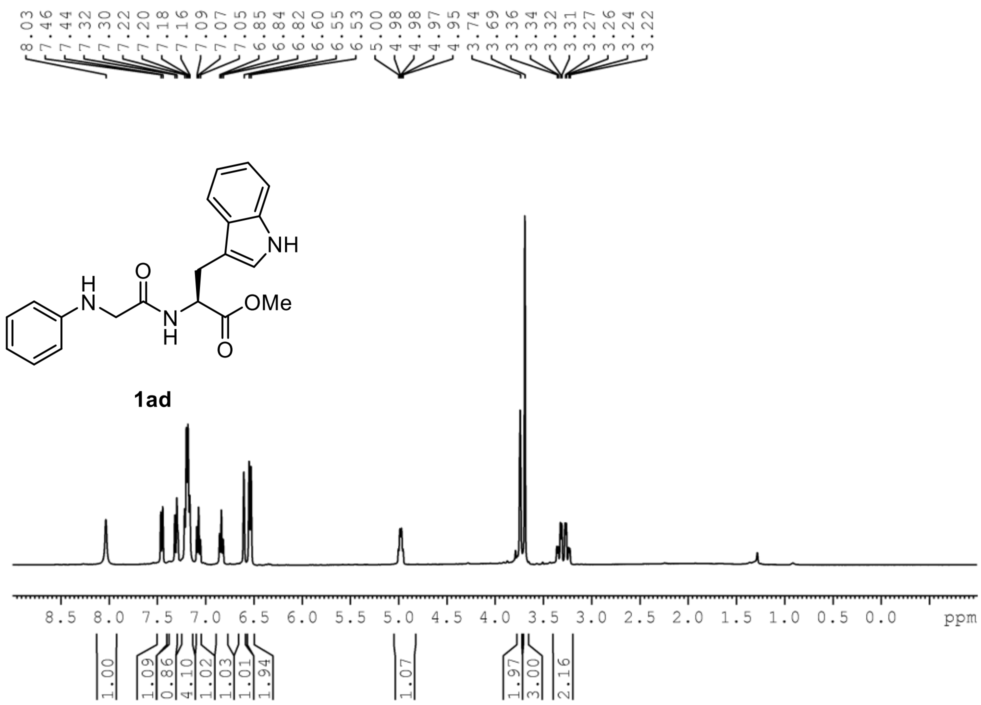

Supplementary Figure 113. ^{1}H NMR spectrum of compound 1ad

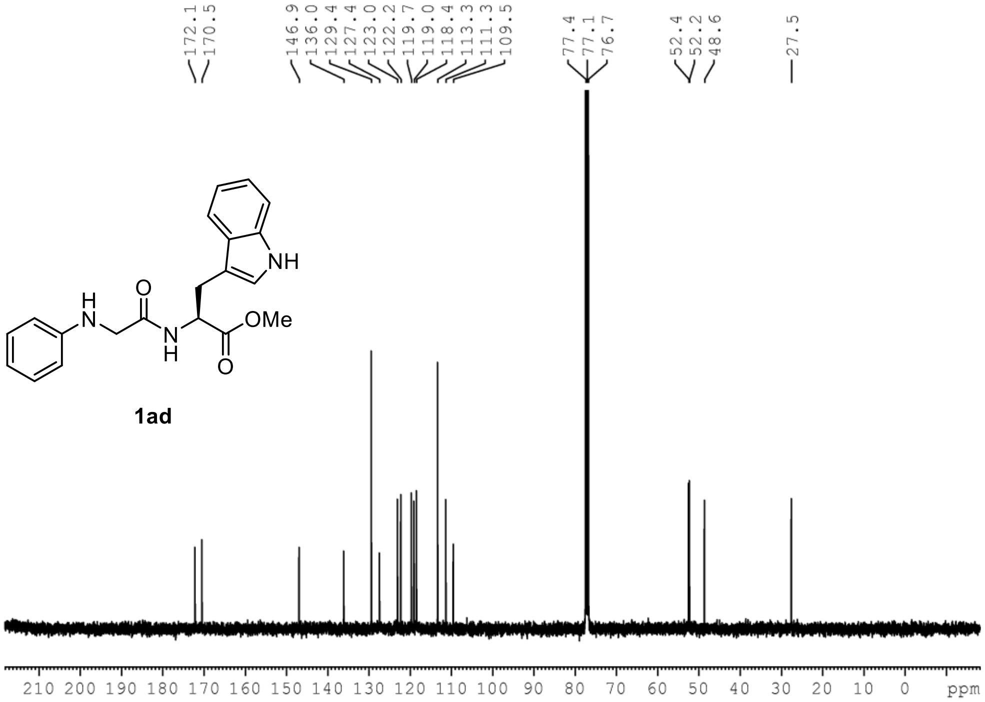

Supplementary Figure 114. ^{13}C NMR spectrum of compound 1ad

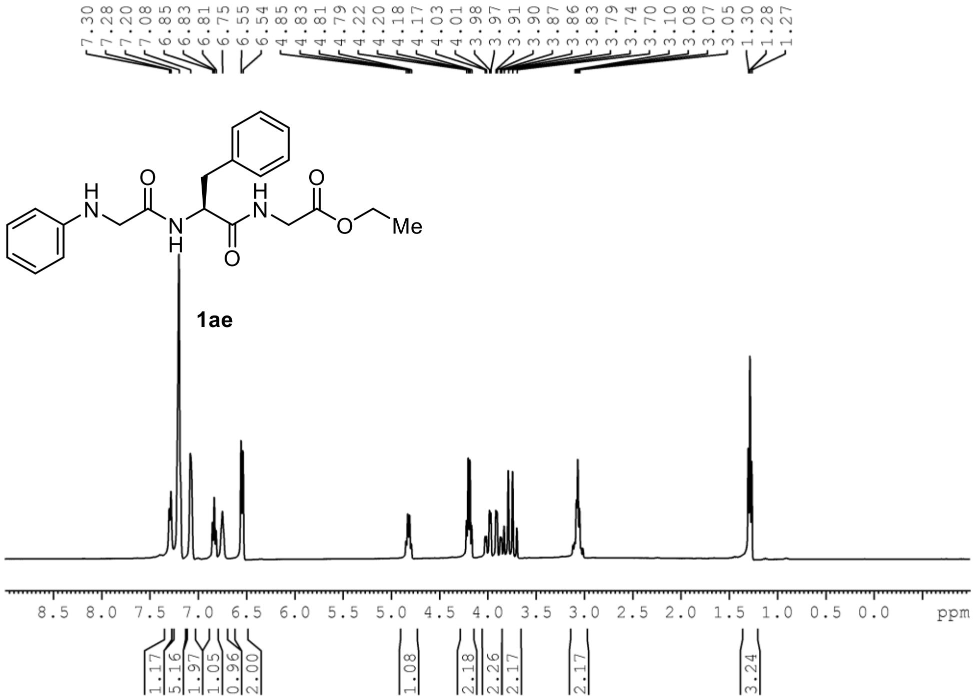

Supplementary Figure 115. ^{1}H NMR spectrum of compound 1ae

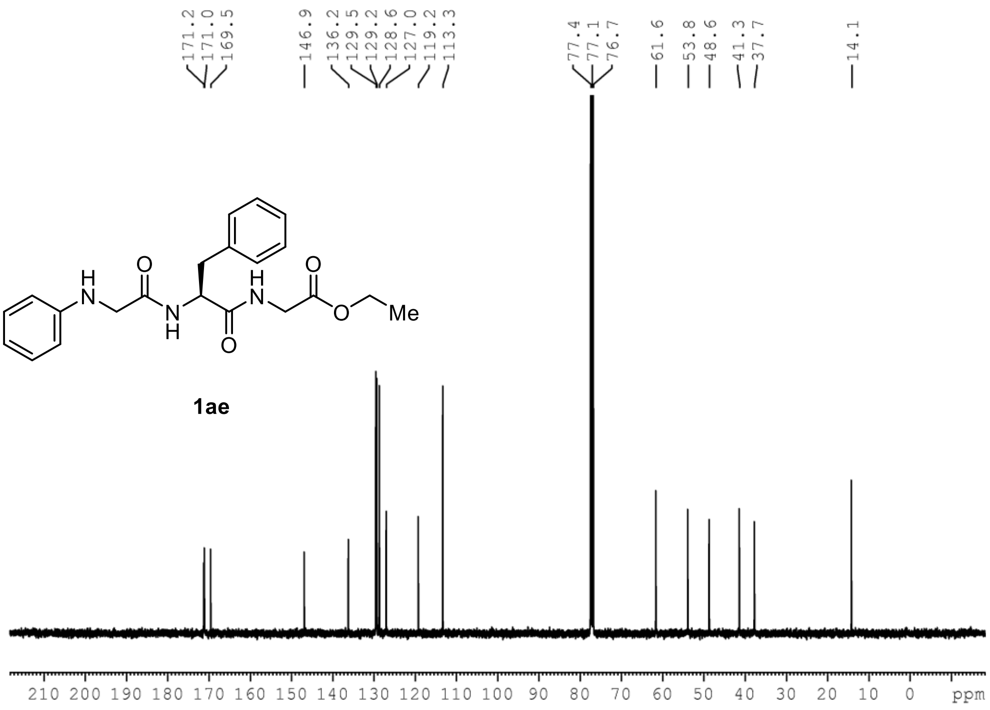

Supplementary Figure 116. ^{13}C NMR spectrum of compound 1ae

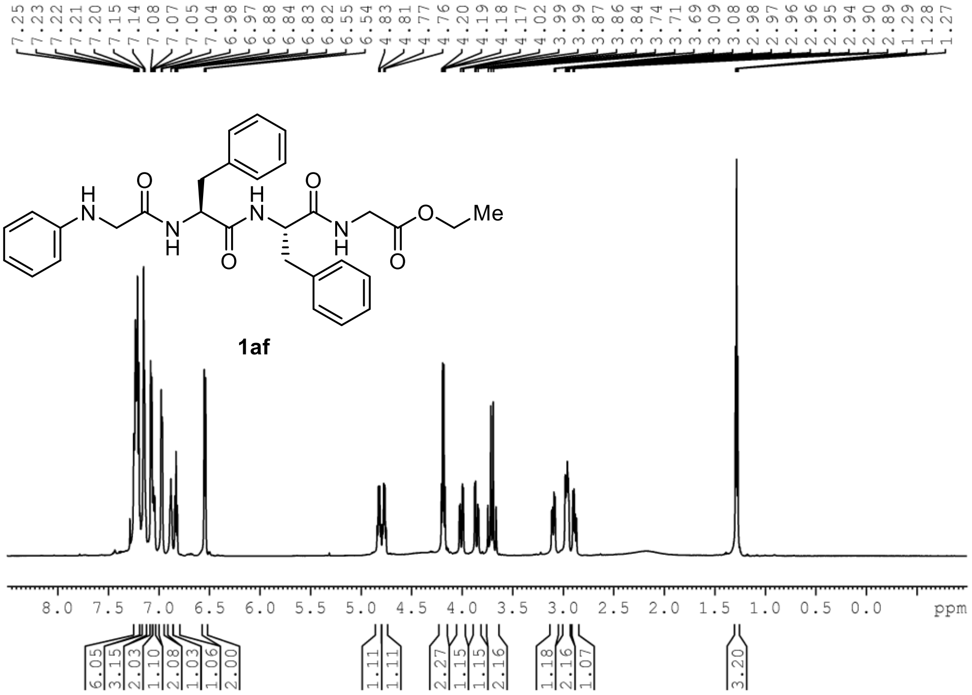

Supplementary Figure 117. ^{1}H NMR spectrum of compound 1af

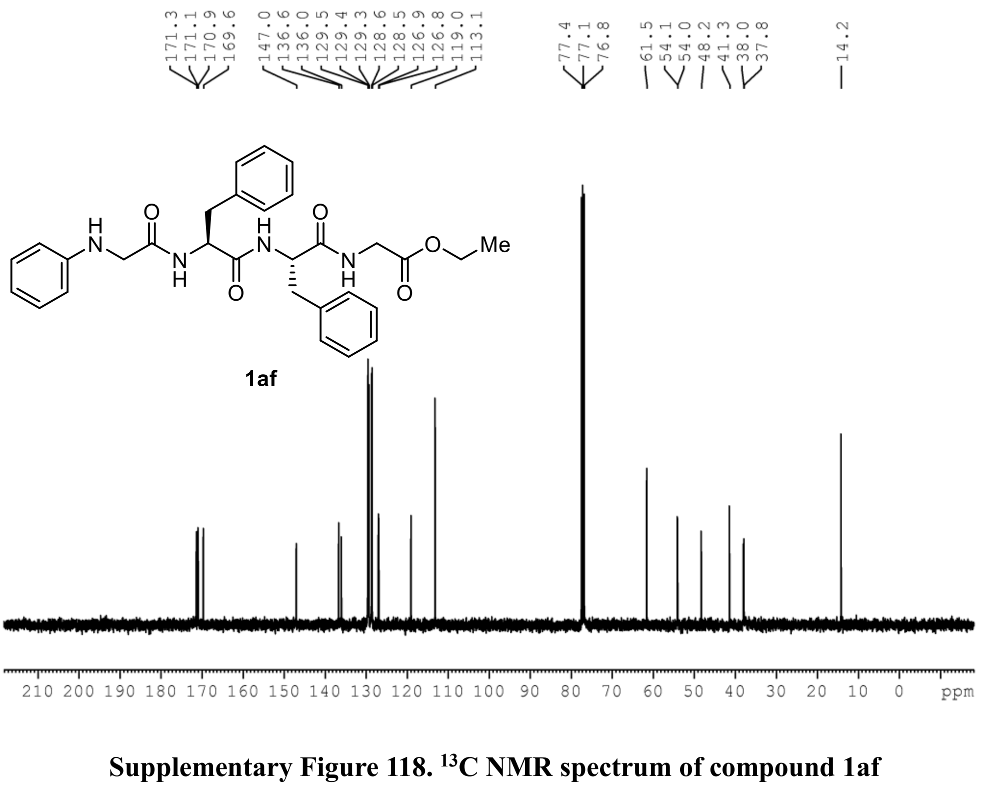

Supplementary Figure 118. ^{13}C NMR spectrum of compound 1af

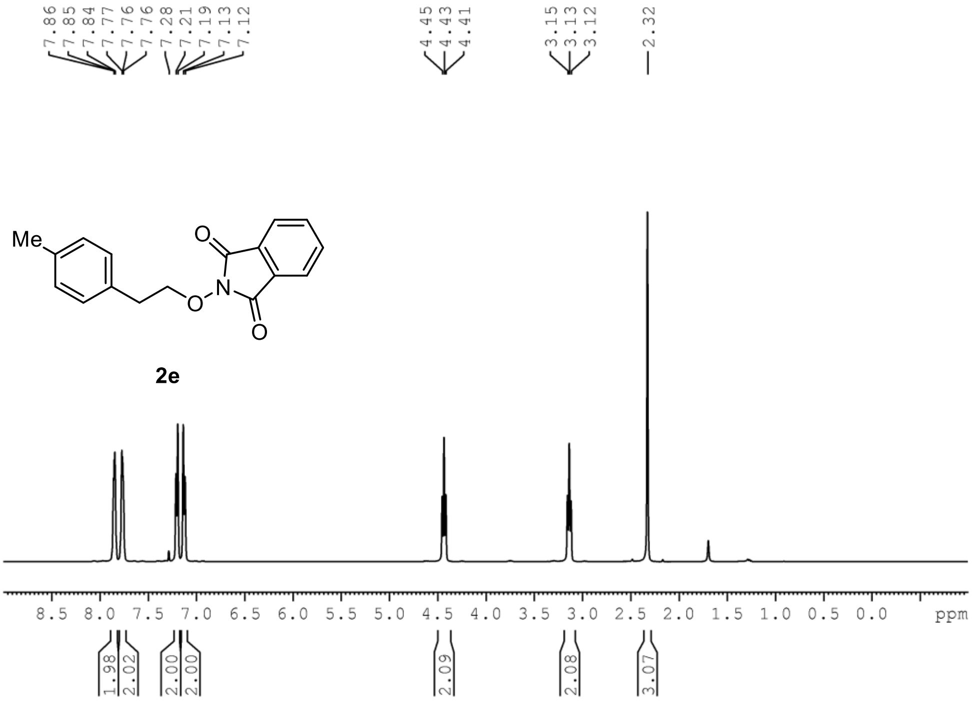

Supplementary Figure 119. ^{1}H NMR spectrum of compound 2e

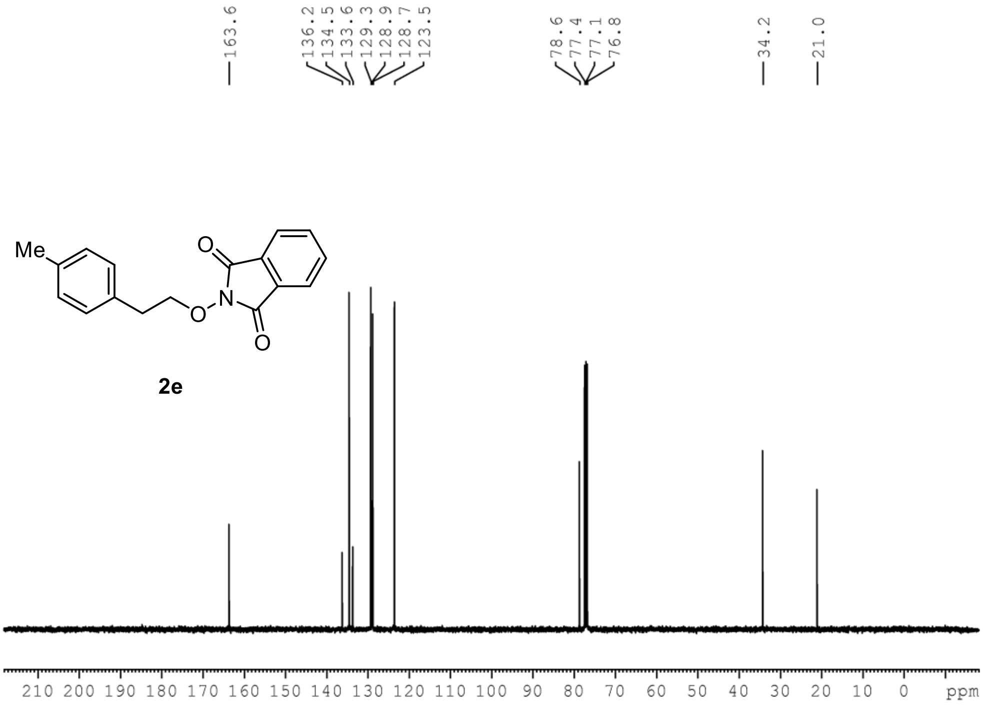

Supplementary Figure 120. ^{13}C NMR spectrum of compound 2e

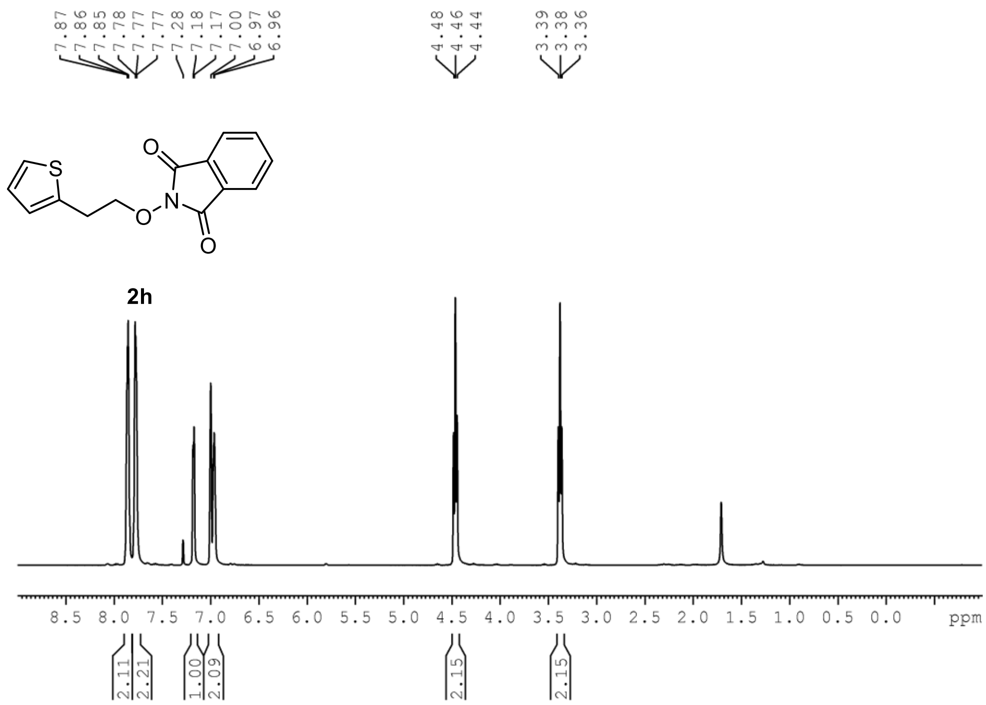

Supplementary Figure 121. ^{1}H NMR spectrum of compound 2h

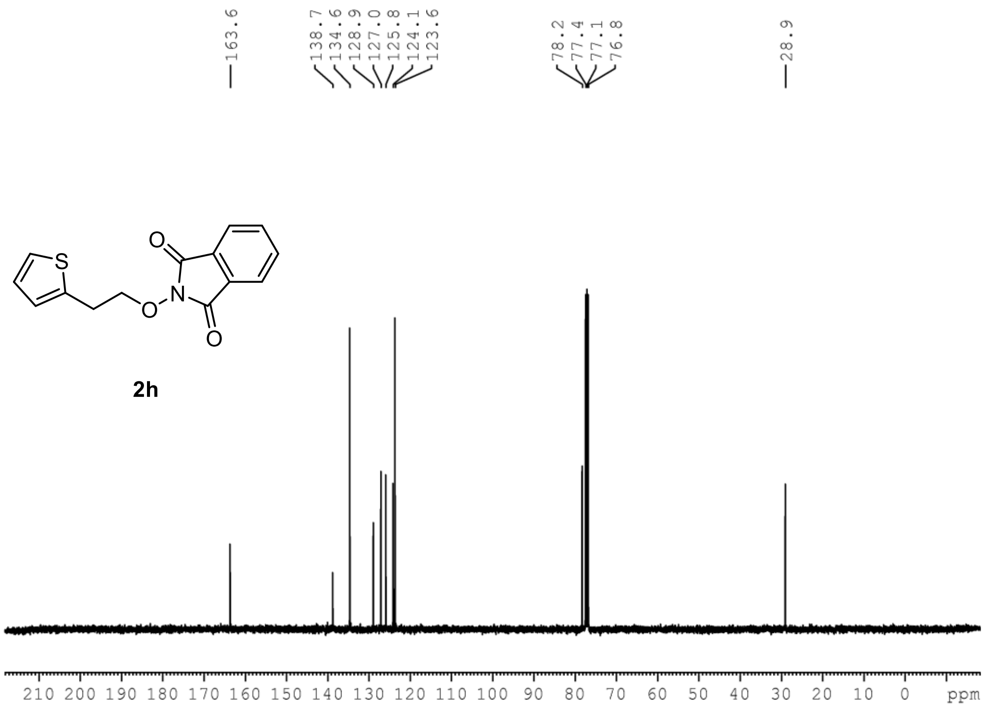

Supplementary Figure 122. ^{13}C NMR spectrum of compound 2h

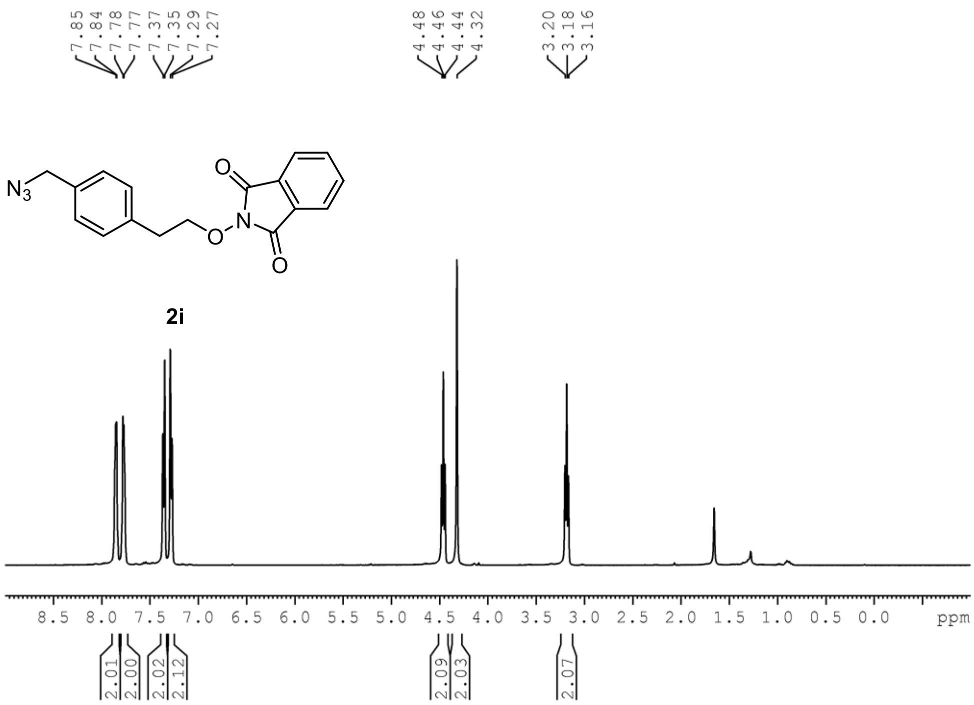

Supplementary Figure 123. ^{1}H NMR spectrum of compound 2i

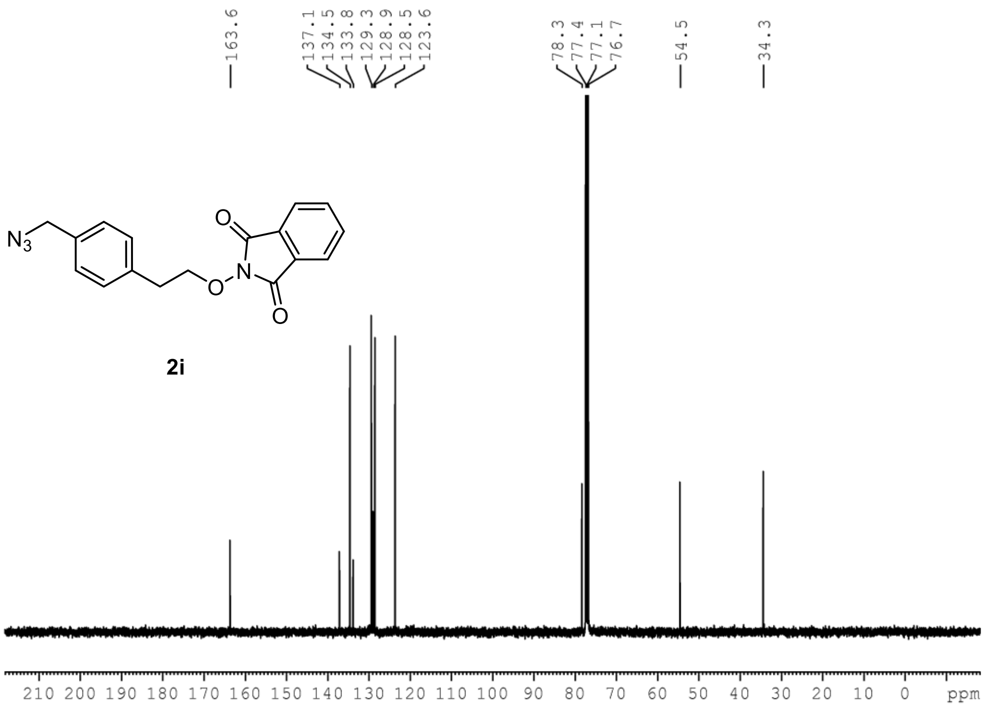

Supplementary Figure 124. ^{13}C NMR spectrum of compound 2i

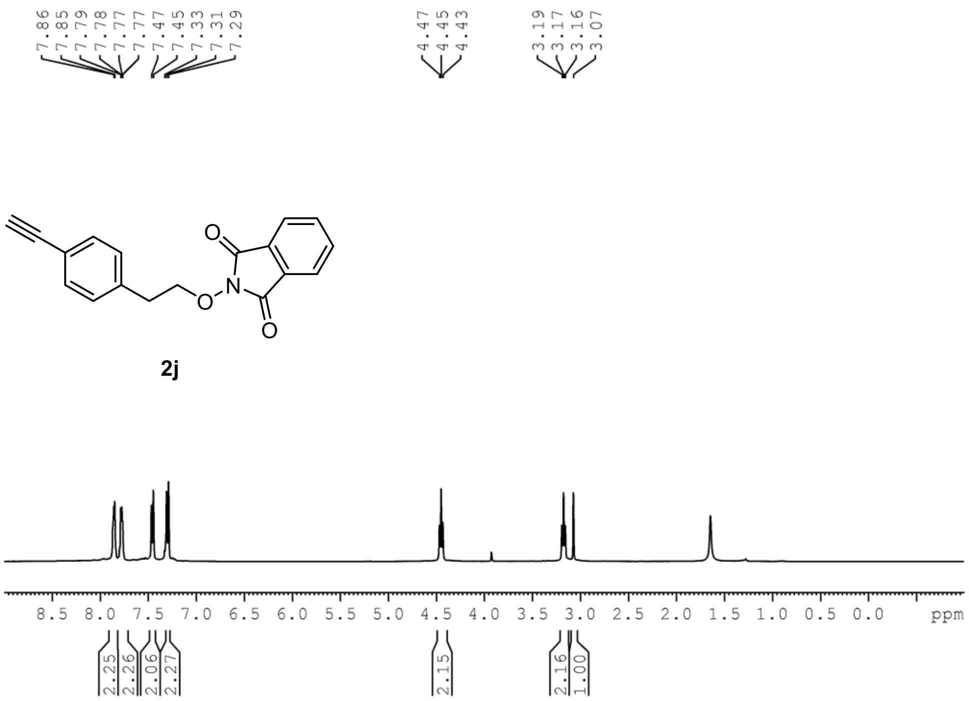

Supplementary Figure 125. ^{1}H NMR spectrum of compound 2j

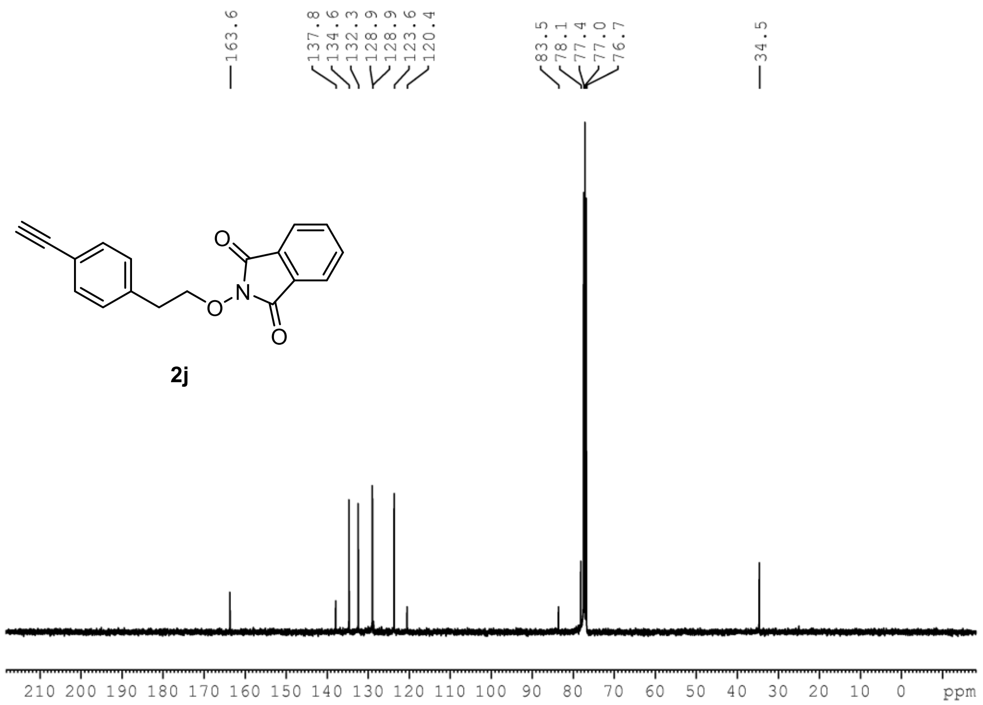

Supplementary Figure 126. ^{13}C NMR spectrum of compound 2j

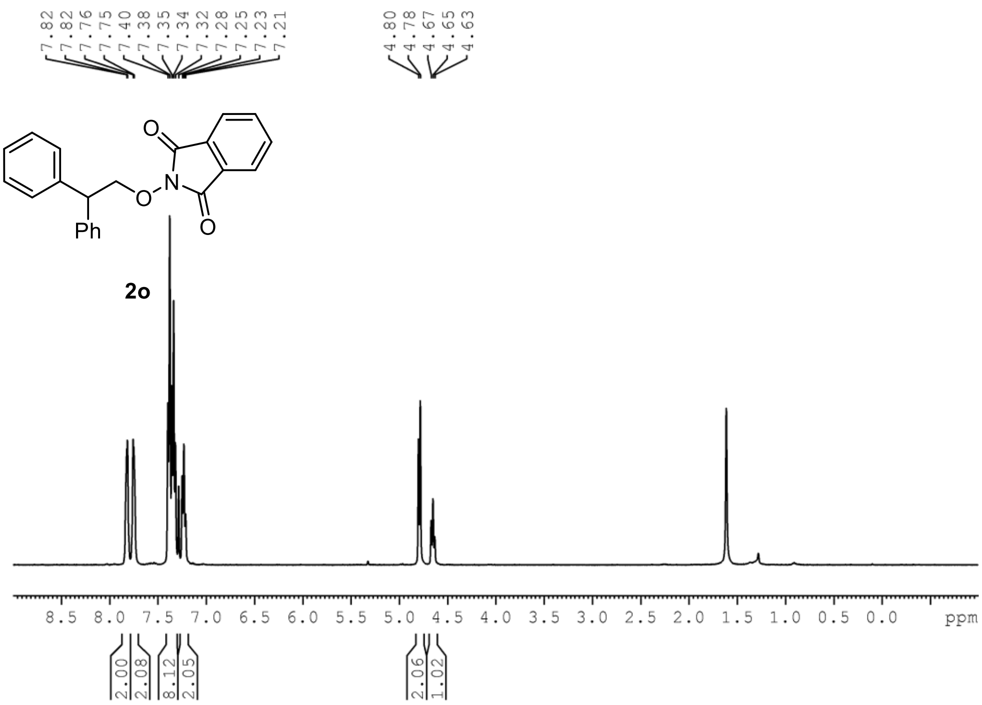

Supplementary Figure 127. ^{1}H NMR spectrum of compound 2o

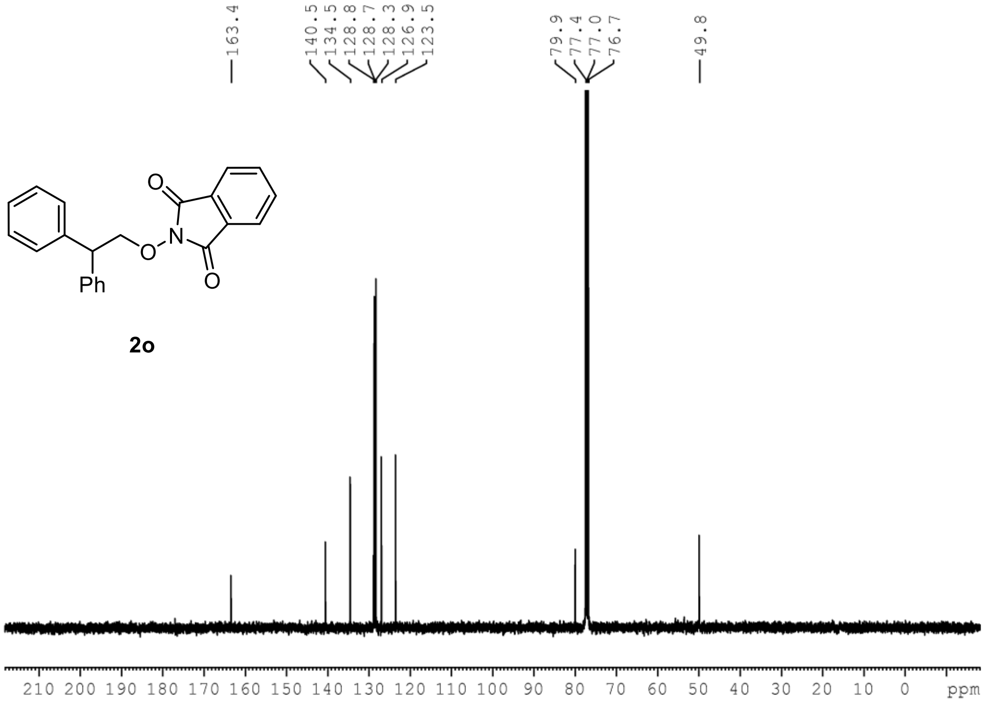

Supplementary Figure 128. ^{13}C NMR spectrum of compound 2o

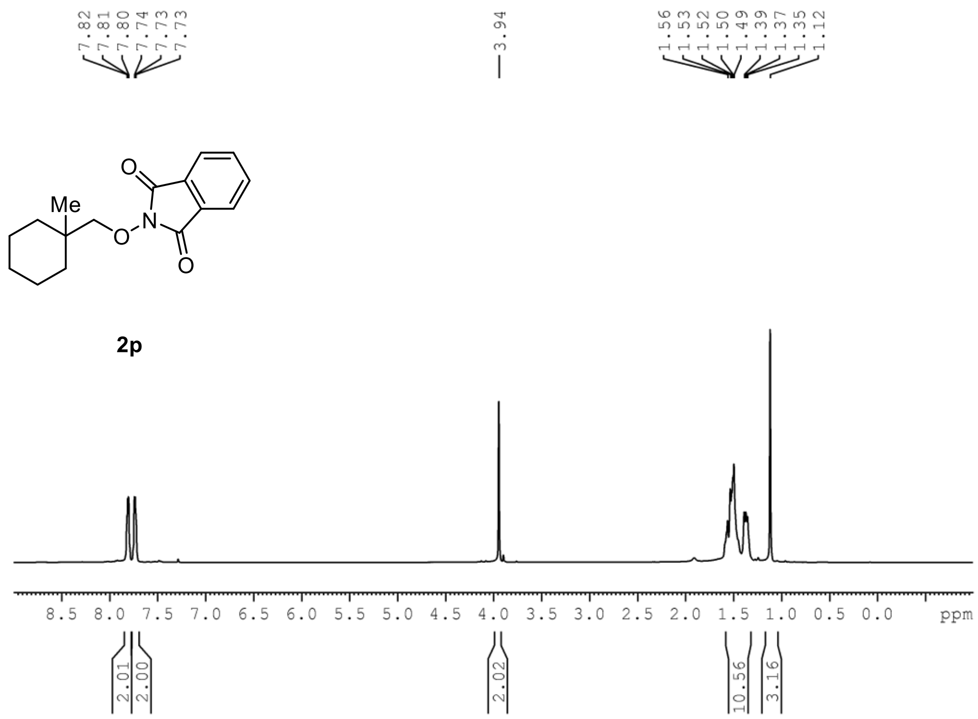

Supplementary Figure 129. ^{1}H NMR spectrum of compound 2p

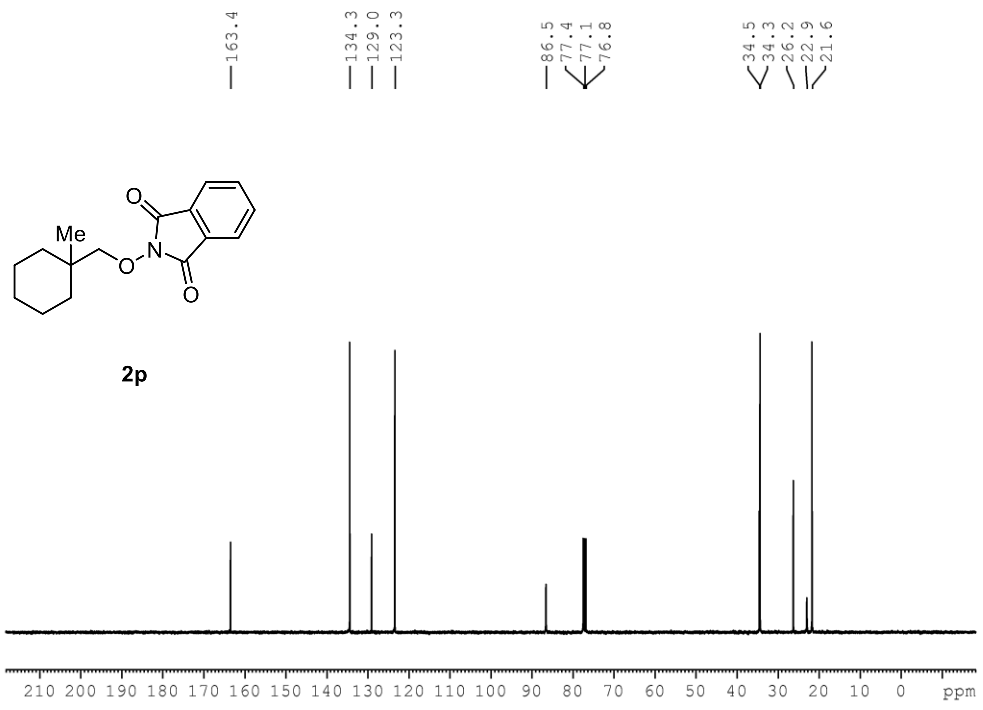

Supplementary Figure 130. ^{13}C NMR spectrum of compound 2p

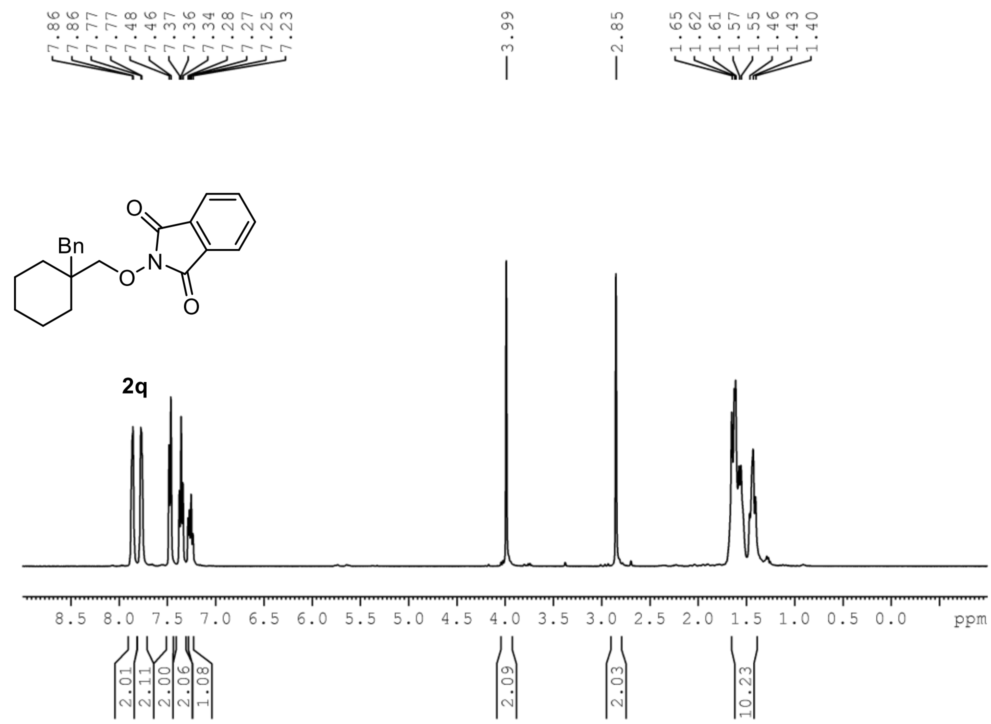

Supplementary Figure 131. ^{1}H NMR spectrum of compound 2q

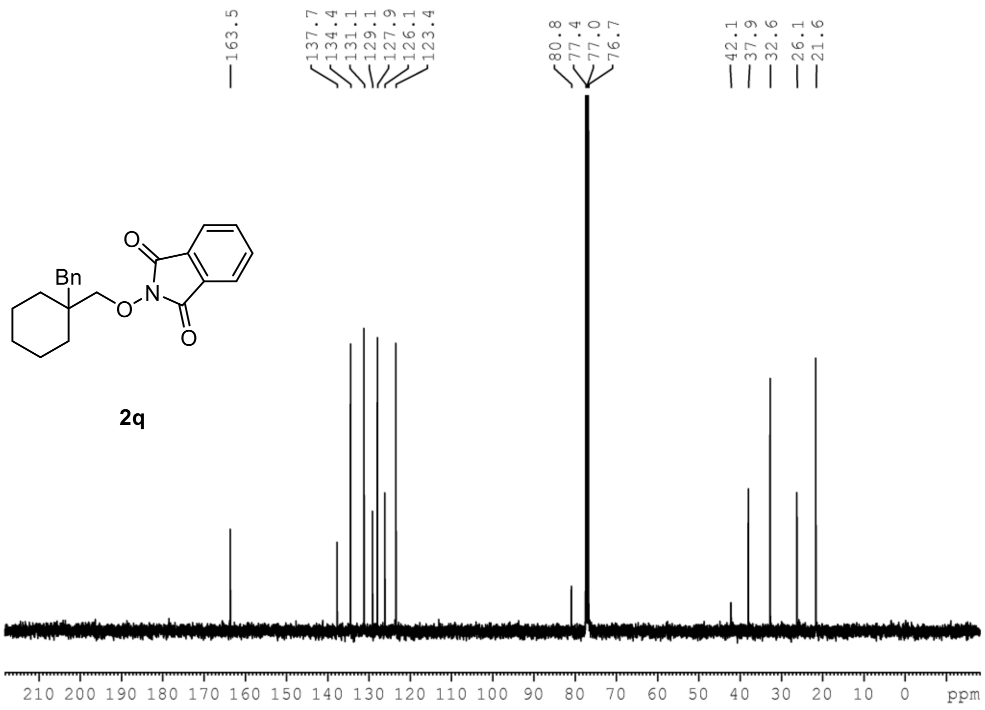

Supplementary Figure 132. ^{13}C NMR spectrum of compound 2q

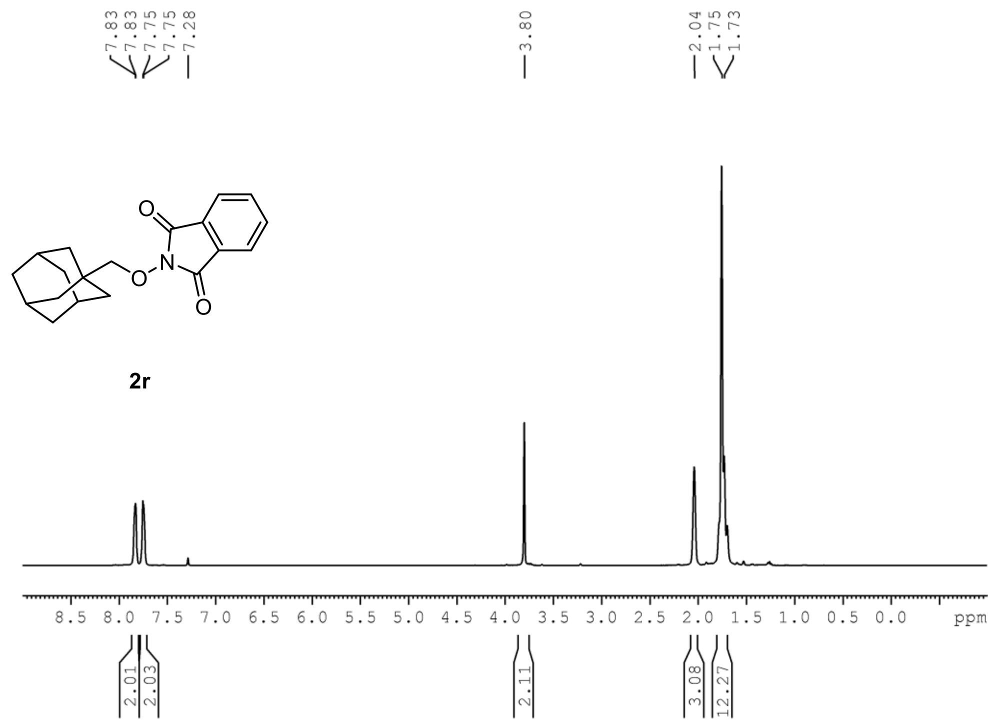

Supplementary Figure 133. ^{1}H NMR spectrum of compound 2r

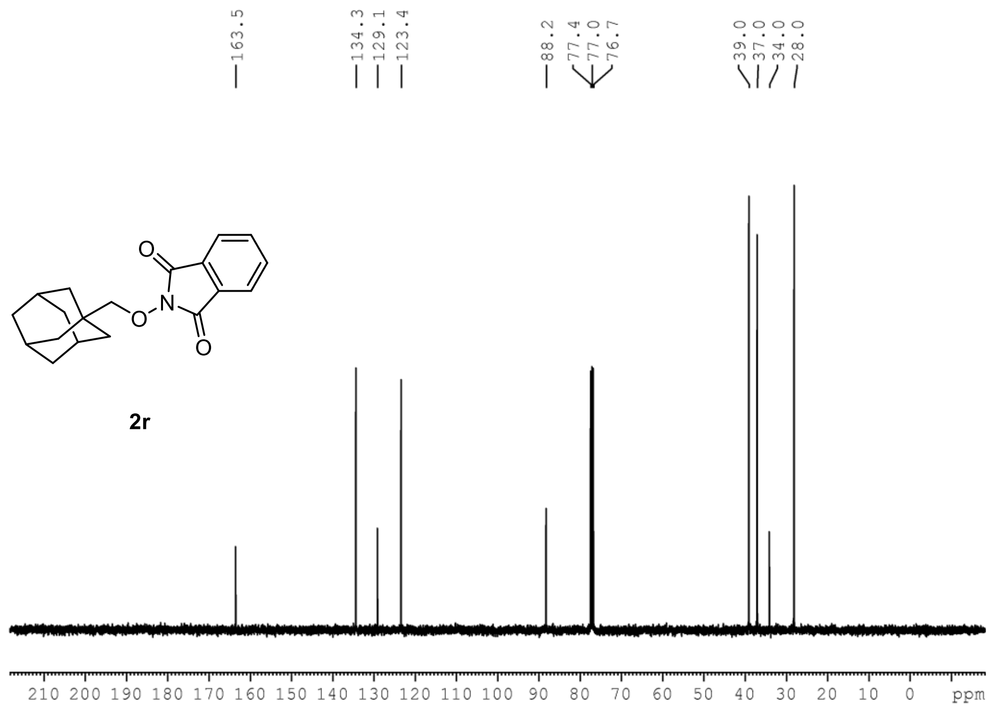

Supplementary Figure 134. ^{13}C NMR spectrum of compound 2r

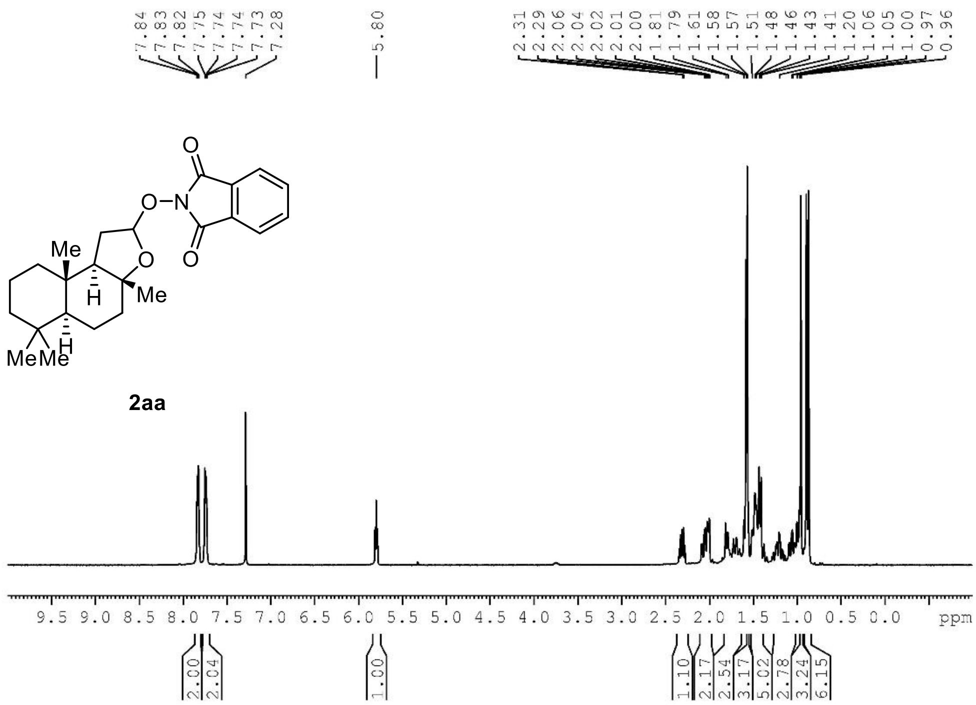

Supplementary Figure 135. ^{1}H NMR spectrum of compound 2aa

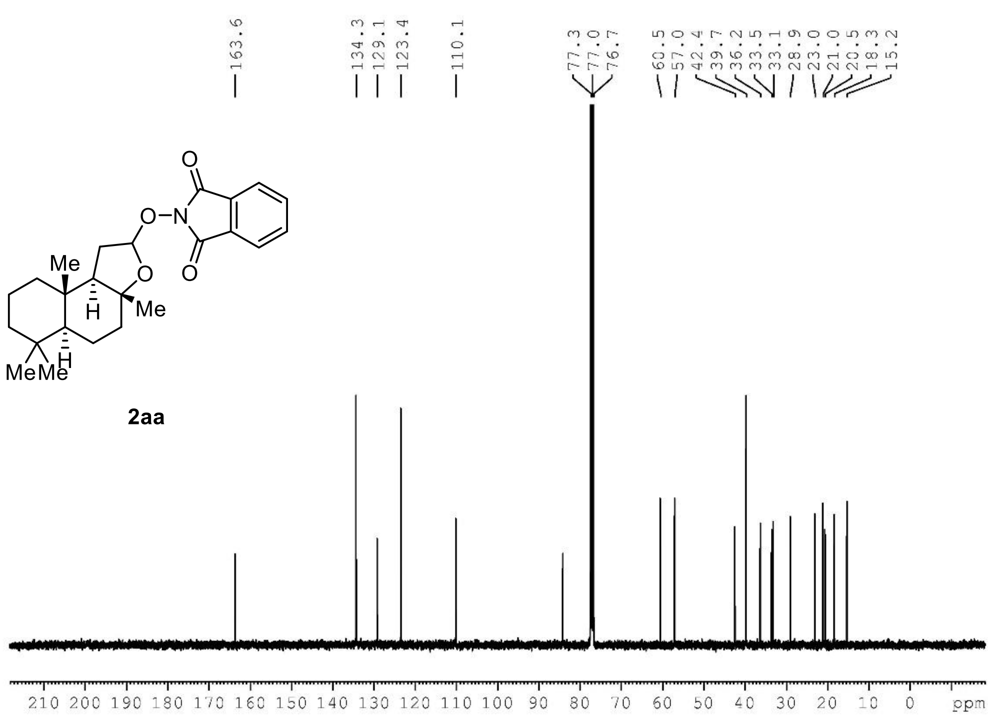

Supplementary Figure 136. ^{13}C NMR spectrum of compound 2aa

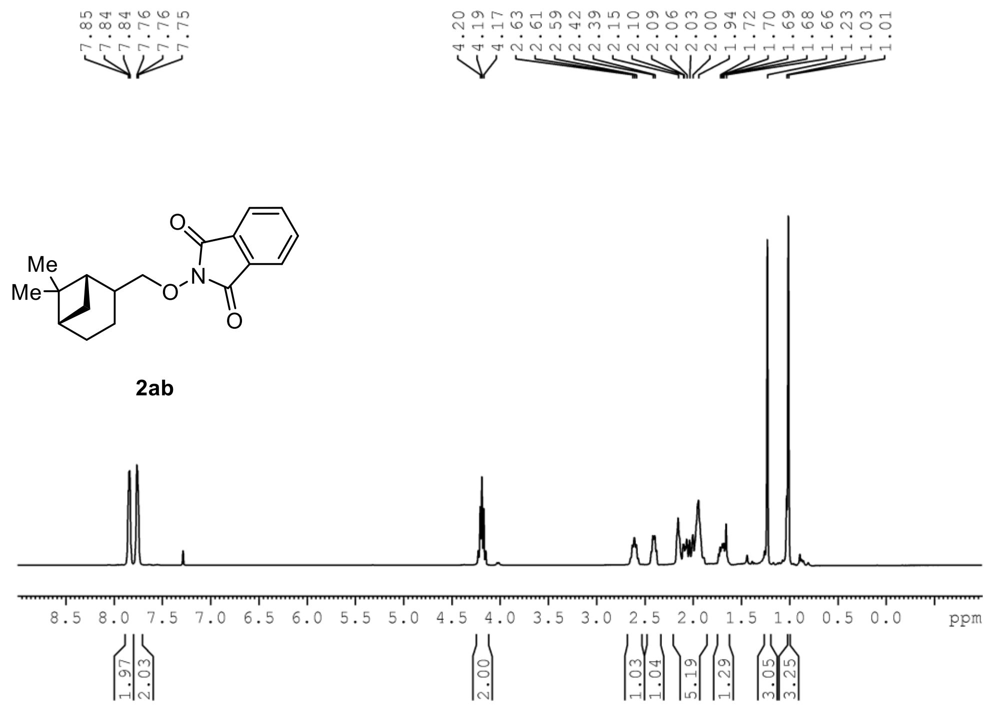

Supplementary Figure 137. ^{1}H NMR spectrum of compound 2ab

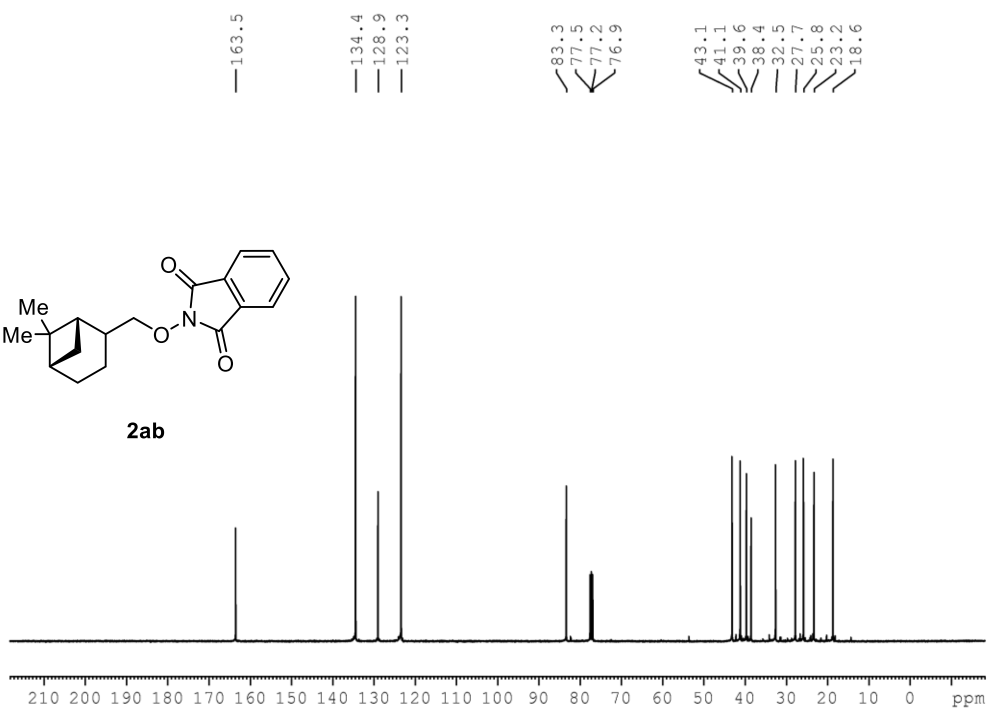

Supplementary Figure 138. ^{13}C NMR spectrum of compound 2ab

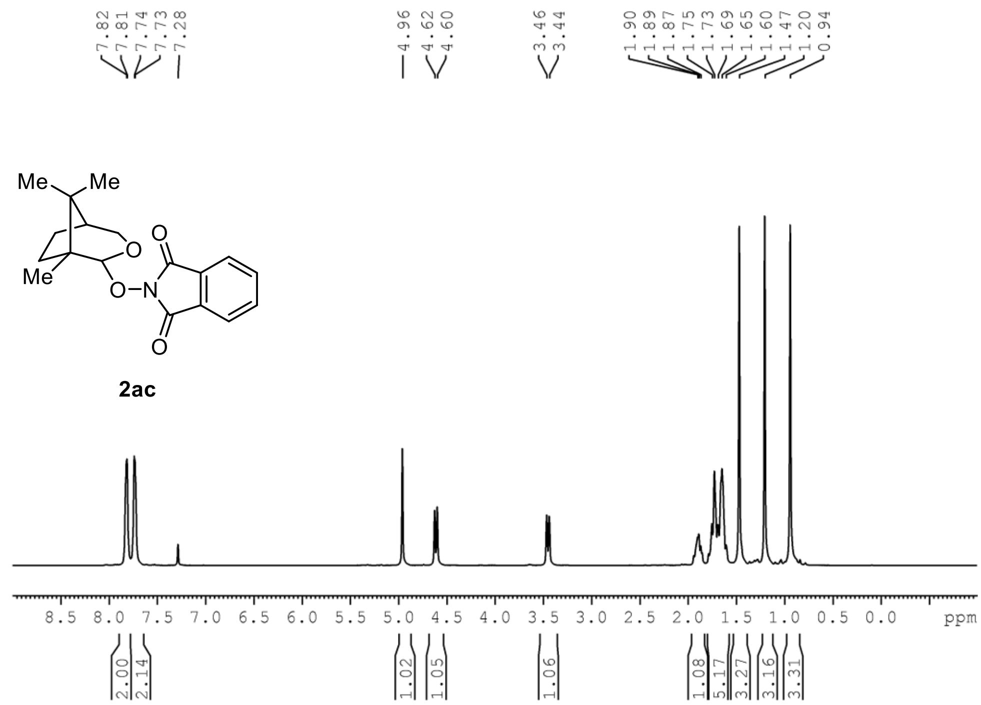

Supplementary Figure 139. ^{1}H NMR spectrum of compound 2ac

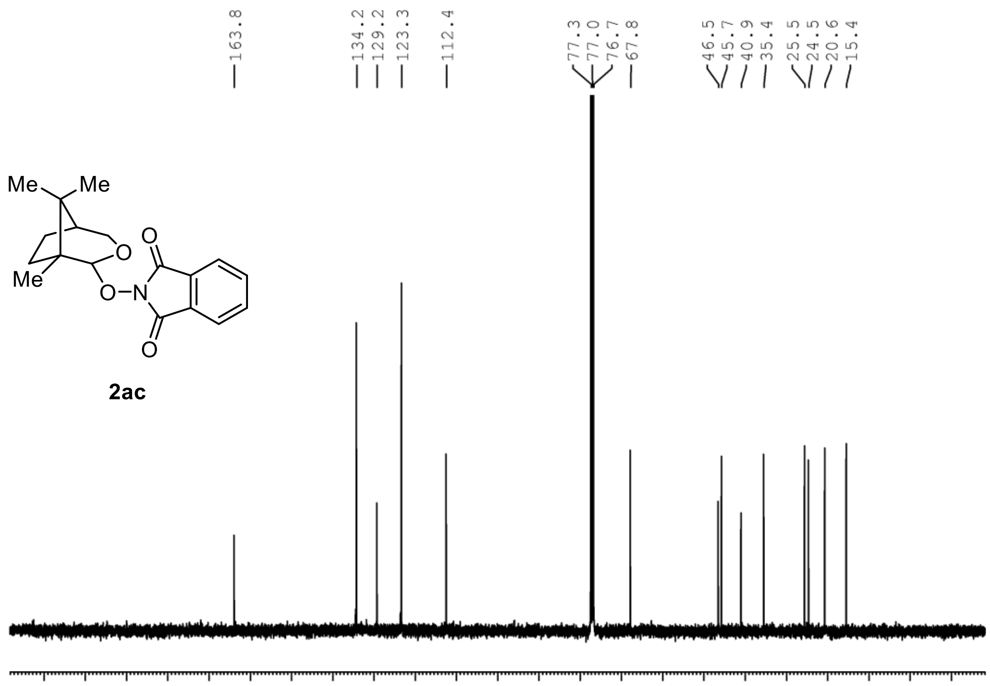

Supplementary Figure 140. ^{13}C NMR spectrum of compound 2ac

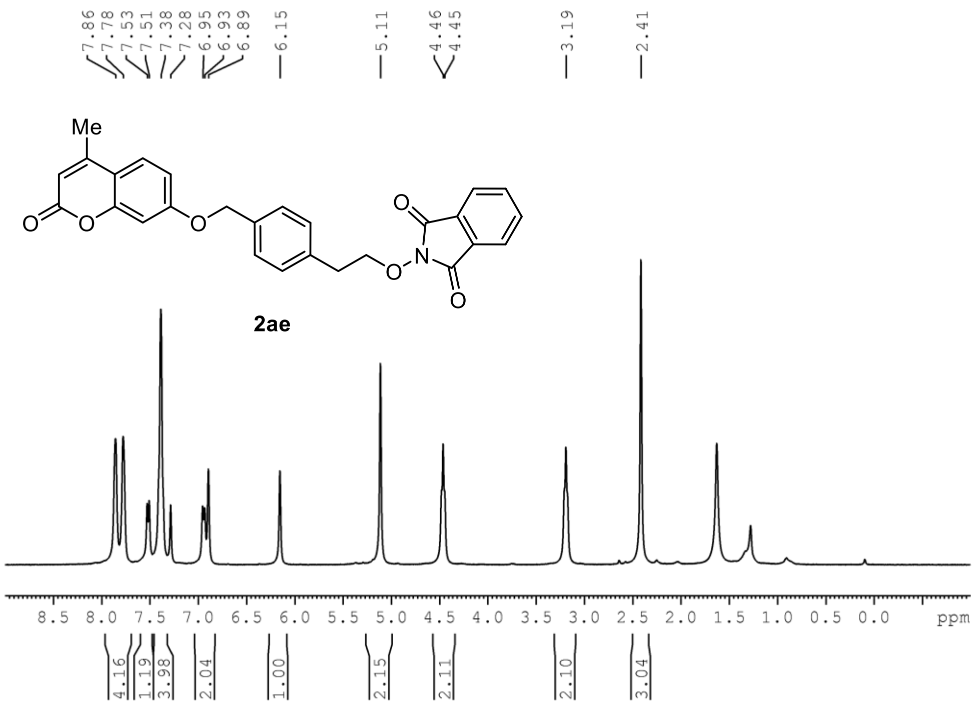

Supplementary Figure 141. ^{1}H NMR spectrum of compound 2ae

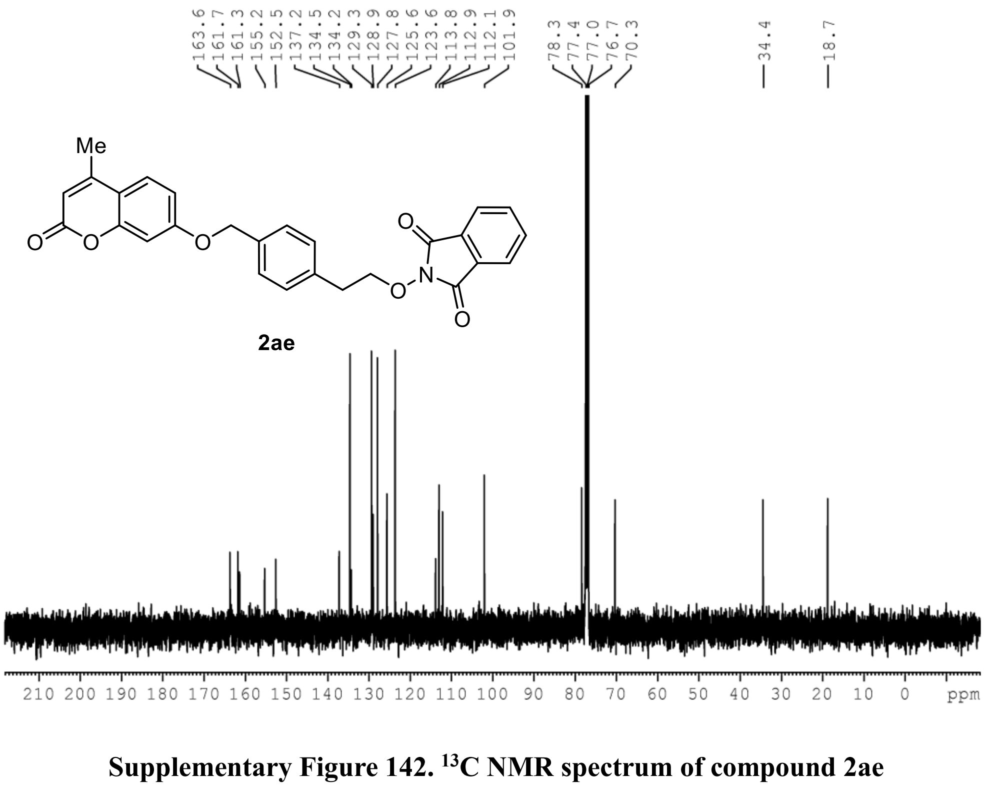

Supplementary Figure 142. ^{13}C NMR spectrum of compound 2ae

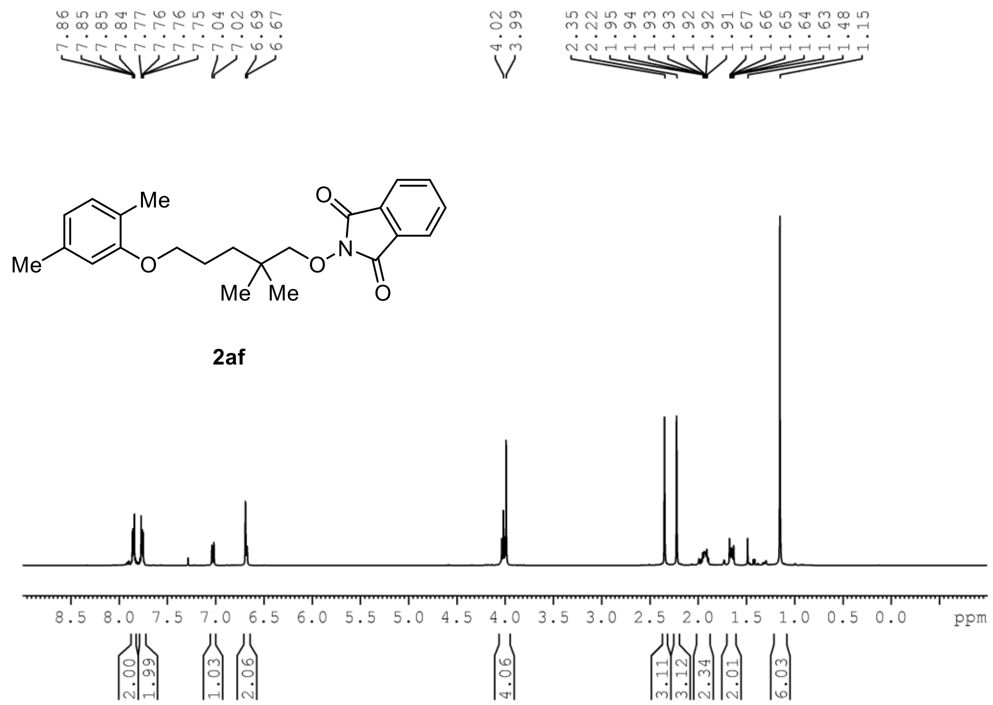

Supplementary Figure 143. ^{1}H NMR spectrum of compound 2af

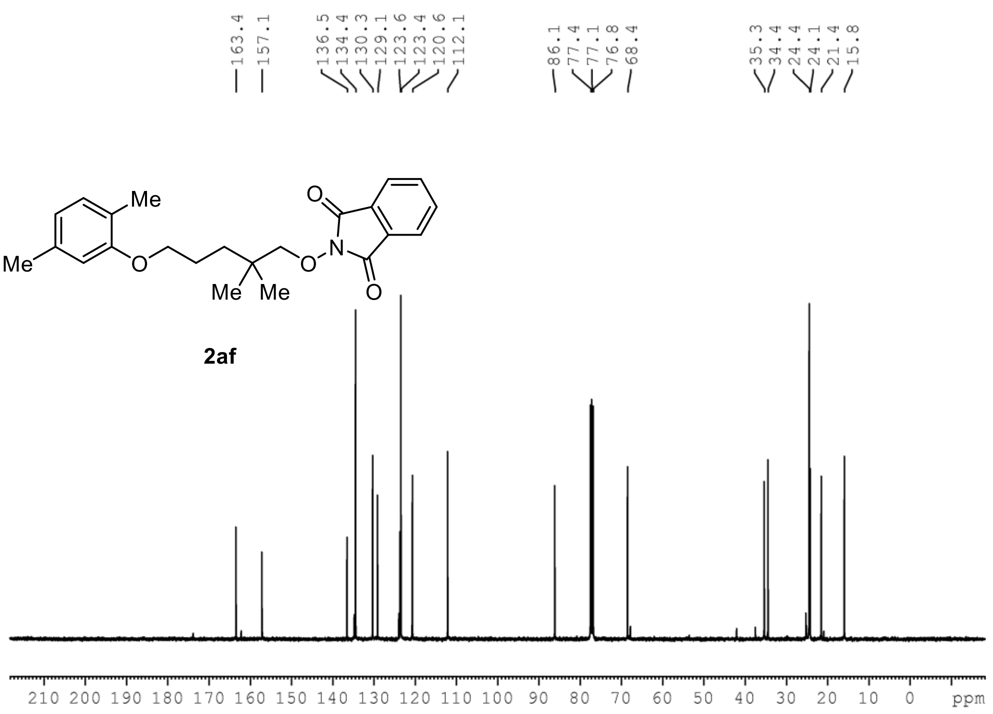

Supplementary Figure 144. ^{13}C NMR spectrum of compound 2af

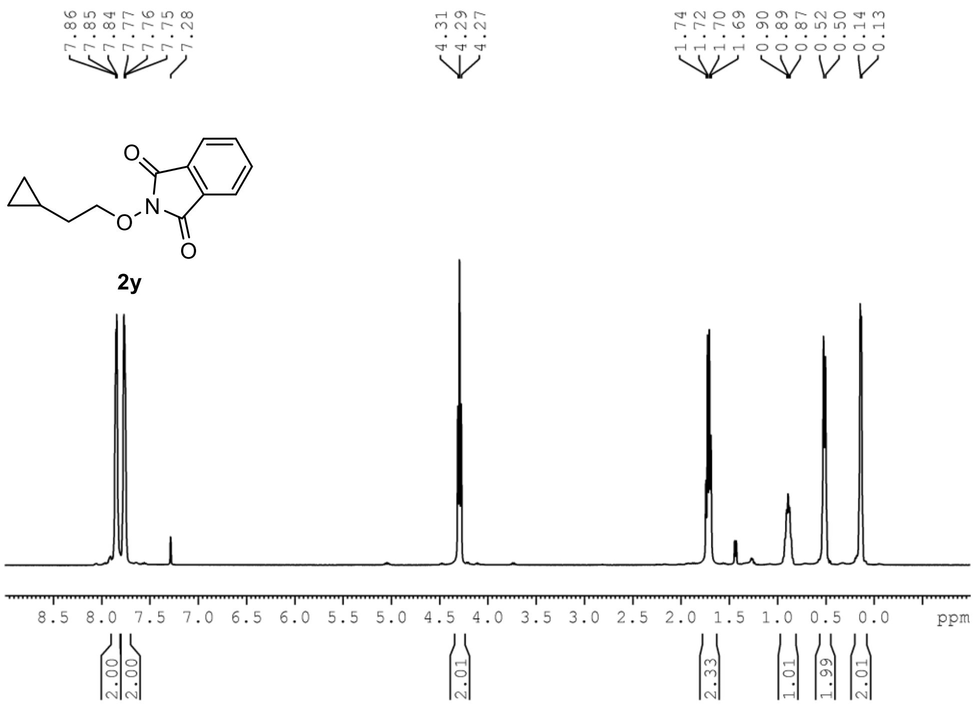

Supplementary Figure 145. ^{1}H NMR spectrum of compound 2y

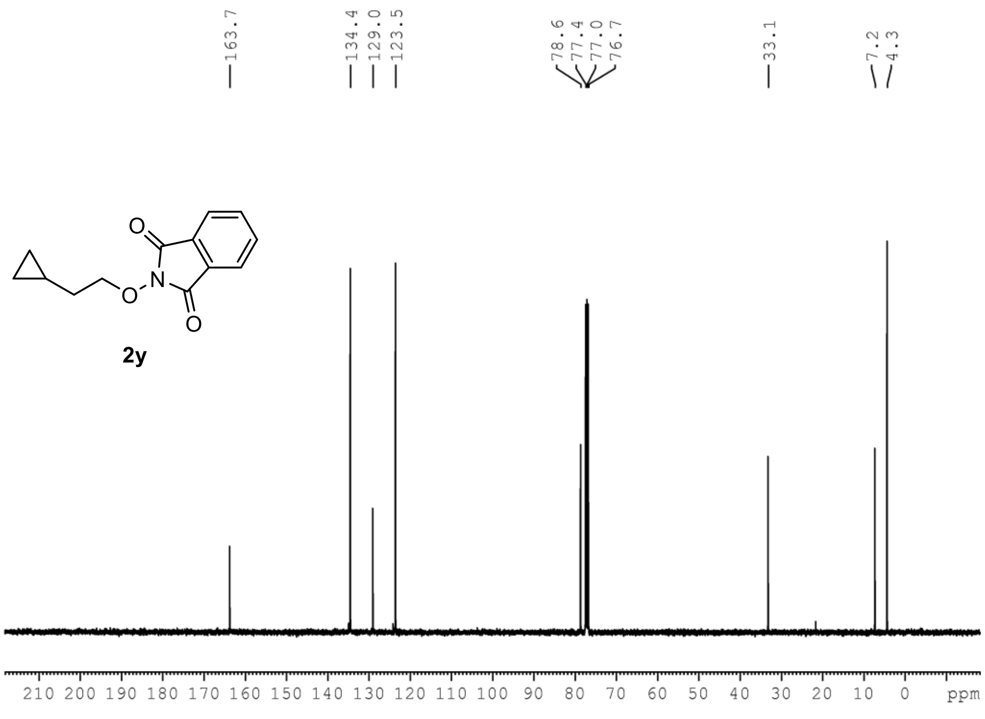

Supplementary Figure 146. ^{13}C NMR spectrum of compound 2y

5. Supplementary Reference

1. Wang, J. *et al.* Visible-light promoted α-alkylation of glycine derivatives with alkyl boronic acids. *Chem. Commun.* **57**, 1959–1962 (2021).
2. Ren, L. & Cong, H. Visible-light-driven decarboxylative alkylation of C-H bond catalyzed by dye-sensitized semiconductor. *Org. Lett.* **20**, 3225–3228 (2018).
3. Zhang, W. *et al.* Mesoionic pyrido[1,2-a]pyrimidinones: a novel class of insecticides inhibiting nicotinic acetylcholine receptors. *Bioorganic Med. Chem. Lett.* **26**, 5444–5449 (2016).
4. LaRochelle, M. C., Thomas, J. L. & Strickler, R. C. Reactivation of human placental 17β, 20α-hydroxysteroid dehydrogenase: affirmation of affinity labeling principles. *Steroids* **43**, 209–217 (1984).
5. Zhu, X., Lin, Y., Sun, Y., Beard, M. C. & Yan, Y. Lead-halide perovskites for photocatalytic α-alkylation of aldehydes. *J. Am. Chem. Soc.* **141**, 733–738 (2019).
6. Jerezano, A. V *et al.* Iodine-mediated one-pot synthesis of indoles and 3-dimethylaminoindoles via annulation of enaminones. *Arkivoc* **2014**, 18–53 (2013).
7. Wang, C. *et al.* Visible-light-driven, copper-catalyzed decarboxylative C(sp^3)−H alkylation of glycine and peptides. *Angew. Chem. Int. Ed.* **57**, 15841–15846 (2018).
8. Zhang, J. *et al.* Donor–acceptor complex enables alkoxyl radical generation for metal-free C(sp^3)–C(sp^3) cleavage and allylation/alkenylation. *Angew. Chem. Int. Ed.* **129**, 12793–12797 (2017).
9. Lei, X., Feng, J., Guo, Q., Xu, C. & Shi, J. Base-promoted formal [3 + 2] cycloaddition of α-halohydroxamates with carbon disulfide to synthesize polysubstituted Rhodanines. *Org. Lett.* **24**, 2837–2841 (2022).
10. Xu, P., Qian, B., Hu, B. & Huang, H. Palladium-catalyzed tandem hydrocarbonylative lactamization and cycloaddition reaction for the construction of bridged polycyclic lactams. *Org. Lett.* **24**, 147–151 (2022).
11. Xu, X., Liu, Y. & Park, C. M. Rhodium(III)-catalyzed intramolecular annulation through C-H activation: Total synthesis of (±)-antofine, (±)-septicine, (±)-tylophorine, and rosettacin. *Angew. Chem. Int. Ed.* **51**, 9372–9376 (2012).
12. Shi, J. L., Wang, Z., Zhang, R., Wang, Y. & Wang, J. Visible-light-promoted ring-opening alkynylation, alkenylation, and allylation of cyclic hemiacetals through β-scission of alkoxy radicals. *Chem. A Eur. J.* **25**, 8992–8995 (2019).
13. Shu, C., Madhavachary, R., Noble, A. & Aggarwal, V. K. Photoinduced fragmentation borylation of cyclic alcohols and hemiacetals. *Org. Lett.* **22**, 7213–7218 (2020).
14. Cong, F., Lv, X. Y., Day, C. S. & Martin, R. Dual catalytic strategy for forging sp^2-sp^3 and sp^3-sp^3 architectures via β-scission of aliphatic alcohol derivatives. *J. Am. Chem. Soc.* **142**, 20594–20599 (2020).
15. Haibach, M. C., Stoltz, B. M. & Grubbs, R. H. Catalytic reduction of alkyl and aryl bromides using propan-2-ol. *Angew. Chem. Int. Ed.* **56**, 15123–15126 (2017).
16. Dixon, D. D., Lockner, J. W., Zhou, Q. & Baran, P. S. Scalable, divergent synthesis of meroterpenoids via 'borono- sclareolide'. *J. Am. Chem. Soc.* **134**, 8432–8435 (2012).
17. Braun, J. C. & Fisher, G. S. Hydroboration of β-pinene. *Tetrahedron Lett.* **1**, 9–11 (1960).
18. Dhoro, F., Kristensen, T. E., Stockmann, V., Yap, G. P. A. & Tius, M. A. Asymmetric

amine-intercepted Nazarov cyclization. *J. Am. Chem. Soc.* **129**, 7256–7257 (2007)

19. Ritter, C. *et al.* Bioorthogonal enzymatic activation of caged compounds. *Angew. Chem. Int. Ed.* **54**, 13440–13443 (2015).
20. Ong, D. Y. *et al.* Controlled reduction of carboxamides to alcohols or amines by zinc hydrides. *Angew. Chem. Int. Ed.* **58**, 4992–4997 (2019).
21. Maercker, A. & Theysohn, W. Versuche zur Umlagerung von 2-cyclopropyl-äthyl-anionen. *Justus Liebigs Ann. Chem.* **759**, 132–157 (1972).
22. Xia, Q. *et al.* Visible light-driven α-alkylation of *N*-aryl tetrahydroisoquinolines initiated by electron donor-acceptor complexes. *Org. Lett.* **22**, 7290–7294 (2020).
23. Nguyen, H. N. *et al.* Synthesis of 4-substituted chlorophthalazines, dihydrobenzoazepinediones, 2-pyrazolylbenzoic acid, and 2-pyrazolylbenzohydrazide via 3-substituted 3-hydroxyisoindolin-1-ones. *J. Org. Chem.* **77**, 3887–3906 (2012).
24. Kang, T., Kim, H., Kim, J. G. & Chang, S. Synthesis of 1,2-amino alcohols via catalytic C-H amidation of sp^3 methyl C-H bonds. *Chem. Commun.* **50**, 12073–12075 (2014).
25. Dubey, N., Sharma, P. & Kumar, A. Clay-supported Cu(II) catalyst: an efficient, heterogeneous, and recyclable catalyst for synthesis of 1,4-disubstituted 1,2,3-triazoles from alloxan-derived terminal alkyne and substituted azides using click chemistry. *Synth. Commun.* **45**, 2608–2626 (2015).
26. Xuan, J. *et al.* Redox-neutral α-allylation of amines by combining palladium catalysis and visible-light photoredox catalysis. *Angew. Chem. Int. Ed.* **54**, 1625–1628 (2015).
27. Qiao, J. *et al.* Direct, site-selective and redox-neutral α-C−H bond functionalization of tetrahydrofurans via quantum dots photocatalysis. *Angew. Chem. Int. Ed.* **60**, 27201–27205 (2021).
28. Zeng, H. *et al.* Site-specific functionalization of 1,3-dioxolane with imines: a radical chain approach to masked α-amino aldehydes. *J. Org. Chem.* **83**, 5256–5266 (2018).
29. Halland, N. & Jørgensen, K. A. Intermolecular addition of alkyl radicals to imines in the absence and in the presence of a Lewis acid. *J. Chem. Soc. Perkin Trans.1* 1290–1295 (2001).
30. Kitoun, C. *et al.* Phosphine-mediated bioconjugation of the 3’-end of RNA. *Org. Lett.* **22**, 8034–8038 (2020).
